# Synthesis of Chiral, Enantiopure Allylic Amines by the Julia Olefination of α-Amino Esters

**DOI:** 10.3390/molecules21060805

**Published:** 2016-06-21

**Authors:** Fabio Benedetti, Federico Berti, Lidia Fanfoni, Michele Garbo, Giorgia Regini, Fulvia Felluga

**Affiliations:** Department of Chemical and Pharmaceutical Sciences, University of Trieste, via Giorgieri 1. 34127 Trieste, Italy; fberti@units.it (F.B.); lidia.fanfoni@gmail.com (L.F.); garb.michele@gmail.com (M.G.); giorgia.regini@phd.units.it (G.R.)

**Keywords:** chiral pool, allylamines, amino acids, sulfones, acylation

## Abstract

The four-step conversion of a series of *N*-Boc-protected l-amino acid methyl esters into enantiopure *N*-Boc allylamines by a modified Julia olefination is described. Key steps include the reaction of a lithiated phenylalkylsulfone with amino esters, giving chiral β-ketosulfones, and the reductive elimination of related α-acetoxysulfones. The overall transformation takes place under mild conditions, with good yields, and without loss of stereochemical integrity, being in this respect superior to the conventional Julia reaction of α-amino aldehydes.

## 1. Introduction

**Figure 1:**
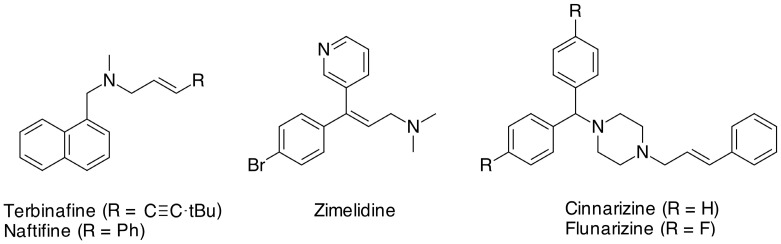
Bioactive compounds based on the allylamine structure.

Allylic amines (allylamines) are compounds of significant synthetic and biological interest [1,2,3]. The allylamine fragment is present in natural products [4,5,6] and other biologically-active compounds [7,8,9], including drugs for the treatment of mycosis (terbinafine, naftifine) [10], depression (zimelidine) [11], motion sickness (cinnarizine) [12], migraine and epilepsy (flunarizine) [13] (Figure 1). 

On the other hand, the characteristic bifunctional unit can undergo a wide range of chemical transformations, thus making allylamines versatile building blocks in organic synthesis [1]. Chiral allylamines, in particular, are valuable intermediates for the synthesis of a variety of chiral, enantiomerically-pure bioactive compounds [14,15,16,17].

Considerable effort has been devoted to the asymmetric synthesis of α-chiral allylamines, resulting in the development of several approaches, among which the transition metal-catalyzed asymmetric allylic amination has been extensively documented [18,19,20]. Other methods include the asymmetric vinylation of imines [21], the Overman rearrangement of allylic imidates [22], the asymmetric hydrogenation of dienamines [23], the transition metal-catalyzed asymmetric allylic C-H amination [24] and the nitroso-ene reaction [25].

In spite of recent advances in asymmetric synthesis, the olefination of α-amino aldehydes from the chiral pool is still an attractive alternative, as the starting materials can be obtained from the corresponding α-amino acids by several routes [26,27,28]. The synthesis of chiral allylamines by the Wittig [29,30,31], Julia [32,33,34] and Peterson [35] olefination of α-amino aldehydes has been reported, but these approaches are often limited by the chemical and configurational instability of α-amino aldehydes [27].

**Scheme 1:**
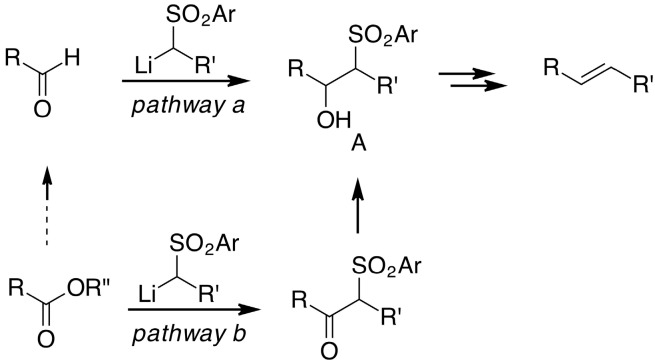
Julia olefination.

In the classical Julia reaction [36,37] (Scheme 1), a lithiated aryl alkyl sulfone is added to an aldehyde to give the corresponding α-hydroxysulfone A (pathway *a*). This, in turn, is converted into the target alkene by in situ acetylation and treatment of the resulting α-acetoxysulfone with sodium or magnesium amalgam [38] or SmI_2_ [39]. The same α-hydroxysulfone A, however, can be obtained in two steps by the acylation of the lithiated phenylalkylsulfone with an ester, followed by the reduction of the corresponding α-ketosulfone (Scheme 1, pathway *b*). Chiral allylamines may thus be obtained starting from readily-available α-amino esters that are chemically and configurationally more stable than α-amino aldehydes [40]; furthermore, the additional step of pathway *b* is not a limitation, as the latter compounds are generally prepared from the corresponding esters in at least one step [26].

**Scheme 2:**
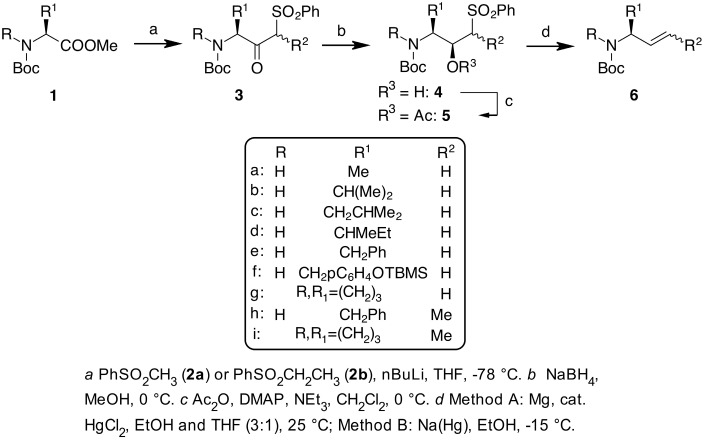
Julia olefination of chiral α-amino esters.

This variant of the Julia reaction has been used in the synthesis of *E*-alkene dipeptide isosteres [41,42], but its wider scope and general applicability is still unexplored. In this paper, we demonstrate that the α-aminoester Julia olefination is a convenient method for the synthesis of terminal and disubstituted allylamines (Scheme 2). 

## 2. Results and Discussion

The synthesis starts with the reaction between *N*-Boc amino esters **1** and lithiated phenylmethylsulfone **2a**, giving the corresponding ketosulfones **3**. The experimental conditions for this key step were initially optimized, with respect to yields and *ee*, using *N*-Boc alanine methyl ester 1a as the substrate (Table 1) [43].

**Scheme 3:**
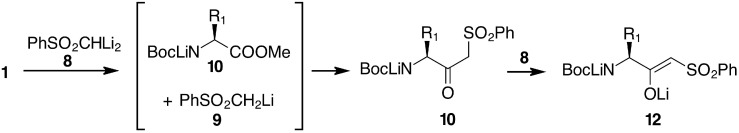
Reactions of mono- and bis-lithiated sulfones.

Addition of **1a** to one equivalent of lithiated phenylmethylsulfone **2a**, even in the presence of two equivalents of *n*-BuLi as originally suggested by Lygo [42,44], resulted in a partial conversion into the ketosulfone **3a** (Table 1, Entries 1 and 2). The low yields are likely due to the presence of ionizable protons, both in the starting amino ester (NHBoc) and in the ketosulfone product (methylene) that can exchange with lithiated **2a**. The yield improved when an excess of BuLi and **2a** was employed (Entries 3 and 4); under these conditions, however, the enantiomeric purity of the ketosulfone **3a** was modest. The use of TMEDA in association with butyllithium [45] (Entry 6) and of other base systems (Entries 7, 8) was not successful. The best results were eventually obtained with two equivalents of sulfone and four equivalents of butyl lithium [46,47], with the resulting yellow suspension added to the amino ester **1a**, at −78 °C under argon. In this case, complete conversion into the ketosulfone **3a** was achieved, which was isolated in excellent yield and enantiomeric purity (Entry 9). It has been suggested that, under similar conditions, the dilithiated sulfone **8** is formed and is the reactive nucleophile [46,47]. It seems more likely, however, that the monolithiated species **9** is formed following H/Li exchange with the protected aminoester (Scheme 3) and is the actual nucleophile. A second equivalent of **8** is then required for a further H/Li exchange giving the dilithiated product **12**, in agreement with the observed stoichiometry.

The optimized reaction conditions were then applied to the *N*-Boc-amino esters **1b**–**f** (Table 2), giving the corresponding ketosulfones **3** in good to excellent yields and *ee*, with the only exception of the protected tyrosine methyl ester **1f**; repeated attempts under several reaction conditions invariably led to a significant loss in enantiomeric purity for this compound. With L-proline methyl ester **1g** (Entry 7), where no ionizable protons are present, the best optical purity, albeit at the expenses of a lower yield, was obtained by using two equivalents of PhSO_2_Me and BuLi with respect to the aminoester, whereas racemization was observed by employing the usual conditions. This further confirms that the reactive nucleophile in the reaction with the amino esters is the monolithiated species PhSO_2_CH_2_Li, as suggested above (Scheme 3).

Finally, the same conditions were applied to the reaction of amino esters **1e** and **1g** with phenylethylsulfone **2b** (Table 2, Entries 8 and 9), giving the corresponding ketosulfones **3h** and **3i**, as mixtures of diastereoisomers, in excellent yield. 

The ketosulfones **3a**–**g** thus obtained were reduced with NaBH_4_ (Scheme 2), giving quantitatively the α-hydroxysulfones **4a**–**g** as mixtures of *syn* (*S,S*) and *anti* (*S*,*R*) diastereoisomers in ratios from approximately 10:1 for branched Compounds **4b**–**d** (Val, Leu, Ile) to 3:1 for compounds having linear side chains **4a,e,f** (Ala, Phe, Tyr) to 1.5:1 for **4g** (Pro), in agreement with previous findings [48]. No attempts were made to optimize the stereoselectivity, as the newly-formed stereogenic center is lost in the final step of the synthesis. Not surprisingly, complex mixtures containing all four possible diastereoisomers were obtained in the reduction of ketosulfones **3h** and **3i**.

Conversion of hydroxysulfones **4** into the allylamines **6** follows the standard protocol of the Julia reaction. Thus, the alcohols **4** were acetylated with acetic anhydride and DMAP, and the corresponding acetoxysulfones **5** were converted to the target aminoalkenes **6** in the final reductive elimination, promoted by sodium amalgam [36] or in situ generated magnesium amalgam [38] (Scheme 2, Table 3). The two methods were shown to proceed with comparable yields and *ee*, except for **5f**: in this case, sodium amalgam caused the deprotection of the phenol group, leading directly to the allylamine **6f′**, while the O-protected product **6f** was smoothly obtained with magnesium amalgam.

The Julia reaction is known to favor the formation of the *E* isomer, when leading to disubstituted alkenes [36,37]. Accordingly, when substituted acetates **5h,i** were subjected to reductive elimination with Na(Hg), the corresponding allylamines **6h,i** were obtained as 4:1 mixtures of *E* and *Z* isomers, as determined by NMR. A complete list of the allylamines synthesized in this work with overall yields and enantiomeric excess is reported in Table 3.

## 3. Experimental Section

Glassware was thoroughly oven-dried for reactions using anhydrous conditions. THF was distilled over sodium benzophenone ketyl, under argon; CH_2_Cl_2_ and EtOH were distilled over CaH_2_ under argon. Yields refer to chromatographically and spectroscopically (^1^H-NMR) homogeneous materials. Reactions were monitored by TLC carried out on silica gel plates using UV light as the visualizing agent and KMnO_4_ as the developing agent. Silica gel 60 (230−400 mesh) was used for flash column chromatography. NMR spectra were recorded on a Varian 500 MHz spectrometer in CDCl_3_, unless otherwise stated, at 500 MHz (^1^H) and 125.68 MHz (^13^C); chemical shifts are in ppm (δ) with chloroform as the reference (δ = 7.26 for ^1^H-NMR and 77.16 for ^13^C-NMR). Coupling constants *J* are given in hertz. ^1^H and ^13^C-NMR resonances were assigned using a combination of DEPT, COSY and HSQC spectra. Infrared spectra were recorded as thin film or Nujol mull on NaCl plates on a Nicolet Avatar FT-IR spectrometer (Thermo Fisher Scientific, Waltham, MA, USA). Melting points are uncorrected. Electrospray (ESI) mass spectra were obtained on a Bruker Daltonics Esquire 4000 spectrometer (Bruker Corporation, Billerica, MA, USA). HRMS analyses were carried out in the ESI mode on a TOF-MS instrument (Bruker Corporation). Enantiomeric excesses were determined by Chiral High Resolution GC analyses on γ- or β-cyclodextrin capillary columns and, for compounds **3f** and **6f** only, by chiral high pressure liquid chromatography on a Cellulose2 column, with a UV detector operating at λ = 280 nm, eluting with nHex/iPrOH 9:1. Optical rotations were measured at 25 °C with a Jasco P-2000 polarimeter (Jasco Inc, Easton, MD, USA). Commercial reagents and solvents were purchased from Sigma-Aldrich (Milan, Italy); PhSO_2_Me and PhSO_2_Et were prepared by reaction of sodium phenylsulfinate with the appropriate iodoalkane according to the literature [49].

### 3.1. General Procedure for the Synthesis of α-Ketosulfones **3**

1.6 M *n*-BuLi in hexane (2.5 mL, 4 mmol for **3a**–**f,h**; 1.25 mL, 2 mmol for **3g,i**) was slowly added at −5 °C, under Ar, to a solution of sulfone **2** (2.0 mmol) in 1.5 mL dry THF, and the mixture was stirred at −5 °C for 30 min (4 h for reactions with sulfone **2b**). The resulting yellow suspension was transferred via a syringe to a three-necked flask, containing a 2.5 M solution of the N-Boc amino ester **1** (1 mmol) in dry THF (1M for **3h,i**), at −78 °C, under an Ar atmosphere, with stirring. The mixture was stirred overnight while the temperature was allowed to reach 25 °C, and partitioned between 5% aqueous citric acid (water for **3f**) and ethyl acetate. The organic phase was extracted with saturated NaHCO_3_ and brine, dried over Na_2_SO_4_ and evaporated to give a solid that was purified by flash chromatography (eluent AcOEt/petroleum ether from 0%–15%). 

### 3.2. Spectroscopic and Analytical Data for α-Ketosulfones **3**

*(3S)-3-(tert-Butoxycarbonylamino)-1-(phenylsulfonyl)-2-butanone* (**3a**) [42]: 89% from **1a** and **2a**; white solid, mp 115–117 °C, ${\left[\alpha \right]}_{D}^{25}$ −36 (*c* 0.8, CHCl_3_); IR: 3387, 1736, 1700, 1288, 1148 cm^−1^; ^1^H-NMR: δ 1.35 (d, *J* = 7.0 Hz, 3H), 1.44 (s, 9H), 4.24 (d, *J* = 14.0 Hz, 1H), 4.33 (m, 1H), 4.44 (d, *J* = 14.0 Hz, 1H), 5.15 (br, 1H), 7.58 (m, 2H), 7.69 (m, 1H), 7.90 (m, 2H) ppm. ^13^C-NMR: δ 16.1, 28.1, 55.9, 63.2, 80.2, 128.4, 129.3, 134.3, 139.0, 155.3, 198.4 ppm; ESI-MS: *m*/*z* 350 [M + Na]^+^.

*(3S)-3-(tert-Butoxycarbonylamino)-4-methyl-1-(phenylsulfonyl)-2-pentanone* (**3b**) [44]: 85% From **1b** and **2a**; white solid, mp 85–88 °C, ${\left[\alpha \right]}_{D}^{25}$ −34 (*c* 1, CHCl_3_); IR: 3372, 1709, 1506, 1323, 1160 cm^−1^; ^1^H-NMR: δ 0.80 (d, *J* = 7.0 Hz, 3H), 0.98 (d, *J* = 7.0 Hz, 3H), 1.44 (s, 9H), 2.29 (m, 1H), 4.20 (d, *J* = 14.5, 1H), 4.25 (dd, *J* = 4.6, 8.7 Hz, 1H), 4.43 (d, *J* = 14.5 Hz, 1H), 5.10 (d, *J* = 8.7 Hz, 1H), 7.57 (m, 2H), 7.67 (m, 1H), 7.93 (m, 2H) ppm; ^13^C-NMR: δ 17.0, 19.9, 28.4, 28.9, 64.0, 65.2, 80.5, 128.6, 129.4, 134.4, 139.1, 156.0, 197.8 ppm; ESI-MS: *m*/*z* 378 [M + Na]^+^, 394 [M + K]^+^.

*(3S)-3-(tert-Butoxycarbonylamino)-5-methyl-1-(phenylsulfonyl)-2-hexanone* (**3c**): 89% From **1c** and **2a**; white solid, mp 76–78 °C; ${\left[\alpha \right]}_{D}^{25}$ −43 (*c* 1.6, CHCl_3_); IR: 3369, 1708, 1511, 1323, 1159 cm^−1^; ^1^H-NMR: δ 0.93 (d, *J* = 6.1 Hz, 3H), 0.94 (d, *J* = 6.1 Hz, 3H), 1.37–1.44 (s and, 10H), 1.67 (m, 2H), 4.21 (d, *J* = 13.9 Hz, 1H), 4.28 (m, 1H), 4.49 (d, *J* = 13.9 Hz, 1H), 5.06 (d, *J* = 7.1 Hz, 1H), 7.58 (t, 2H), 7.67 (t, 1H), 7.94 (dd, *J* = 8.0, 1.5 Hz, 2H) ppm; ^13^C-NMR: δ 21.4, 23.1, 24.8, 28.2, 39.1, 58.9, 63.3, 80.5, 128.4, 129.2, 134.2, 138.9, 155.5, 198.6 ppm; ESI-MS: *m*/*z* 392 [M + Na]^+^, 408 [M + K]^+^. Anal. Calcd. for C_18_H_27_NO_5_S: C, 58.51; H, 7.37; N, 3.79. Found C, 58.50; H, 7.43; N, 3.86.

*(3S,4S)-3-(tert-Butoxycarbonylamino)-4-methyl-1-(phenylsulfonyl)-2-hexanone* (**3d**): 86% from **1d** and **2a**; white solid, mp 83–85 °C;$\;{\left[\alpha \right]}_{D}^{25}$ –0.2 (*c* 0.9, CHCl_3_); IR: 3368, 1708, 1504, 1323, 1156 cm^−1^; ^1^H-NMR: δ 0.87 (t, *J* = 7.1 Hz, 3H), 0.95 (d, *J* = 6.5 Hz, 3H), 1.05 (m, 1H), 1.27 (m, 1H), 1.42 (s, 9H), 1.99 (m, 1H), 4.20 (d, 1H), 4.27 (dd, *J* = 5.0, 8.7 Hz, 1H), 4.43 (d, *J* = 14.5 Hz, 1H), 5.08 (d, *J* = 8.7 Hz, 1H), 7.56 (m, 2H), 7.67 (m, 1H), 7.92 (m, 2H) ppm. ^13^C-NMR: δ 11.6, 16.1, 24.3, 28.4, 35.7, 64.1, 65.0, 80.5, 128.6, 129.4, 134.3, 139.1, 155.9, 197.9 ppm. ESI-MS: *m*/*z* 392 [M + Na]^+^, 408 [M + K]^+^. Anal. Calcd. for C_18_H_27_NO_5_S: C, 58.51; H, 7.37; N, 3.79. Found C, 58.42; H, 7.40; N, 3.91.

*(3S)-3-(tert-Butoxycarbonylamino)-4-phenyl-1-(phenylsulfonyl)-2-butanone* (**3e**) [44]: 94% from **1e** and **2a**; white solid, mp 137–139 °C; ${\left[\alpha \right]}_{D}^{25}$ –22 (*c* 1, CHCl_3_); IR: 3386, 1732, 1691, 1295, 1147 cm^−1^; ^1^H-NMR: δ 1.38 (*s*, 9H), 2.91 (dd, *J* = 8.5, 14.0 Hz, 1H), 3.16 (dd, *J* = 5.5, 14.0 Hz, 1H), 4.22 (d, *J* = 14.0 Hz, 1H), 4.35 (d, *J* = 14.0 Hz, 1H), 4.47 (m, 1H), 5.12 (d, *J* = 6.0 Hz, 1H), 7.15 (m, 2H), 7.23–7.31 (m, 3H), 7.56 (m, 2H), 7.67 (m, 1H), 7.86 (m, 2H) ppm; ^13^C-NMR: δ 28.2, 36.3, 61.4, 63.7, 80.6, 127.1, 128.4, 128.8, 129.3, 134.3, 136.2, 138.8, 155.4, 197.4 ppm; ESI-MS: *m*/*z* 426 [M + Na]^+^, 442 [M + K]^+^.

*(3S)-3-(tert-Butoxycarbonylamino)-4-[4-(tert-butyldimethylsilyloxy)phenyl]-1-(phenylsulfonyl)-2-butanone* (**3f**): 78% from **1f** and **2a**; *ee* 81%; white solid, mp 111–113 °C; ${\left[\alpha \right]}_{D}^{25}$ −21(*c* 1, CHCl_3_); IR: 3369, 1709, 1609, 1584, 1511 cm^−1^; ^1^H-NMR: δ 0.19 (s, 6H), 0.98 (s, 9H), 1.39 (s, 9H), 2.86 (dd, *J* = 8.6, 14.6 Hz, 1H), 3.18 (dd, *J* = 5.7, 14.6 Hz, 1H), 4.17 (d, *J* = 13.9 Hz, 1H), 4.32 (d, *J* = 13.9 Hz, 1H), 4.42 (*m*, 1H), 5.04 (d, *J* = 6.4 Hz, 1H), 6.77 (d, *J* = 8.0 Hz, 2H), 7.00 (d, *J* = 8.0 Hz, 2H), 7.57 (t, *J* = 7.8 Hz, 2H), 7.68 (t, *J* = 7.8 Hz, 1H), 7.87 (d, *J* = 7.8 Hz, 2H) ppm; ^13^C NMR: δ −4.4, 18.2, 25.6, 28.2, 35.7, 61.5, 63.9, 80.6, 120.4, 128.4, 128.5, 129.2, 130.9, 134.2, 138.8, 154.8, 197.6 ppm; ESI-MS: *m*/*z* 556.2 [M + Na]^+^, 572 [M + K]^+^. Anal. Calcd. for C_27_H_39_NO_6_SSi: C, 60.76; H, 7.37; N, 2.62. Found C, 60.81; H, 7.38; N, 2.55.

*1-((2S)-1-(tert-Butoxycarbonyl)-2-pyrrolidinyl)-2-(phenylsulfonyl)-1-ethanone* (**3g**): 60% from **1g** and **2a**; yellow oil; ${\left[\alpha \right]}_{D}^{25}$ −43.1 (*c* 1.6, CHCl_3_); IR: 1698, 1693, 1323, 1157 cm^−1^; ^1^H-NMR (mixture of rotamers): δ 1.36 and 1.43 (2s, 9H), 1.85–1.96 (m, 2H), 2.00–2.24 (m, 2H), 3.46 and 3.55 (m, 2H), 4.18–4.42 (m, 3H), 7.58 (m, 2H), 7.67 (m, 1H), 7.94 (m, 2H) ppm; ^13^C-NMR (Mixture of rotamers): δ 23.9, 24.8, 28.4 and 28.5, 47.0 and 47.2, 62.8 and 64.1, 65.7 and 66.3, 80.6 and 81.1, 128.6, 129.3 and 129.4, 134.2 and 134.4, 139.3, 153.7 and 155.0, 198.1 and 198.6 ppm; HRMS (ESI) *m*/*z* Calcd. for C_17_H_23_NNaO_5_S [M + Na]^+^ 376.1195, found 376.1192. 

*(2S,4S) and (2S,4R)-2-(tert-Butoxycarbonylamino)-1-phenyl-4-(phenylsulfonyl)-3-pentanone* (**3h**): A 3:2 mixture of two diastereoisomers (*a* and *b*) was obtained from **1e** and **2b** in 77% yield; white solid; IR: 3390, 1737, 1700, 1286, 1150 cm^−1^; ^1^H-NMR: δ 1.28 and 1.37 (2d, 3H), 1.39 (s, 5.4H), 1.44 (s, 3.6H), 2.85 (dd, *J* = 9.6, 14.0 Hz, 0.67H), 3.02 (dd, *J* = 7.8, 13.8 Hz, 0.33H), 3.22 (dd, *J* = 5.0, 13.8 Hz, 0.33H), 3.33 (dd, *J* = 5.0, 14.0 Hz, 0.67H), 4.40–4.55 (m, 1H), 4.65–4.75 (m, 1H), 4.97 (d, 0.67H), 5.40 (d, 0.33H), 7.17–7.35 (m, 5H), 7.52–7.77 (m, 5H) ppm; ^13^C-NMR: δ 12.1 (*b*), 12.5 (*a*), 28.0 (*a*), 28.1 (*b*), 35.0 (*a*), 37.7 (*b*), 61.5 (*a*), 61.6 (*b*), 65.9 (*b*), 66.5 (*a*), 80.2 (*b*), 80.7 (*a*), 126.8 (*a*), 127.1 (*b*), 128.3 (*b*), 128.7, 129.1, 129.2, 129.3, 129.5, 129.6, 129.7, 134.3, 134.5, 135.9, 136.0, 136.3, 137.3, 155.4, 155.7, 200.8, 202.2 ppm; ESI-MS: *m*/*z* 440 [M + Na]^+^, 456 [M + K]^+^. Anal. Calcd. for C_22_H_27_NO_5_S: C, 63.29; H, 6.52; N, 3.35. Found C, 63.23; H, 6.38; N, 3.38.

*(2S) and (2R) 1-((2S)-1-(tert-Butoxycarbonyl)-2-pyrrolidinyl)-2-(phenylsulfonyl)-1-propanone* (**3i**): A 55:45 mixture of two diastereoisomers (*a* and *b*) was obtained from **1g** and **2b** in 80% yield. Yellow oil; IR: 1700, 1689, 1322, 1157 cm^−1^; ^1^H-NMR (*d*_6_-DMSO, 80 °C): δ 1.30 (d, *J* = 7.4, 1.65H), 1.34 (d, *J* = 7.4 Hz, 1.35H), 1.35 (s, 4.95H), 1.41 (s, 4.05H), 1.59–2.25 (m, 4H), 3.28–3.45 (m, 2H), 4.55 (dd, *J* = 3.0, 8.0 Hz, 0.55H), 4.67 (br, 0.45H), 4.73 (m, 0.45H), 4.85 (br, 0.55H), 7.67 (m, 2H), 7.78 (m, 1H), 7.88 (m, 2H) ppm; ^13^C-NMR (d_6_-DMSO, 80 °C): δ 12.1 (*b*), 12.3 (*a*), 22.7 (*a* and *b*), 27.6 (*a* or *b*), 27.7 (*a* or *b*), 46.1 (*b*), 46.3 (*a*), 64.3, 64.7, 65.7, 78.8 (*a*), 79.0 (*b*), 128.5, 128.6 and 128.8, 133.7 (*b*), 133.8 (*a*), 137.0, 153.0, 201.6 ppm; HRMS (ESI) *m*/*z* Calcd. for C_18_H_25_NNaO_5_S [M + Na]^+^ 390.1351, found 390.1342. 

### 3.3. General Procedure for the Synthesis of Allylamines **6** from Ketosulfones ***3***

NaBH_4_ (0.04 g, 1 mmol) was added portion-wise, at 0 °C, to a solution of ketone **3** (1 mmol) in methanol (5 mL). When the reaction was complete (TLC), the solvent was removed *in vacuo*; aqueous workup and extraction with ethyl acetate gave the β-hydroxysulfones **4** as mixtures of diastereoisomers, whose ratio was determined by NMR. To the crude mixtures of diastereoisomeric alcohols, in the minimum amount of dry dichloromethane, were added, in the order, 2-dimethylaminopyridine (0.01 mmol), triethylamine (10 mmol) and acetic anhydride (5 mmol), at 0 °C with stirring. The reaction mixture was stirred at room temperature until the formation of the product was complete (TLC: AcOEt/petroleum ether 2:8), extracted with 10% aqueous citric acid, saturated NaHCO_3_ and brine, dried over Na_2_SO_4_ and evaporated. If necessary, co-evaporation with toluene was performed to completely remove the excess anhydride. The crude acetates **5** were obtained in quantitative yield and were used in the final step without further purification. Where possible, analytical data are given for the main diastereoisomer obtained in pure form by crystallization (vide infra). Allylamines **6** were finally obtained from β-acetoxysulfones **5** following two methods. Method A: in a Schlenk reactor filled with Ar, Mg powder (2.5 mmol) and HgCl_2_ (0.1 mmol) were added, in the order, to a 0.2 M solution of sulfone **5** (1mmol, mixture of diastereoisomers) in 3:1 EtOH/THF. The reaction mixture was stirred at room temperature monitoring the progress by TLC (petroleum ether/diethyl ether 8:2); 10% aqueous citric acid was added, and the aqueous phase was extracted with ethyl acetate. The combined organic layers were washed with brine, dried over Na_2_SO_4_ and evaporated. The crude product was purified by column chromatography using 9:1 petroleum ether/Et_2_O as the eluent. Method B: in a round-bottomed flask, 5% Na/Hg amalgam (3 mmol of Na) was added, at −15 °C, under an Ar atmosphere, to a stirred 0.5 M solution of sulfone **5** (1 mmol) in anhydrous, degassed ethanol. The reaction mixture was stirred at room temperature monitoring the progress by TLC (petroleum ether/diethyl ether 8:2); water was added, and the workup as above gave the crude allylamines **6**.

### 3.4. Spectroscopic and Analytical Data for Alcohols ***4***, Acetates ***5*** and Allylamines ***6***

*(3S)-3-(tert-Butoxycarbonylamino)-1-butene* (**6a**) [50]: 68% via alcohol **4a** and acetate **5a** (Method A). **4a**: 3:1 mixture of *syn* (*s*) and *anti* (*a*) diastereoisomers. Pale yellow oil; IR: 3370, 1693, 1304, 1146 cm^−1^; ^1^H-NMR: δ 1.13 (d, *J* = 8.5 Hz, 2.25H), 1.21 (d, *J* = 8.5 Hz, 0.75H), 1.40 (s, 9H), 3.21 (m, 1.5H), 3.26 (m, 0.5H), 3.64 (br, 2H), 4.10 (br, 1H), 4.73 (br, 1H), 7.59 (m, 2H), 7.69 (m, 1H), 7.92 (m, 2H) ppm; ^13^C-NMR: δ 15.4 (*s*), 18.1 (*a*), 28.3, 50.1, 59.8 (*s*), 60.5 (*s*), 68.5 (*s*), 68.9 (*s*), 79.9, 127.3 (*s*), 127.9 (*s*), 129.3 (*a*), 129.4 (*s*), 133.7 (*a*), 134.1 (*s*), 139.1, 155.5 ppm; ESI-MS: *m*/*z* 296 [M + Na]^+^, 368 [M + K]^+^. **5a**: 3:1 mixture of *syn* (*s*) and *anti* (*a*) diastereoisomers. White solid, mp 110–111 °C; IR: 3358, 1742, 1712, 1521 cm^−1^; ^1^H-NMR: δ 1.06 (d, *J* = 7.0 Hz, 0.75H), 1.09 (d, *J* = 7.0 Hz, 2.25H), 1.40 (s, 6.75H), 1.42 (s, 2.25H), 1.82 (s, 2.25H), 1.95 (s, 0.75H), 3.37 (m, 2H), 3.86 (br, 1H), 4.43 (br, 0.75H), 4.52 (br, 0.25H) 5.21 (br, 0.75H), 5.34 (br, 0.25H), 7.57 (m, 2H), 7.66 (m, 1H), 7.91 (m, 2H) ppm; ^13^C-NMR: δ 16.3 (*s*), 18.1 (*a*), 20.6, 28.3, 48.3 (*s*) 49.4 (*a*), 56.7 (*s*), 57.8 (*a*), 69.5 (*a*), 70.2 (*s*), 80.0, 128.3, 129.3, 133.9, 139.2, 155.1, 169.8 ppm; ESI-MS: *m*/*z* 394 [M + Na]^+^. **6a**: oil, *ee* 95% (by GLC); ${\left[\alpha \right]}_{D}^{25}$–6.0 (*c* 0.45, CHCl_3_); ^1^H-NMR: δ 1.20 (d, *J* = 8.5 Hz, 3H), 1.44 (s, 9H), 4.22 (br, 1H), 4.45 (br, 1H), 5.10 (m, 2H), 5.81 (m, 1H) ppm; ^13^C NMR: δ 20.7, 28.4, 48.1, 79.3, 113.6, 140.1, 155.1 ppm; ESI-MS: *m*/*z* 194 [M + Na]^+^.

*(3S)-3-(tert-Butoxycarbonylamino)-4-methyl-1-pentene* (**6b**) [51]: 60% via alcohol **4b** and acetate **5b** (method A). **4b**: 10:1 mixture of *syn* (*s*) and *anti* (*a*) diastereoisomers. Yellow oil; IR: 3368, 1691, 1304, 1146 cm^−1^; ^1^H-NMR (*syn* diastereoisomer): δ 0.87 (d, *J* = 7.0 Hz, 3H), 0.90 (d, *J* = 7.0 Hz, 3H), 1.40 (s, 9H), 2.18 (m, 1H), 3.23 (m, 1H), 3.32 (m, 1H), 3.48 (m, 1H), 3.56 (d, *J* = 2.9 Hz, 1H) 4.12 (m, 1H), 4.41 (d, *J* = 9.7 Hz, 1H), 7.59 (m, 2H), 7.68 (m, 1H), 7.92 (m, 2H) ppm. ^13^C-NMR ((2*S*,3*S*) diastereoisomer): δ 15.7, 20.0, 27.1, 28.3, 58.5, 60.4, 67.4, 80.1, 128.1, 129.7, 134.4, 139.6, 156.7 ppm; ESI-MS: *m*/*z* 380 [M + Na]^+^, 396 [M + K]^+^. **5b** (*syn* isomer): white solid, mp: 98–100 °C; ${\left[\alpha \right]}_{D}^{25}$ −5 (*c* 0.6, CHCl_3_); IR: 3365, 1744, 1712, 1523 cm^-1^; ^1^H-NMR: δ 0.86 (d, *J* = 8.5 Hz, 3H), 0.90 (d, *J* = 8.5 Hz, 3H), 1.41 (s, 9H), 1.62 (m, 1H), 1.75 (s, 3H), 3.33 (d, *J* = 18.3 Hz, 1H), 3.44 (dd, *J* = 12.5, 18.3 Hz, 1H), 3.63 (m, 1H), 4.35 (d, *J* = 13.0 Hz, 1H), 5.29 (m, 1H), 7.57 (m, 2H), 7.66 (m, 1H), 7.90 (m, 2H) ppm; ^13^C-NMR: δ 17.1, 19.6, 20.6, 28.3, 56.6, 57.3, 68.1, 79.9, 127.4, 128.3, 129.1, 133.9, 139.1, 155.6, 169.7 ppm; ESI-MS: *m*/*z* 422 [M + Na]^+^, 438 [M + K]^+^. **6b**: oil, *ee* > 99.5% (by GLC); ${\left[\alpha \right]}_{D}^{25}$ + 28 (*c* 0.85, CHCl_3_), lit. [33] + 25 (CHCl_3_); ^1^H-NMR: δ 0.88 (d, *J* = 7 Hz, 3H), 0.90 (d, *J* = 7 Hz, 3H), 1.44 (s, 9H), 1.77 (m, 1H), 3.98 (br, 1H), 4.50 (br, 1H), 5.12 (m, 2H), 5.73 (m, 1H) ppm. ^13^C-NMR: δ 18.2, 18.8, 28.6, 32.4, 58.1, 79.3, 115.2, 137.5, 155.7 ppm; ESI-MS: *m*/*z* 222 [M + Na]^+^, 237 [M + K]^+^.

*(3S)-3-(tert-Butoxycarbonylamino)-5-methyl-1-hexene* (**6c**) [52]: 67% via alcohol **4c** and acetate **5c** (method A). **4c**: 7:1 mixture of *syn* (*s*) and *anti* (*a*) diastereoisomers. Orange oil; IR: 3368, 1694, 1305, 1146 cm^−1^; ^1^H-NMR: δ 0.85 (d, *J* = 6.7 Hz, 3H), 0.90 (d, *J* = 6.7 Hz, 3H), 1.31 (m, 2H), 1.39 (s, 9H), 1.60 (m, 1H), 3.25 (m, 1.76H), 3.27 (m, 0.24H), 3.49 (br, 0.12H), 3.56 (br, 0.88H), 3.74, (br, 0.12H), 3.78 (br, 0.88H), 4.10 (m, 1H), 4.55 (br d, *J* = 7.3 Hz, 0.88H), 4.72 (br d, *J* = 9.0 Hz, 0.12H), 7.57 (t, 2H), 7.66 (t, 1H), 7.92 (d, 2H) ppm. ^13^C-NMR: δ 21.5 (*s*), 22.0 (*a*), 23.0 (*a*), 23.6 (*s*) 24.6 (*s*), 24.7 (*a*), 28.2 (*s*), 28.3 (*a*), 38.9 (*s*), 41.3 (*a*), 52.6 (*a*), 52.9 (*s*), 59.8 (*s*), 60.3 (*a*), 67.8 (*s*), 69.4 (*a*), 79.6 (*s*), 79.8 (*a*), 127.9 (*s*), 128.0 (*a*), 129.4 (*s*), 129.5 (*a*), 134.0 (*a*), 134.1 (*s*), 134.2 (*a*), 139.2 (a), 139.3 (*s*), 156.1 ppm. ESI-MS: *m*/*z* 394 [M + Na]^+^, 410 [M + K]^+^. **5c**: 7:1 mixture of *syn* (*s*) and *anti* (*a*) diastereoisomers. White solid; IR: 3372, 1745, 1707, 1519 cm^-1^; ^1^H-NMR: δ 0.86 (d, *J* = 6.6 Hz, 3H), 0.91 (d, *J* = 6.6 Hz, 3H), 1.13–1.25 (m, 2H), 1.41 (s, 7.9H) 1.43 (s, 1.1H), 1.60 (m, 1H), 1.78 (s, 2.6H), 1.96 (s, 0.4H), 3.35 (m, 1H) 3.44 (m, 1H), 3.76 (m, 0.13H), 3.89 (br, 0.87H), 4.33 (d, *J* = 10.0 Hz, 0.9H), 4.45 (d, *J* = 10.0 Hz, 0.1H), 5.18 (m, 0.9H), 5.37 (dbt, *J* = 9.4, 2.5 Hz, 0.1H), 7.58 (t, 2H), 7.67 (t, 1H), 7.91 (dd, *J* = 8.0, 1.5 Hz, 2H) ppm. ^13^C-NMR: δ 20.6, 21.6 (*s*), 21.8 (*a*), 23.1 (*a*), 23.2 (*s*), 24.6 (*a*), 24.7 (*s*), 28.2 (*s*), 28.3 (*a*), 39.5 (*s*), 41.1 (*a*), 50.6 (*s*), 52.0 (*a*), 56.3 (*s*), 57.8 (*a*), 69.1 (*a*), 70.1 (*s*), 79.8 (*a*), 79.9 (*s*) 128.26 (*s)*, 128.33 (*a*), 129.27 (*s*) 129.33 (*a*), 133.8, 139.3, 155.4, 169.7 ppm. ESI-MS: *m*/*z* 436 [M + Na]^+^, 452 [M + K]^+^. **6c**: oil, *ee* > 99.5% (by GLC); ${\left[\alpha \right]}_{D}^{25}$ +3.5 (*c* 1.0, CHCl_3_); ^1^H-NMR: δ 0.89 (d, *J* = 6.7 Hz, 3H) 0.90 (d, *J* = 6.7 Hz, 3H), 1.31 (m, 2H), 1.42 (s, 9H), 1.65 (sept, 1H), 4.12 (br, 1H), 4.62 (br, 1H), 5.03 (d, *J*_=_ 9.9 Hz, 1H), 5.12 (d, *J* = 15.8 Hz, 1H), 5.71 (ddd, *J* = 6.9, 9.9, 15.8 Hz, 1H) ppm; ^13^C NMR: δ 22.0, 22.7, 24.4, 28.1, 44.7, 51.0, 79.2, 113.9, 139.4, 155.1 ppm. ESI-MS: *m*/*z* 236 [M + Na]^+^.

*(3S,4S)-3-(tert-Butoxycarbonylamino)-4-methyl-1-hexene* (**6d**) [53]: 69% via alcohol **4d** and acetate **5d** (method A). **4d**: 94:6 mixture of *syn* (*s*) and *anti* (*a*) diastereoisomers. Pale yellow oil; IR: 3370, 1693, 1304, 1146 cm^−1^; ^1^H-NMR: δ 0.85 (t, 0.18H), 0.90 (d, *J* = 7.0 Hz, 3H), 0.92 (t, *J* = 7.0 Hz, 0.82H), 0.99 (m, 1H), 1.39 (s, 9H), 1.54 (m, 1H), 1.85 (m, 1H), 3.27 (m, 2H), 3.50 (m, 1H), 3.58 (br, 1H), 4.18 (m, 1H), 4.40 (d, *J* = 9.5 Hz, 0.94H), 4.84 (d, *J* = 10.0 Hz, 0.06H), 7.58 (m, 2H), 7.68 (m, 1H), 7.92 (m, 2H) ppm. ^13^C-NMR: δ 11.1 (*a*), 11.8 (*s*), 15.6 (*a*), 16.3 (*s*), 23.1 (*s*), 25.7 (*a*), 28.4, 31.0 (*a*) 34.2 (*s*), 58.8 (*a*), 59.0 (*s*), 60.4 (*s*), 60.7 (*a*), 65.2 (*s*), 67.2 (*s*), 80.1, 127.5 (*a*), 128.0 (*s*), 129.4 (*a*), 129.6 (*s*), 133.8 (*a*), 134.2 (*s*), 139.6, 156.6 ppm; ESI-MS: *m*/*z* 394 [M + Na]^+^, 410 [M + K]^+^. **5d**: 94:6 Mixture of *syn* (*s*) and *anti* (*a*) diastereoisomers. White solid, mp 108–110 °C; ${\left[\alpha \right]}_{D}^{25}$ −3.8 (*c* 1.5, CHCl_3_); IR: 3367, 1746, 1708, 1518 cm^−1^; ^1^H-NMR: δ 0.84 (t, *J* = 7.2 Hz, 3H), 0.89 (d, *J* = 6.5 Hz, 3H), 1.00 (m, 1H) 1.30 (m, 1H), 1.41 (s, 8.5H), 1.43 (s, 0.5H), 1.50 (m, 1H), 1.73 (s, 2.8H), 1.98 (s, 0.2H), 3.29 (d, *J* = 15Hz, 1H), 3.43 (dd, *J* = 9.5, 15.0 Hz, 1H), 3.68 (m, 1H), 4.31 (d, 1H, *J* = 10.0 Hz, 0.94H), 4.55 (d, *J* = 10.5 Hz, 0.06H), 5.34 (dt, *J* = 6.3, 1.2 Hz, 0.94H), 5.55 (dt, *J* = 9.3, 2.2 Hz, 0.06H), 7.57 (m, 2H), 7.66 (m, 1H), 7.90 (m, 2H) ppm. ^13^C NMR (*syn* diastereoisomer): δ 10.8, 15.5, 20.6, 24.2, 27.9, 35.1, 56.3, 56.4, 67.9, 80.0, 128.1, 129.1, 133.6, 139.0, 155.5, 169.7 ppm; ESI-MS: *m*/*z* 436 [M + Na]^+^, 452 [M + K]^+^. **6d**: oil, *ee* > 99.5% (by GLC). ${\left[\alpha \right]}_{D}^{25}$ + 32 (*c* 1.0, CHCl_3_). ^1^H NMR: δ 0.85 (d, *J* = 6.5 Hz, 3H,), 0.89 (t, *J* = 7.5 Hz, 3H), 1.10 (m, 1H), 1.43 (s, 9H), 1.40–1.53 (m, 2H), 4.05 (br, 1H), 4.54 (br, 1H), 5.12 (m, 2H), 5.70 (m, 1H) ppm; ^13^C-NMR: δ 11.7, 15.0, 25.3, 28.4, 38.6, 56.9, 78.9, 115.2, 136.8, 155.5 ppm; ESI-MS: *m/z* 236 [M + Na]^+^, 252 [M + K]^+^.

*(3S)-3-(tert-Butoxycarbonylamino)-4-phenyl-1-butene* (**6e**) [54]: 70% via alcohol **4e** and acetate **5e** (method B). **4e**: 3:1 mixture of *syn* (*s*) and *anti* (*a*) diastereoisomers. White solid, mp 130–131 °C; IR: 3370, 1692, 1304, 1146 cm^−1^; ^1^H-NMR: δ 1.30 (s, 6.75H), 1.35 (s, 2.25H), 2.78–3.01 (m, 2H), 3.16–3.33 (m, 2H), 3.62–3.92 (multiplets, 2H), 4.07 (br, 0.25H), 4.14 (br, 0.75H), 4.55 (br, 0.75H), 4.93 (d, *J* = 9.5 Hz, 0.25H), 7.16–7.29 (m, 5H), 7.51 (m, 0.5H), 7.57 (m, 1.5H), 7.64 (m, 0.25H), 7.68 (m, 0.75H), 7.82 (m, 0.5H), 7.89 (m, 1.5H) ppm. ^13^C NMR: δ 28.1 (*s*), 28.2 (*a*), 35.8 (*s*), 38.3 (*a*), (55.1 (*s*), 55.8 (*a*), detected in HSQC only) 59.8 (*s*), 60.3 (*a*), 65.7 (*a*), 68.1 (*s*), 79.8 (*s*) 80.0 (*a*), 126.5 (*s*), 126.7 (*s*), 127.8 (*a*) 127.9 (*s*), 128.4 (*a*), 128.5 (*s*), 129.2 (*s*), 129.3 (*a*), 129.4 (*a*), 129.5 (*s*), 134.1 (*a*), 134.2 (*s*), 136.9, 139.1, 155.7 ppm; ESI-MS: *m/z* 428 [M + Na]^+^. **5e** (*syn* isomer): White solid, mp 122–124 °C; ${\left[\alpha \right]}_{D}^{25}$ −7 (*c* 1, CHCl_3_); IR: 3336, 1732, 1705 cm^−1^; ^1^H-NMR: δ 1.28 (s, 9H), 1.84 (s, 3H), 2.61 (m, 1H), 2.84 (dd, *J* = 5.5, 14.0 Hz, 1H), 3.41 (m, 2H), 4.08 (br, 1H), 4.40 (m, 1H), 5.30 (m, 1H), 7.12–7.29 (m, 5H), 7.52–7.57 (m, 2H), 7.66 (m, 1H), 7.81–7.89 (m, 2H) ppm. ^13^C NMR: δ 20.6, 28.1, 36.8, 53.5, 56.7, 69.7, 80.0, 126.8, 128.2, 128.6, 129.0, 129.3, 133.8, 136.5, 139.3, 155.3, 169.8 ppm; ESI-MS: *m/z* 470 [M + Na]^+^, 486 [M + K]^+^. **6e**: white solid, mp 72–74 °C; *ee* > 99.5%. ${\left[\alpha \right]}_{D}^{25}$ + 15 (*c* 0.85, CHCl_3_); ^1^H-NMR: α 1.44 (s, 9H), 2.83 (d, *J* = 6.3 Hz, 2H), 4.47 (br, 2H), 5.08 (m, 2H), 5.80 (m, 1H), 7.17–7.29 (m, 5H) ppm; ^13^C NMR: δ 28.3, 41.3, 53.3, 79.1, 114.5, 126.2, 128.1, 129.7, 137.1, 137.9, 155.3 ppm; ESI-MS: *m/z* 270 [M + Na]^+^.

*(3S)-3-(tert-Butoxycarbonylamino)-4-[4-(tert-butyldimethylsilyloxy)phenyl]-1-butene* (**6f**): 76% via alcohol **4f** and acetate **5f** (method A). **4f**: 5:1 mixture of *syn* (*s*) and *anti* (*a*) diastereoisomers. White solid, mp 107–109 °C; IR: 3369, 1693, 1610, 1511, 1256, 1145 cm^−1^; ^1^H-NMR: δ 0.17 (s, 7.5H) and 0.18 (s, 1.5H), 0.97 (s, 5H) and 0.98 (s, 1H), 1.32 (s, 7.5 H) and 1.35 (s, 1.5H), 2.67–2.95 (m, 2H), 3.14–3.35 (m, 2H), 3.56–3.92 (m, 2H), 4.05 (br, 0.17H) and 4.11 (br, 0.83H), 4.51 (br, 0.83H) and 4.89 (d, *J* = 8.6, 0.17H), 6.71 (d, 0.36H) and 6.75 (d, 1.64H), 7.01 (overlapping d, 2H), 7.61 (m, 2H), 7.67 (m, 1H), 7.83 (d, 0.36H) and 7.90 (d, 1.64H), ppm.^13^C-NMR: δ −4.45, 18.2, 25.7, 28.2, 35.0 (*s*), 37.5 (*a*), 55.3 (*s*), 55.9 (*a*), 59.9 (*s*), 60.4 (*a*) 65.7 (*a*), 68.1 (*s*), 79.7, 120.0 (*a*), 120.1 (*s*), 127.8 (*a*), 127.9 (*s*), 129.4, 130.1, 130.4, 134.1, 139.1, 154.4 (*s*), 155.7 (*a*) ppm. ESI-MS: *m/z* 558.2 [M + Na]^+^, 574.2 [M + K]^+^. **5f** (*syn* isomer): White solid, mp 159–161 °C; ${\left[\alpha \right]}_{D}^{25}$ −6.7 (*c* 1.5, CHCl_3_), IR: 3369, 1747, 1705, 1609, 1512 cm^−1^; ^1^H-NMR: δ 0.16 (s, 6H), 0.96 (s, 9H), 1.30 (s, 9H) 1.80 (s, 3H), 2.45 (m, 1H), 2.84 (m, 1H), 3.43 (m, 2H), 4.04 (br, 1H), 4.48 (br, 1H), 5.29–5.34 (m, 1H) 6.73 (d, *J* = 8.3 Hz, 2H), 6.98 (d, *J* = 8.3 Hz, 2H), 7.56 (t, 2H), 7.65 (m, 1H), 7.88 (d, *J* = 7.5 Hz, 2H) ppm; ^13^C-NMR: δ −4.5, 18.2, 20.6, 25.6, 28.1, 35.9, 53.5, 56.6, 69.7, 79.9, 120.1, 128.2, 129.0, 129.3, 129.9, 133.8, 139.3, 154.4, 155.3, 169.8 ppm; ESI-MS: *m/z* 600 [M + Na]^+^, 616.2 [M + K]^+^. **6f**: white solid, mp 51–53 °C; *ee* = 80% (by HPLC); ${\left[\alpha \right]}_{D}^{25}\;$+ 1.2 (*c* 1, CHCl_3_); IR 2957, 2930, 2858, 1702, 1610, 1510 cm^−1^; ^1^H-NMR: δ 0.19 (s, 6H), 0.98 (s, 9H), 1.41 (s, 9H), 2.76 (m, 2H), 4.36 (br, 1H), 4.47 (br, 1H), 5.06 (dt, *J* = 10.3, 1.2 Hz, 1H), 5.09 (dt, *J* = 17.4, 1.2 Hz, 1H), 5.78 (ddd, *J* = 10.3, 17.4, 5.5 Hz, 1H), 6.76 (d, 2H), 7.02 (d, 2H) ppm; ^13^C-NMR: δ −4.8, 18.0, 25.7, 28.4, 40.4, 79.0, 114.5, 119.9, 130.0, 130.1, 130.4, 138.2, 154.2 ppm. ESI-MS: *m/z* 400 [M + Na]^+^. Anal. Calcd for C_21_H_35_NO_3_Si: C, 66.80; H, 9.34; N, 3.71. Found: C, 66.88; H, 9.26; N, 3.73.

*(3S)-3-(tert-Butoxycarbonylamino)-4-[4-(hydroxy)phenyl]-1-butene* (**6f′**): 81% from **5f** by method B. White solid, mp 82–84 °C; ${\left[\alpha \right]}_{D}^{26}$ +21.5 (*c* 1.5, CHCl_3_); IR = 3339, 1683, 1615, 1516 cm^−1^; ^1^H-NMR: δ 1.41 (s, 9H), 2.75 (m, 2H), 4.35 (br, 1H), 4.50 (br, 1H), 5.07 (dd, *J* = 10.3, 1.2 Hz, 1H), 5.10 (dd, *J* = 17.4, 1.2 Hz, 1H), 5.60 (br, 1H), 5.78 (ddd, *J* = 17.4, 10.3, 5.5 Hz, 1H), 6.75, (d, 2H), 7.01 (d, 2H) ppm; ^13^C-NMR: δ 28.4, 40.6, 53.7, 79.7, 114.7, 115.3, 129.0, 130.6, 138.1, 154.6, 155.5 ppm; ESI-MS: *m/z* 286.1 [M + Na]^+^, 302 [M + K]^+^. Anal. Calcd for C_15_H_21_NO_3_: C, 68.42; H, 8.04; N, 5.32; O, 18.23; Found: C, 68.41; H, 8.10; N, 5.24.

*(2S)-1-(tert-Butoxycarbonyl)-2-vinylpyrrolidine* (**6g**) [35]: 84% via alcohol **4g** and acetate **5g** (method B). **4g**: 3:2 mixture of *syn* (*s*) and *anti* (*a*) diastereoisomers. Pale yellow oil; IR: 3493, 1687, 1305, 1148 cm^−1^; ^1^H-NMR (*d*_6_-DMSO, 80 °C): δ 1.38 (s, 5.4H), 1.39 (s, 3.6H), 1.66–1.92 (m, 2H), 3.08–3.40 (m, 2H), 3.66 (m, 0.6H), 3.80 (m, 0.4H), 4.25 (br, 1H), 4.84 (d, *J* = 6.0 Hz, 0.4H), 4.91 (d, *J* = 6.5 Hz, 0.6H), 7.63 (m, 2H), 7.70 (m, 1H), 7.89 (m, 2H) ppm; ^13^C-NMR (*d*_6_-DMSO, 80 °C): δ 22.9 (*a*), 24.7 (*s*), 26.2 (*s*), 27.6 (*a*), 27.8 (*s*), 30.0 (*a*), 46.2 (*s*), 46.7 (*a*), 58.8 (*a*), 59.8 (*s*), 60.4 (*a*), 60.8 (*s*), 65.6 (*s*), 66.8 (*a*), 78.1 (*s*), 78.3 (*a*), 126.3 (*a*), 127.1 (*s*), 128.6 (*s*), 128.8 (*a*), 132.8 (*a*), 132.9 (*s*), 140.26 (*a*), 140.27 (*s*), 153.5 (*s*), 153.8 (*a*) ppm; ESI-MS: *m/z* 378 [M + Na]^+^, 394 [M + K]^+^. **5g** (*syn* isomer): white solid, mp 140–143 °C; ${\left[\alpha \right]}_{D}^{25}$ –33.1 (*c* 1.25, CHCl_3_) IR: 1747, 1692, 1520 cm^−1^; ^1^H-NMR (*d*_6_-DMSO, 80 °C): δ 1.38 (s, 9H), 1.67–1.87 (m, 4H), 1.76 (s, 3H), 3.04 (m, 1H), 3.31 (m, 1H), 3.60, 3.65 (part AB of an ABX system, *J* = 4.1, 7.9, 15.0 Hz, 2H), 3.84 (br, 1H), 5.52 (m, 0.5H), 5.73 (m, 1H), 7.65 (m, 2H), 7.74 (m, 1H), 7.87 (m, 2H) ppm; ^13^C-NMR (*d*_6_-DMSO, 80 °C): δ 19.5, 22.4, 24.7, 27.2, 45.7, 55.6, 58.8, 66.9, 78.0, 127.2, 128.2, 133.0, 139.1, 153.2, 168.1 ppm; ESI-MS: *m/z* 378 [M + Na]^+^, 394 [M + K]^+^. **6g**: oil, *ee* > 99.5% (by GLC); ${\left[\alpha \right]}_{D}^{25}$ −15 (*c* 0.75, CHCl_3_), lit. [18] −15.7 (*c* 0.55, CHCl_3_]; ^1^H-NMR (rotamers): δ 1.43 (s, 9H), 1.69 (m, 1H), 1.77–1.79 (m, 2H), 2.02 (m, 1H), 3.38 (m, 2H), 4.27 (br, 1H), 5.04 (m, 2H), 5.73 (m, 1H) ppm; ^13^C-NMR (rotamers): δ 22.6 and 23.2, 28.3, 31.4 and 31.8, 46.0 and 46.4, 59.0, 79.0, 113.6, 138.8 and 139.0, 154.8 ppm; ESI-MS: *m/z* 220 [M + Na ]^+^.

*(4S)-4-(tert-Butoxycarbonylamino)-5-phenyl-1-butene* (**6h**): 8:2 mixture of *E* and *Z* isomers, 60% via alcohol **4h** and acetate **5h** (method B). **4h**: mixture of 4 diastereoisomers in approximately 1:1:3:3 ratio. White solid; IR: 3372, 1704, 1297, 1142 cm^−1^; ^1^H-NMR: δ [1.03, 1.06, 1.20 (d, 3H)], [1.23, 1.29, 1.31, 1.36 (s, 9H)], 2.75–3.15 (multiplets, 2H, CH_2_), 3.20–3.30 (m, 1H, C*H*(CH_3_)), [3.70, 3.85, 3.98 (multiplets, 1H, C*H*(NH))], [3.80, 4.06, 4.15 (multiplets, 1H, C*H*(OH))], [4.38, 4.50 (br, 1H, O*H*)], [4.85, 4.99, 5.05 (d, 1H, N*H*)], 7.20–7.30 (m, 5H), 7.53–7.73 (m, 3H), 7.80–7.91 (m, 2H). ^13^C-NMR: δ [7.1, 11.0, 11.4, 11.7 (*C*H_3_CH)], [28.0, 28.17, 28.22, 28.25 (tBu)], [34.2, 36.8, 38.9, 39.8 (*C*H_2_)], [52.4, 52.7, 53.0, 54.8 (*C*HNH)], [60.6, 63.1, 63.4, 63.6 (*C*HSO_2_Ph)], [69.1, 69.4, 70.0, 72.3 (*C*HOH)], [79.4, 79.7, 79.8 (tBu)], [126.4–137.7 (*C*Ar)], [155.1, 155.4 (*C*O)]; ESI-MS: *m/z* 442 [M + Na]^+^, 458 [M + K]^+^. **5h**: White solid, mixture of four diastereoisomers. IR: 3369, 1747, 1704, 1513 cm^−1^. ^1^H-NMR: δ [1.12, 1.16, 1.24, 1.28 (s, 9H, tBu), [1.17, 1.44 (d, 3H, C*H*_3_)], [1.99, 2.02, 2.28, 2.35 (s, 3H, COC*H*_3_)], 2.45–2.90 (m, 2H, C*H*_2_), 3.38 (m, 1H, C*H*SO_2_Ph), 3.90–4.10 (m, 1H, C*H*NH), [4.64, 4.67, 4.77, 4.82 (m, 1H, N*H*)], 5.25–5.40 (m, 1H, C*H*OAc), 7.07–7.90 (m, 5H), 7.45–7.60 (m, 3H), 7.76–7.83 (m, 2H) ppm; ^13^C-NMR: δ [10.8, 12.0, 13.0, 14.2, 20.9, 21.2, 22.1, 22.8, 27.9, 28.0, 28.3, 28.4 (*C*H_3_)], [36.4, 37.6, 39.8, 40.7 (*C*H_2_)], [52.3, 52.7, 53.1, 54.6 (*C*HNH)], [60.2, 60.7, 61.1, 61.3 (*C*HSO_2_Ph)], [70.5, 71.7, 71.9, 73.9 (*C*HOAc)], [79.8, 80.1, 80.5, (tBu)], 126.4–138.2 (*C*Ar), [155.0, 155.4, 155.5 (*C*O)], [170.0, 170.1, 170.3 (*C*O)] ppm; ESI-MS: *m/z* 484 [M + Na]^+^, 500 [M + K]^+^. **6h**: Pale yellow oil, IR: 3340, 1692, 1390, 1365, 1255 cm^−1^; ^1^H-NMR: α 1.40 (s, 7.2H), 1.42 (s, 1.8H), 1.47 (d, *J* = 7.5 Hz, 0.6H), 1.65 (d, *J* = 7.4 Hz, 2.4H), 2.65–2.95 (m, 2H), 4.23–4.66 (br, 2H), 5.21 (m, 0.2 H), 5.30 (m, 0.8H), 5.52 (m, 1H), 7.10–7.34 (m, 5H) ppm; ^13^C-NMR: δ 13.1 (*Z*), 17.7 (*E*), 28.36 (*Z*), 28.37 (*E*), 42.0, 53.2, 79.3, 126.2, 126.3, 128.17 (*E*) 128.20 (*Z*), 129.6 (*E*), 129.7 (*Z*), 130.9, 137.7 (*E*), 137.8 (*Z*), 155.0 (*Z*), 155.1 (*E*) ppm; HRMS (ESI) *m/z* Calcd, for C_16_H_23_NNaO_2_ [M + Na]^+^ 284.1626, found 284.1628.

*1-[(2S)-1-(tert-Butoxycarbonyl)-2-pyrrolidinyl]-1-propene* (**6i**). 85:15 mixture of *E* and *Z* isomers, 65% via alcohol **4i** and acetate **5i** (method B). **4i**: mixture of diastereoisomers. Yellow oil; IR: 3420, 1687, 1302, 1148 cm^−1^; ^1^H-NMR (major diastereoisomer) δ: 1.37 (d, 3H), 1.44 (s, 9H), 1.70–1.90 (m, 3H), 2.18 (m, 1H), 3.30 (m, 1H), 3.37 (m, 1H), 3.50 (m, 1H), 3.73 (m, 1H), 4.40 (m, 1H), 5.18 (m, 1H), 7.53 (m, 2H), 7.64 (m, 1H), 7.93 (m, 2H) ppm; ^13^C-NMR (major diastereoisomer) δ: 13.2, 24.3, 27.8, 29.9, 48.1, 60.1, 63.8, 77.4, 80.3, 129.0, 130.1, 134.1, 157.5 ppm; ESI-MS: *m/z* 392 [M + Na]^+^, 408 [M + K]^+^. **5i**: yellow oil, mixture of diastereoisomers. IR: 3496, 1744, 1691 cm^−1^; ^1^H-NMR (major diastereoisomer): δ 1.40 (br, 12H, C*H*_3_), 1.76 (s, 3H, C*H*_3_CO), 1.80–2.05 (m, 4H, C*H*_2_), 3.07–3.67 (m, 3H, C*H*_2_N and C*H*SO_2_Ph), 4.2–4.4 (m, 1H, C*H*N), 5.12 (m, 1H, C*H*OAc), 7.53 (m, 2H), 7.64 (m, 1H), 7.93 (m, 2H); ^13^C-NMR (major diastereoisomer): δ 11.9 (*C*H_3_), 21.1 (*C*H_3_CO), 23.7 (*C*H_2_), 28.4 (tBu), 29.4 (*C*H_2_), 46.9 (*C*H_2_N), 56.9 (*C*HN), 61.8 (*C*HSO_2_Ph), 73.4 (*C*HOAc), 79.5 (tBu), 128.8, 129.0, 133.5, 139.0, 155.4, 170.2 ppm; ESI-MS: *m/z* 434 [M + Na]^+^, 450 [M + K]^+^. **6i**: pale yellow oil, IR: 1695, 1394, 1365, 1252 cm^−1^; ^1^H-NMR (*d*_6_-DMSO, 80 °C): δ 1.39 (s, 7.65H), 1.40 (s, 1.35H), 1.51 (m, 0.15 H), 1.60–1.65 (m, 0.85H), 1.64 (d, 3H), 1.75 (m, 2H), 1.94 (m, 0.85H), 2.04 (m, 0.15H), 3.20–3.34 (m, 2H), 4.14 (m, 0.85H), 4.48 (m, 0.15H), 5.30–5.50 (m, 2H); ^13^C-NMR (*d*_6_-DMSO, 80 °C): δ 12.2 (*Z*), 16.6 (*E*), 22.3 (*Z*), 22.8 (*E*), 27.8 (*E,Z*), 31.3 (*E*), 32.0 (*Z*), 45.5 (*E*), 45.6 (*Z*), 53.3 (*Z*), 57.5 (*E*), 77.5 (*E*), 77.6 (*Z*), 122.3 (*Z*), 123.6 (*E*), 131.9 (*E*), 132.5 (*Z*), 153.2 (*E,Z*). HRMS (ESI) *m/z* Calcd. for C_12_H_21_NNaO_2_ [M + Na]^+^ 234.1470, found 234.1477.

## 4. Conclusions

We have developed a general procedure for the synthesis of α-chiral allylamines from readily available α-amino esters, by a simple modification of the Julia olefination reaction. While retaining all of the advantages of the original Julia reaction, this approach avoids using amino aldehydes as precursors in favor of the more readily-available and chemically and configurationally more stable amino esters. Epimerization at the chiral center, which is a frequent problem in the standard Julia and Wittig olefination of N-protected α-amino aldehydes, is thus prevented, and the desired allylamines are obtained in excellent yield and enantiomeric excess. The approach described here for the synthesis of allylamines is quite general and should provide a useful alternative to the classical Julia reaction whenever the starting aldehyde is unstable or, otherwise, not readily accessible. Moreover, its scope might be further expanded by replacing the phenyl alkyl sulfones of this work with benzotriazolylalkyl sulfones or other heteroarylalkyl sulfones, as in the Julia–Kocienski reaction [55,56], where the intermediate hydroxysulfones directly collapse to alkenes, thus avoiding the final reduction step.

## Figures and Tables

**Table 1:**
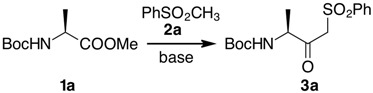
Optimized synthesis of ketosulfone **3a**.

Entry	2a (equiv.)	Base (equiv.)	T (°C)	Yield ^a^ %	*ee* ^a,b^ %
1	1	*n*-BuLi (2)	−78	55	<70
2	1	*n*-BuLi (2)	−30	66	<70
3	1	*n*-BuLi (3)	−78	86	<70
4	2	*n*-BuLi (4)	−78	89	65
5	2	*n*-BuLi (4)	−30	42	<70
6	2	*n*-BuLi/TMEDA (4)	−78	-	-
7	2	KHMDS	−78	-	-
8	2	NaH	−78	-	-
9	2	*n*-BuLi (4)	−78	89 ^c^	95 ^c^

^a^ By addition of **1a** to lithiated **2a**; ^b^ *ee* determined after conversion to the allylamine **6a**; ^c^ By the reverse addition of a suspension of lithiated **2a** to **1a**.

**Table 2:**
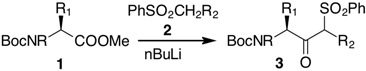
Reactions between amino esters **1** and lithiated sulfones **2** ^a^.

Entry	Aminoester	Sulfone	R_2_	Product	Yield%
1	Ala (**1a**)	**2a**	H	**3a**	89
2	Val (**1b**)	**2a**	H	**3b**	84
3	Leu (**1c**)	**2a**	H	**3c**	89
4	Ile (**1d**)	**2a**	H	**3d**	86
5	Phe (**1e**)	**2a**	H	**3e**	94
6	Tyr (**1f**) ^b^	**2a**	H	**3f**	80
7	Pro (**1g**)	**2a**	H	**3g**	60 ^c^
8	Phe (**1e**)	**2b**	Me	**3h**	77 ^d^
9	Pro (**1g**)	**2b**	Me	**3i**	80 ^c,e^

^a^ Reactions were carried out with 2 equivalents of bis-lithiated sulfone (PhSO_2_CLi_2_R); ^b^ Protected as O-TBDMS (see Scheme 2); ^c^ With 2 equivalents of mono-lithiated PhSO_2_CHRLi; ^d^ 3:2 mixture of diastereoisomers; ^e^ 55:45 mixture of diastereoisomers.

**Table 3:** Synthesis of allylamines **6**.

Allylamine		Method ^a^	Yield ^b^ %	*ee* ^c^ %	Allylamine		Method ^a^	Yield ^b^ %	*ee ^c^* %
**6a**	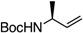	A (1h)	68 (60)	95	**6f**	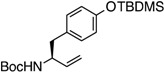	A ^d^ (2h)	65 (52)	80 ^e^
**6b**	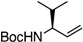	A (2h)	60 (50)	>99	**6f′**	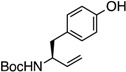	B ^d^ (4h)	71 (57)	ND
**6c**	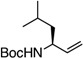	A (2h)	67 (62)	>99	**6g**	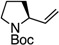	B (2h)	84 (50)	>99
**6d**	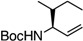	A (3h)	69 (59)	>99	**6h**	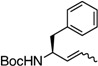	B (4h)	71 ^f^ (55)	ND
**6e**	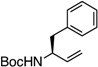	A (2h)	68 (64)	>99	**6i**	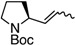	B (12h)	65 ^g^ (52)	ND
		B (4h)	71 (67)	>99					

^a^ Method A: Mg powder (2.5 eq), HgCl_2_ (0.1 equiv.), EtOH/THF (3:1), 25 °C; Method B: 5% Na/Hg (3 equiv.), EtOH, −15 °C; ^b^ Overall yields from amino esters **1** in brackets; ^c^ By chiral HRGC; ^d^ From O-TBDMS protected sulfone 5f; ^e^ By chiral HPLC; ^f^ 80:20 mixture of *E* and *Z* isomers; ^g^ 85:15 mixture of *E* and *Z* isomers.

## Supplementary Materials

Supplementary materials are available online at http://www.mdpi.com/1420-3049/21/6/805/s1.

## Abbreviations

The following abbreviations are used in this manuscript:

Boc: *t*-Butoxycarbonyl
*n*-BuLi: *n*-Butyllithium
COSY: Correlation Spectroscopy
DEPT: Distortionless Enhancement by Polarization Transfer
*ee*: Enantiomeric Excess
ESI: Electrospray Ionization
HRGC: High Resolution Gas Chromatography
HPLC: High Performance Liquid Chromatography
HRMS: High Resolution Mass Spectrometry
HSQC: Heteronuclear Single Quantum Coherence Spectroscopy
IR: Infrared
KHMDS: Potassium Hexamethyldisilazanide
MS: Mass Spectrometry
NMR: Nuclear Magnetic Resonance
TBDMS: *t*-Butyldimethylsilyl
THF: Tetrahydrofuran
TLC: Thin Layer Chromatography
TMEDA: Teramethylethylenediamine
