# Supplementary material for: Synthesis of Chiral, Enantiopure Allylic Amines by the Julia Olefination of α-Amino Esters
Source: Molecules. 2016 Jun 21;21(6):805. doi: 10.3390/molecules21060805 (PMC6273449; doi:10.3390/molecules21060805)
Supplement: Supplementary file 1 [file molecules-21-00805-s001.pdf]

Supplementary Materials: Synthesis of Chiral, Enantiopure Allylic Amines by the Julia Olefination of α-Amino Esters

Fabio Benedetti, Federico Berti, Lidia Fanfoni, Michele Garbo, Giorgia Regini and Fulvia Felluga

Table of Contents

^{1}H and ^{13}C-NMR Spectra for Compounds 3–6……………………………………..p. S2

Chiral GC Chromatograms for Compounds 6a–e,g…………………………….p. S84

Chiral HPLC Chromatograms for Compounds 3f, 6f…………………………..p. S95

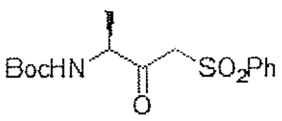

3a

$CDCl_3$, 500MHz

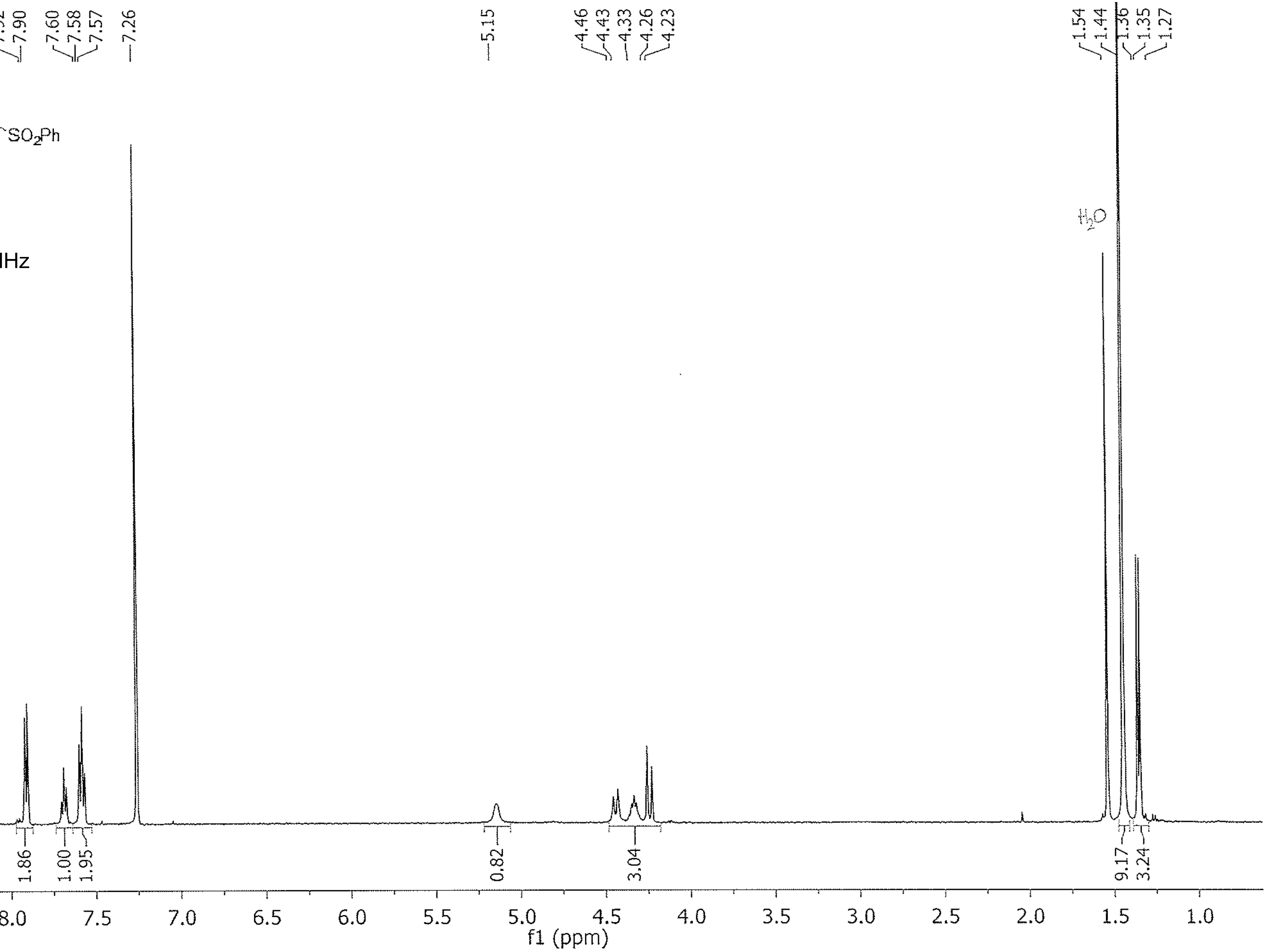

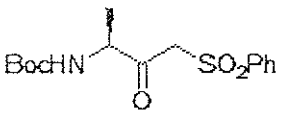

3a

CDCl$_3$, 125.68 MHz

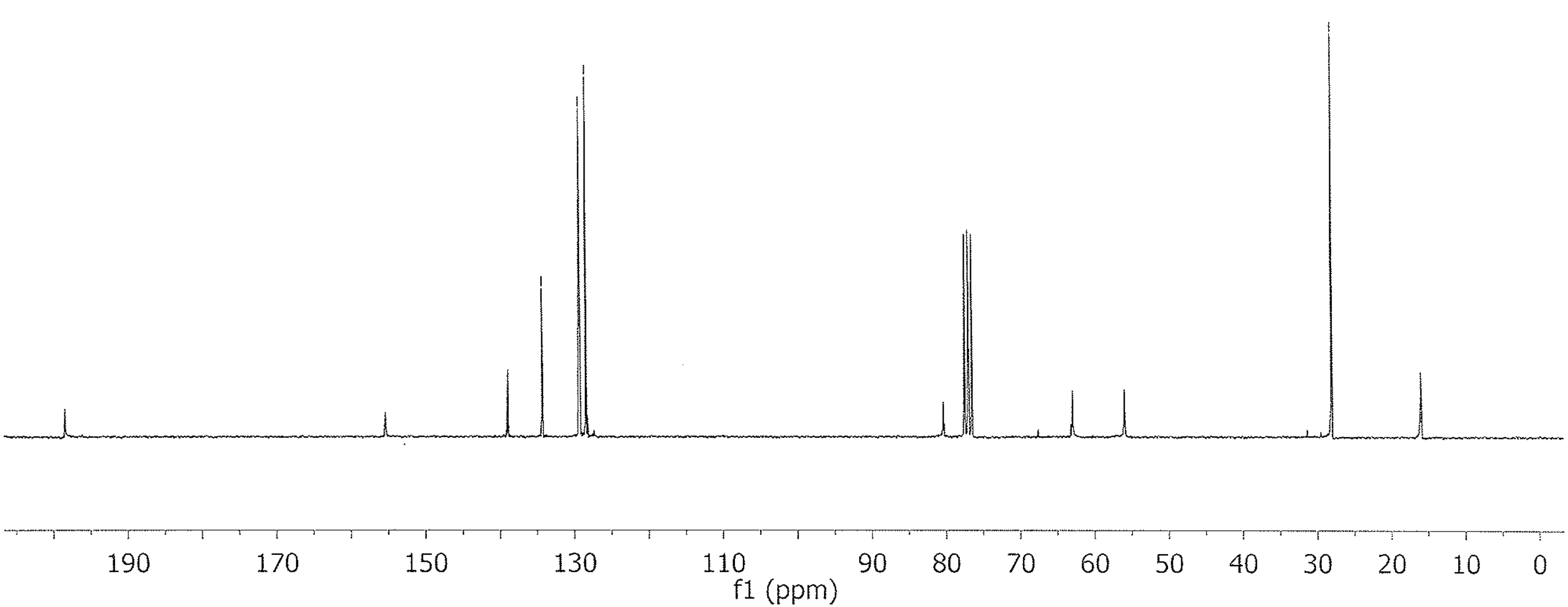

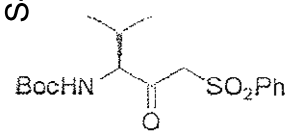

3b

$CDCl_3$, 500MHz

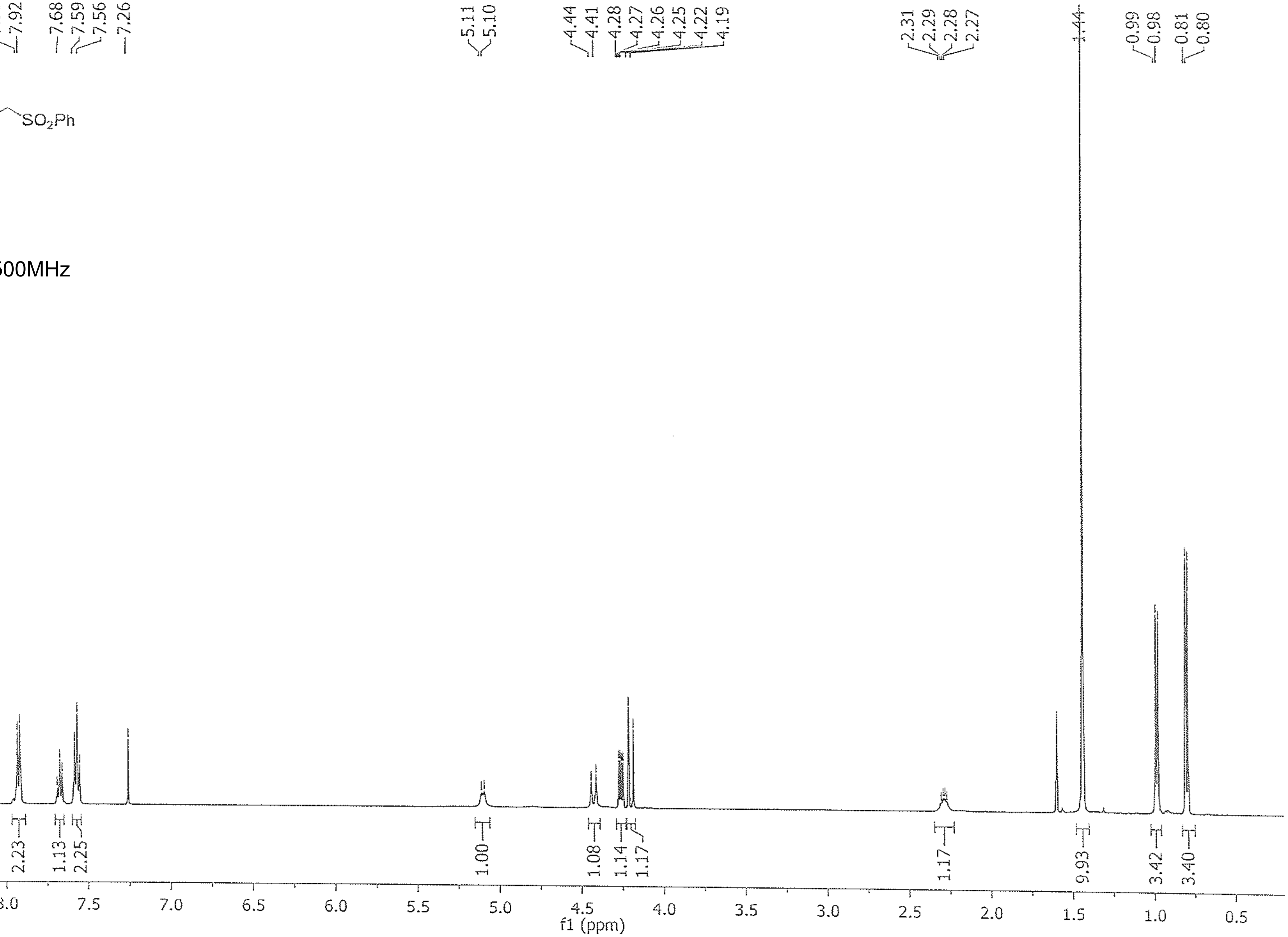

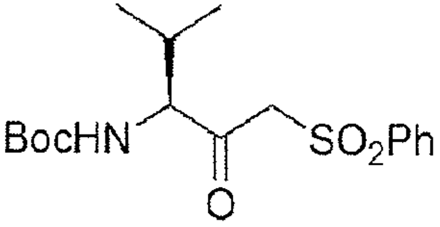

3b

$CDCl_3$, 125.68 MHz

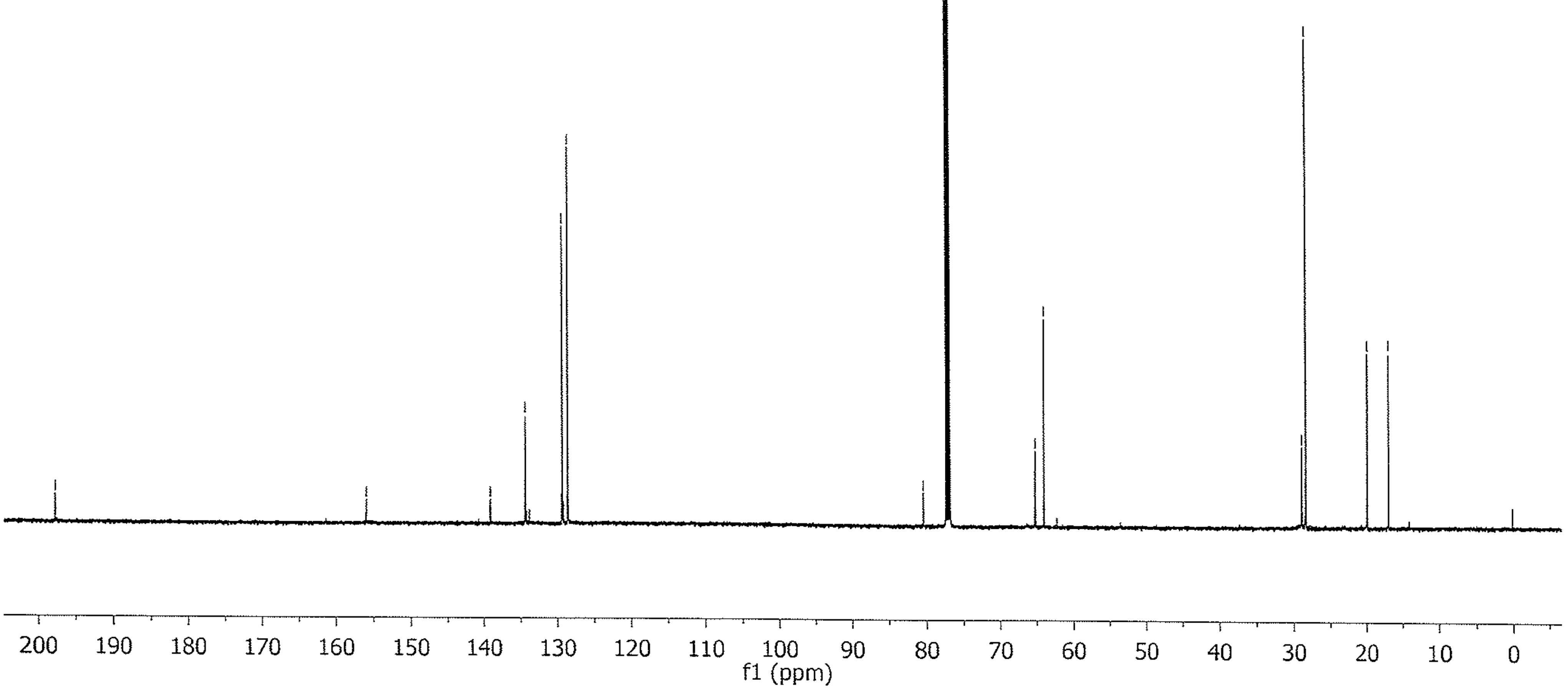

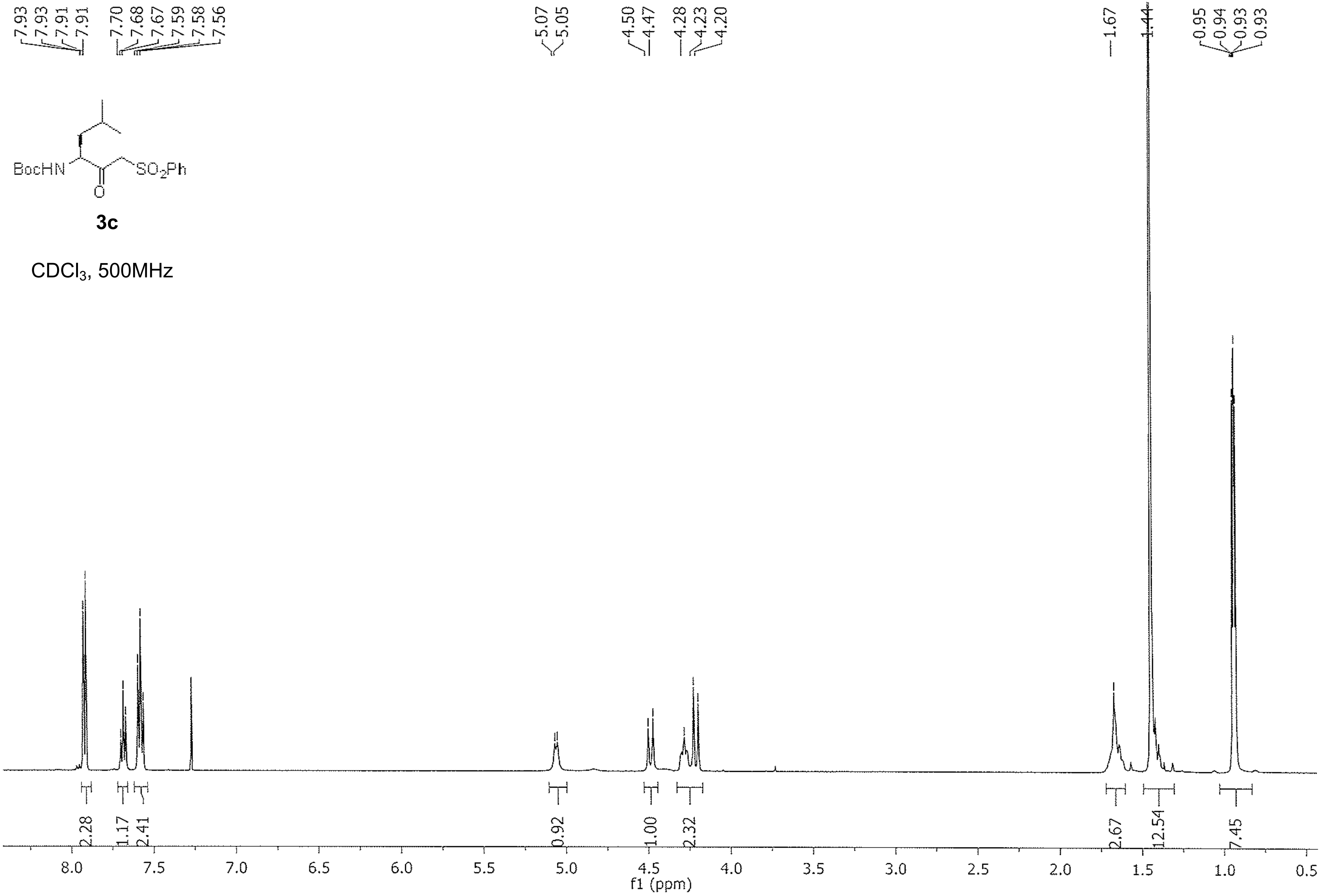

3c

$CDCl_3$, 500MHz

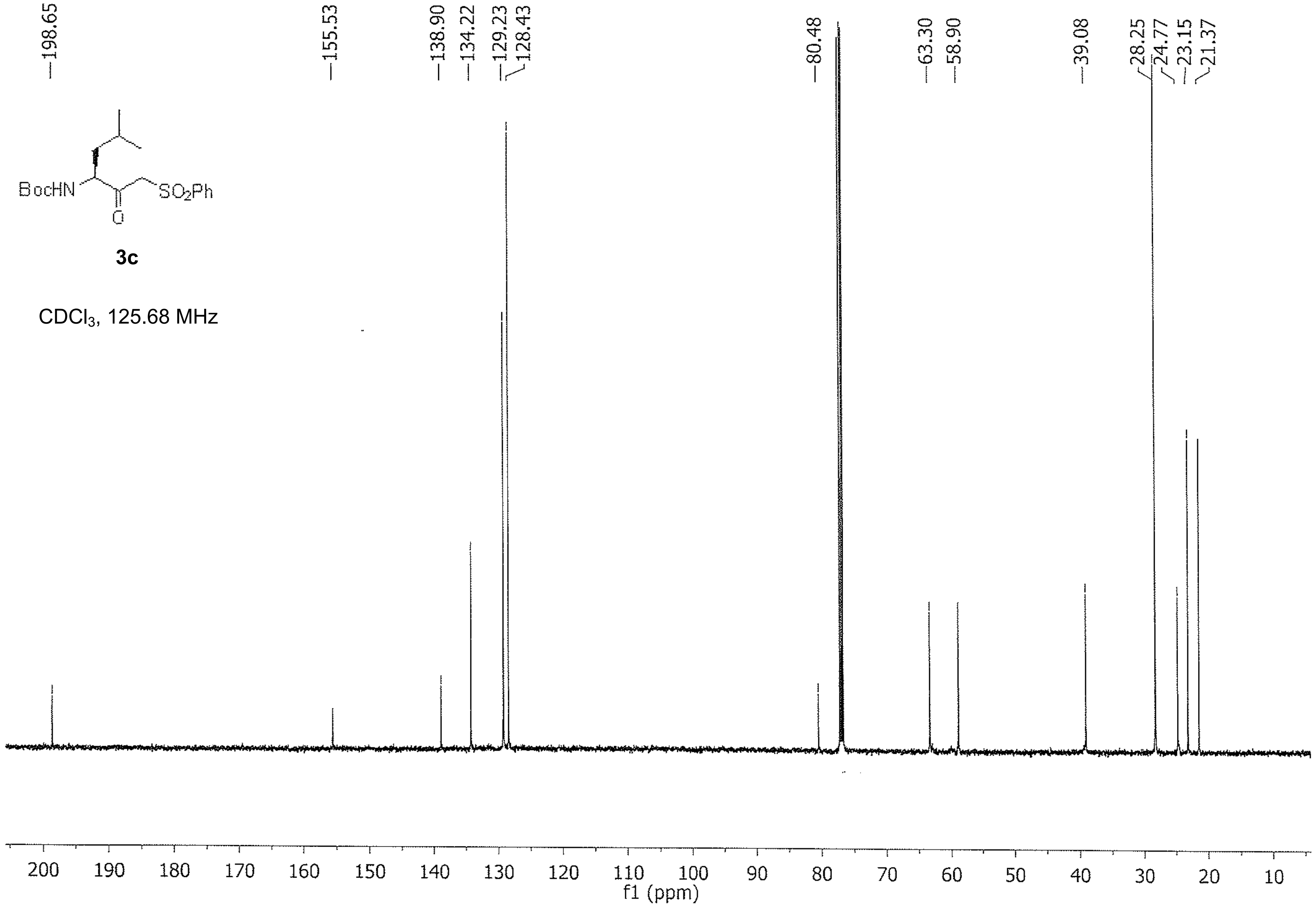

3c

$CDCl_3$, 125.68 MHz

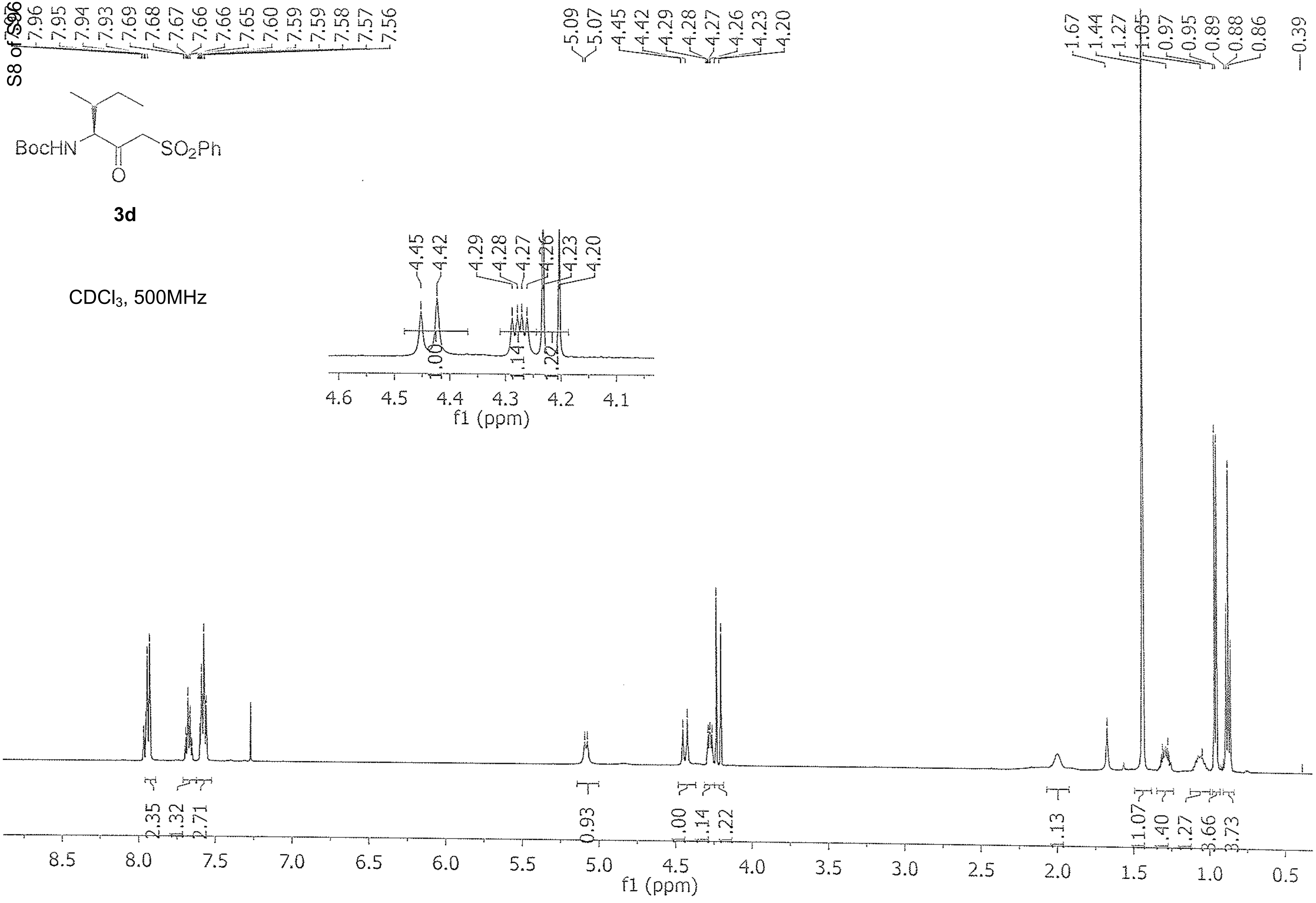
7.97 7.96 7.95 7.94 7.93 7.69 7.68 7.67 7.66 7.66 7.65 7.60 7.59 7.59 7.58 7.57 7.56
5.09 5.07 4.45 4.42 4.29 4.28 4.27 4.26 4.23 4.20
1.67 1.44 1.27 1.05 0.97 0.95 0.89 0.88 0.86 0.39
BocHN
SO_2Ph
O
3d
$CDCl_3$, 500MHz
4.45 4.42 4.29 4.28 4.27 4.26 4.23 4.20
1.00 1.14 1.22
4.6 4.5 4.4 4.3 4.2 4.1
f1 (ppm)
2.35 1.32 2.71 0.93 1.00 1.14 1.22 1.13 1.07 1.40 1.27 3.66 3.73
8.5 8.0 7.5 7.0 6.5 6.0 5.5 5.0 4.5 4.0 3.5 3.0 2.5 2.0 1.5 1.0 0.5
f1 (ppm)

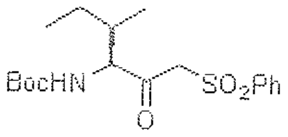

3d

$CDCl_3$, 125.68 MHz

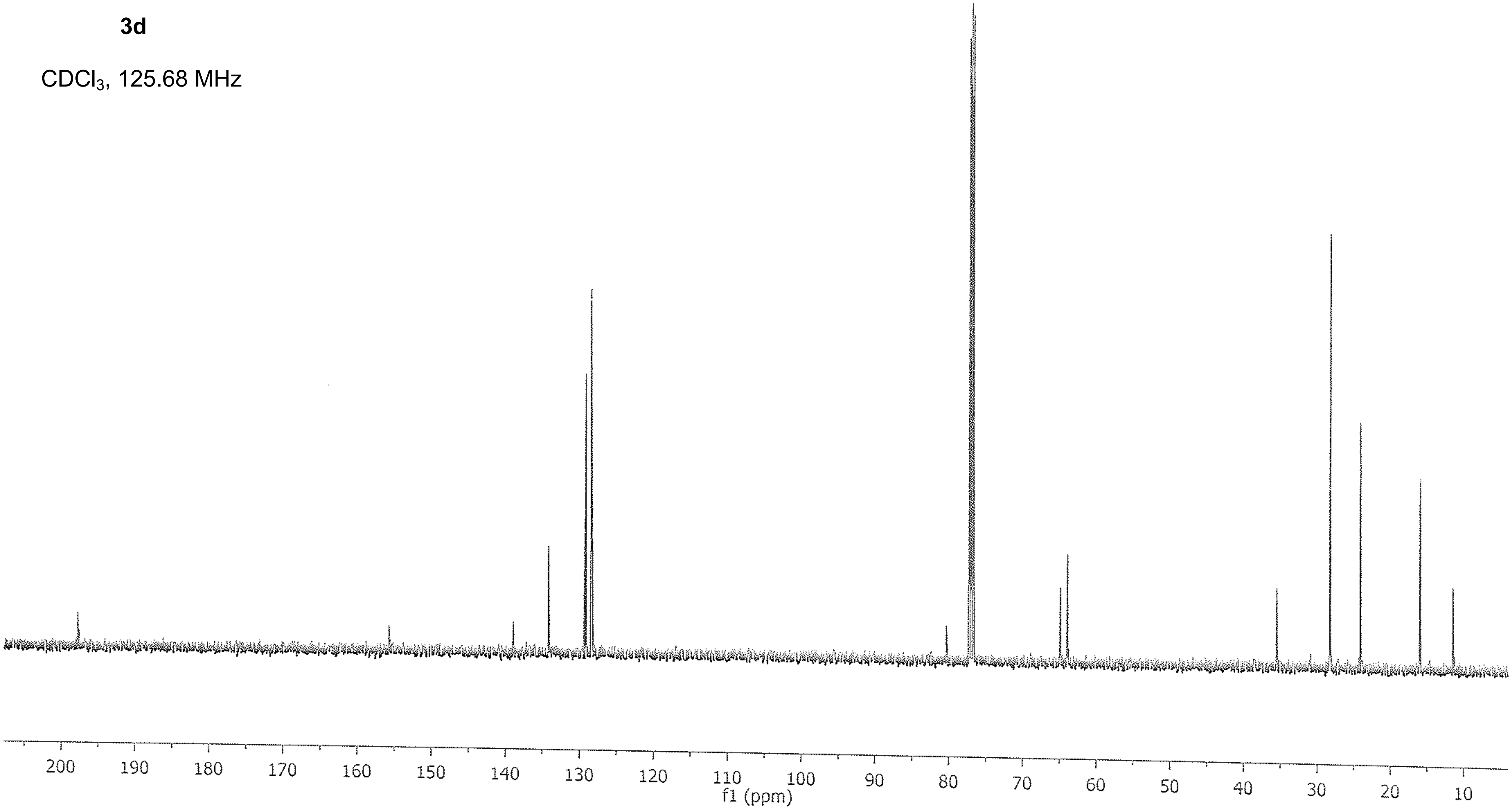

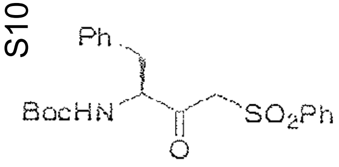

3e

$CDCl_3$, 500MHz

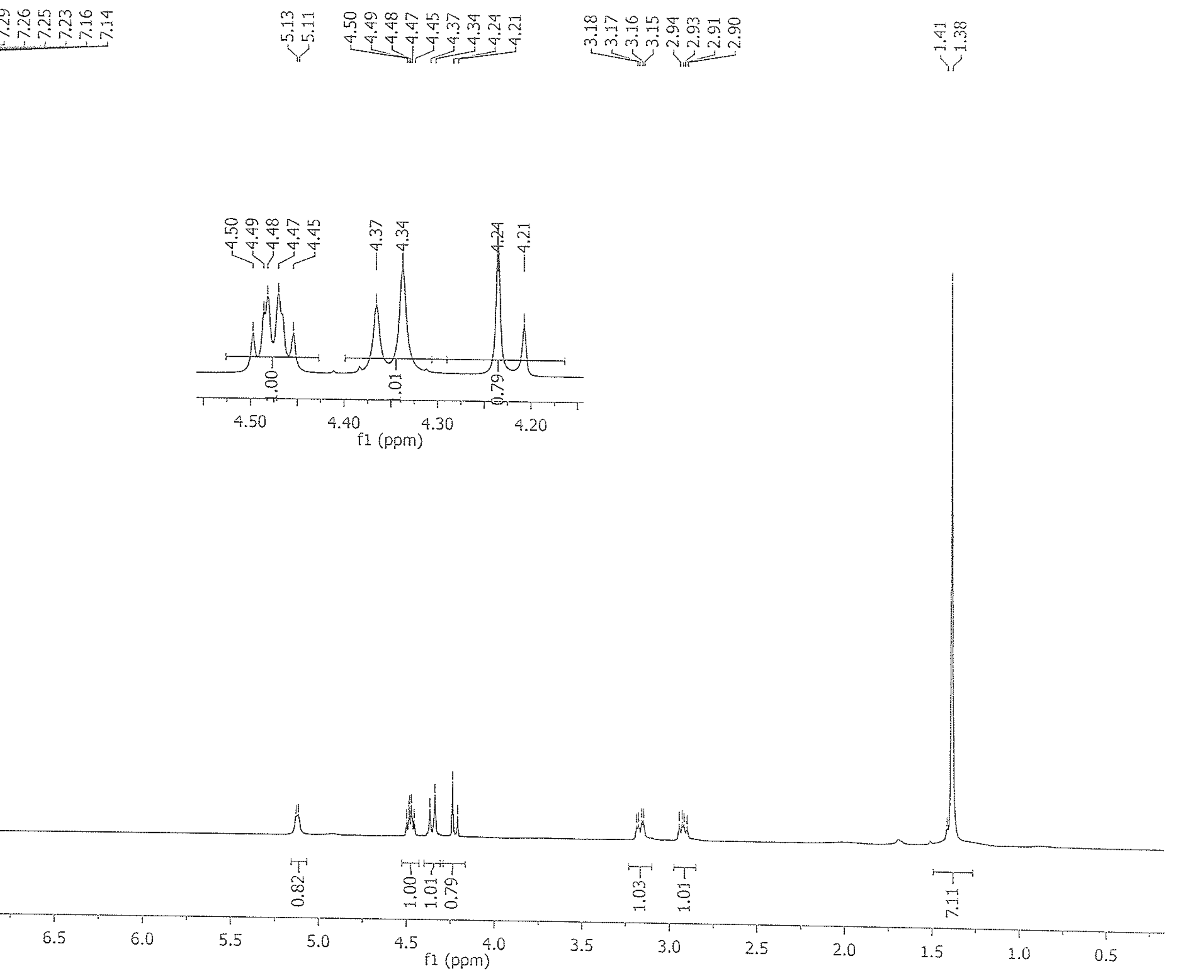

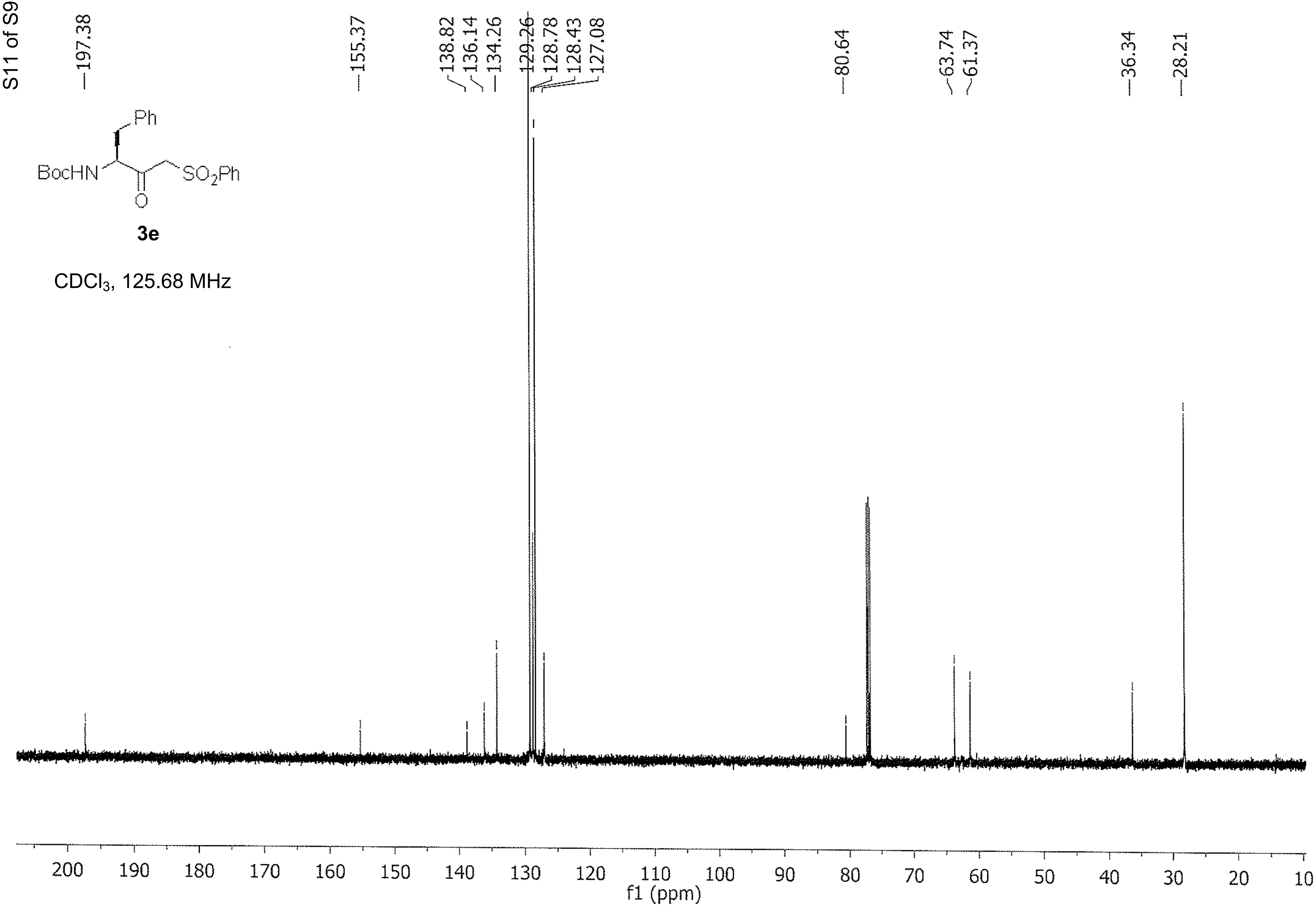

3e

$CDCl_3$, 125.68 MHz

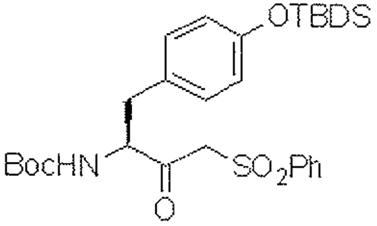

3f

$CDCl_3$, 500MHz

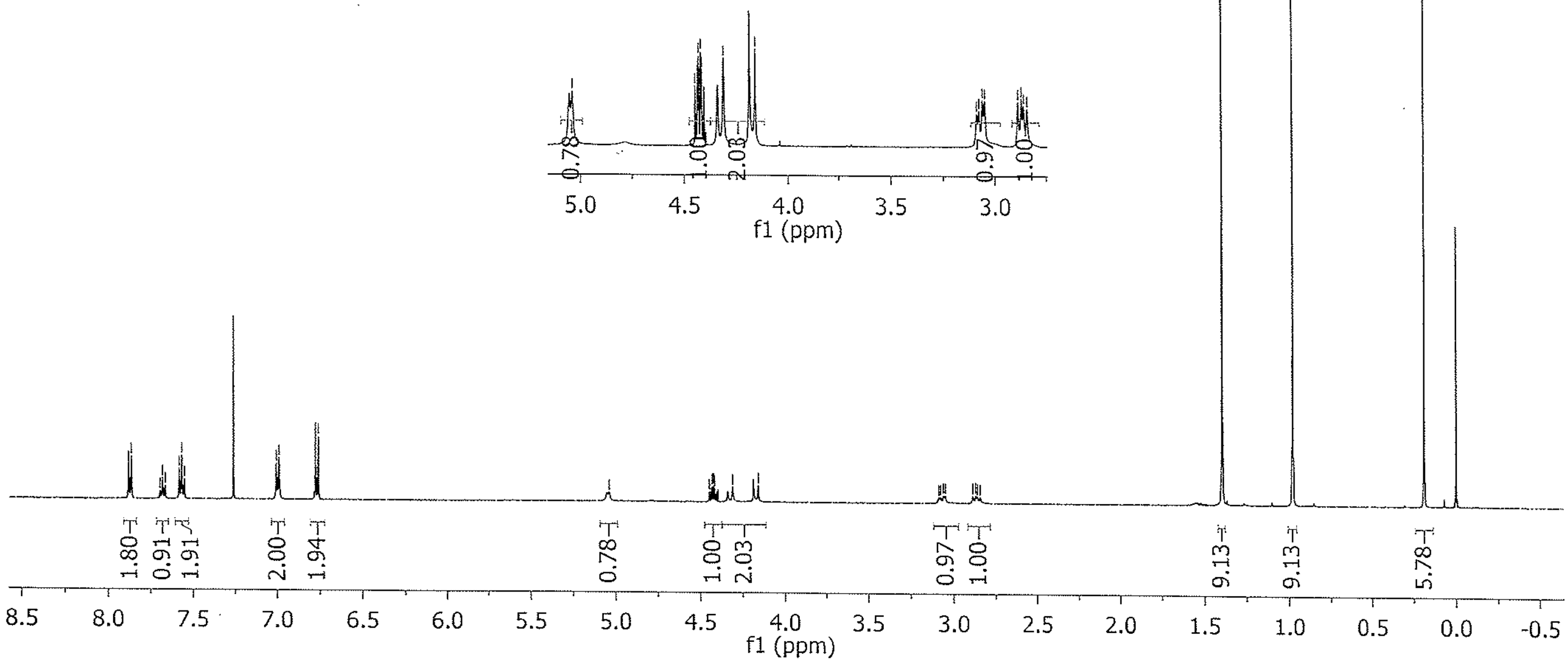

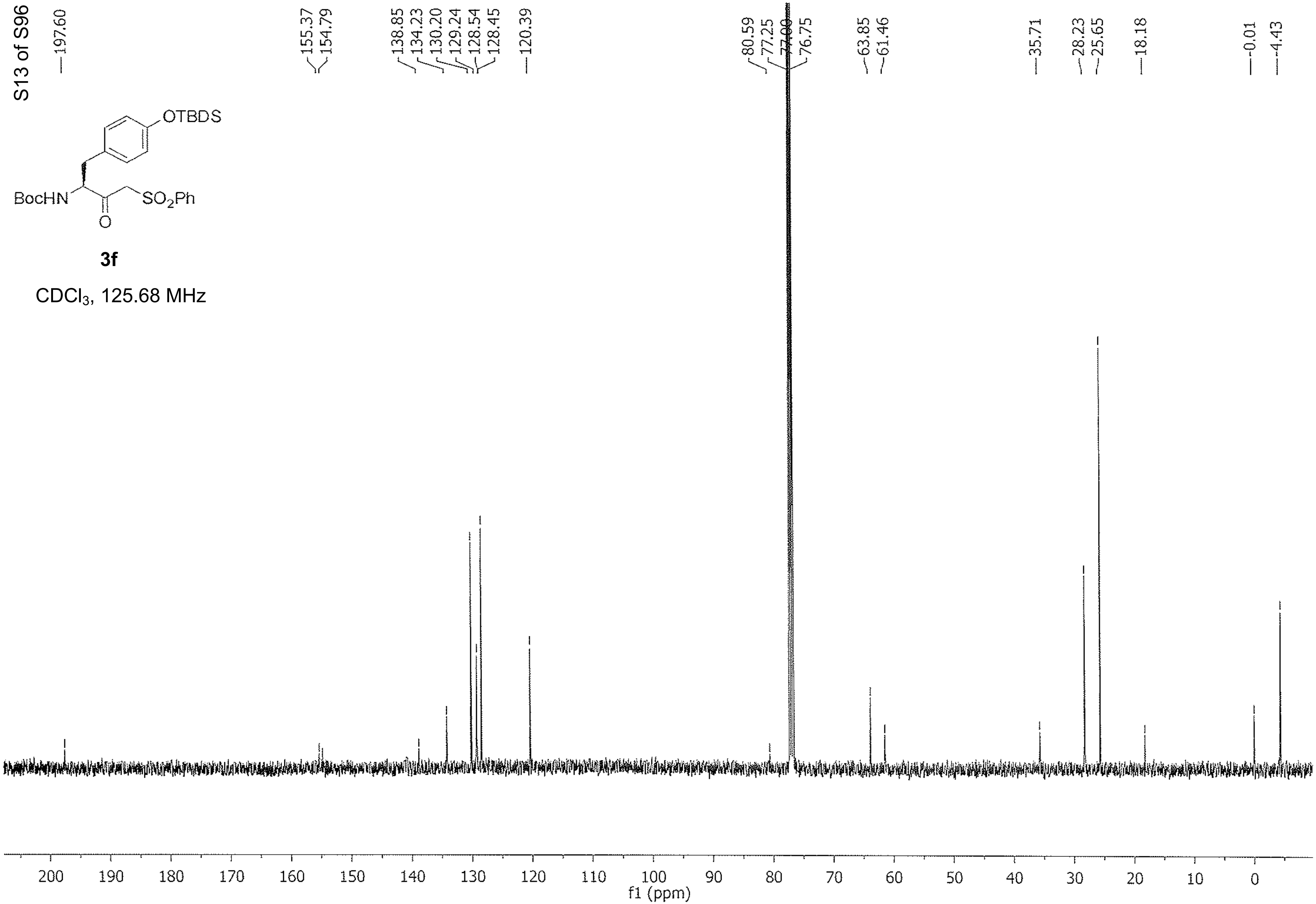

3f

$CDCl_3$, 125.68 MHz

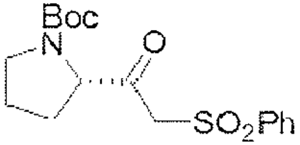

3g

CDCl$_3$, 500MHz

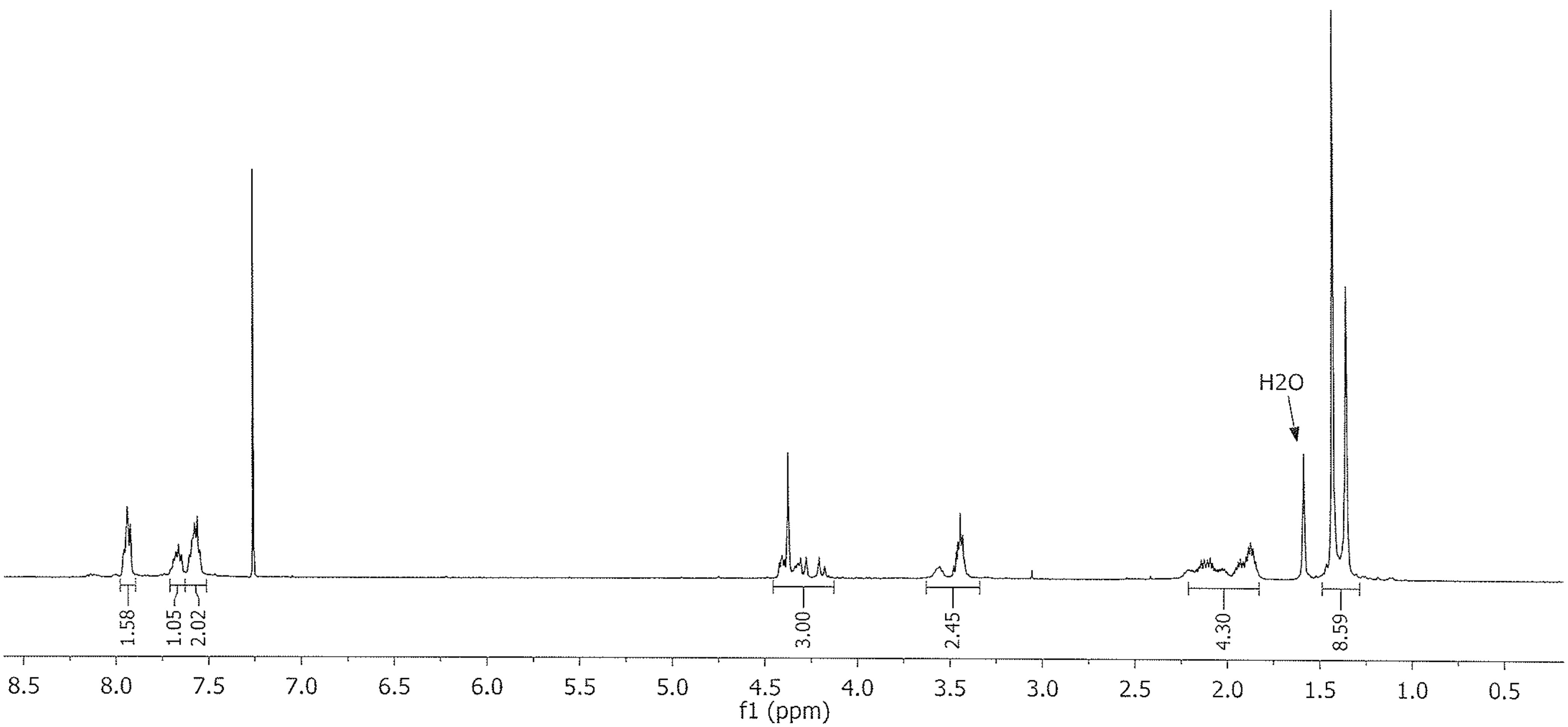

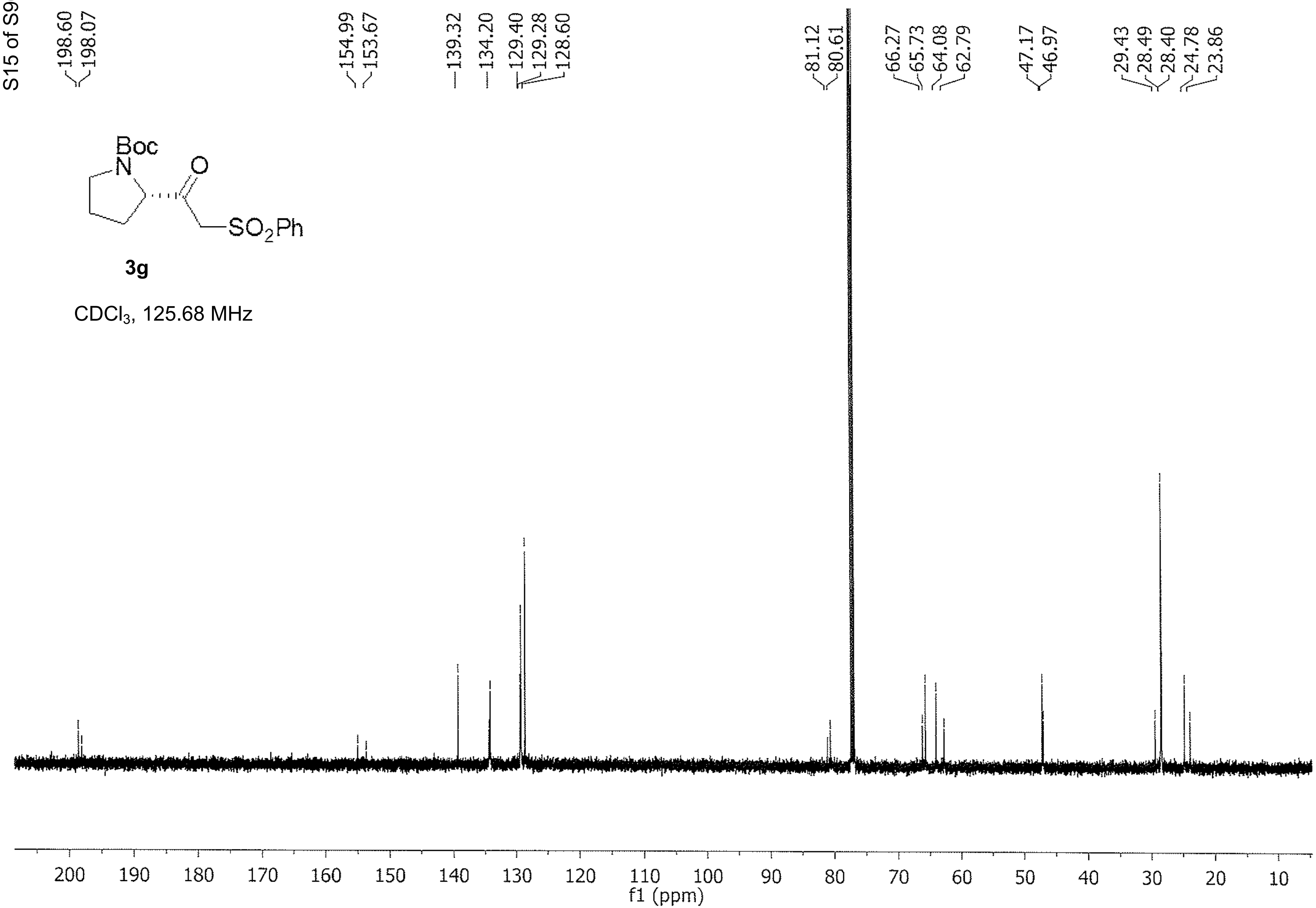
Boc
N
O
SO2Ph
3g
CDCl3, 125.68 MHz
198.60
198.07
154.99
153.67
139.32
134.20
129.40
129.28
128.60
81.12
80.61
66.27
65.73
64.08
62.79
47.17
46.97
29.43
28.49
28.40
24.78
23.86
200 190 180 170 160 150 140 130 120 110 100 90 80 70 60 50 40 30 20 10
f1 (ppm)

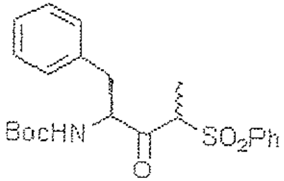

3h

$CDCl_3$, 500MHz

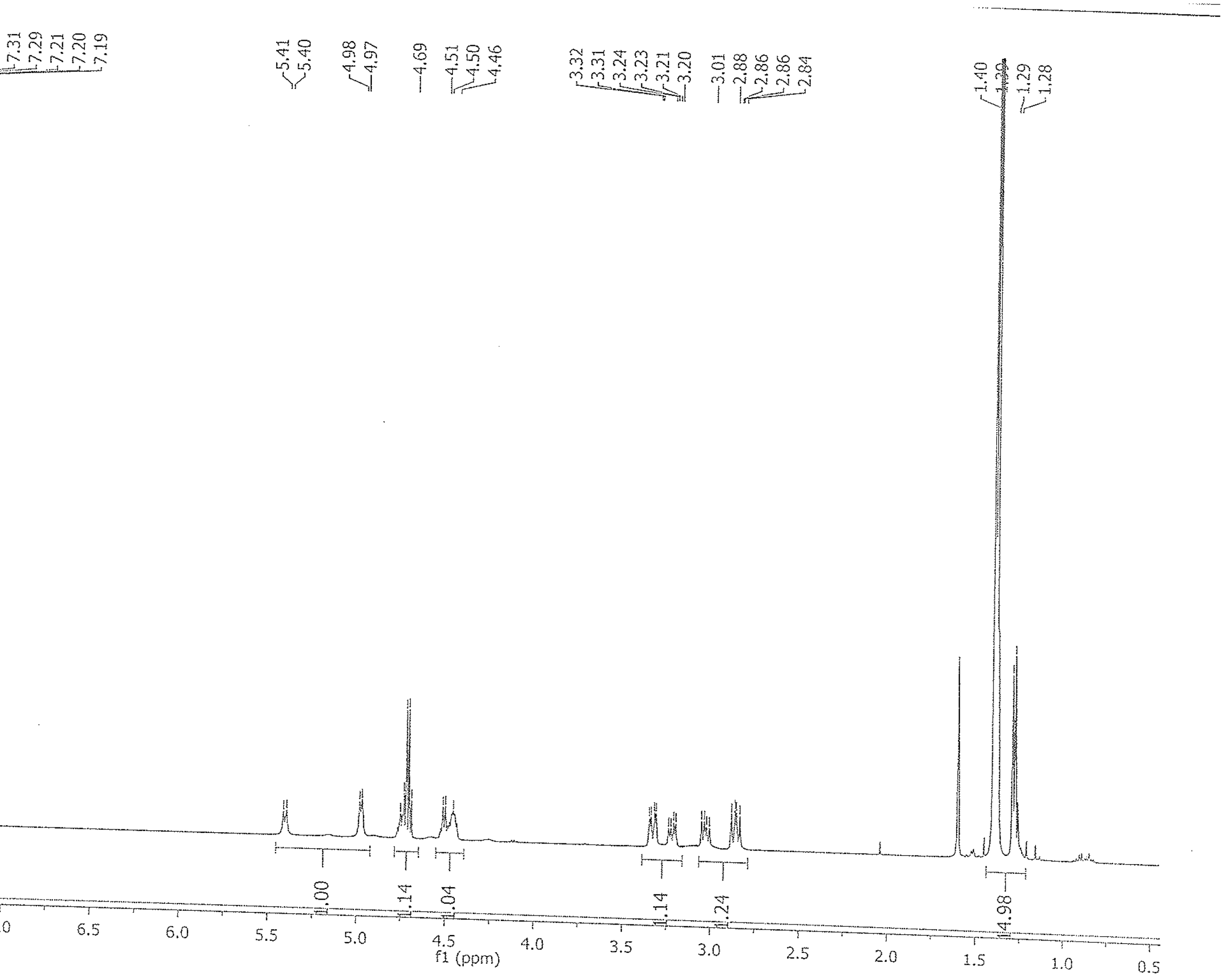

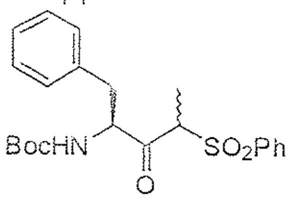

3h

$CDCl_3$, 125.68 MHz

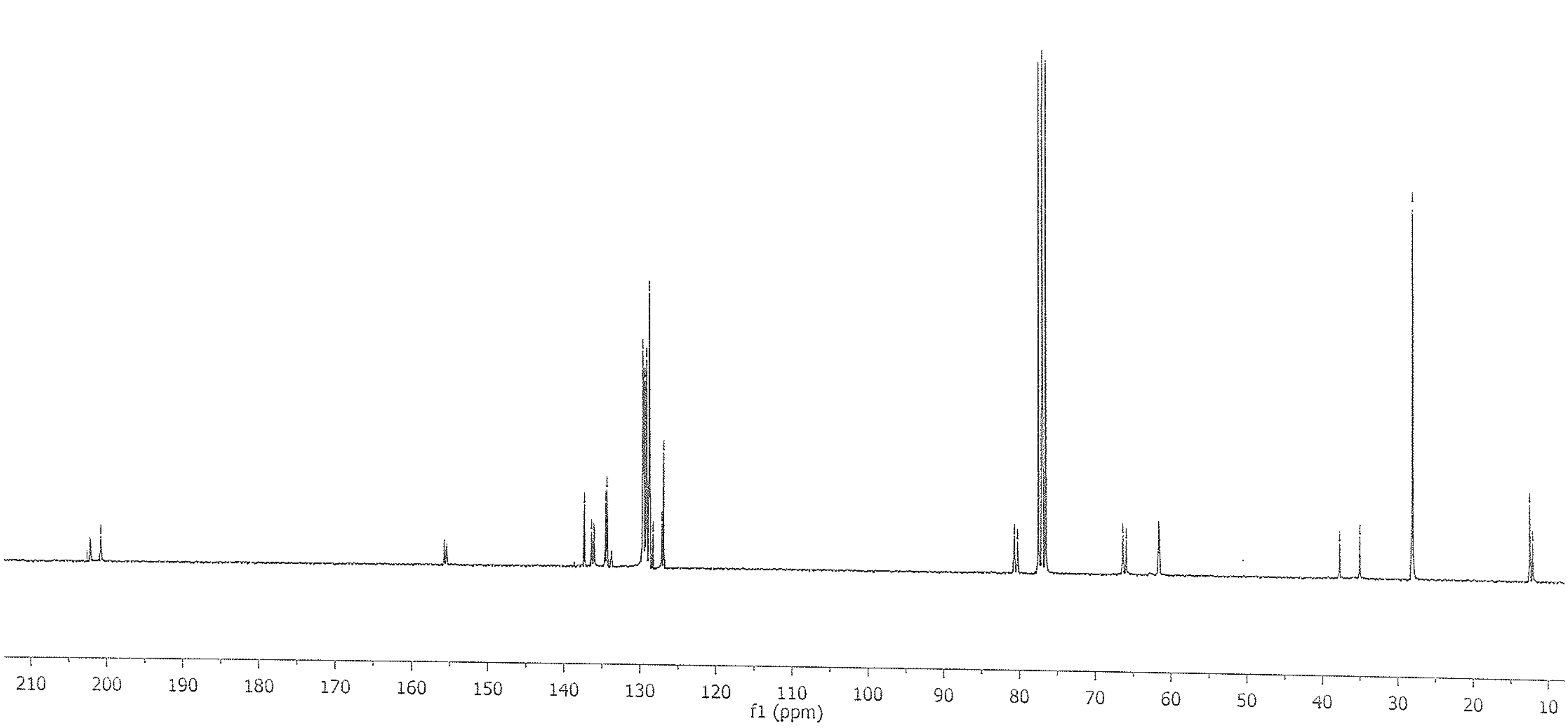

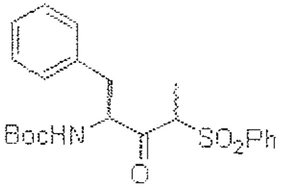

3h

$CDCl_3$

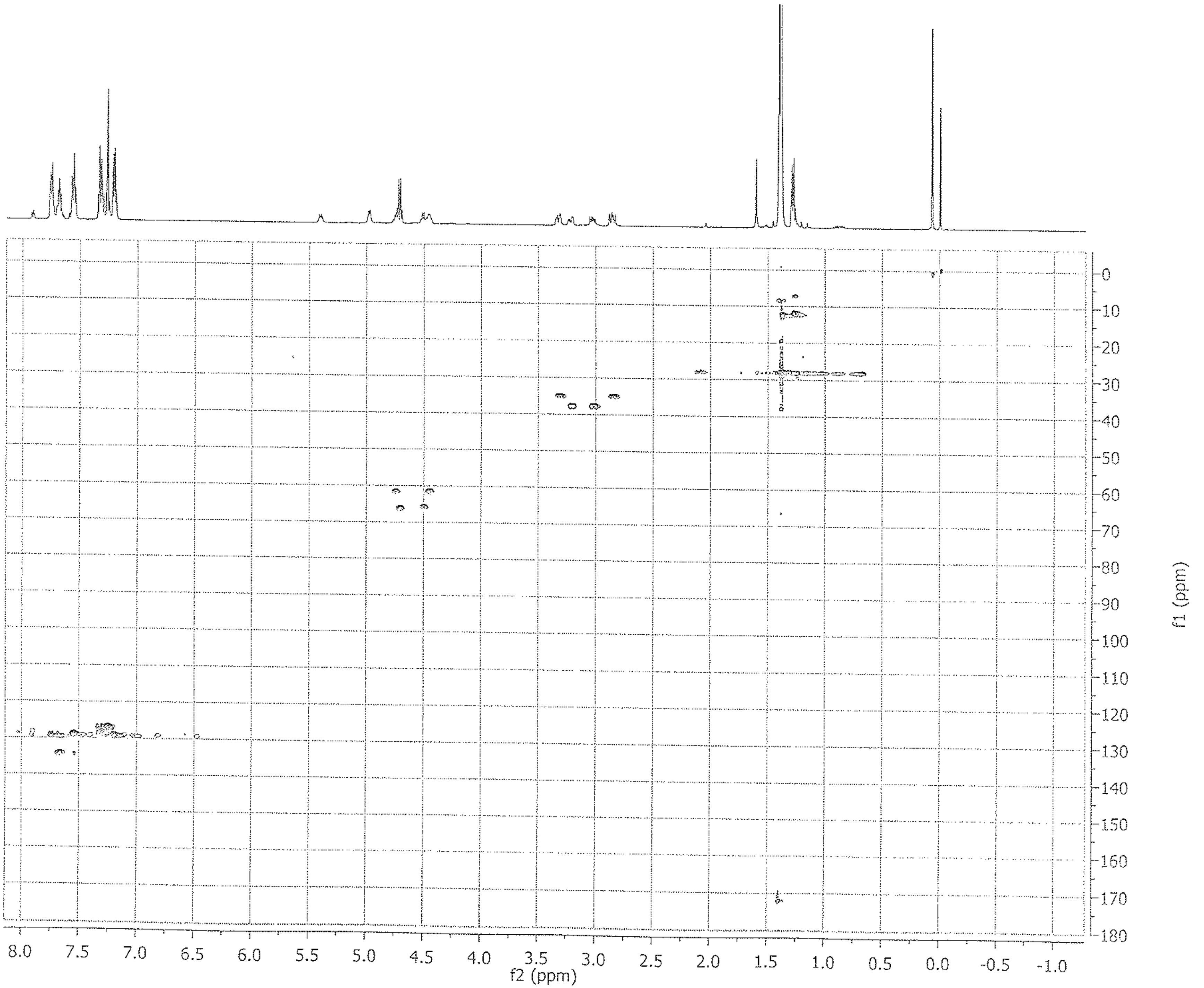

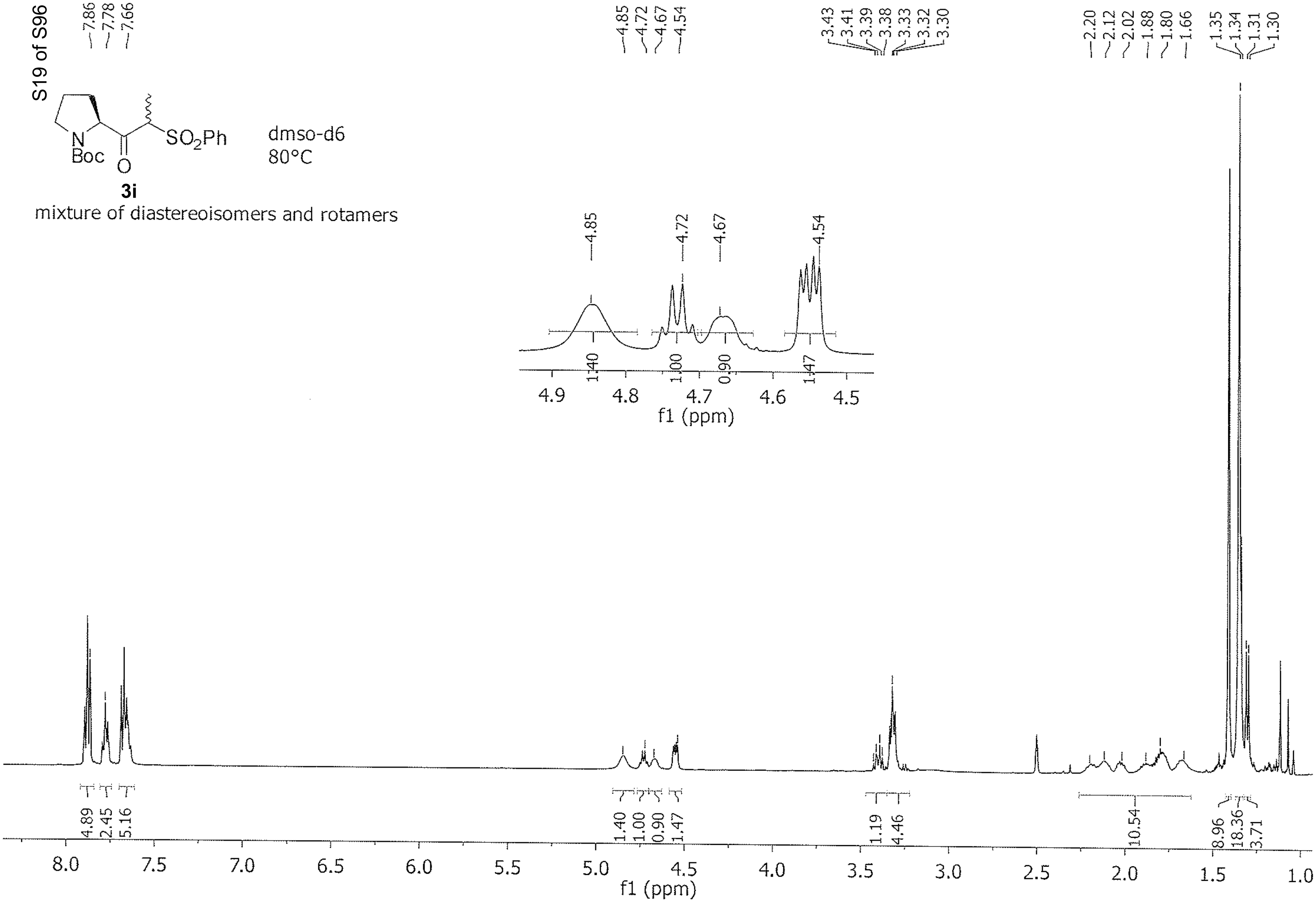
3i
dmso-d6
80°C
mixture of diastereoisomers and rotamers
7.86
7.78
7.66
4.85
4.72
4.67
4.54
3.43
3.41
3.39
3.38
3.33
3.32
3.30
2.20
2.12
2.02
1.88
1.80
1.66
1.35
1.34
1.31
1.30
1.40
1.00
0.90
1.47
4.9
4.8
4.7
4.6
4.5
f1 (ppm)
4.89
2.45
5.16
1.40
1.00
0.90
1.47
1.19
4.46
10.54
8.96
18.36
3.71
8.0
7.5
7.0
6.5
6.0
5.5
5.0
4.5
4.0
3.5
3.0
2.5
2.0
1.5
1.0
f1 (ppm)

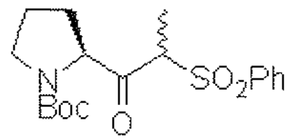

3i

dmso-d6
80°C

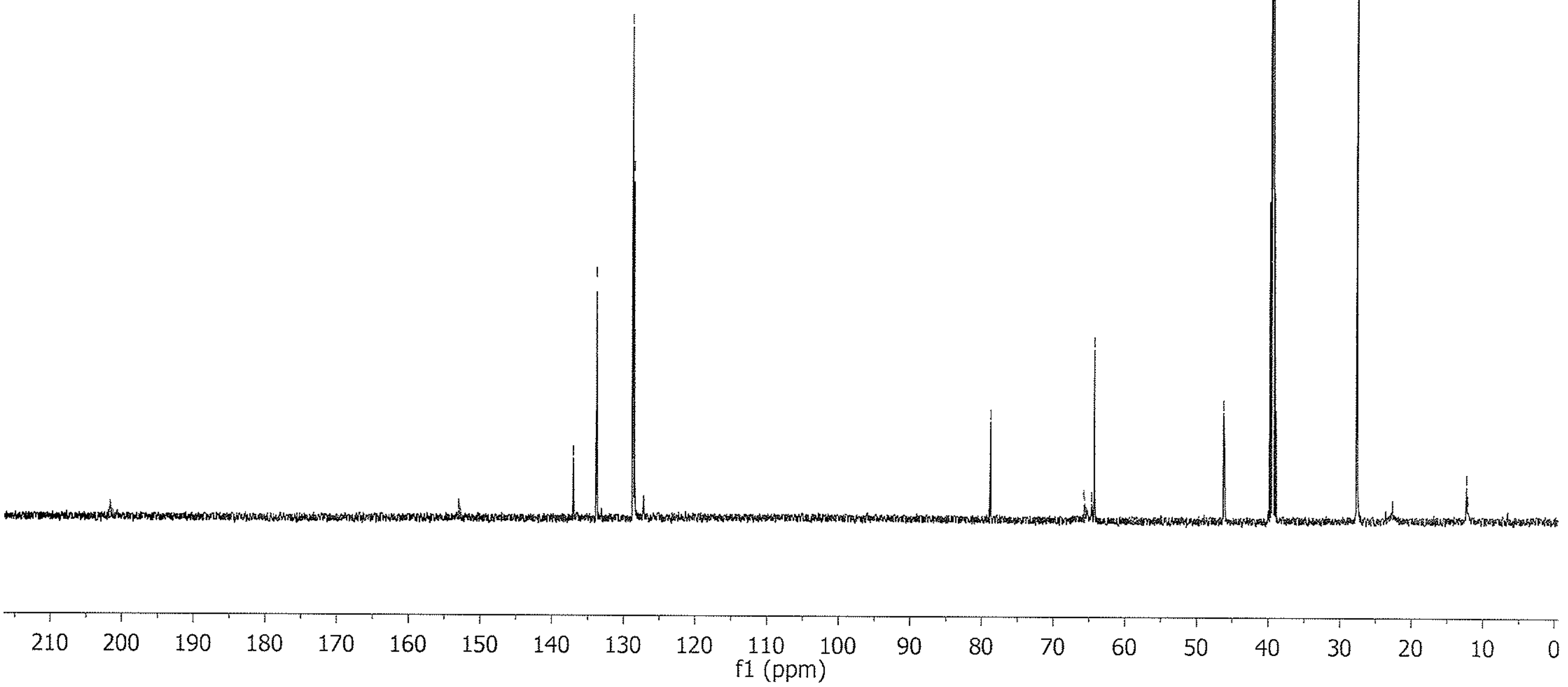

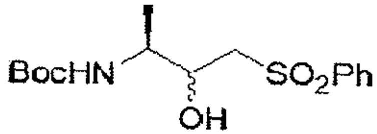

4a

$CDCl_3$, 500MHz

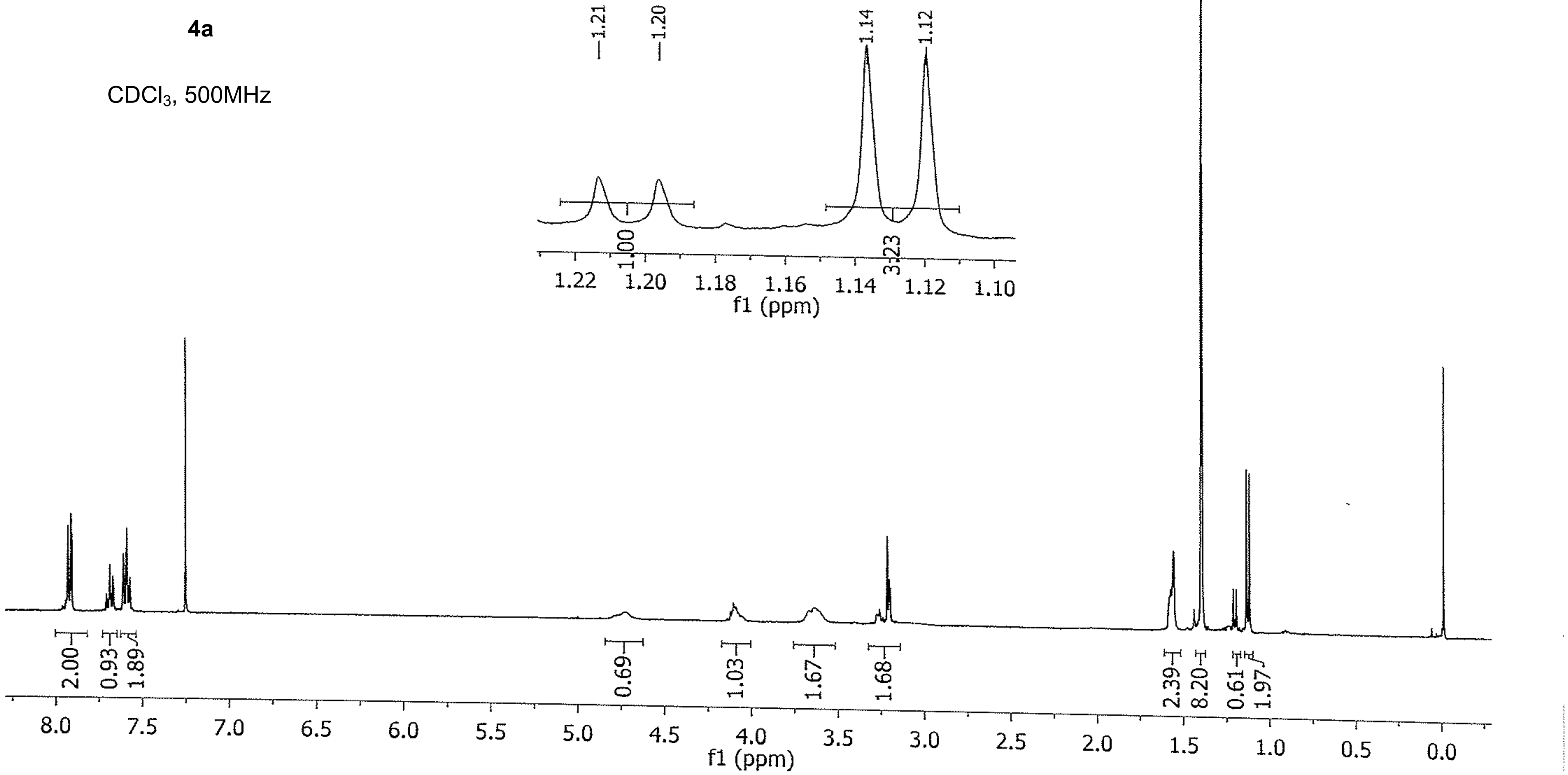

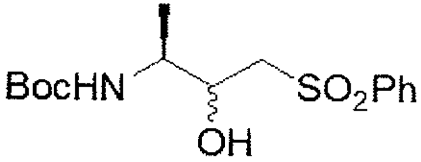

4a

$CDCl_3$, 125.68 MHz

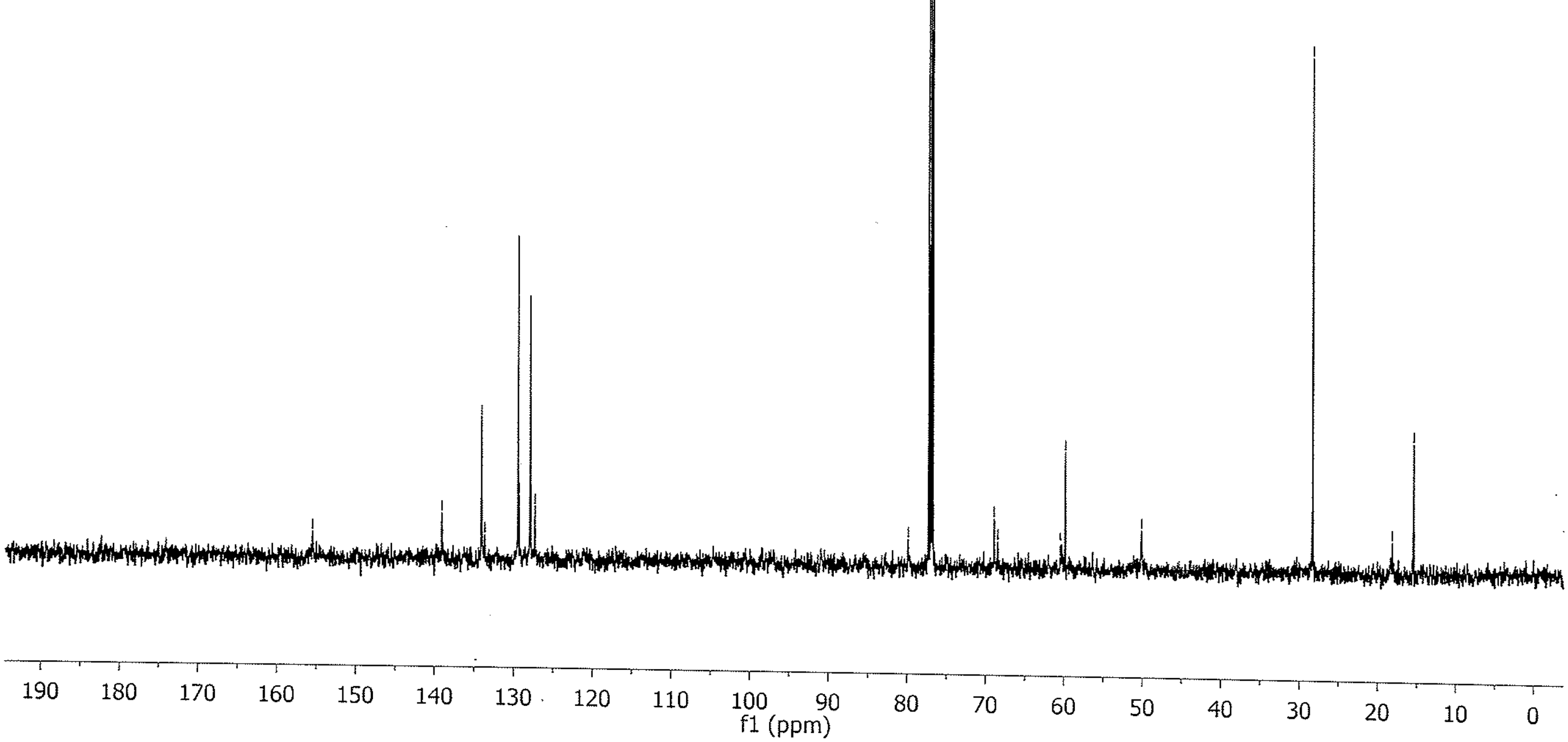

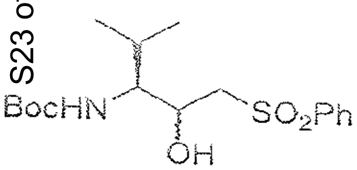

4b

$CDCl_3$, 500MHz

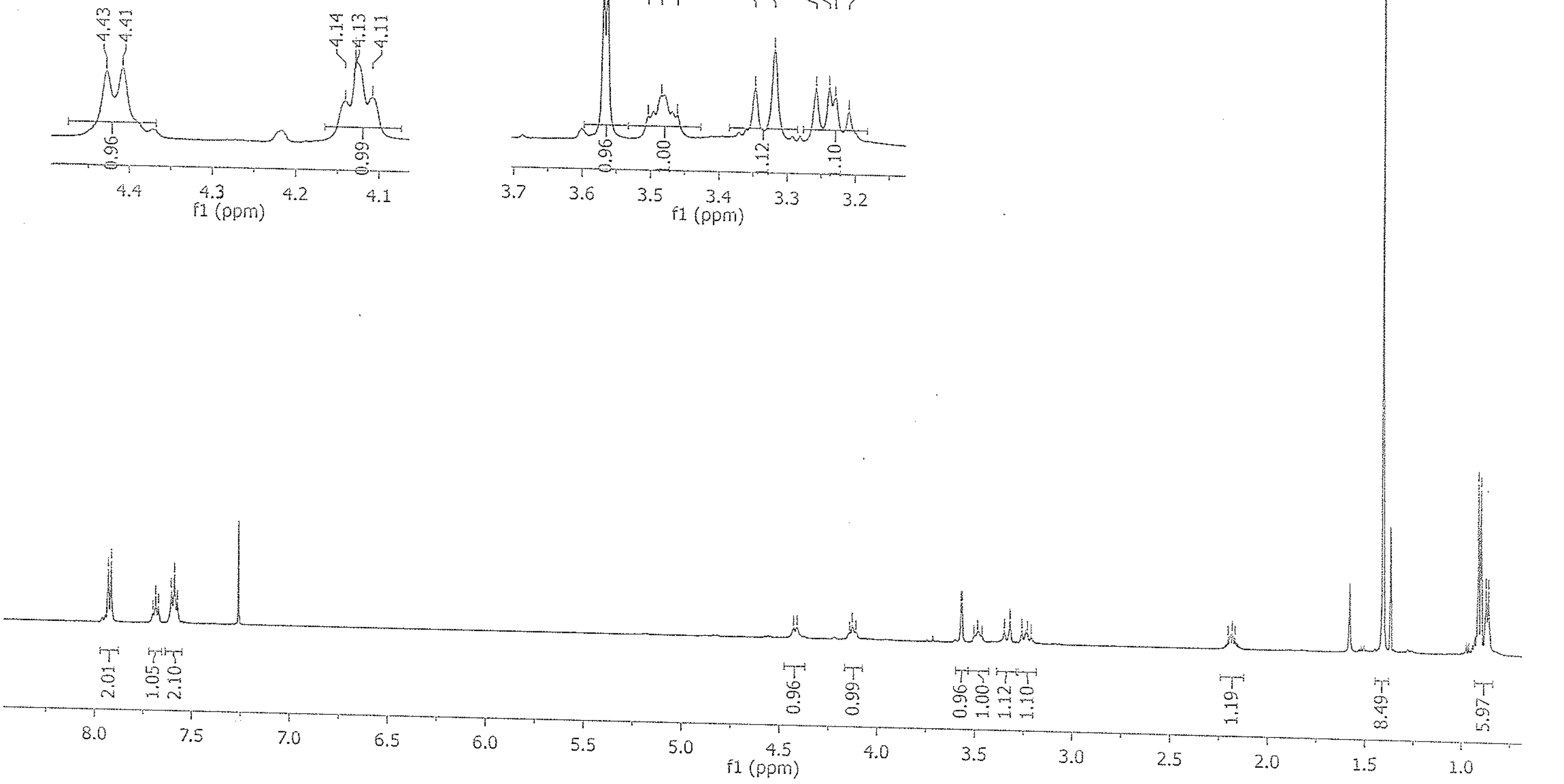

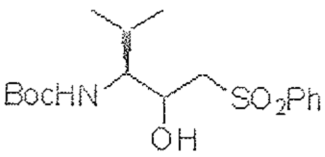

4b

$CDCl_3$, 125.68 MHz

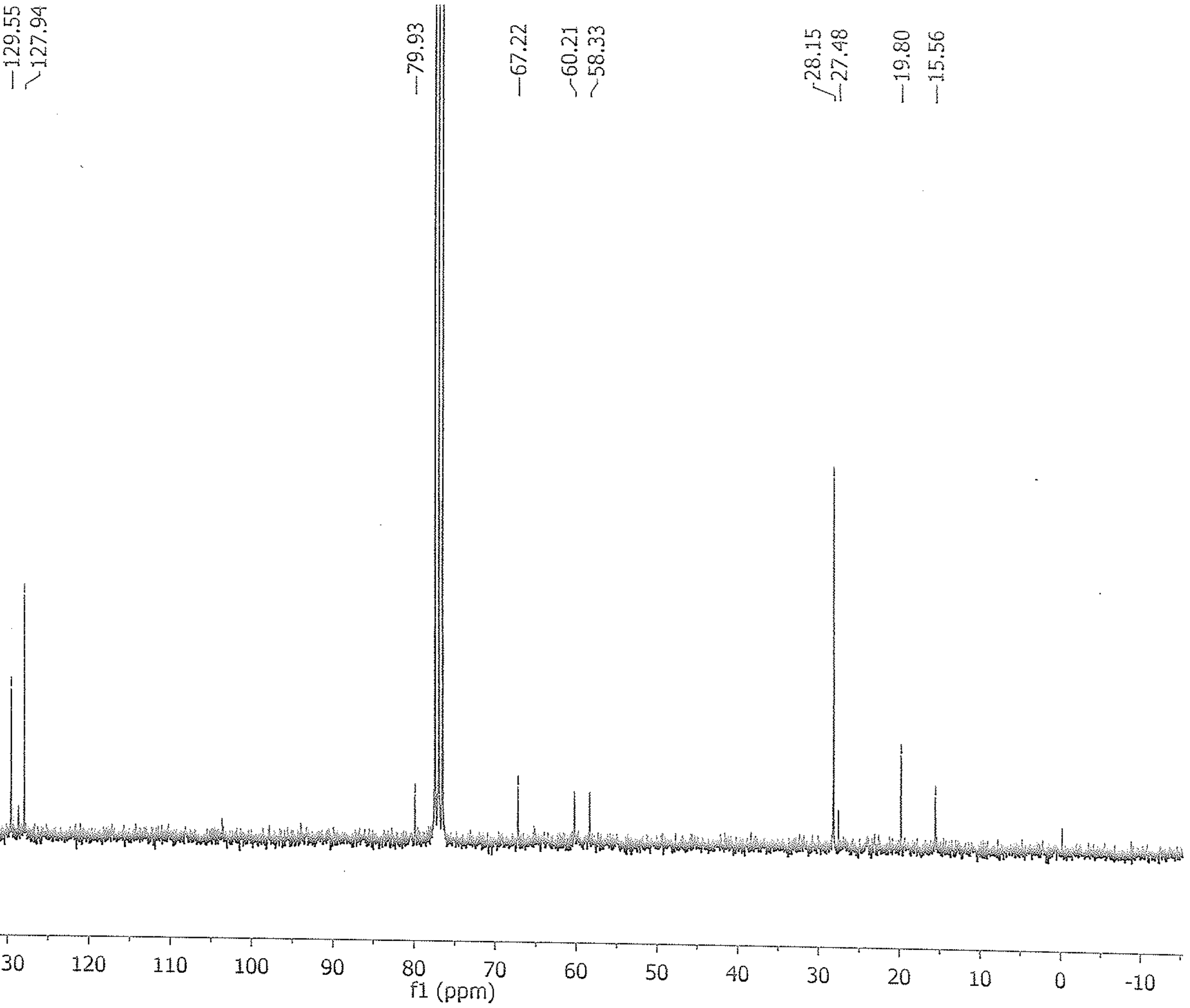

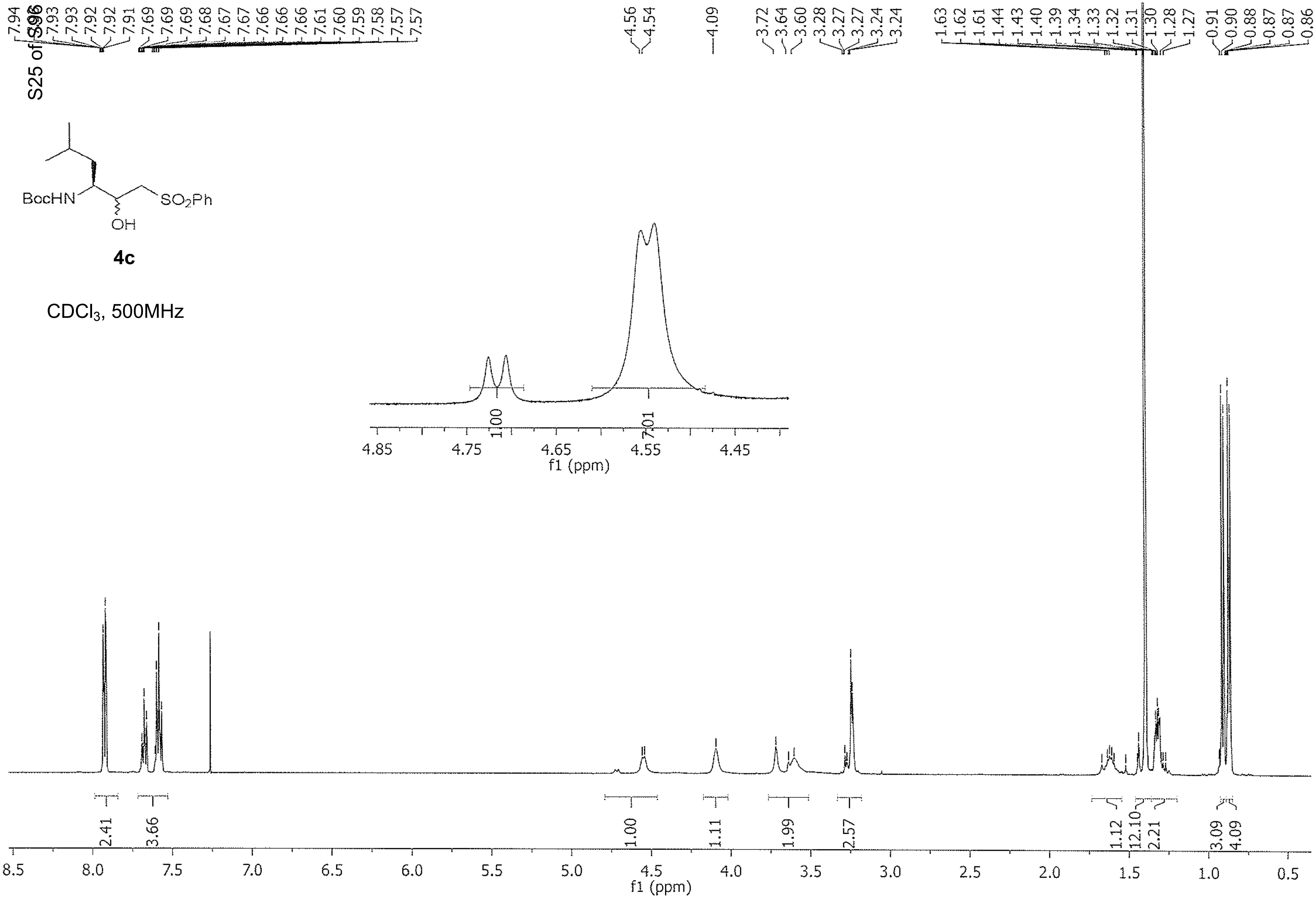
7.94 7.93 7.93 7.92 7.92 7.91 7.69 7.69 7.69 7.68 7.67 7.67 7.66 7.66 7.66 7.61 7.60 7.59 7.58 7.57 7.57
4.56 4.54
4.09
3.72 3.64 3.60 3.28 3.27 3.27 3.24 3.24
1.63 1.62 1.61 1.44 1.43 1.40 1.39 1.34 1.33 1.32 1.31 1.30 1.28 1.27 0.91 0.90 0.88 0.87 0.87 0.86
BocHN
SO2Ph
OH
4c
CDCl3, 500MHz
1.00
7.01
4.85 4.75 4.65 4.55 4.45
f1 (ppm)
2.41 3.66 1.00 1.11 1.99 2.57 1.12 12.10 2.21 3.09 4.09
8.5 8.0 7.5 7.0 6.5 6.0 5.5 5.0 4.5 4.0 3.5 3.0 2.5 2.0 1.5 1.0 0.5
f1 (ppm)

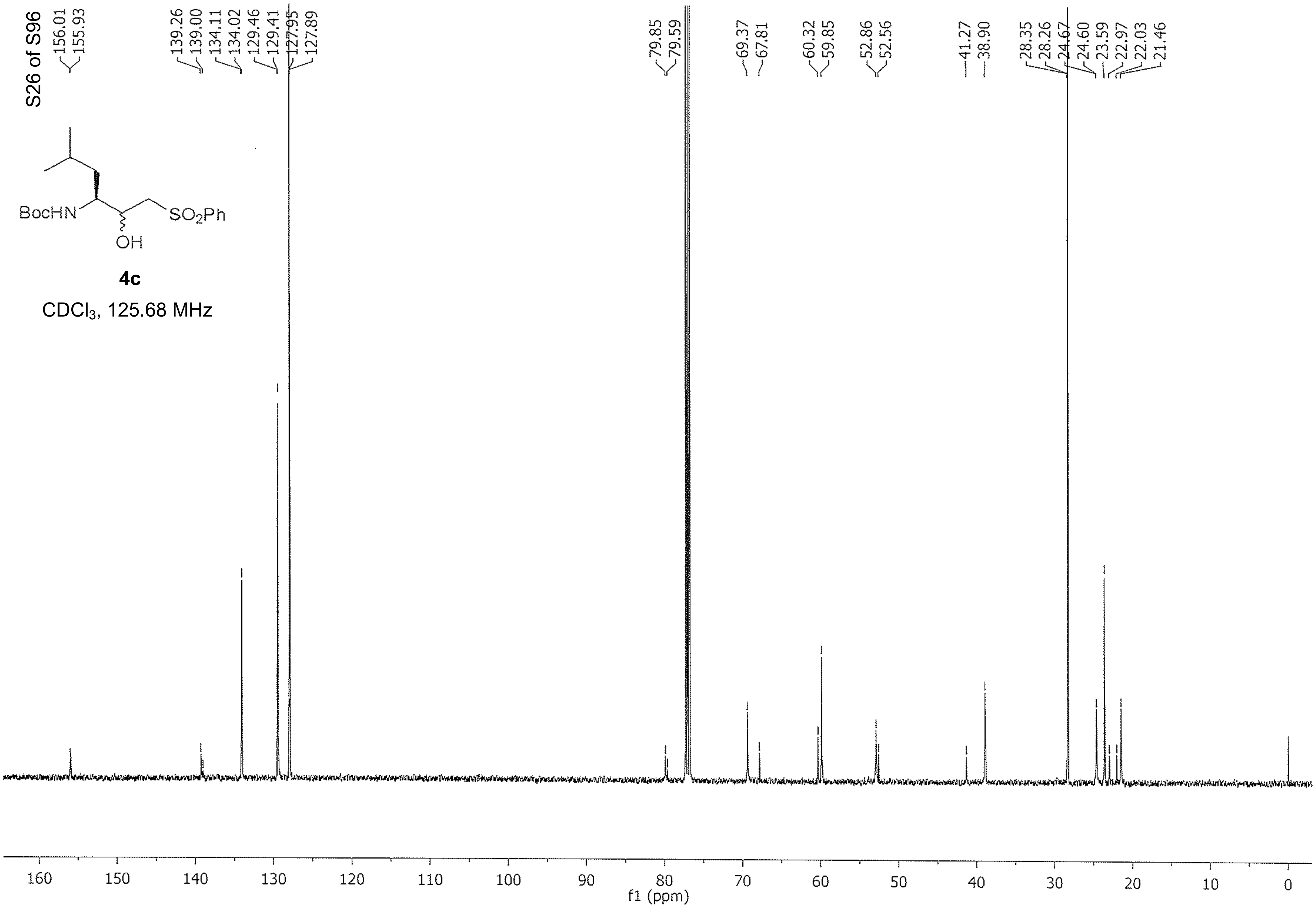

4c

$CDCl_3$, 125.68 MHz

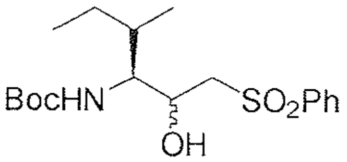

4d

$CDCl_3$, 500MHz

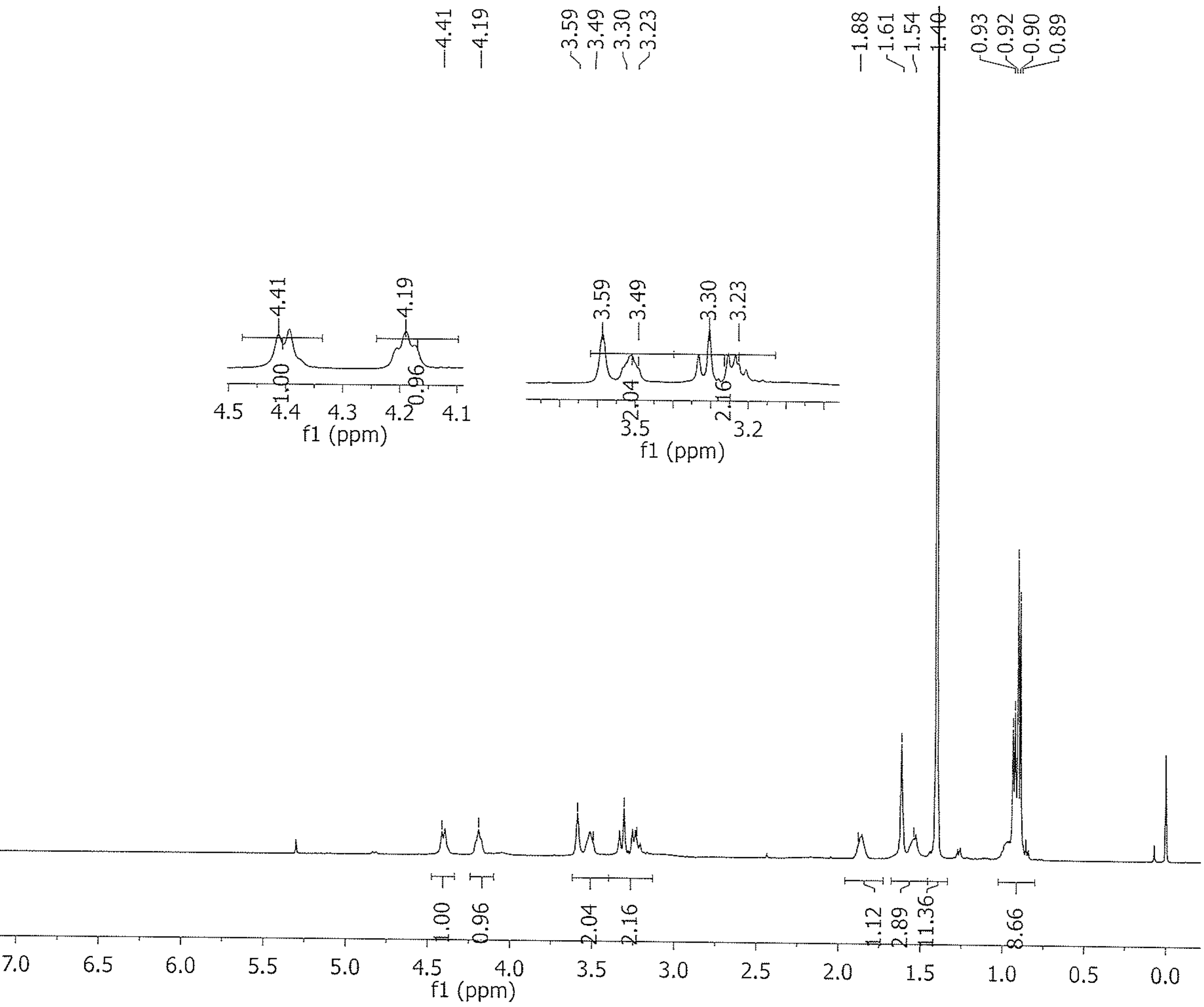

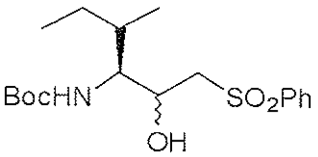

4d

CDCl$_3$, 125.68 MHz

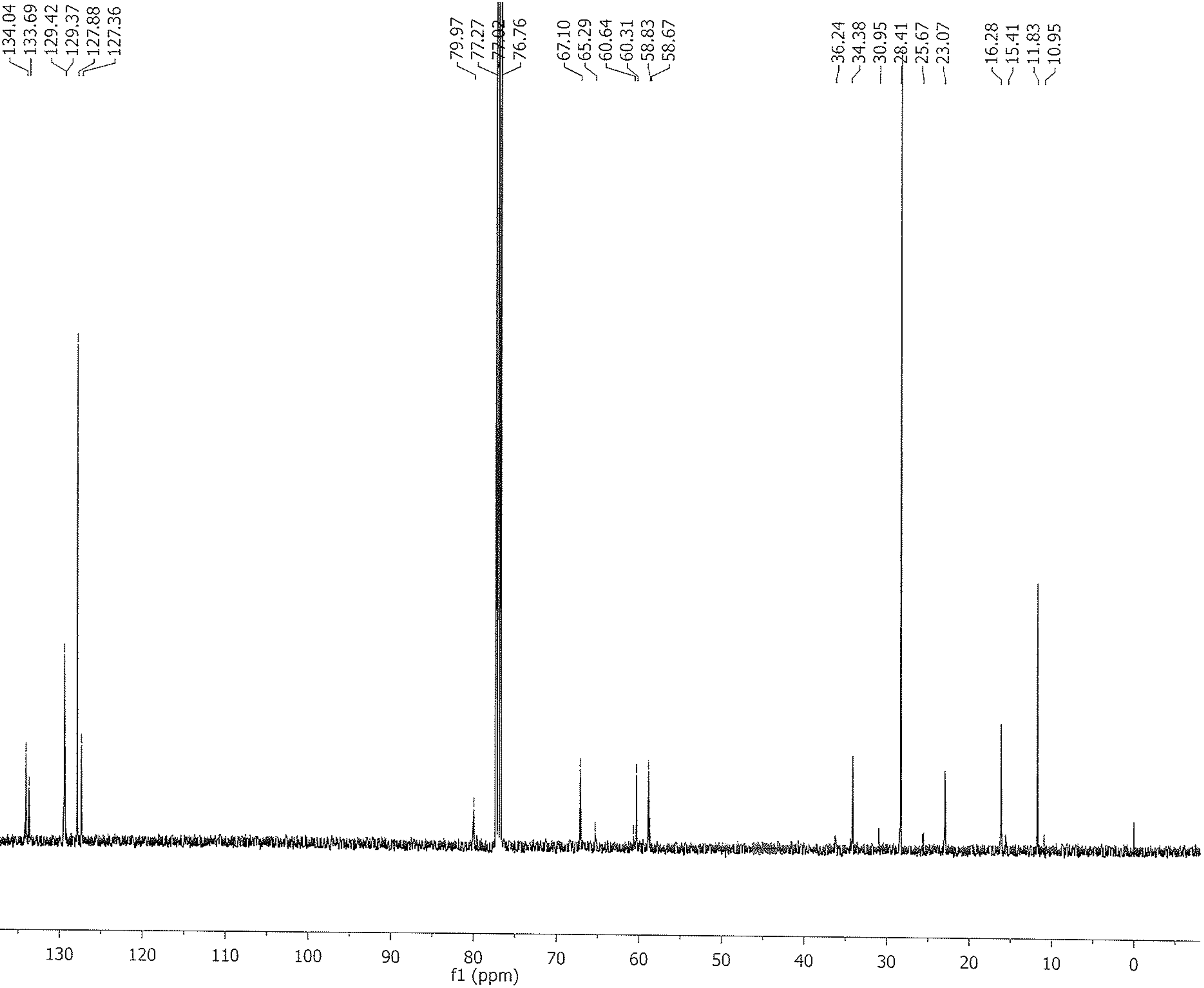

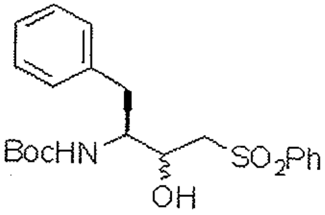

4e

$CDCl_3$, 500MHz

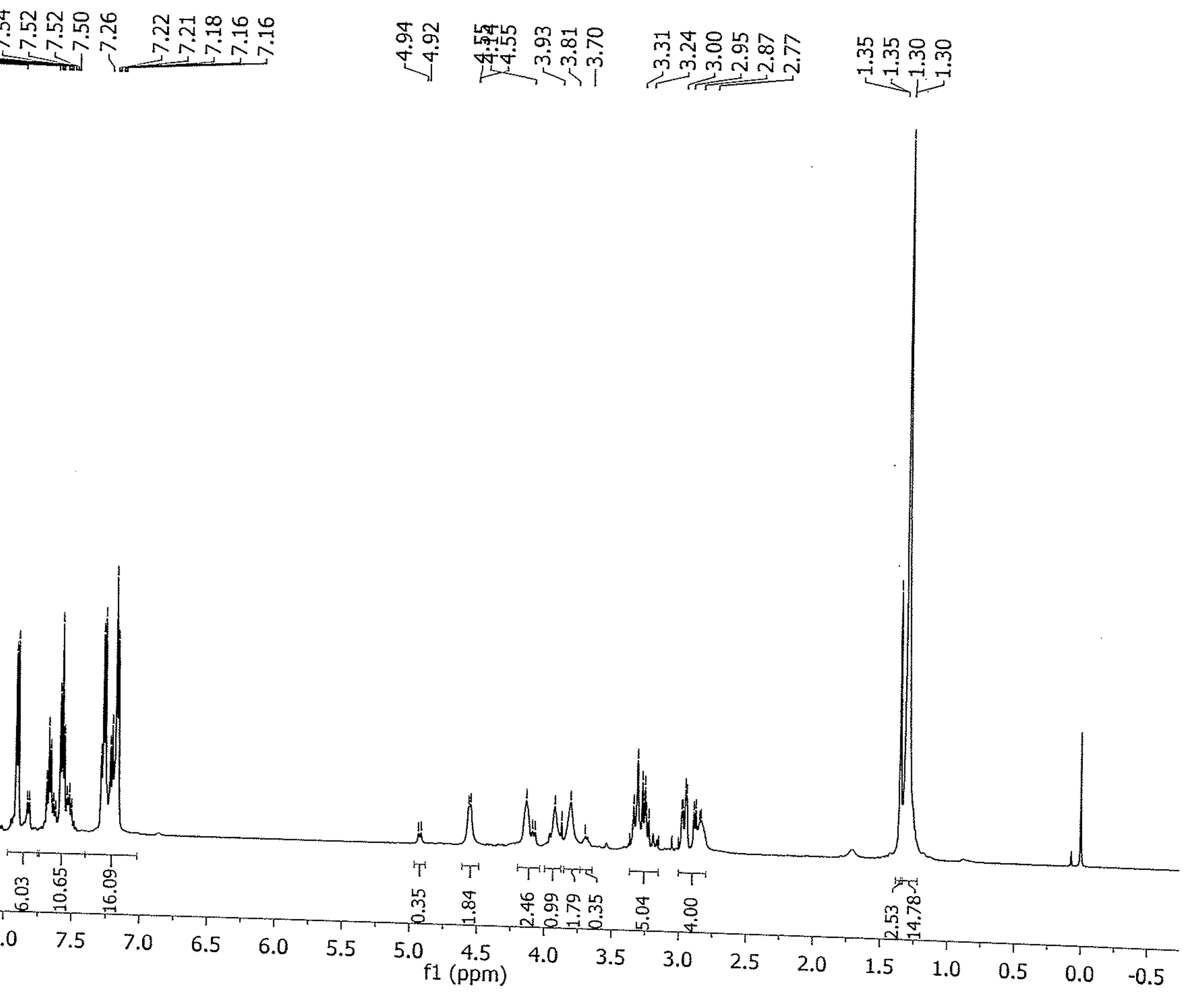

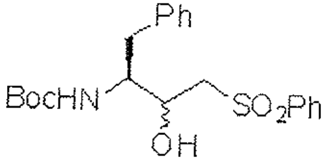

4e

$CDCl_3$, 125.68 MHz

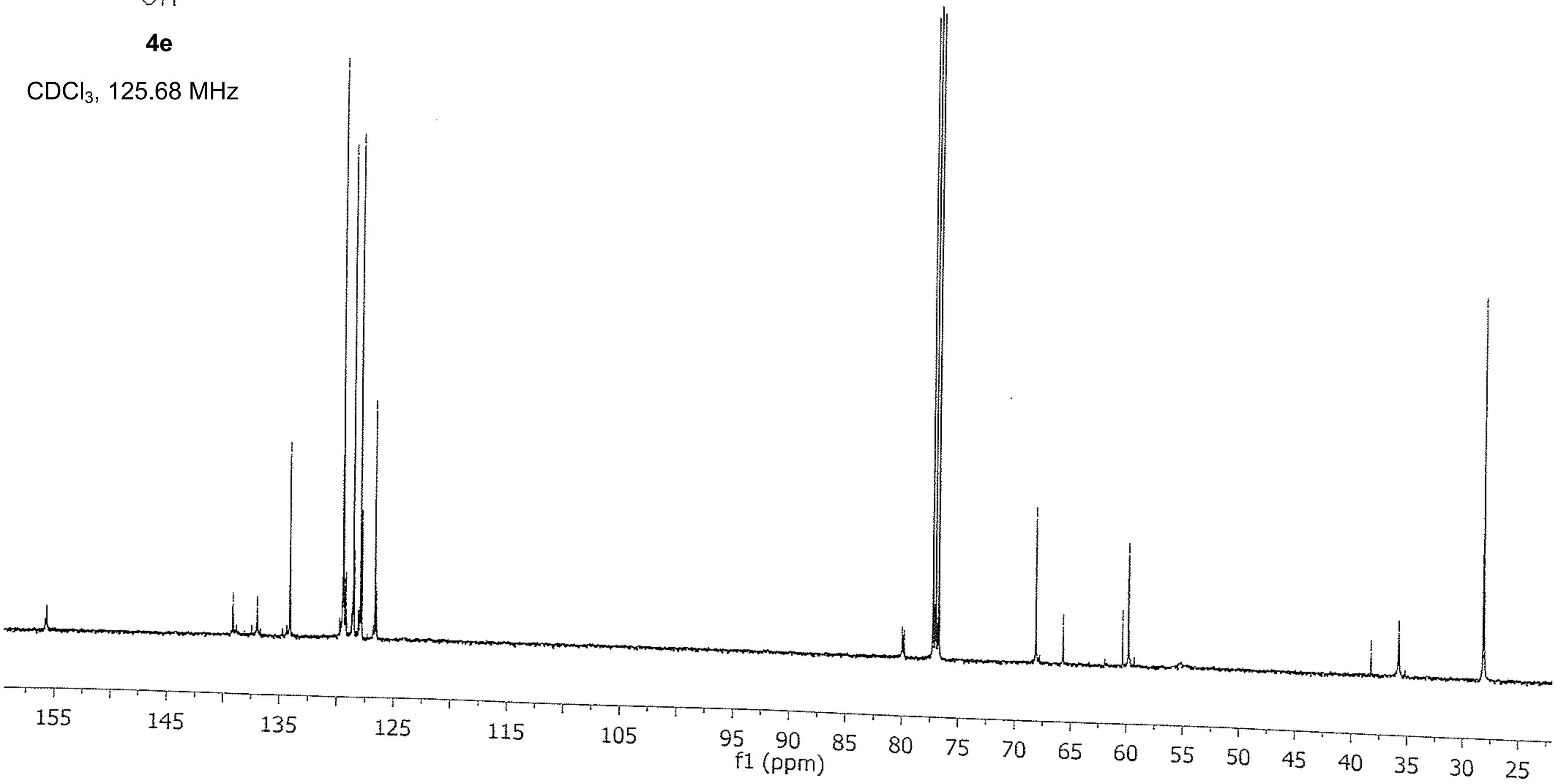

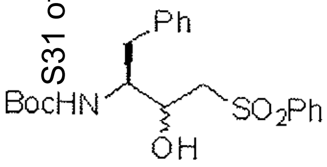

Ph
BocHN
SO$_2$Ph
OH

4e

CDCl$_3$

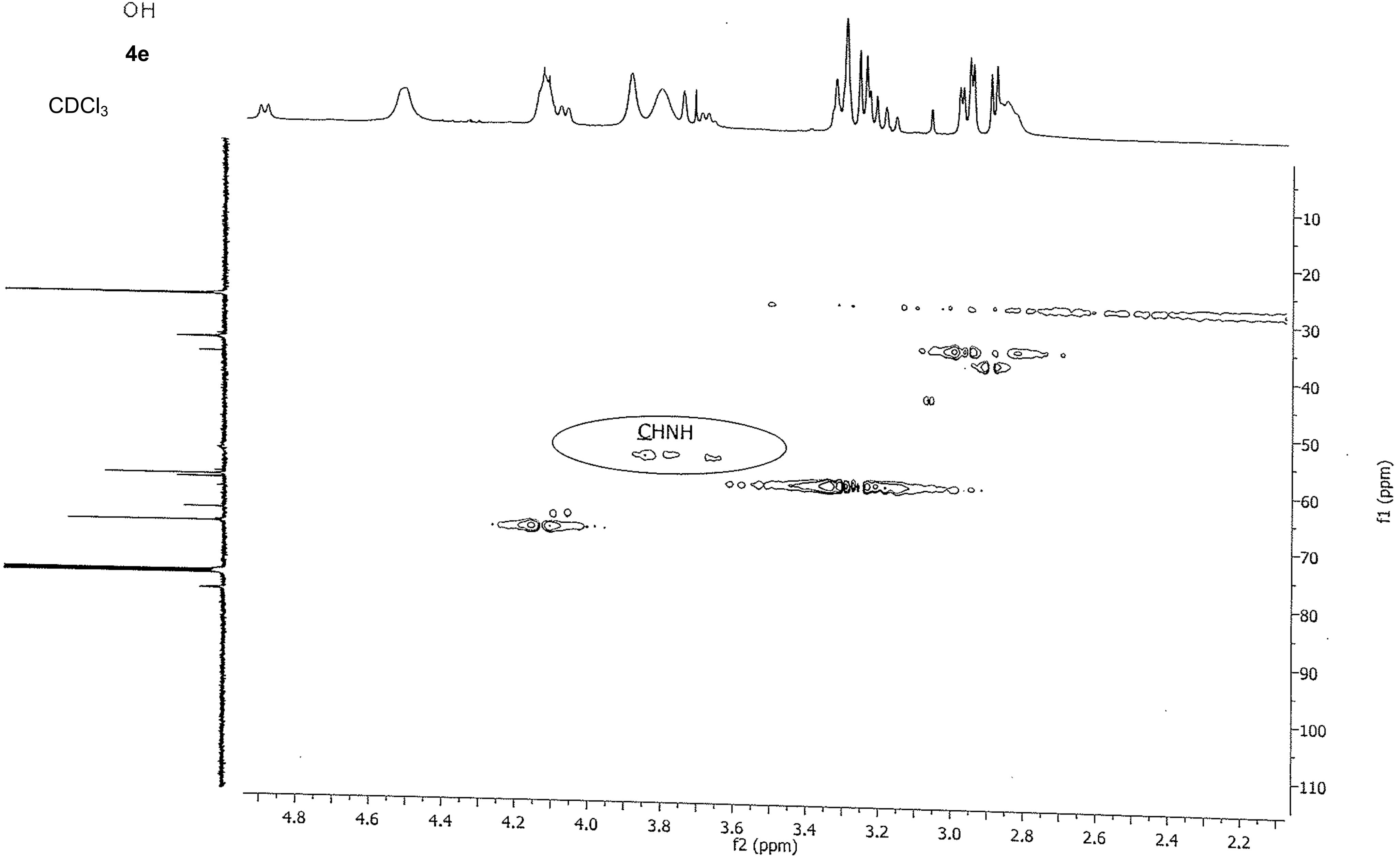

CHNH
4.8 4.6 4.4 4.2 4.0 3.8 3.6 3.4 3.2 3.0 2.8 2.6 2.4 2.2
f2 (ppm)
10 20 30 40 50 60 70 80 90 100 110
f1 (ppm)

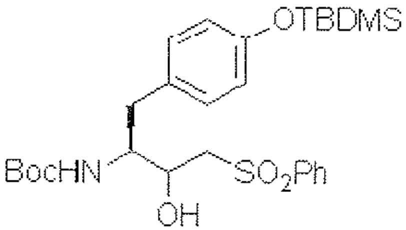

4f

CDCl3, 500MHz

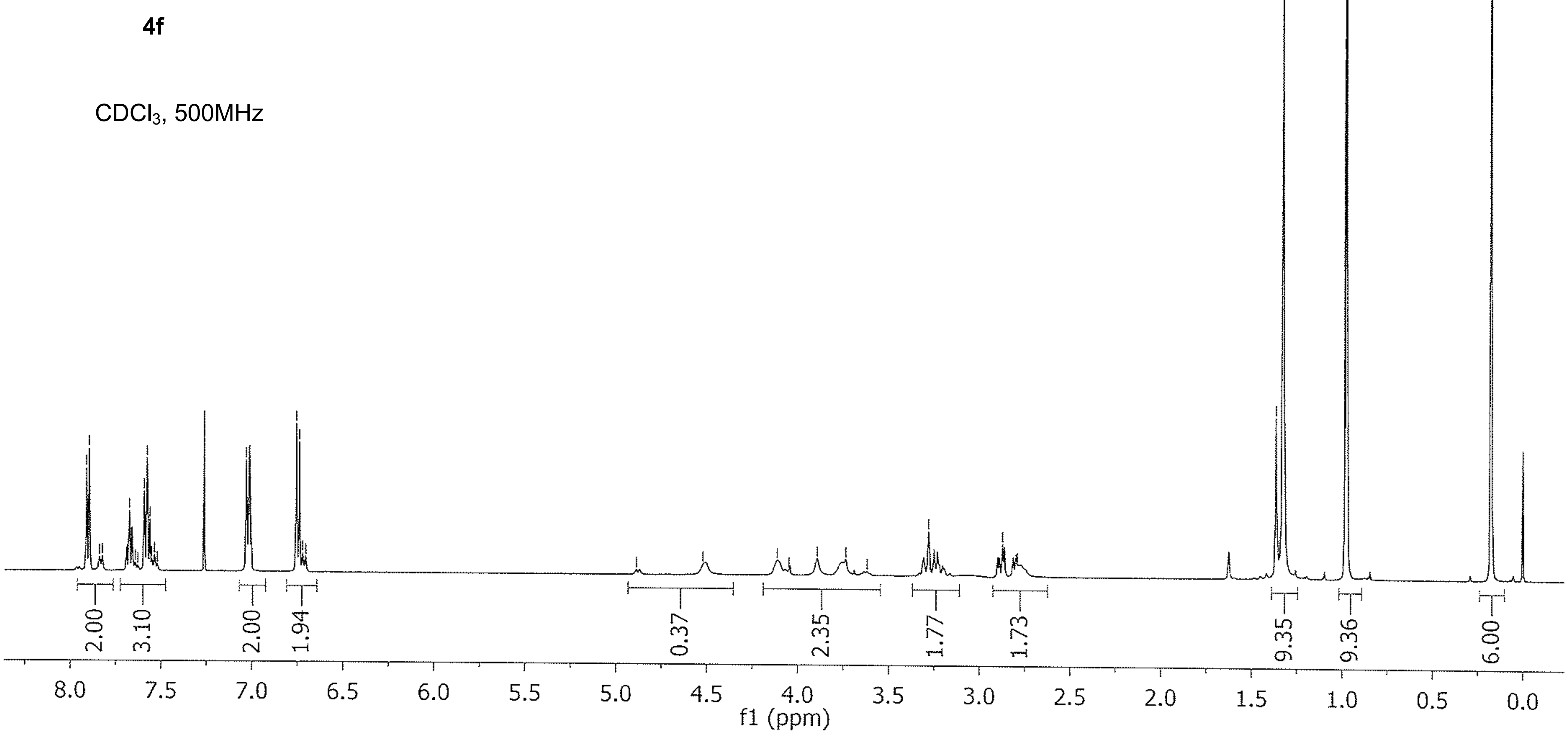

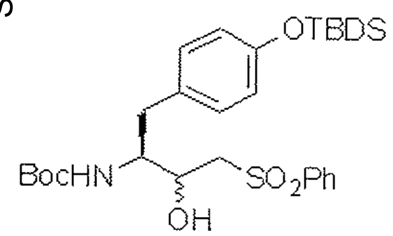

4f

$CDCl_3$, 125.68 MHz

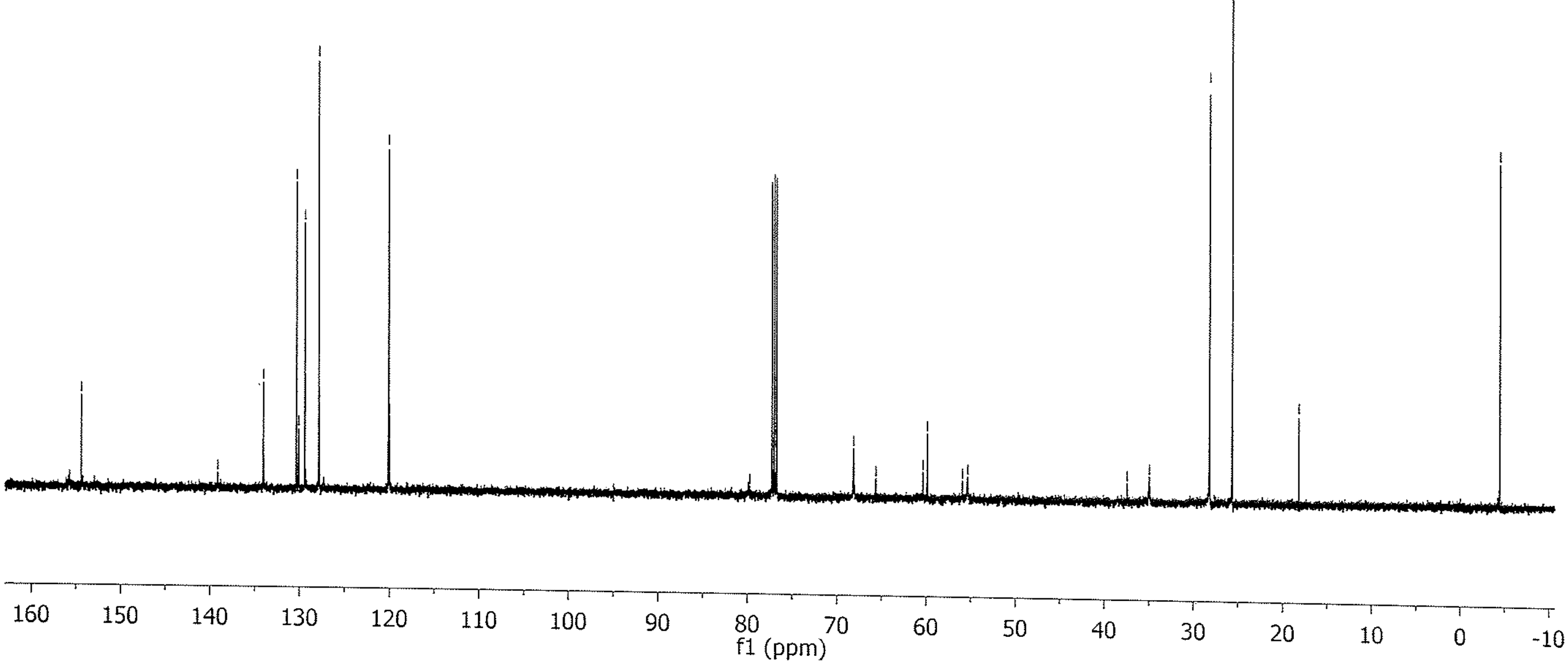

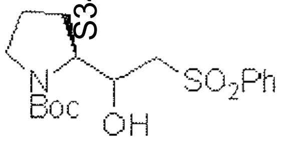

4g

dmso-d6

80°C

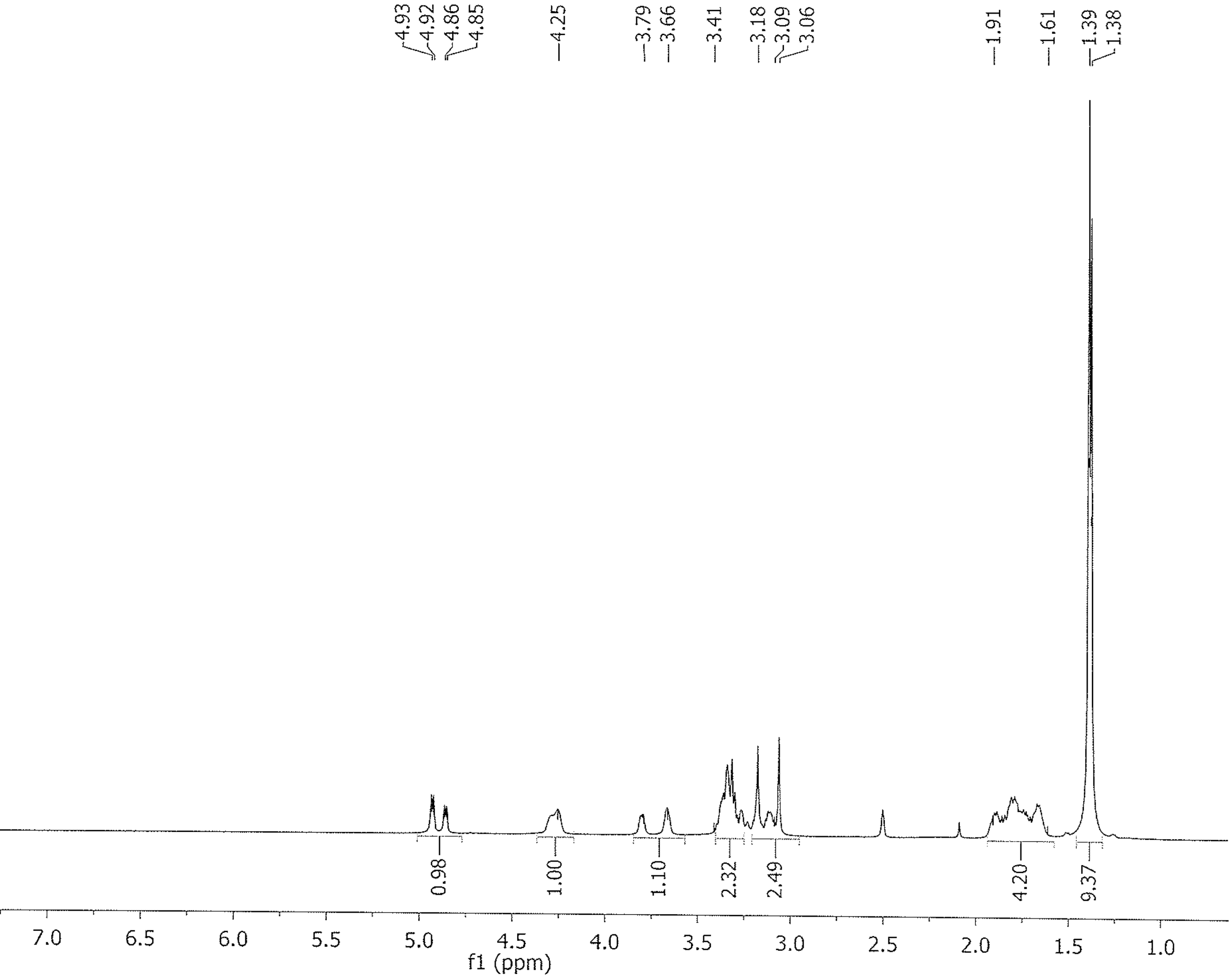

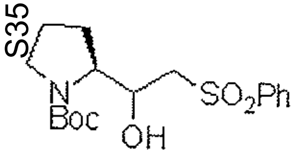

4g

dmso-d6
80°C

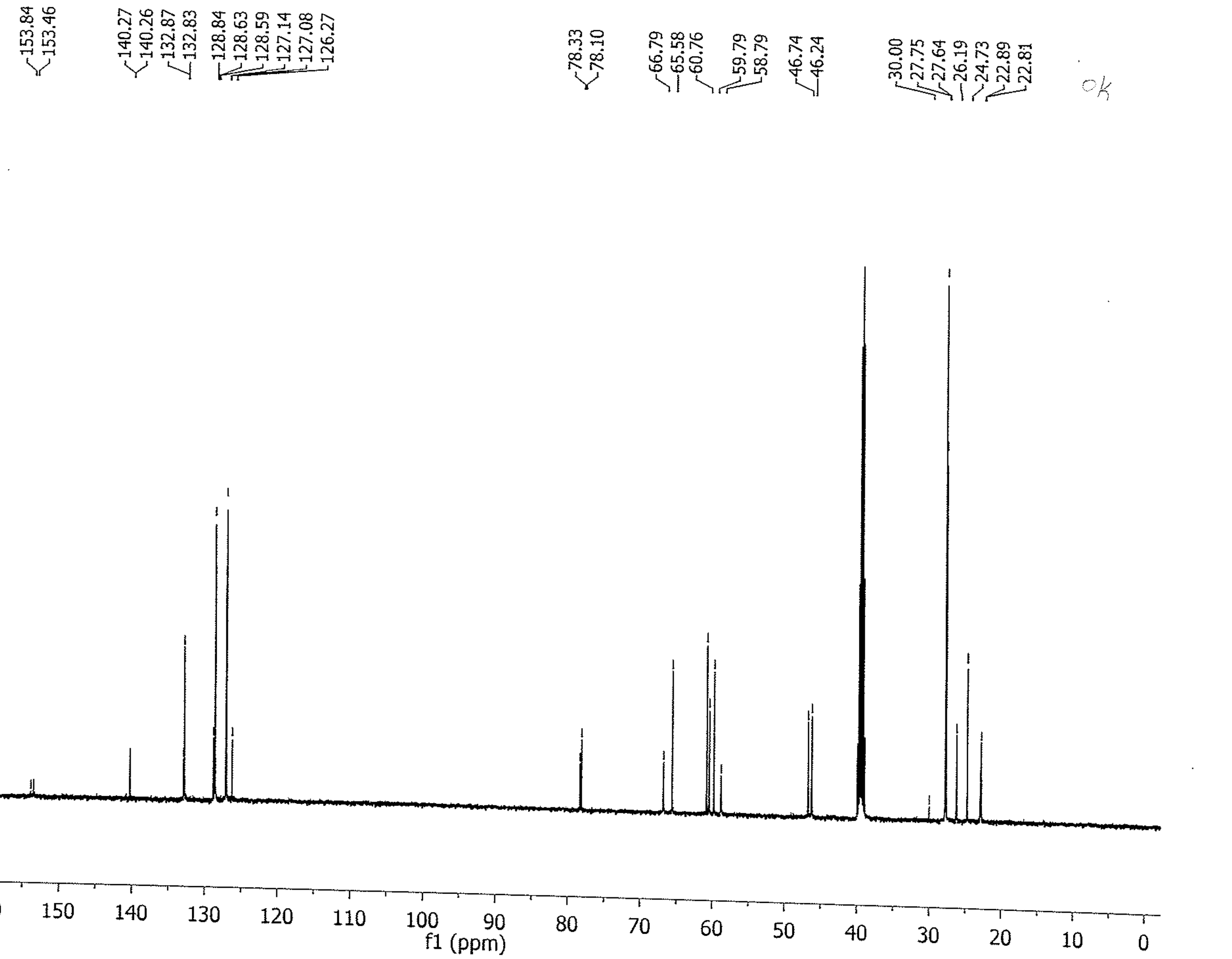

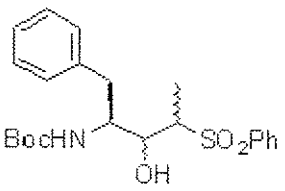

4h

$CDCl_3$, 500MHz

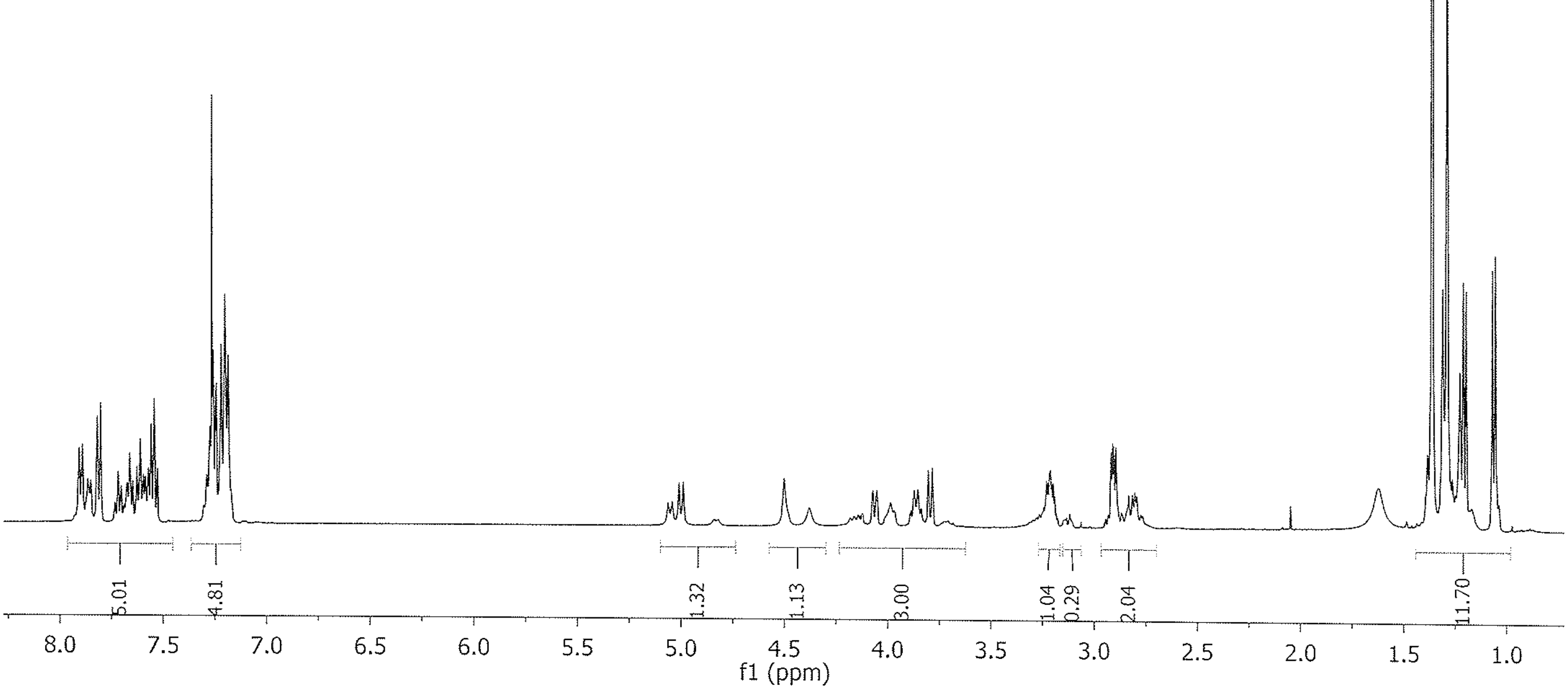

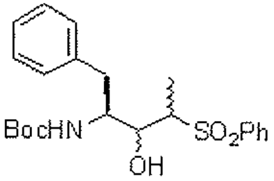

4h

$CDCl_3$, 125.68 MHz

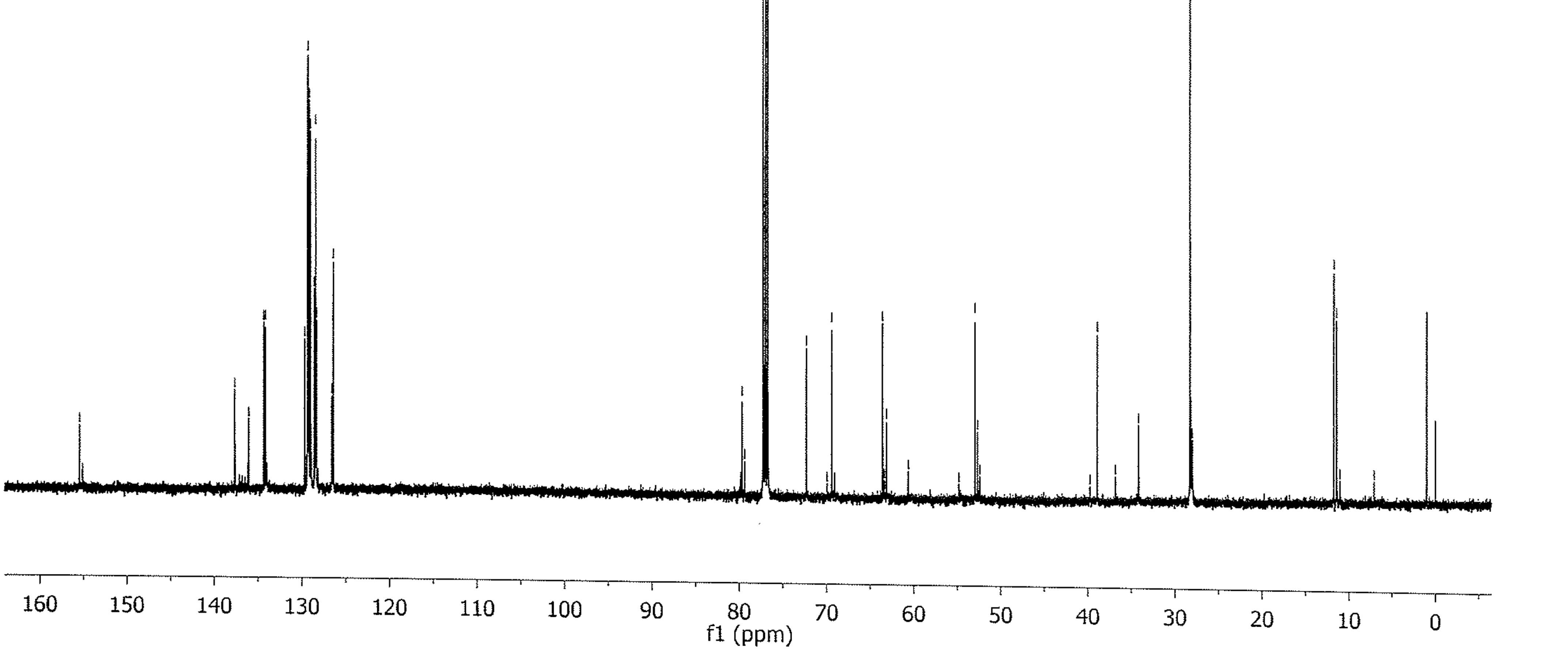

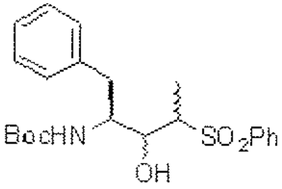
BocHN
OH
SO2Ph

4h

$CDCl_3$

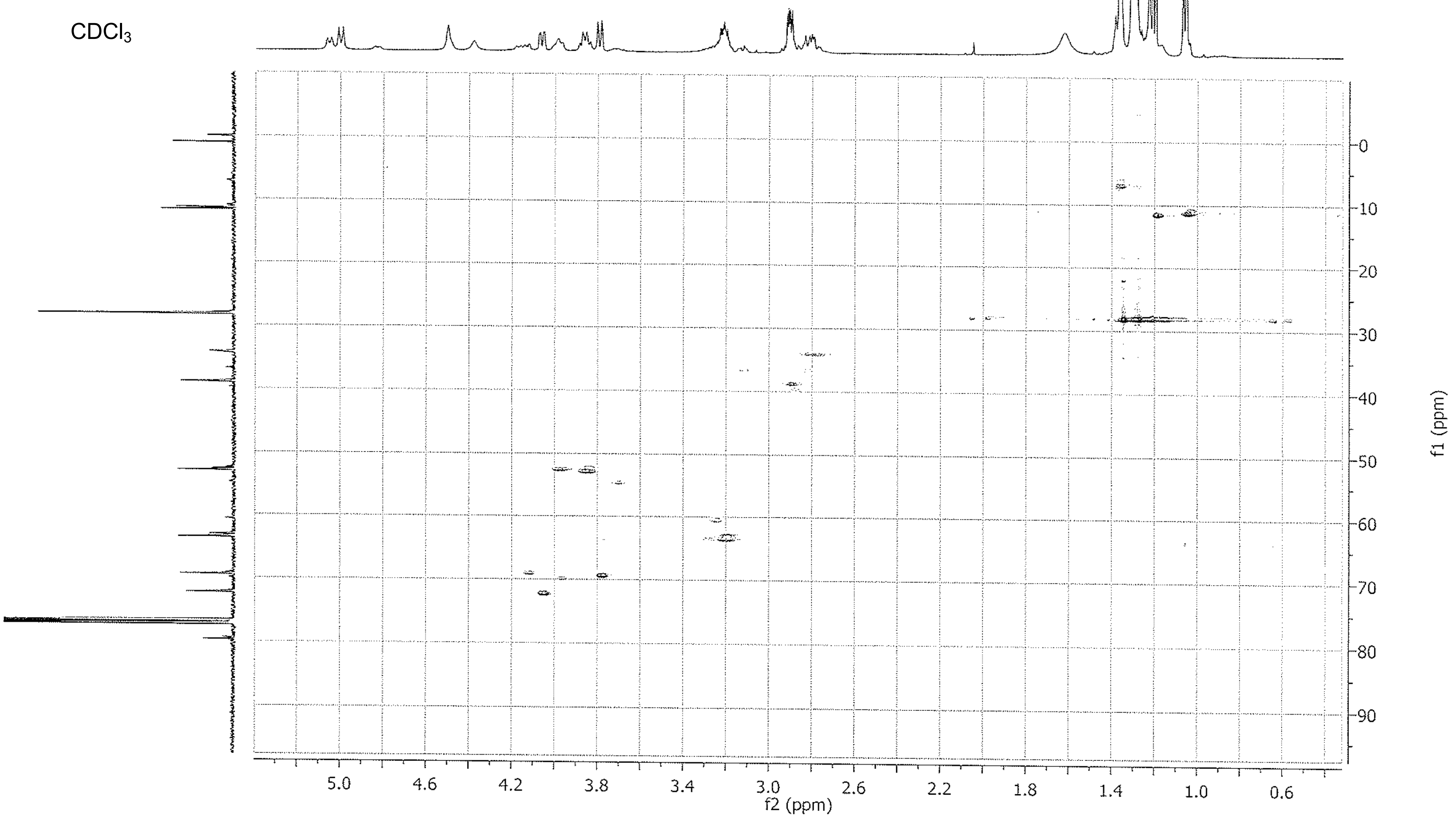
f2 (ppm)
f1 (ppm)
5.0
4.6
4.2
3.8
3.4
3.0
2.6
2.2
1.8
1.4
1.0
0.6
0
10
20
30
40
50
60
70
80
90

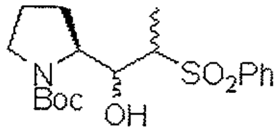

4i

$CDCl_3$, 500MHz

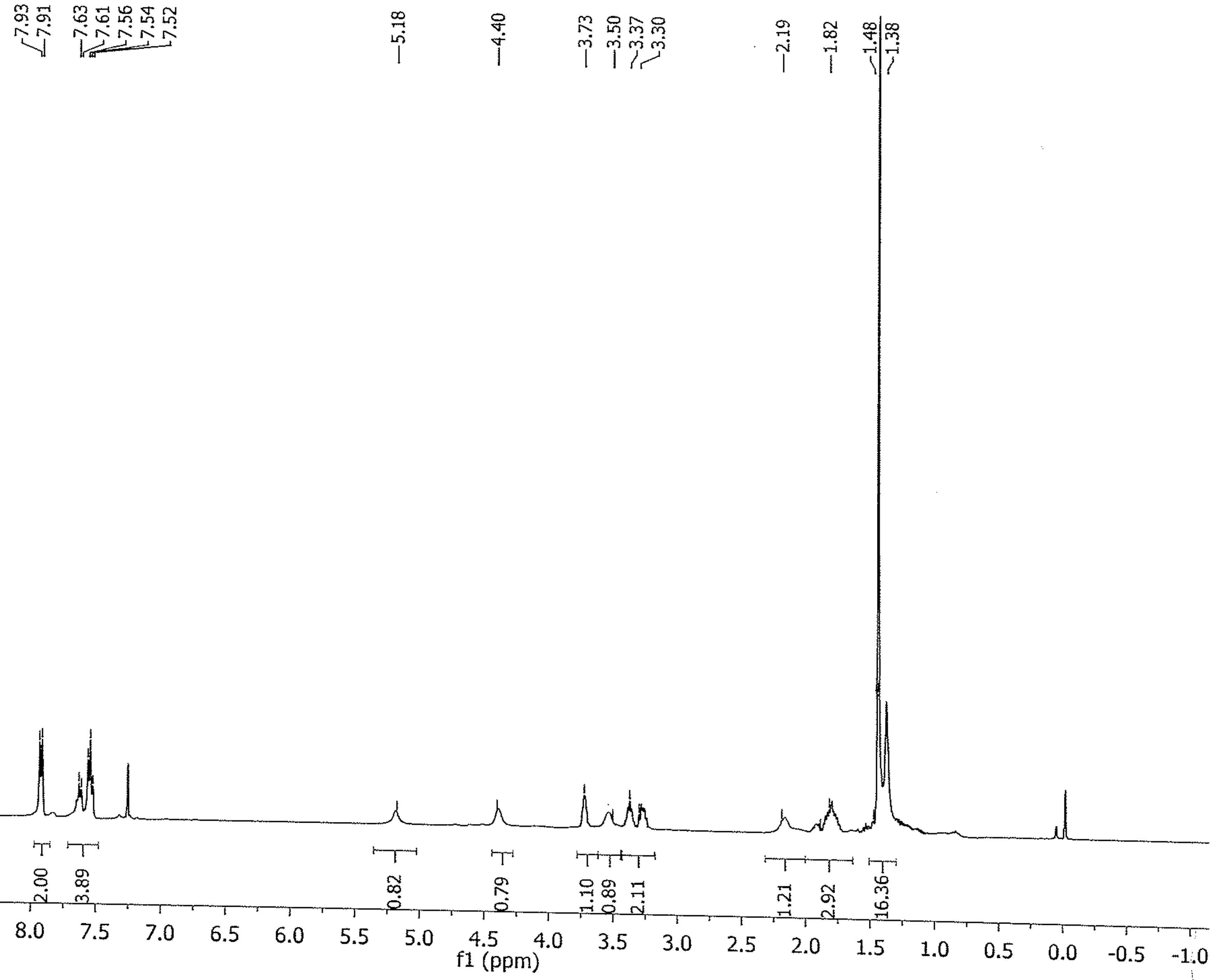

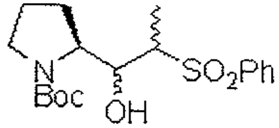

4i

$CDCl_3$, 125.68 MHz

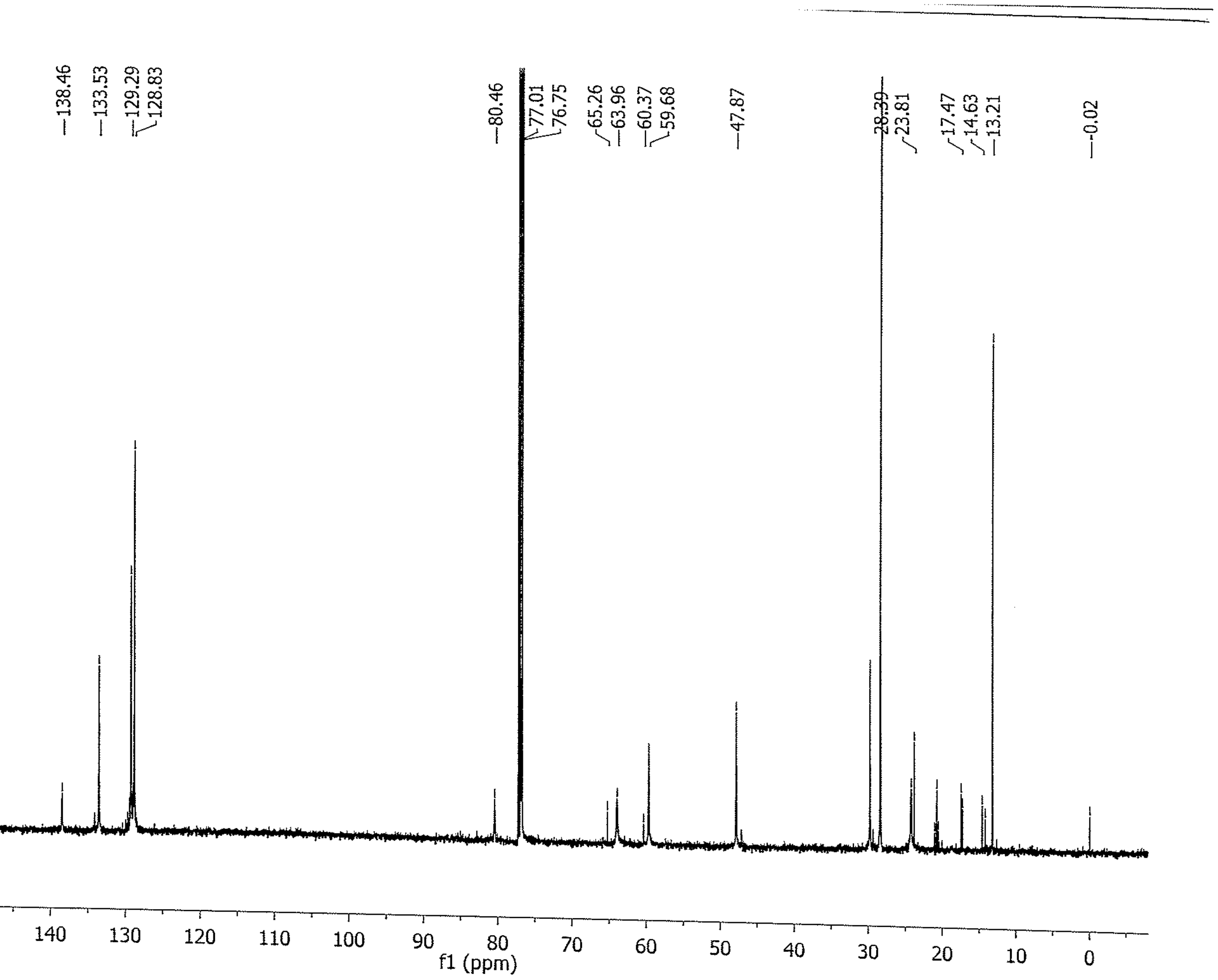

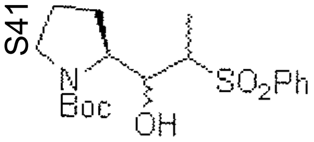
N
Boc
OH
SO_2Ph

4i

$CDCl_3$

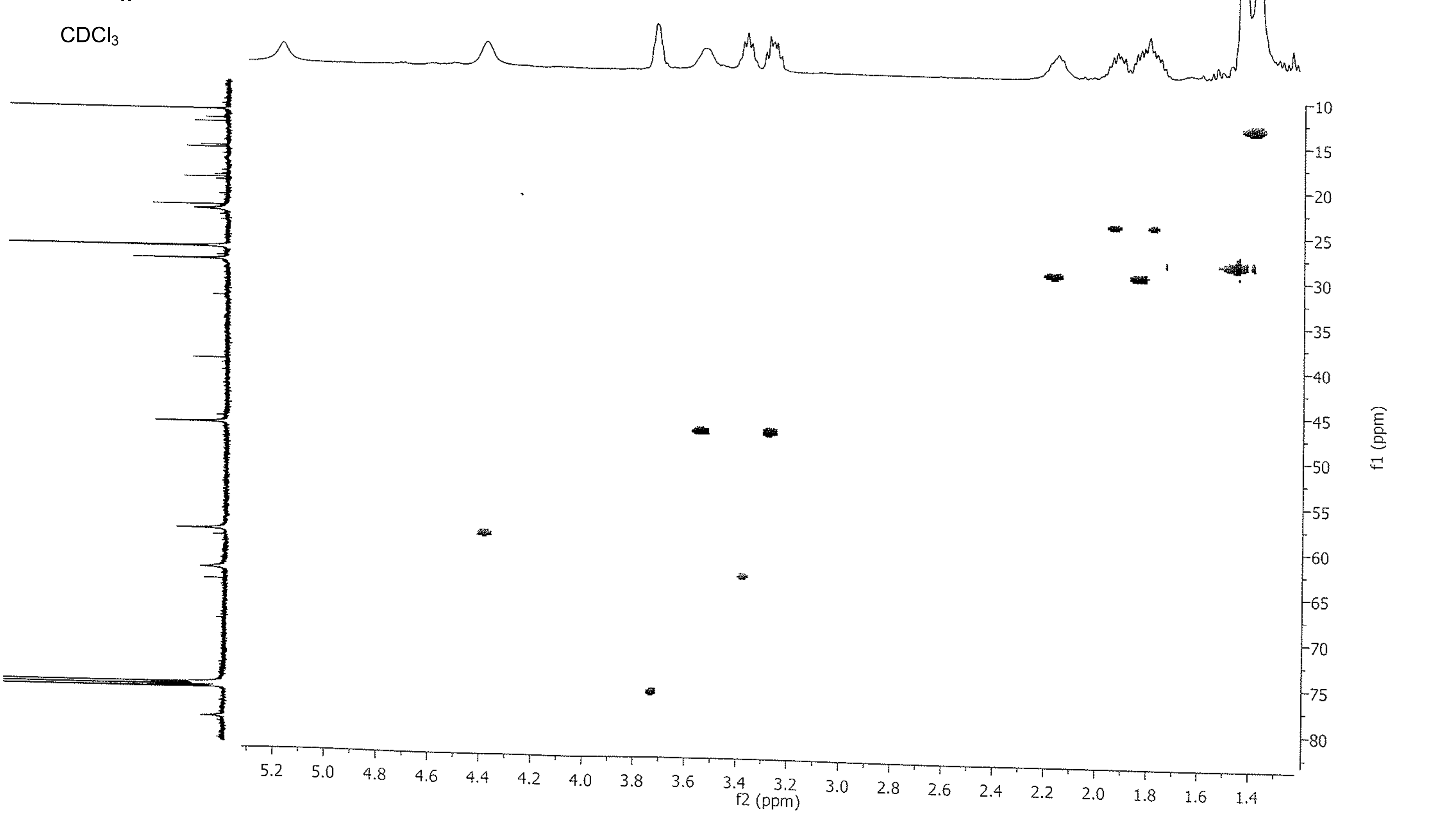
OK
5.2 5.0 4.8 4.6 4.4 4.2 4.0 3.8 3.6 3.4 3.2 3.0 2.8 2.6 2.4 2.2 2.0 1.8 1.6 1.4
f2 (ppm)
10 15 20 25 30 35 40 45 50 55 60 65 70 75 80
f1 (ppm)

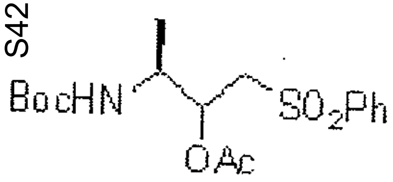

5a

$CDCl_3$, 500MHz

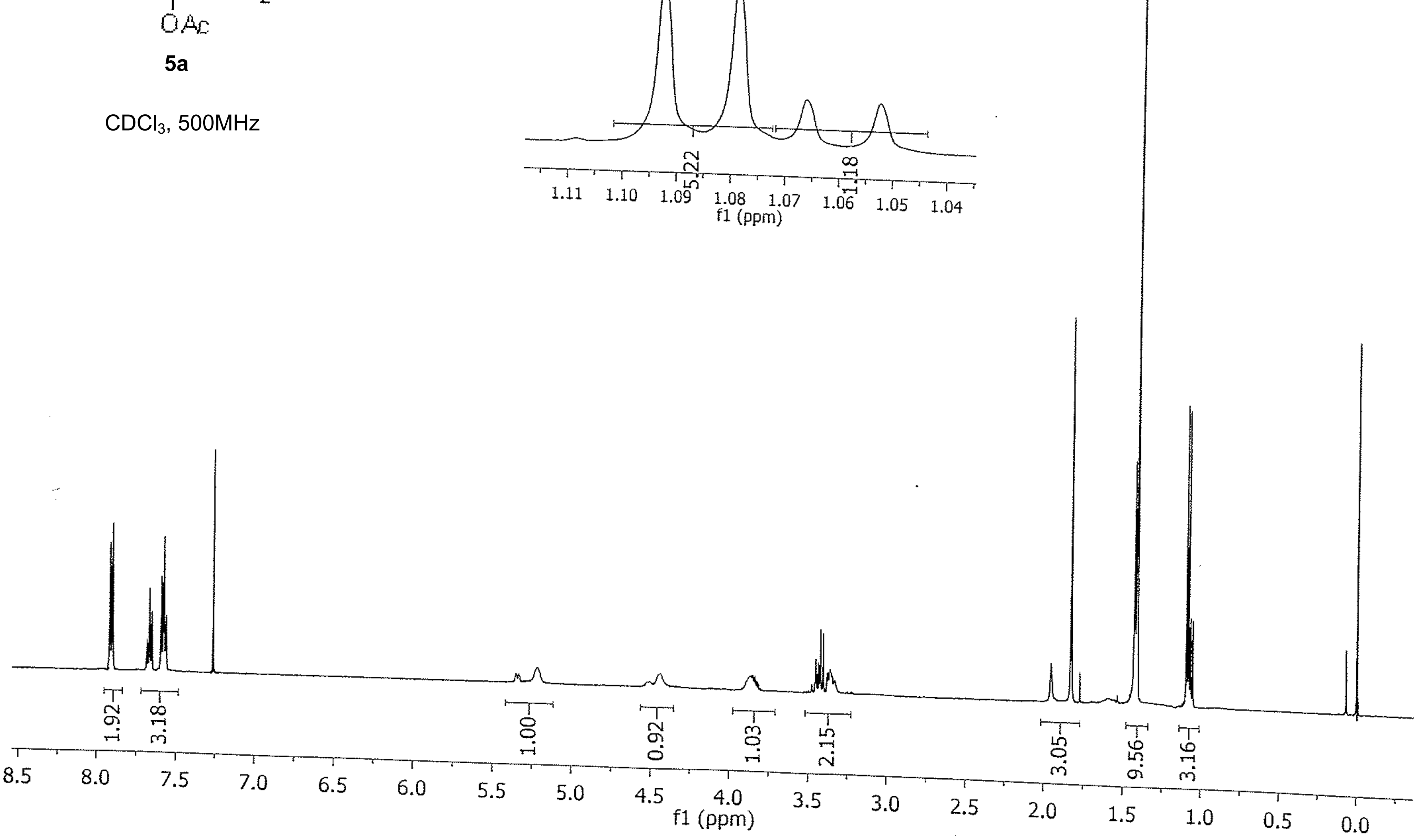

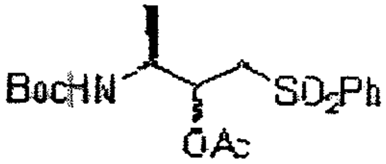

5a

$CDCl_3$, 125.68 MHz

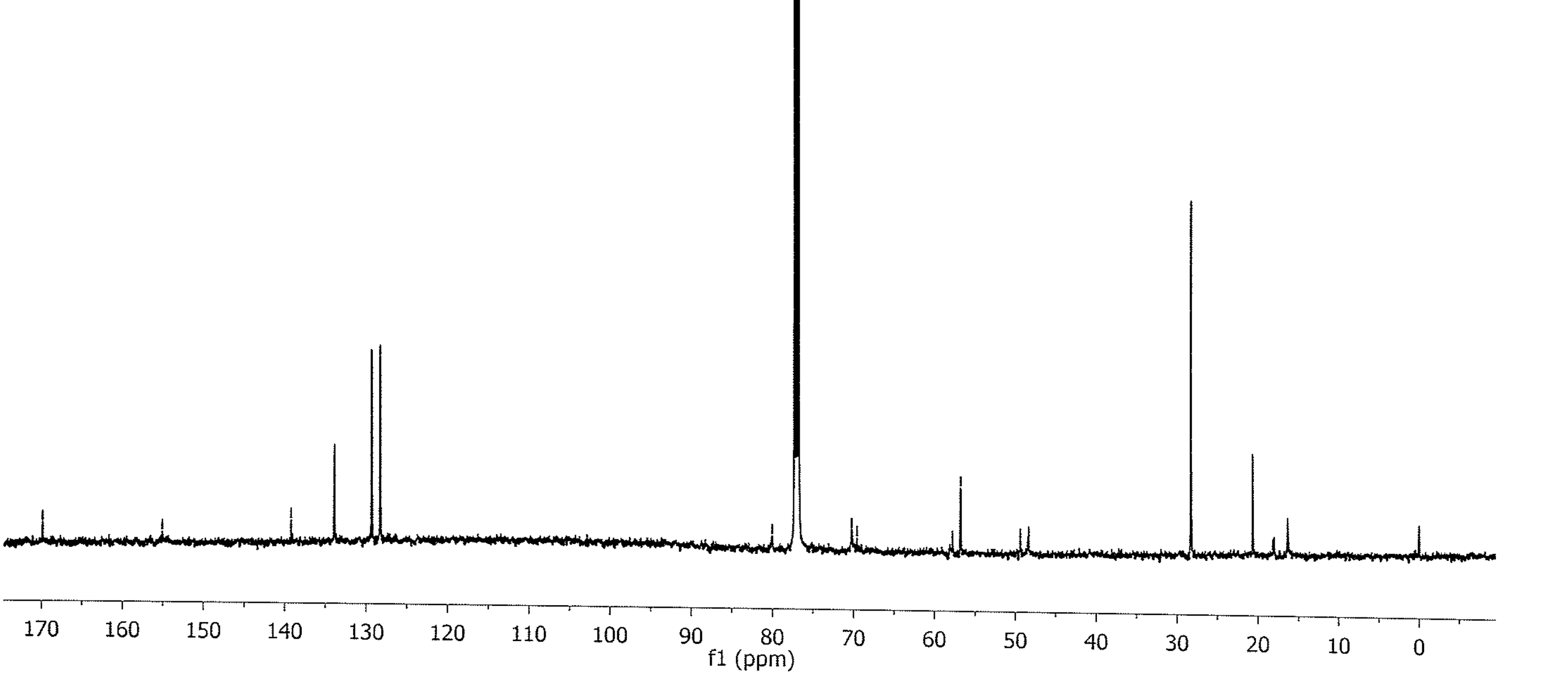

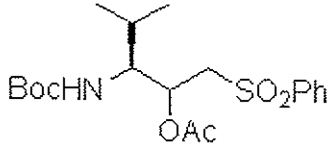

5b

$CDCl_3$, 500MHz

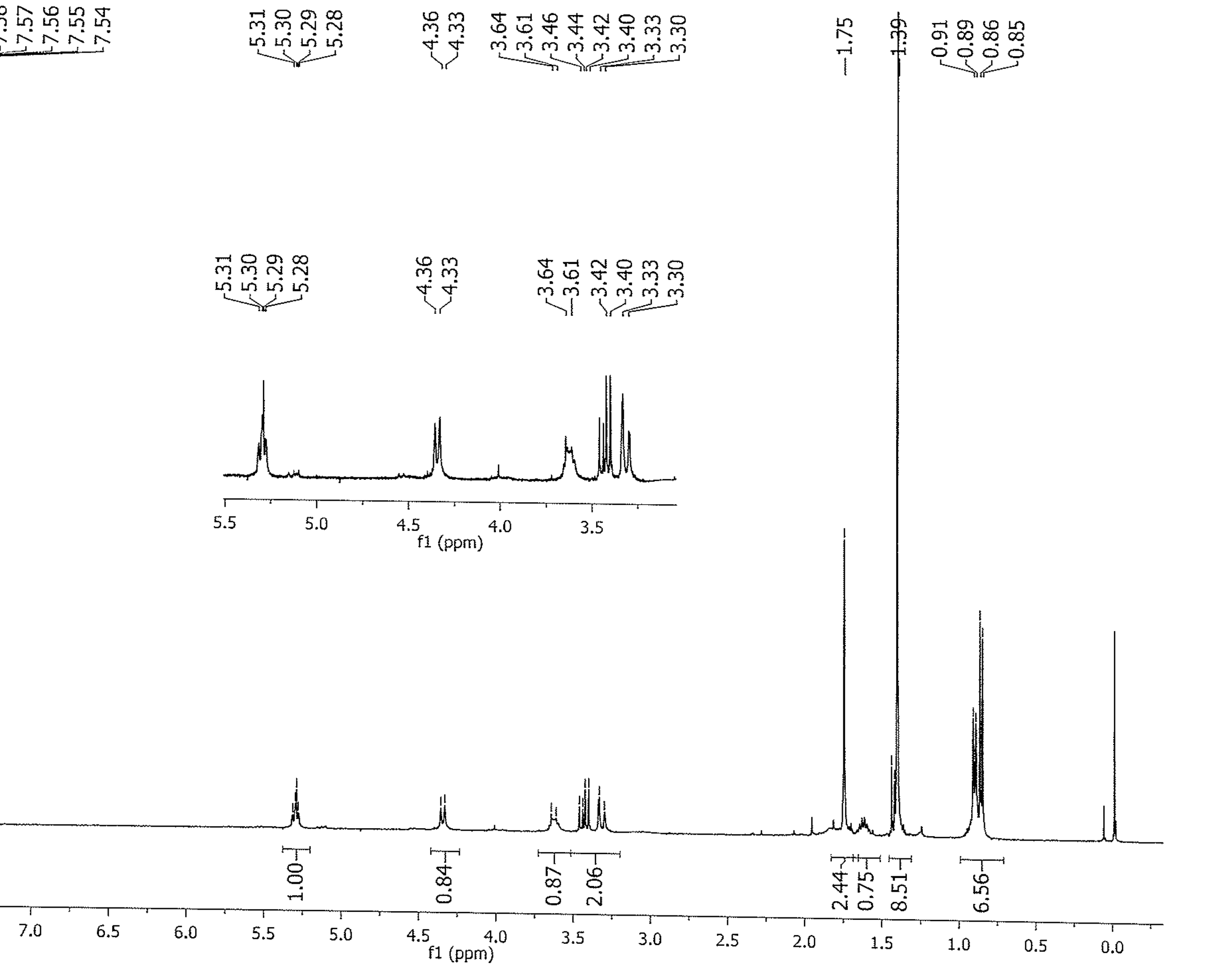

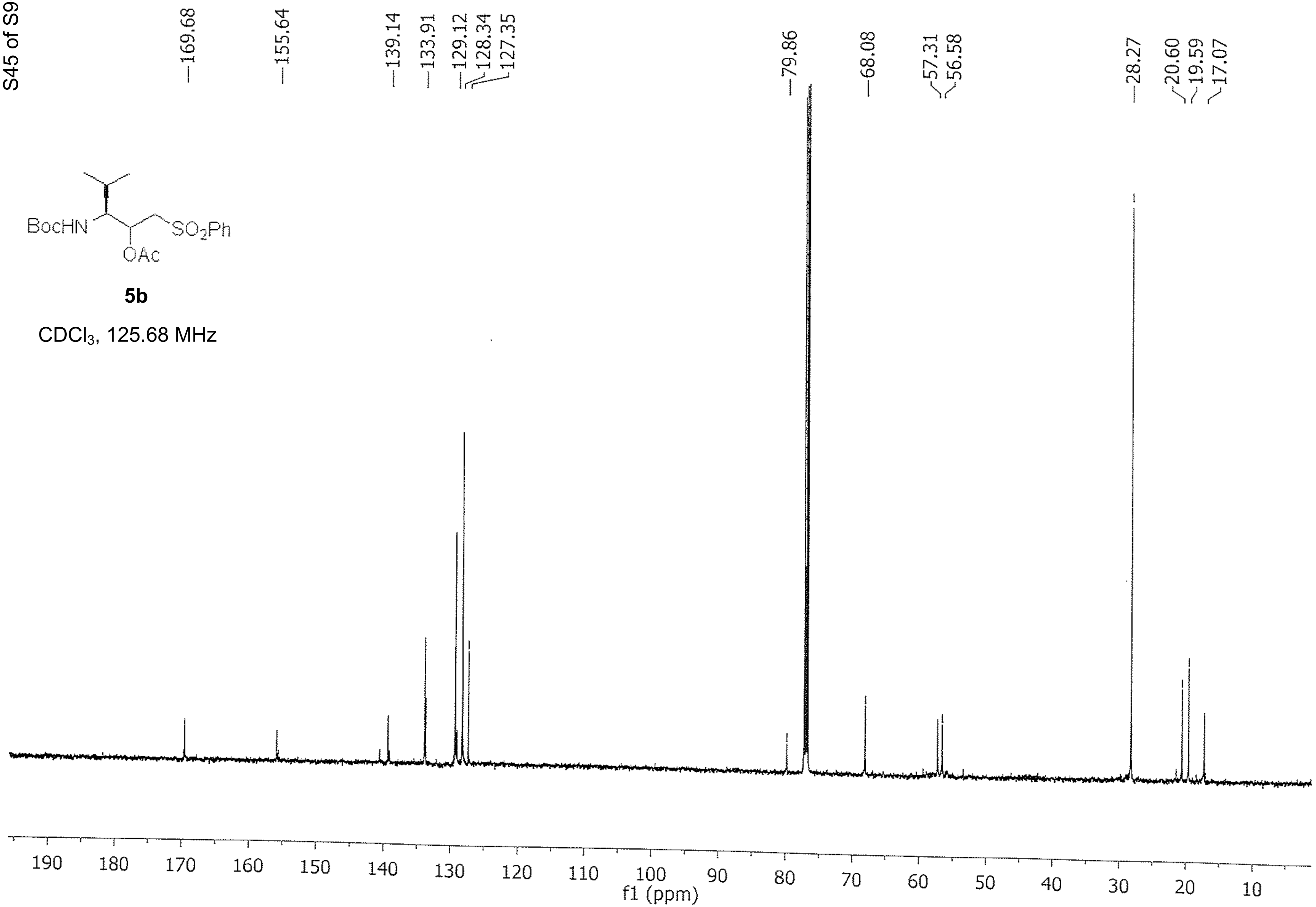

5b

$CDCl_3$, 125.68 MHz

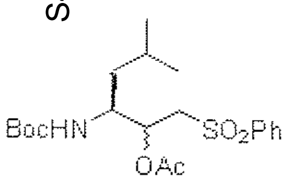

5c

$CDCl_3$, 500MHz

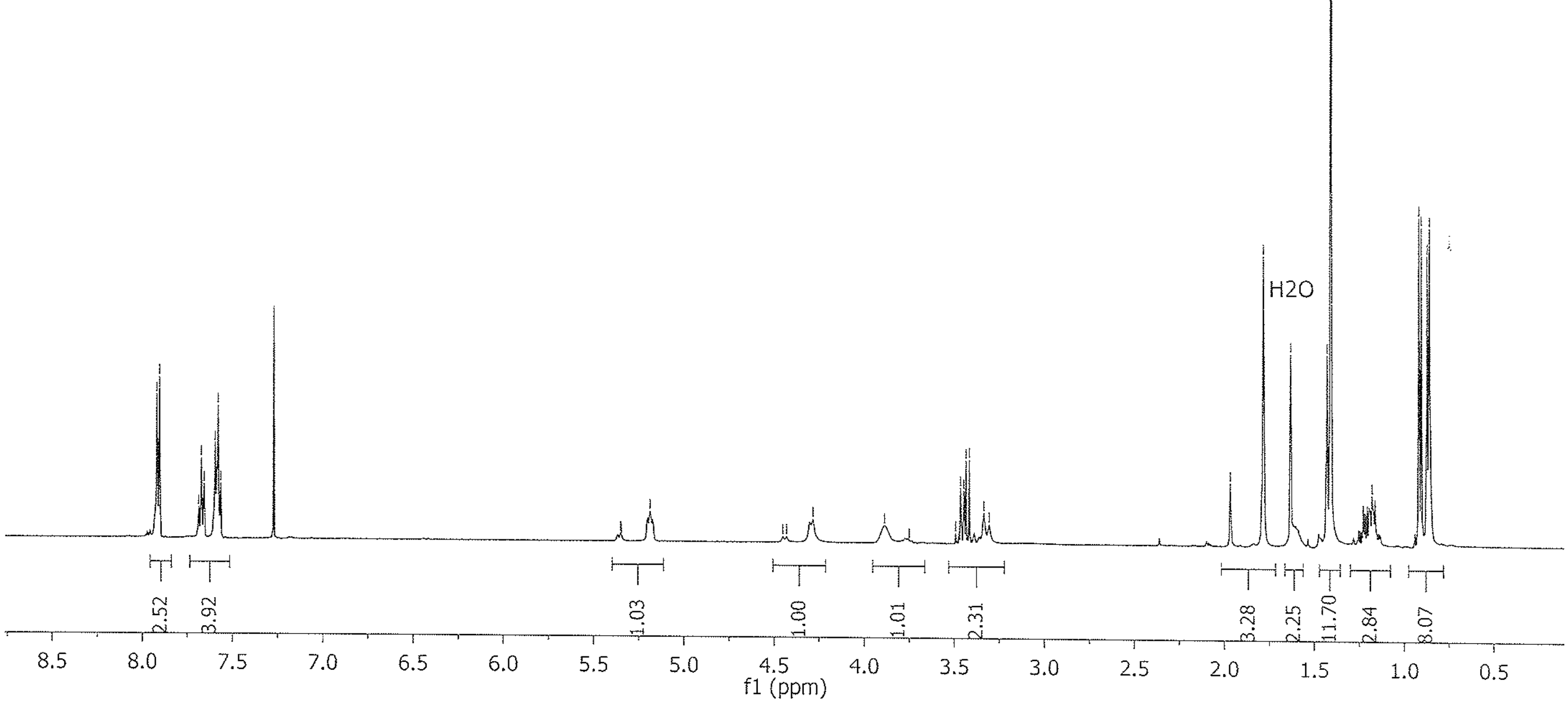

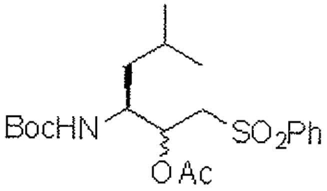

5c

$CDCl_3$, 125.68 MHz

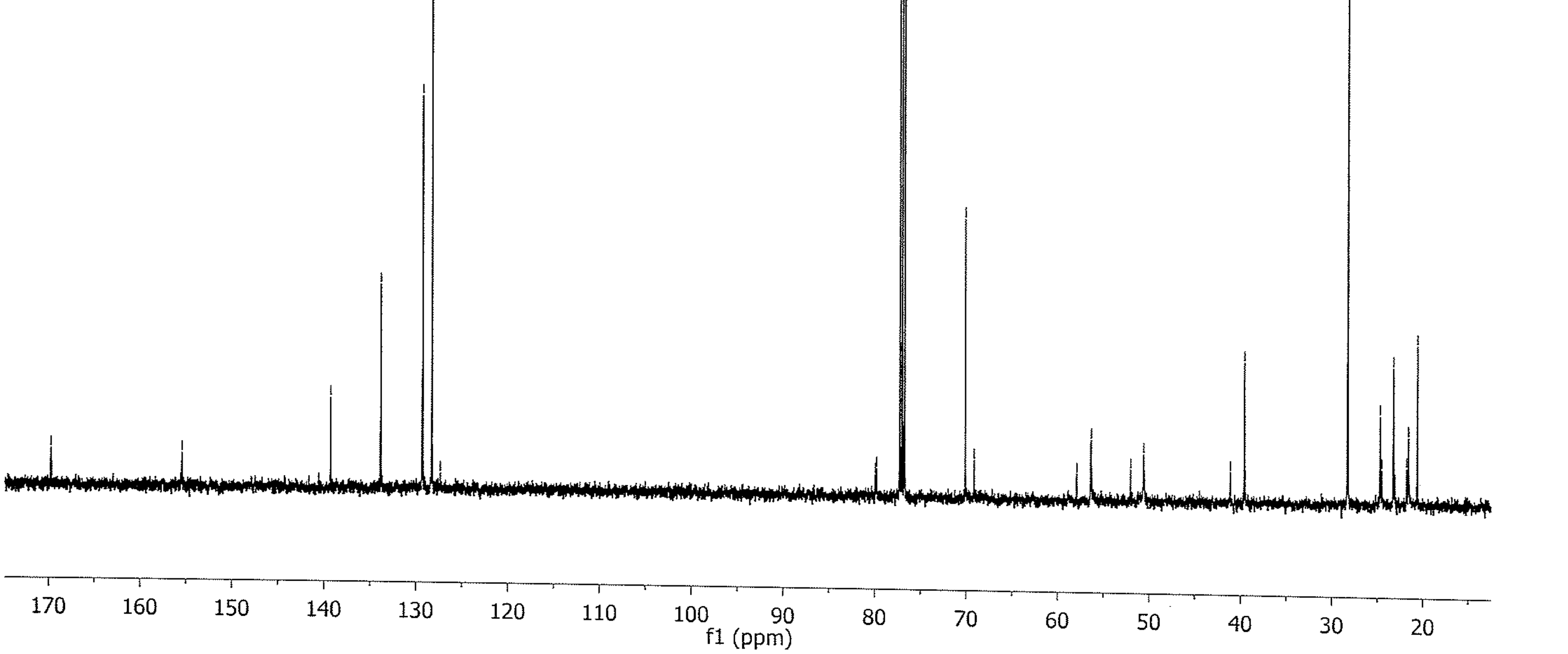

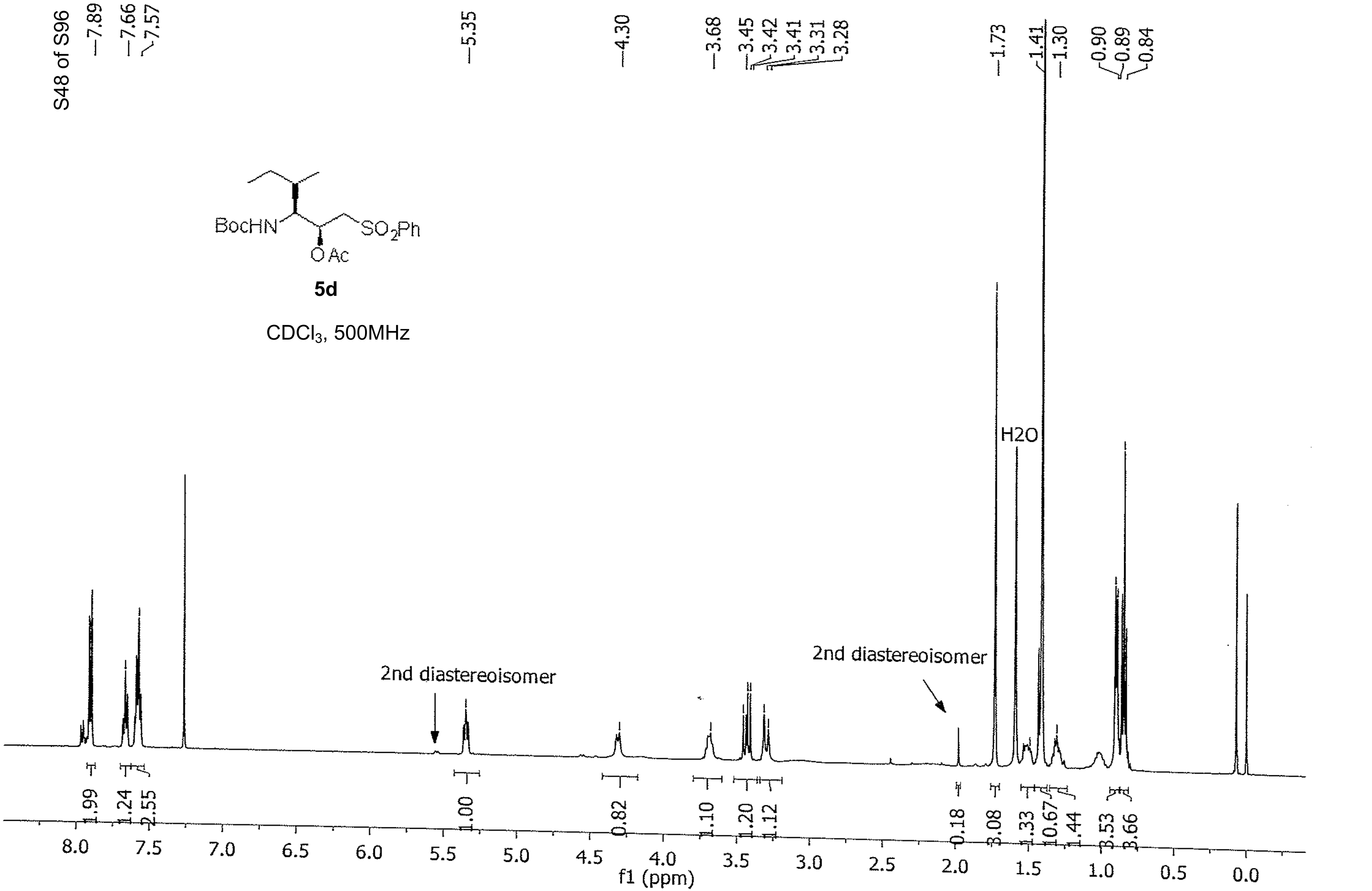

7.89
7.66
7.57
5.35
4.30
3.68
3.45
3.42
3.41
3.31
3.28
1.73
1.41
1.30
0.90
0.89
0.84
BocHN
SO2Ph
OAc
5d
CDCl3, 500MHz
H2O
2nd diastereoisomer
2nd diastereoisomer
1.99
1.24
2.55
1.00
0.82
1.10
1.20
1.12
0.18
3.08
1.33
10.67
1.44
3.53
3.66
8.0
7.5
7.0
6.5
6.0
5.5
5.0
4.5
4.0
3.5
3.0
2.5
2.0
1.5
1.0
0.5
0.0
f1 (ppm)

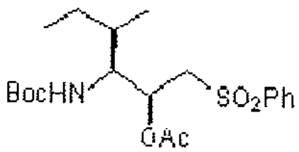

5d

$CDCl_3$, 125.68 MHz

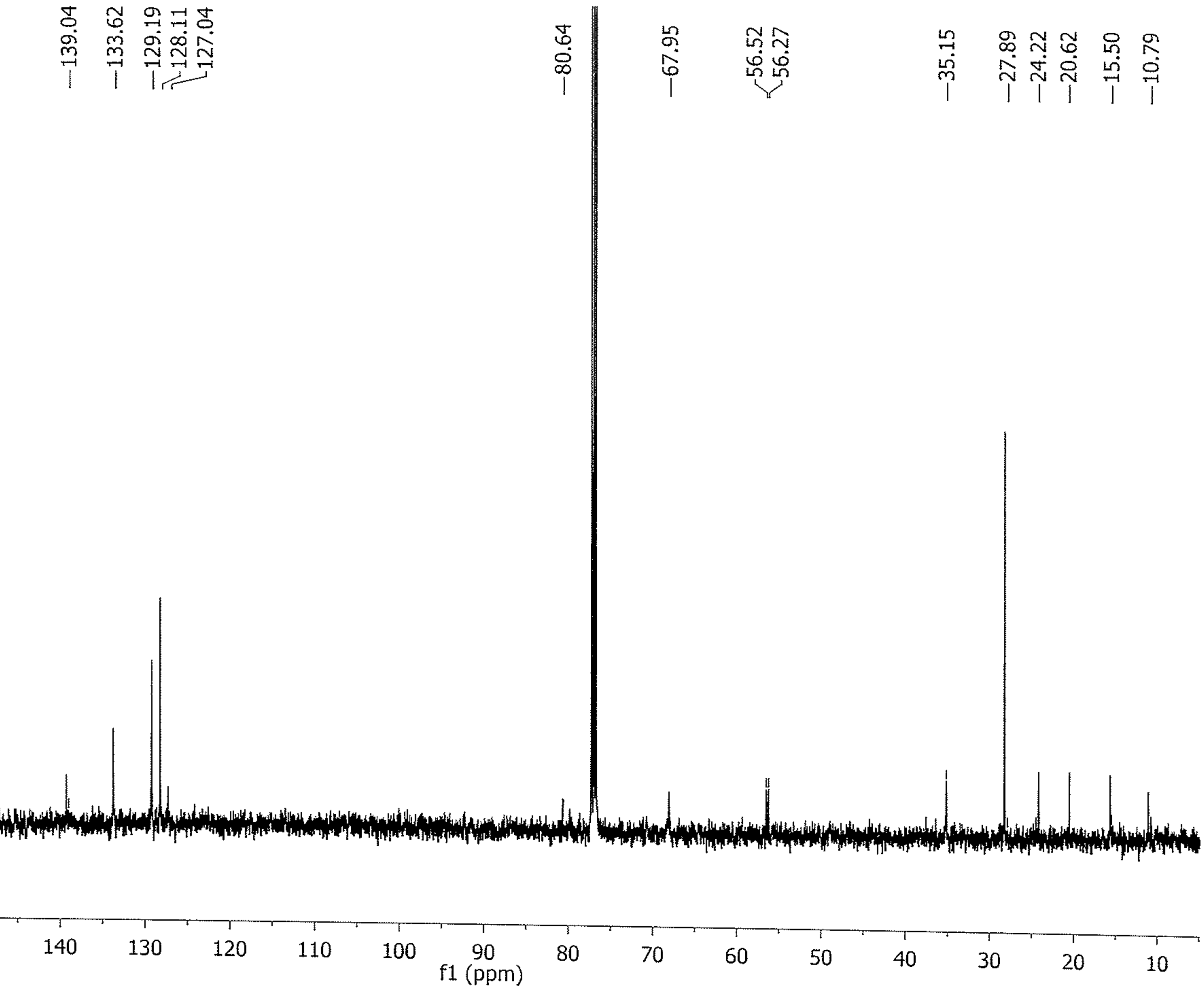

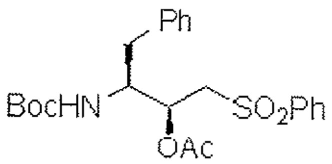

5e

1 diastereoisomer

$CDCl_3$, 500MHz

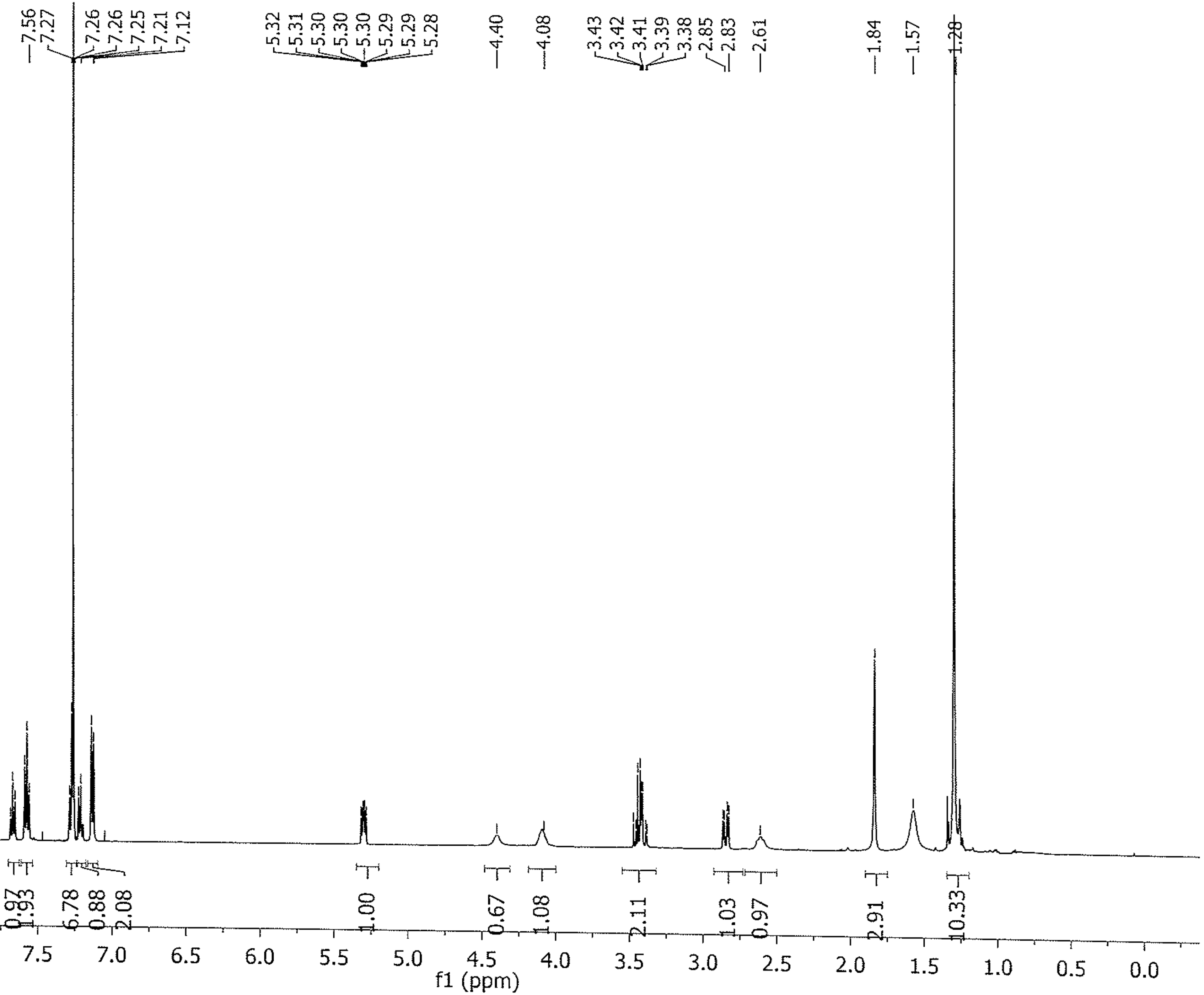

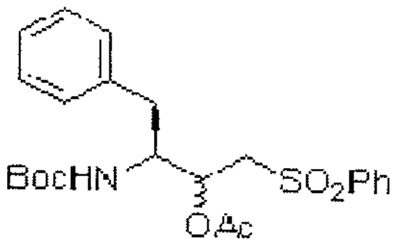

5e

$CDCl_3$, 125.68 MHz

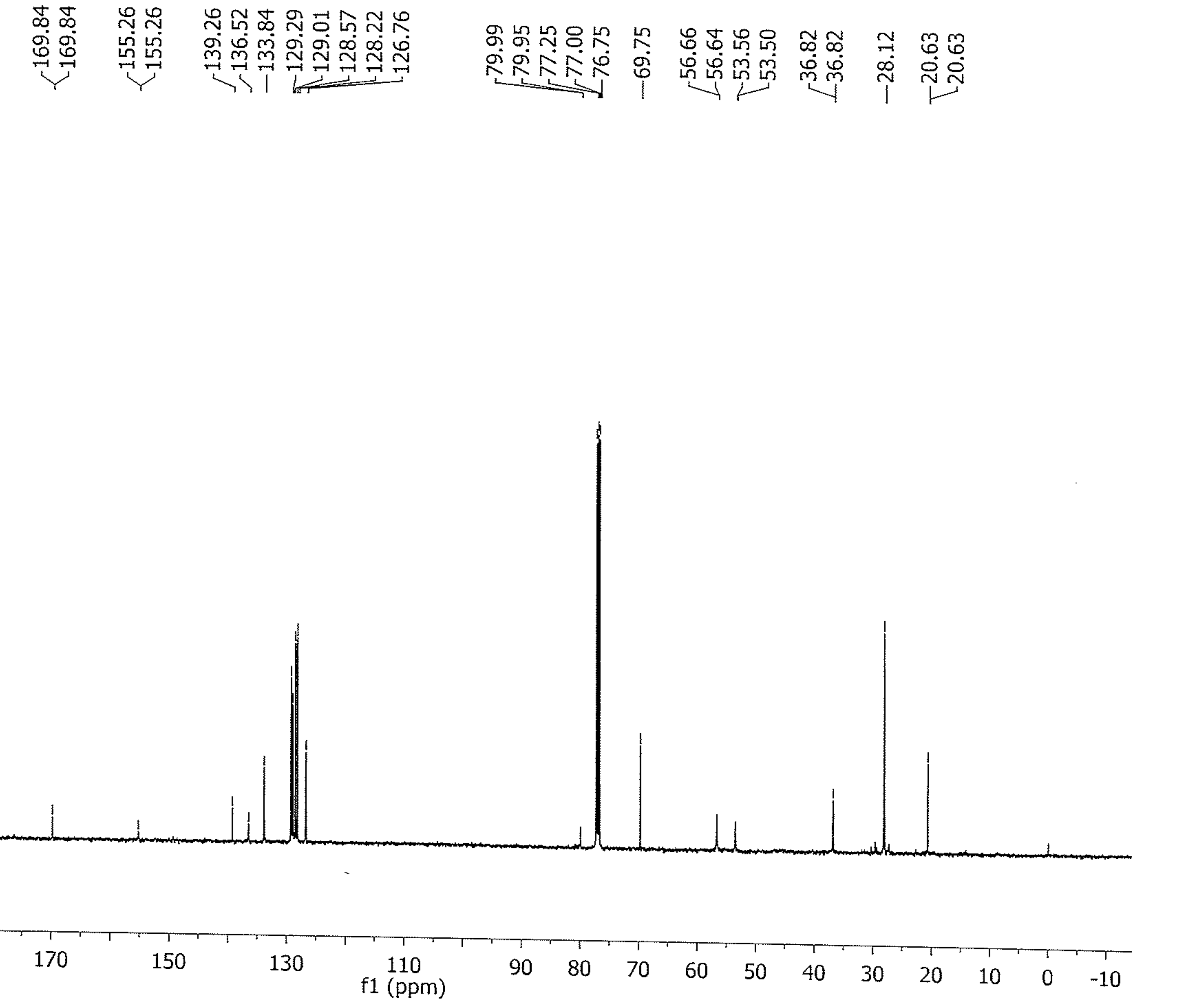

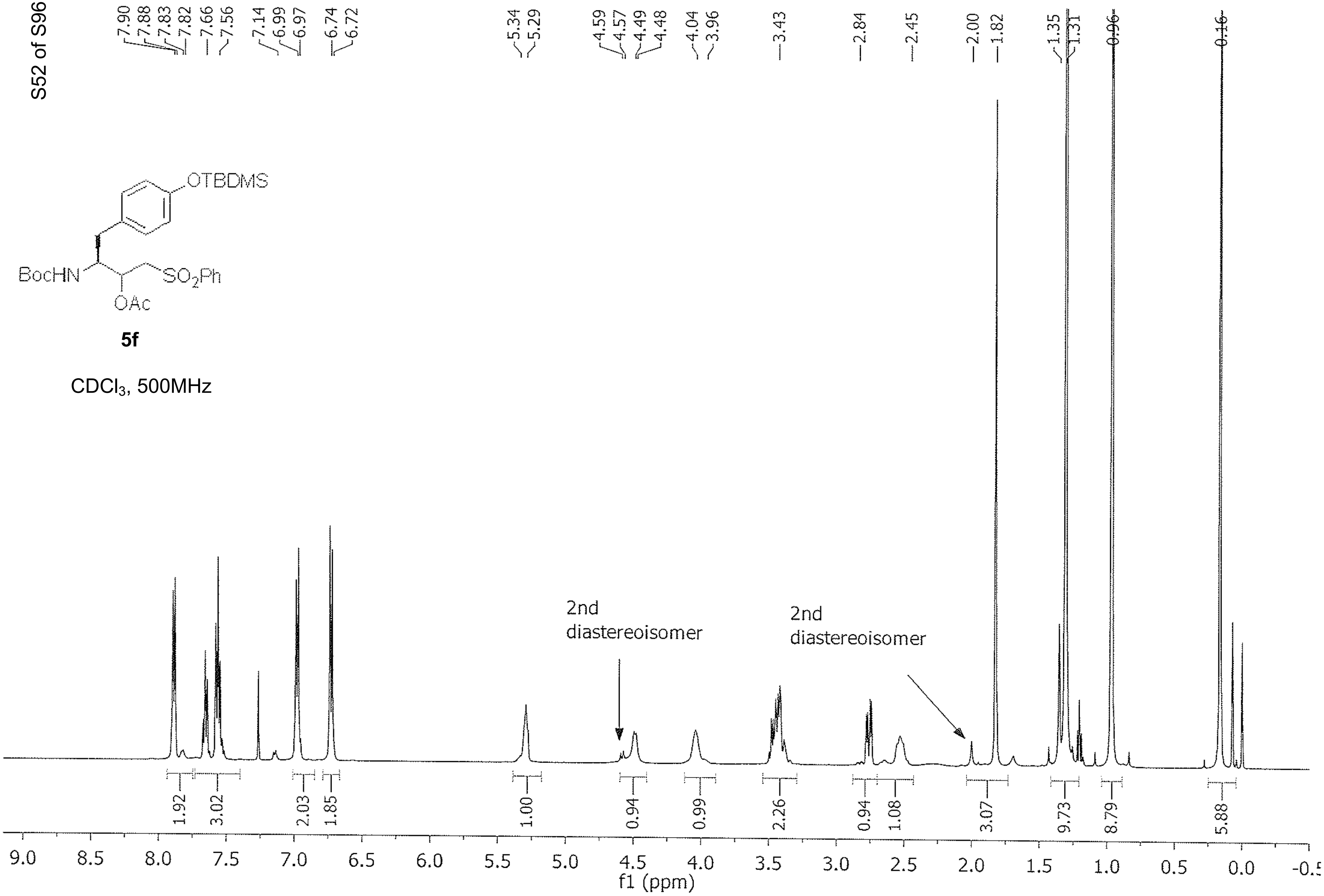

5f

$CDCl_3$, 500MHz

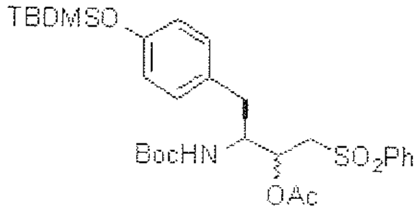

5f

$CDCl_3$, 125.68 MHz

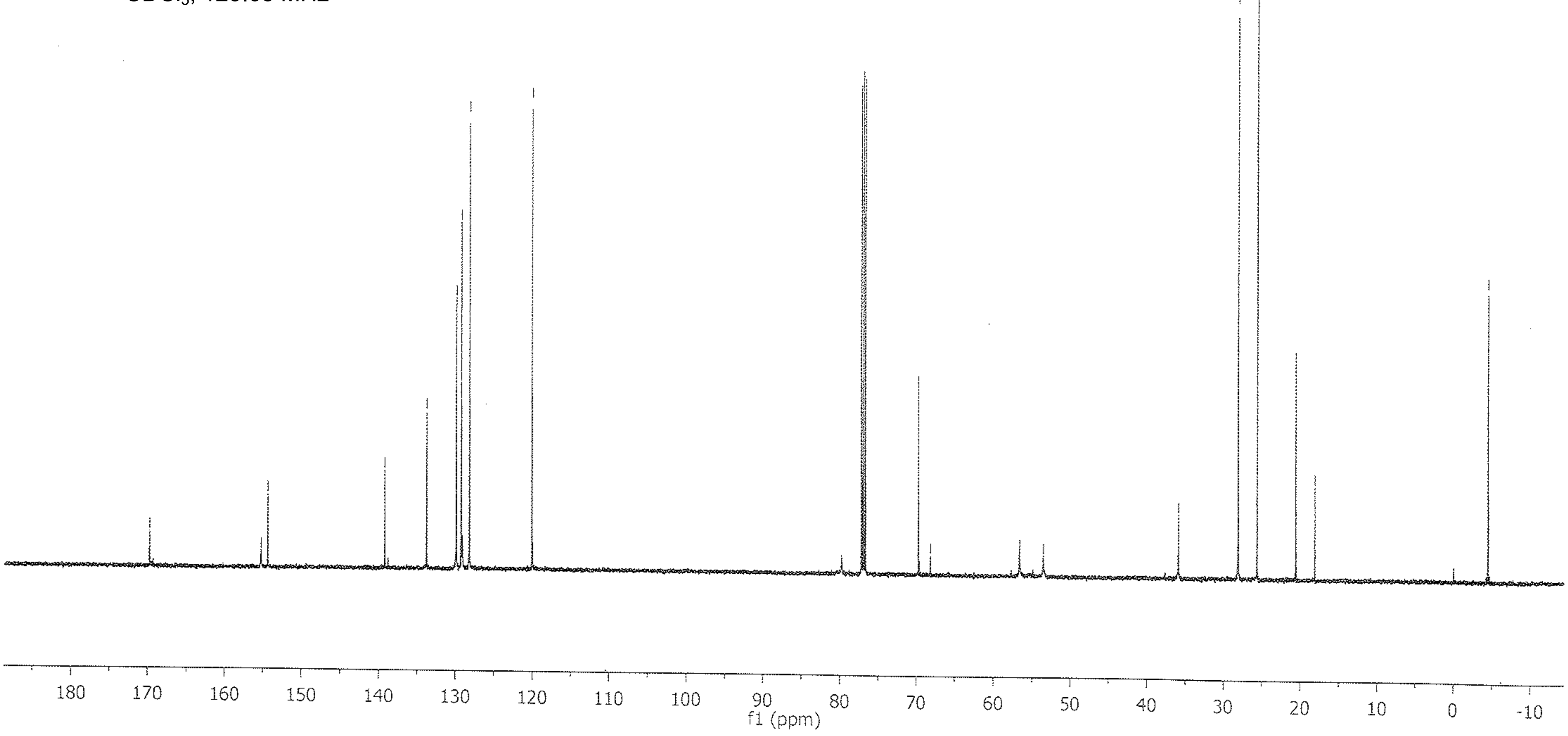

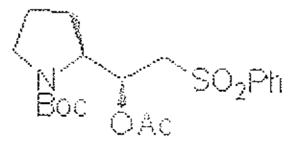

5g

dmso-d6
80°C

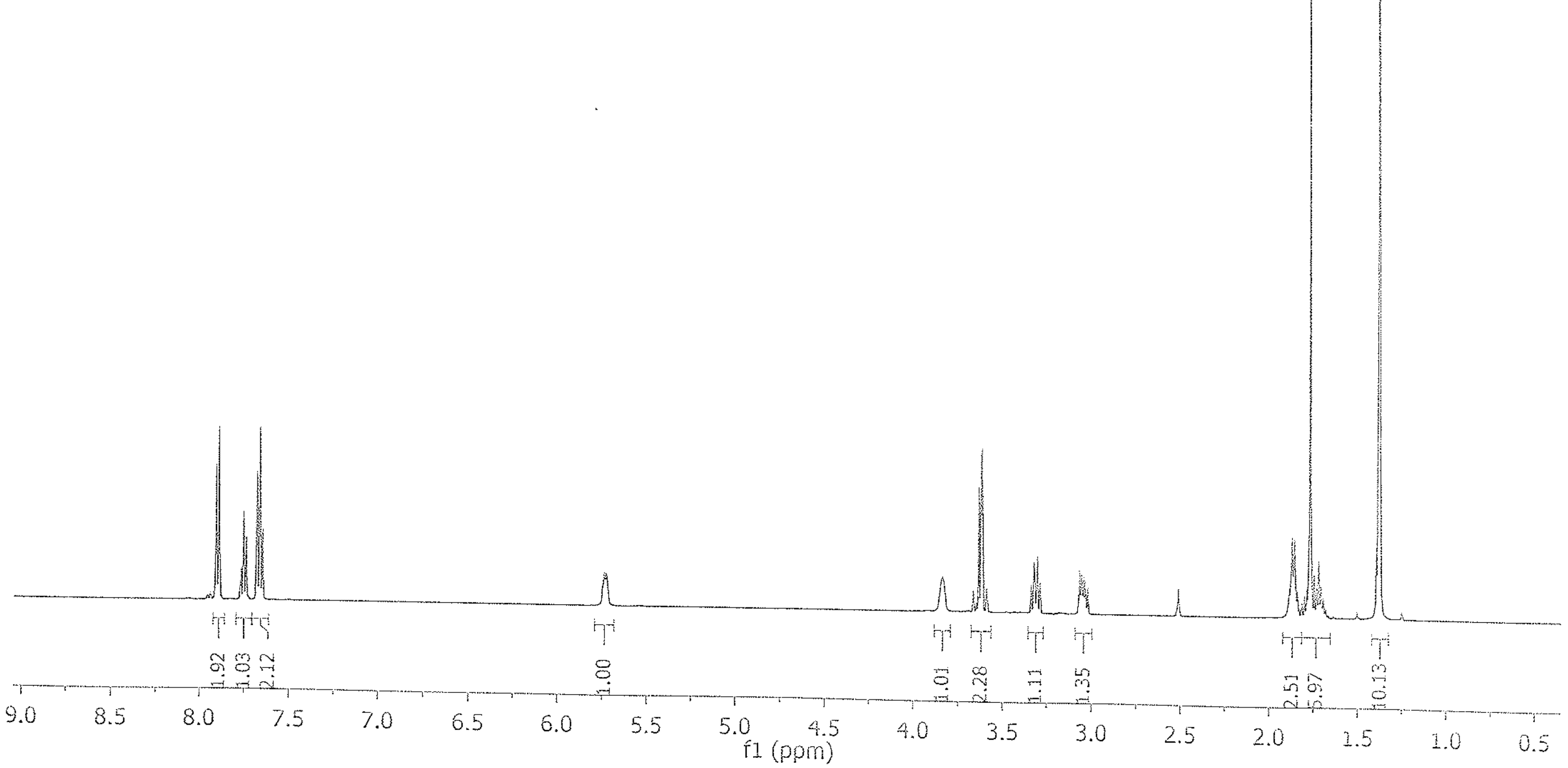

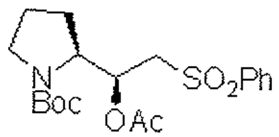

5g

dmso-d6
80°C

1 diastereoisomer

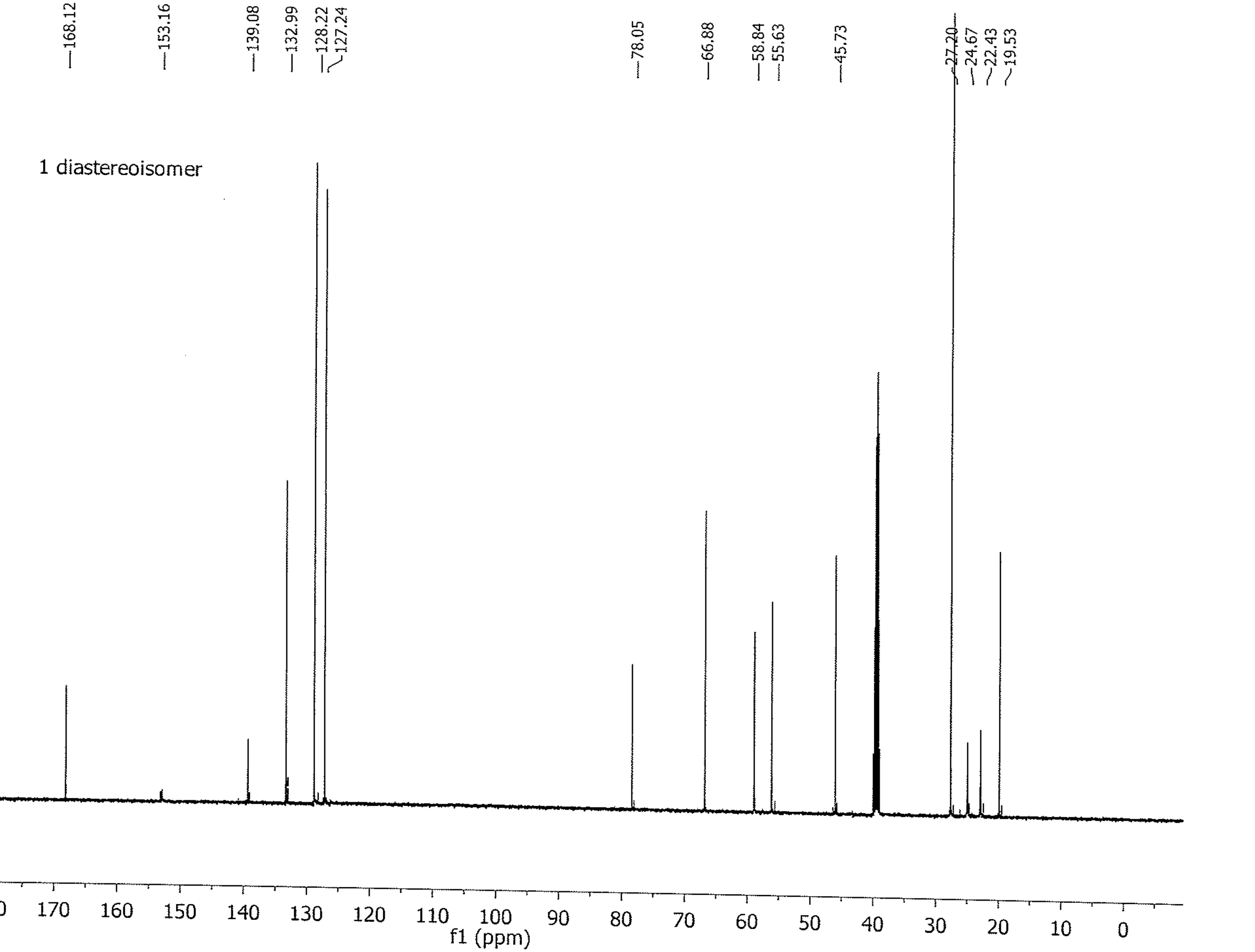

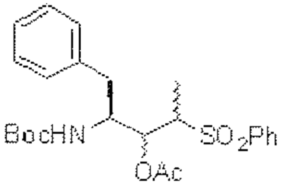

5h

$CDCl_3$, 500MHz

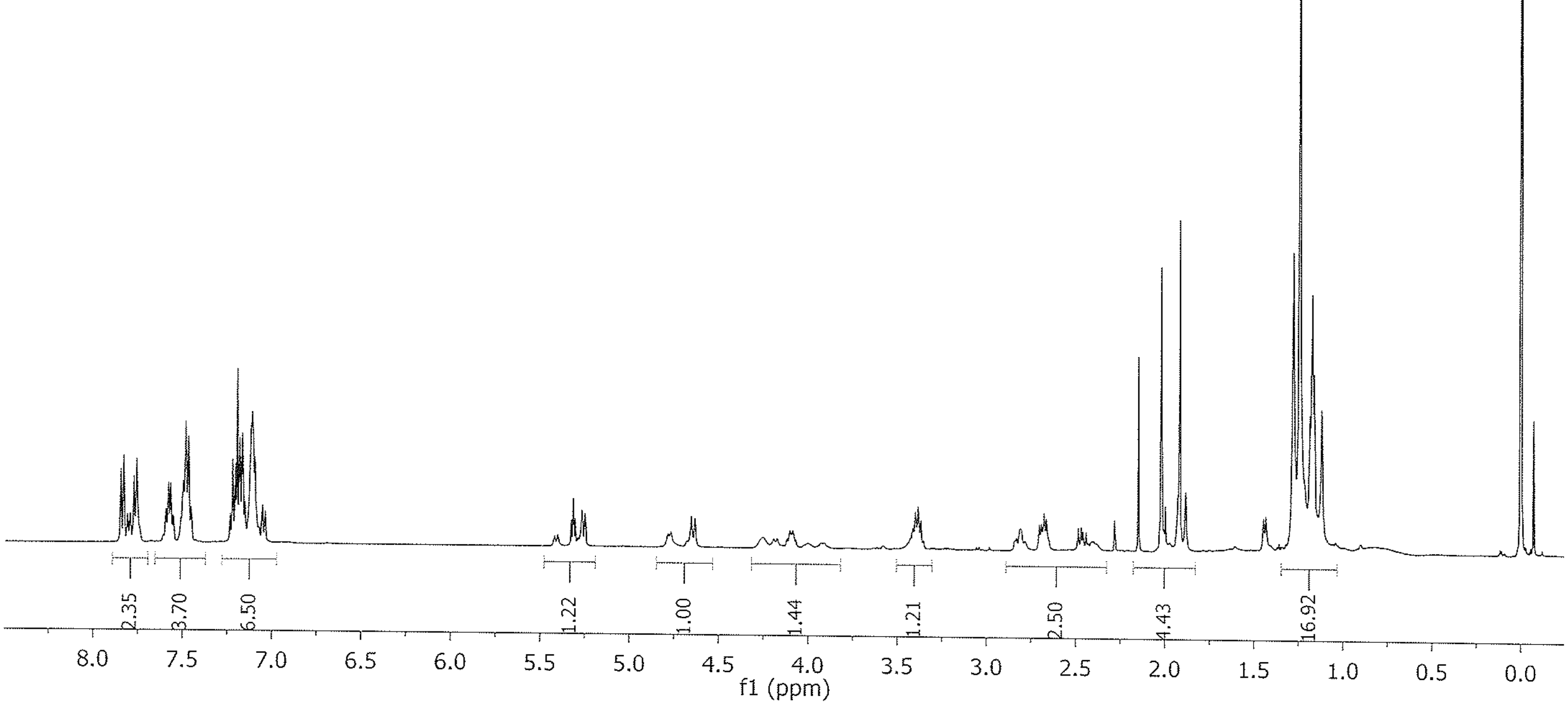

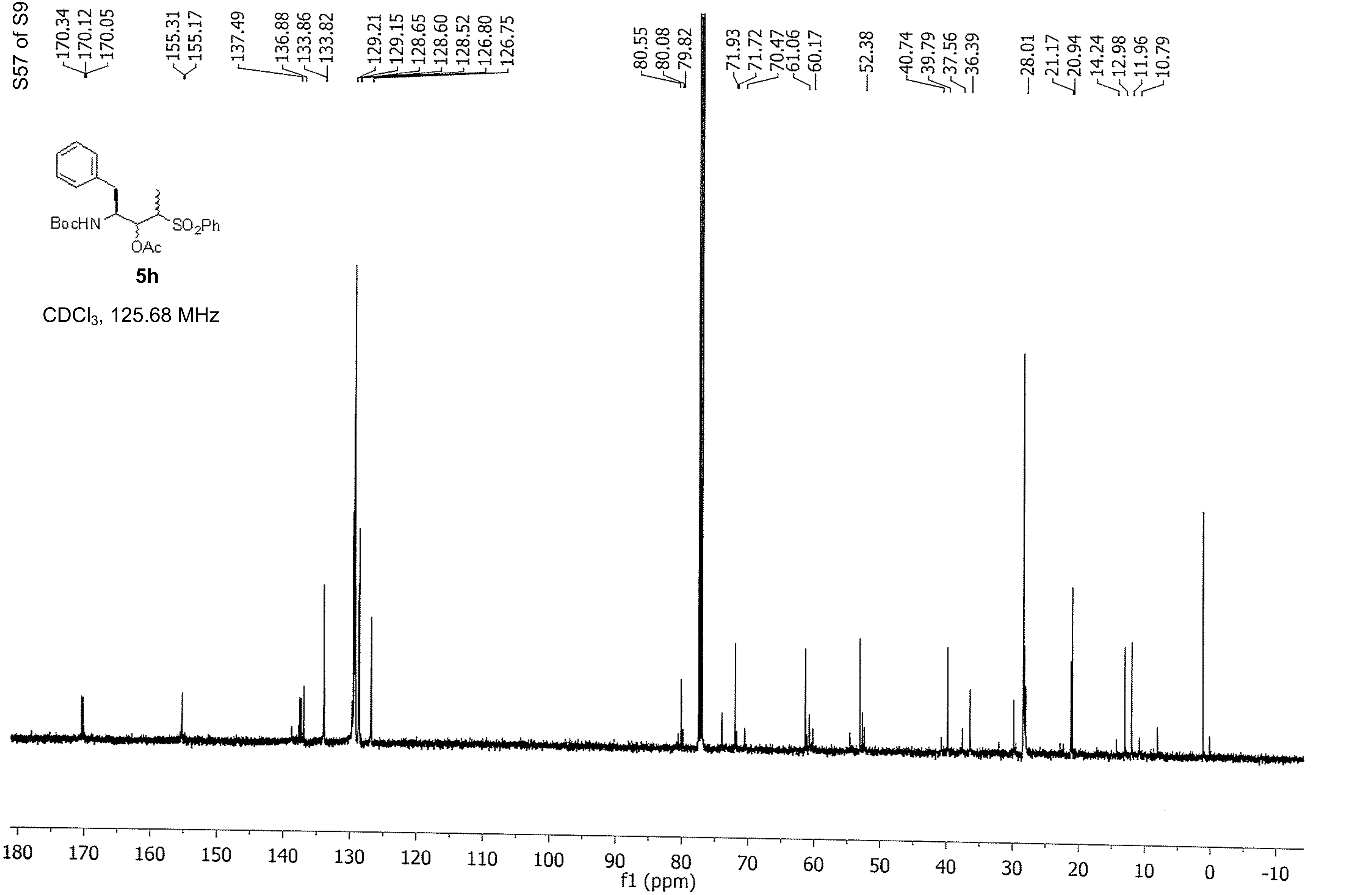

5h

$CDCl_3$, 125.68 MHz

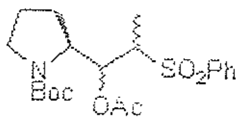

5i

$CDCl_3$, 500MHz

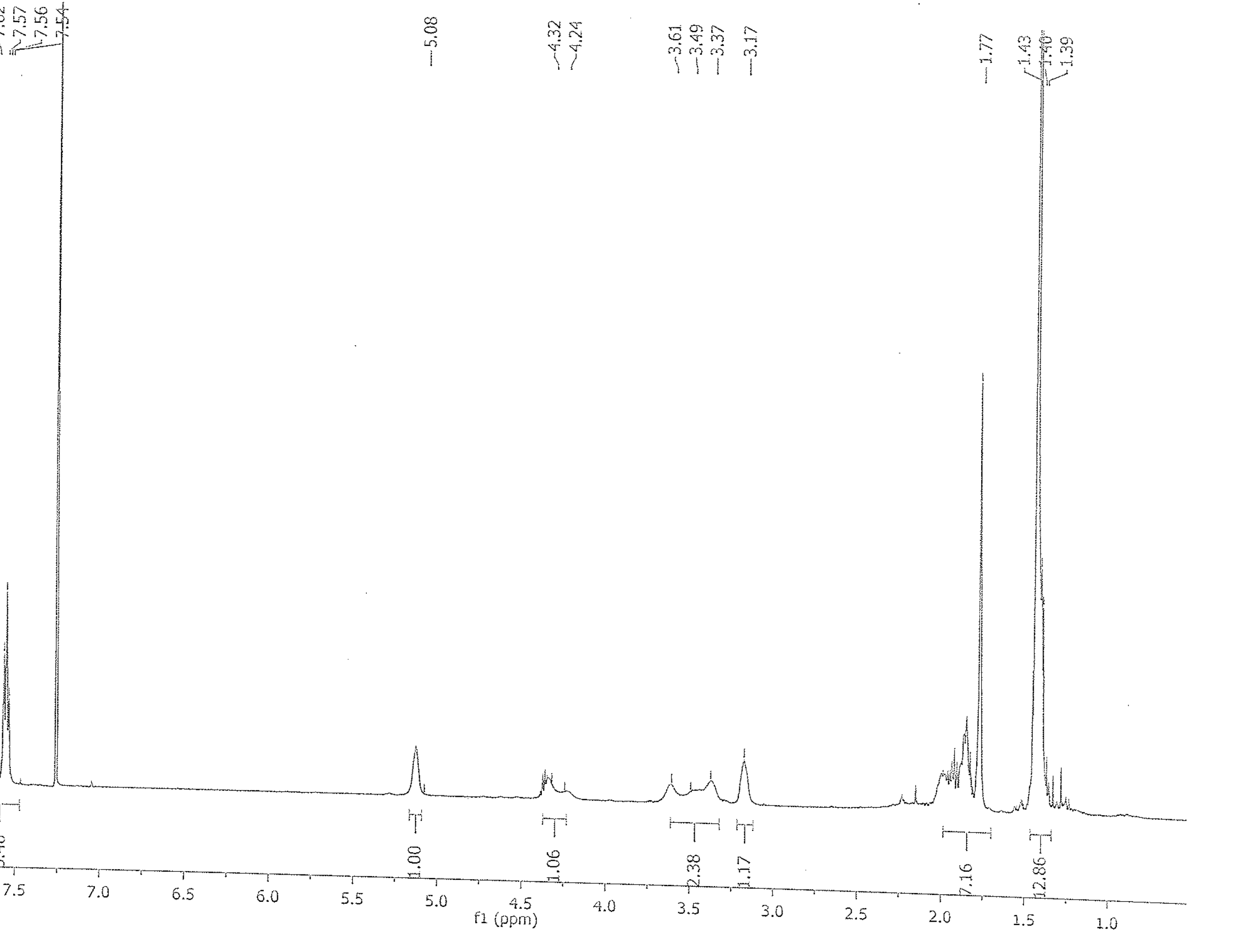

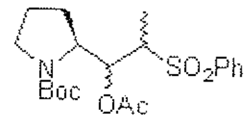

5i

$CDCl_3$, 125.68 MHz

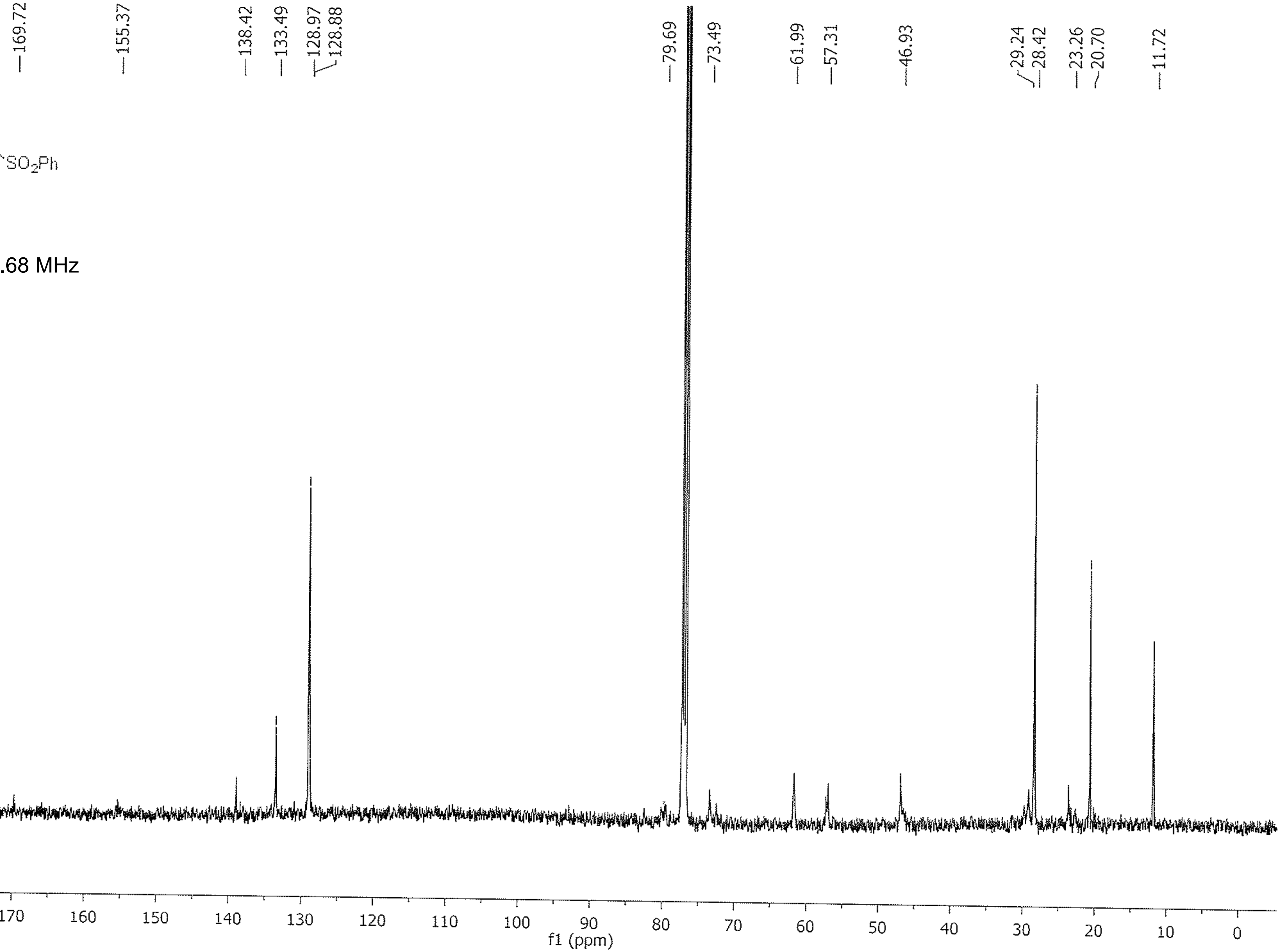

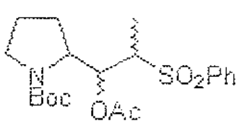
N
Boc
OAc
SO2Ph

5i

$CDCl_3$

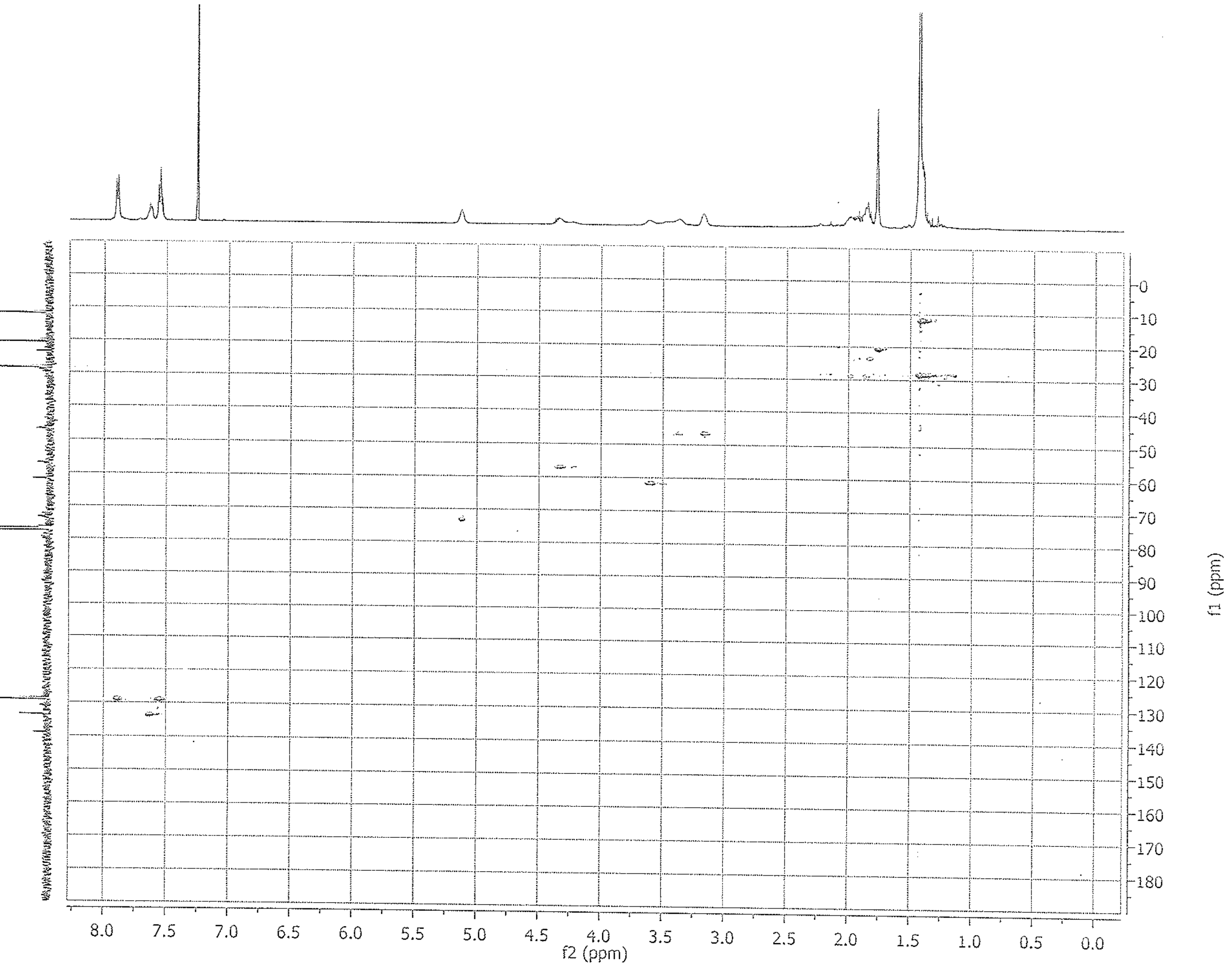
8.0
7.5
7.0
6.5
6.0
5.5
5.0
4.5
4.0
3.5
3.0
2.5
2.0
1.5
1.0
0.5
0.0
f2 (ppm)
0
10
20
30
40
50
60
70
80
90
100
110
120
130
140
150
160
170
180
f1 (ppm)

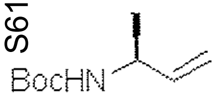

6a

$CDCl_3$, 500MHz

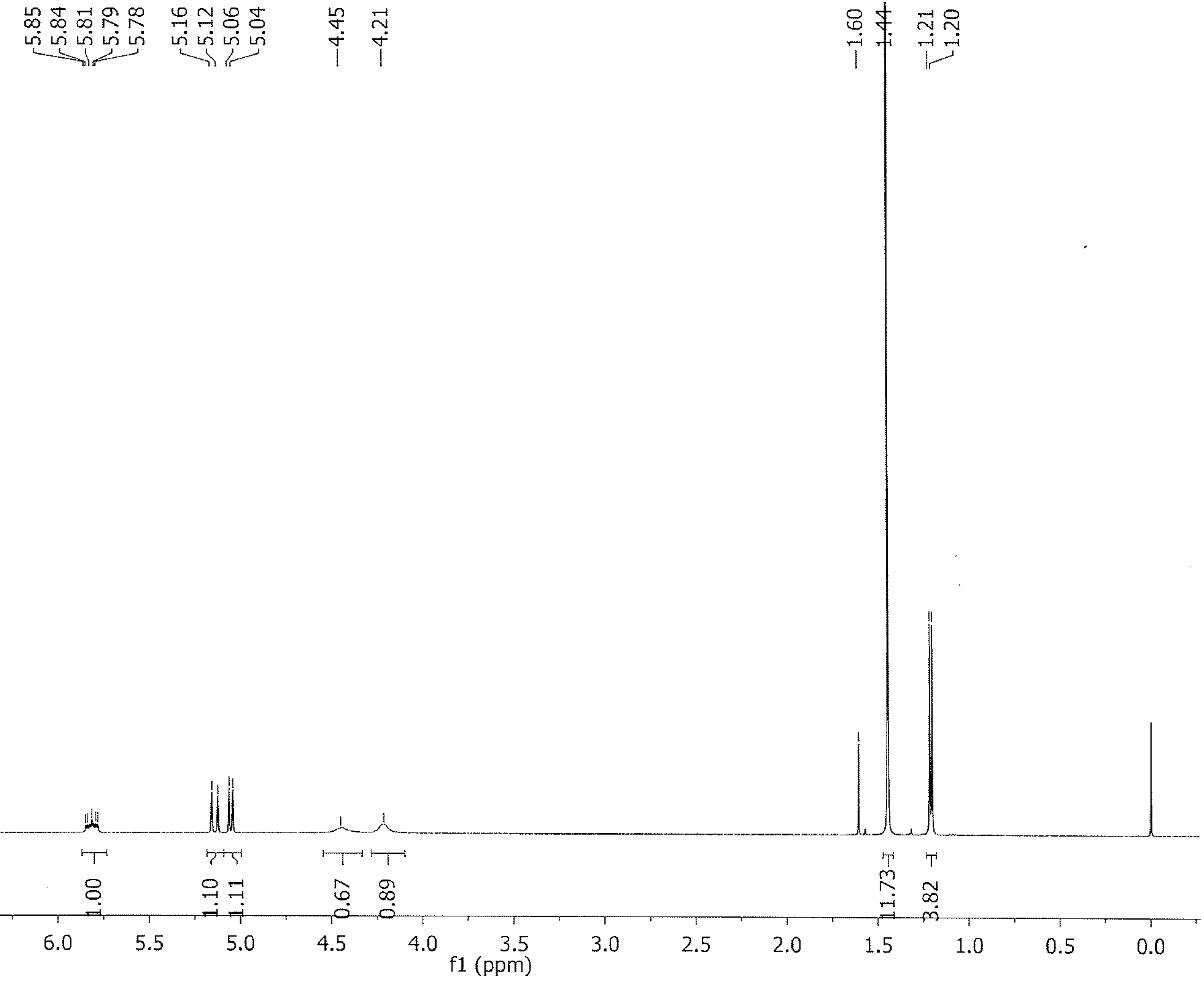

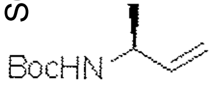

6a

$CDCl_3$, 125.68 MHz

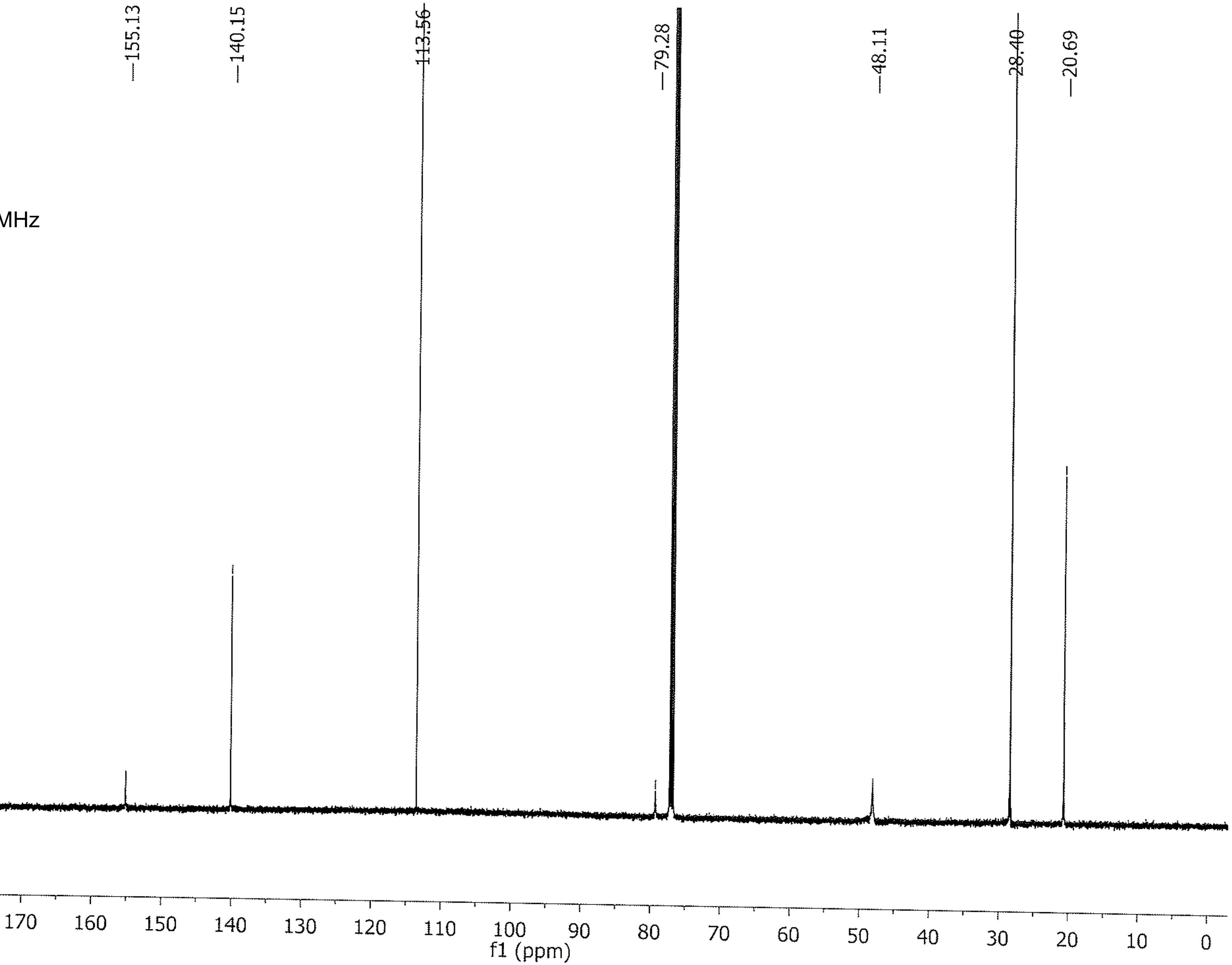

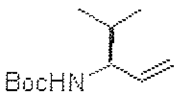

6b

$CDCl_3$, 500MHz

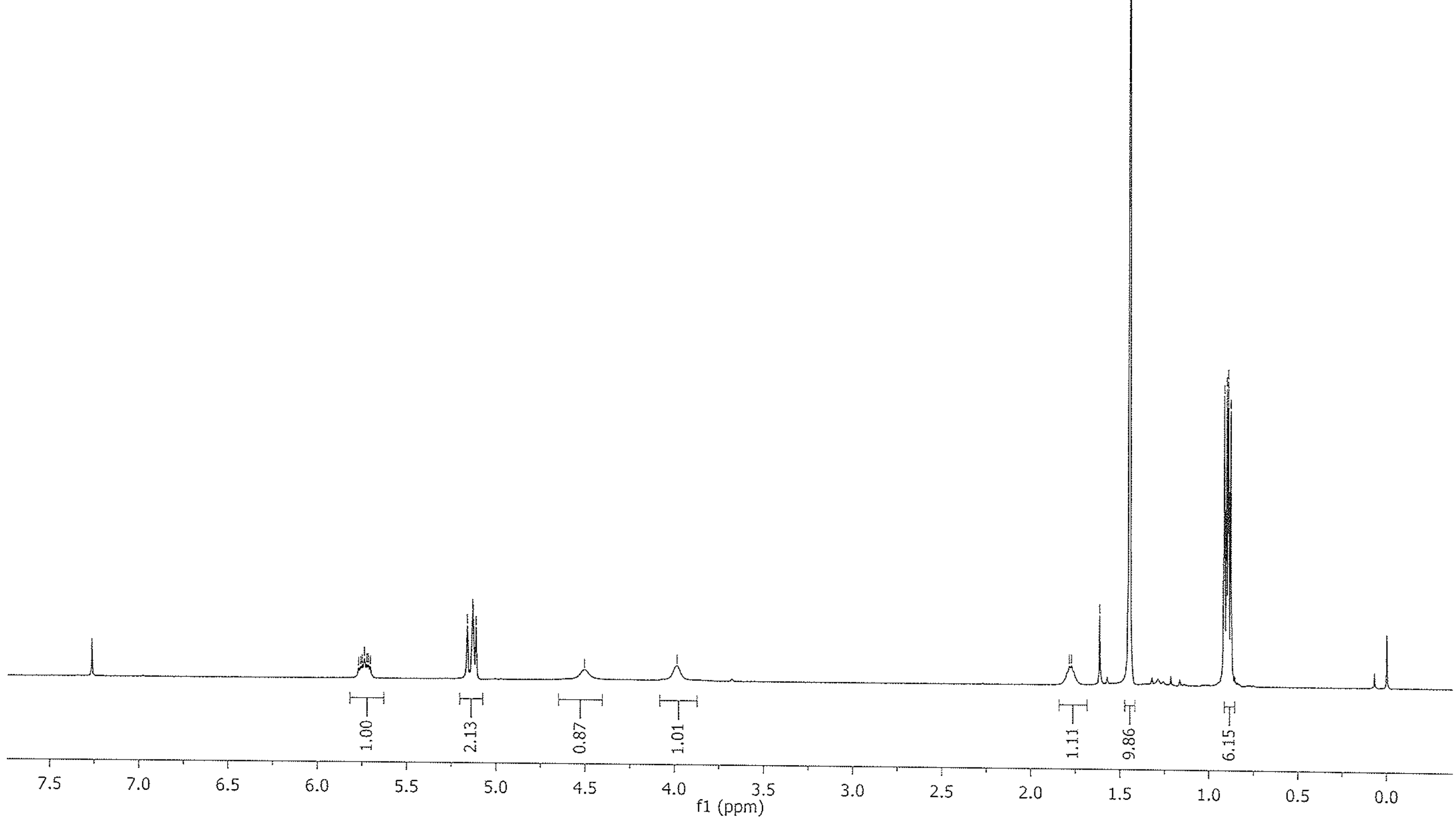

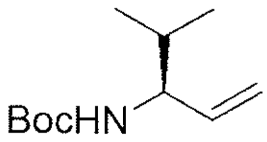

6b

$CDCl_3$, 125.68 MHz

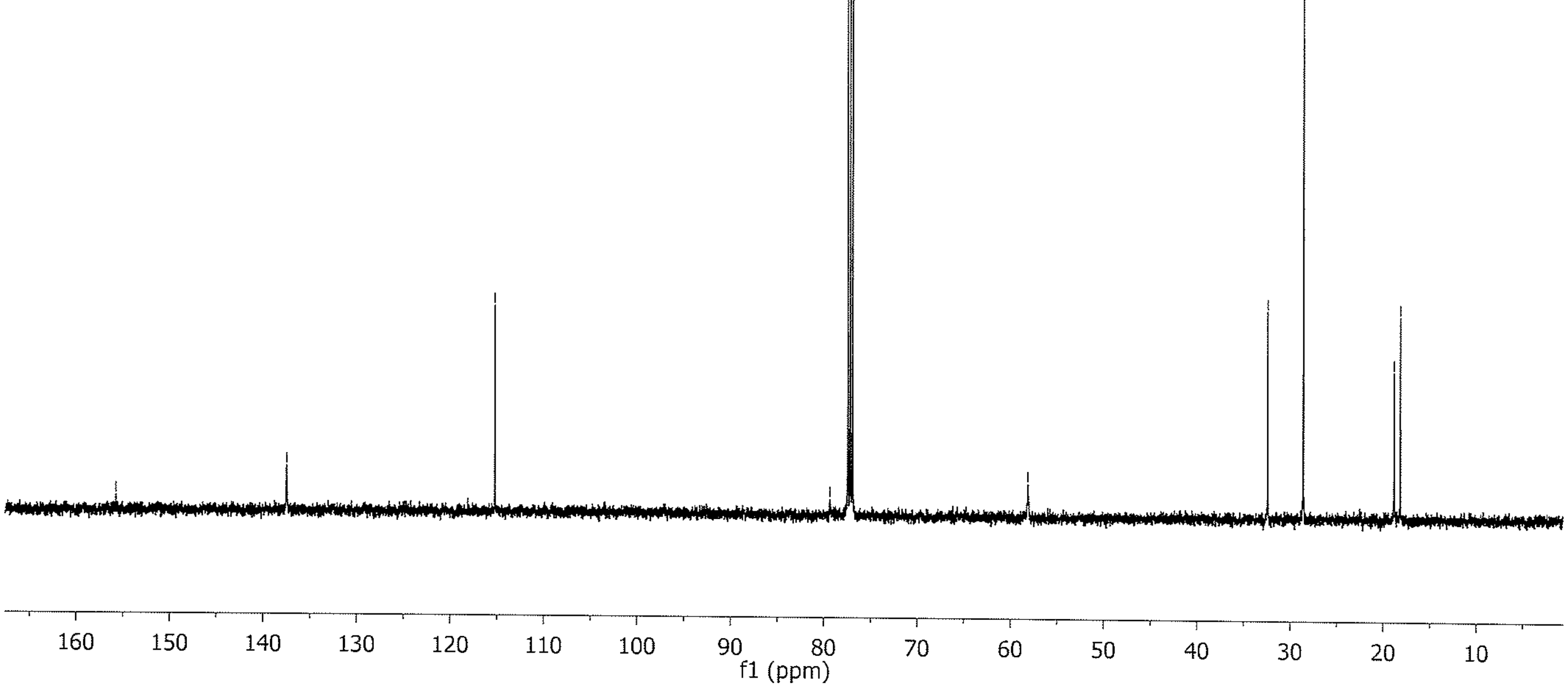

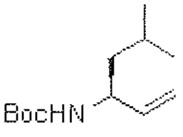

6c

$CDCl_3$, 500MHz

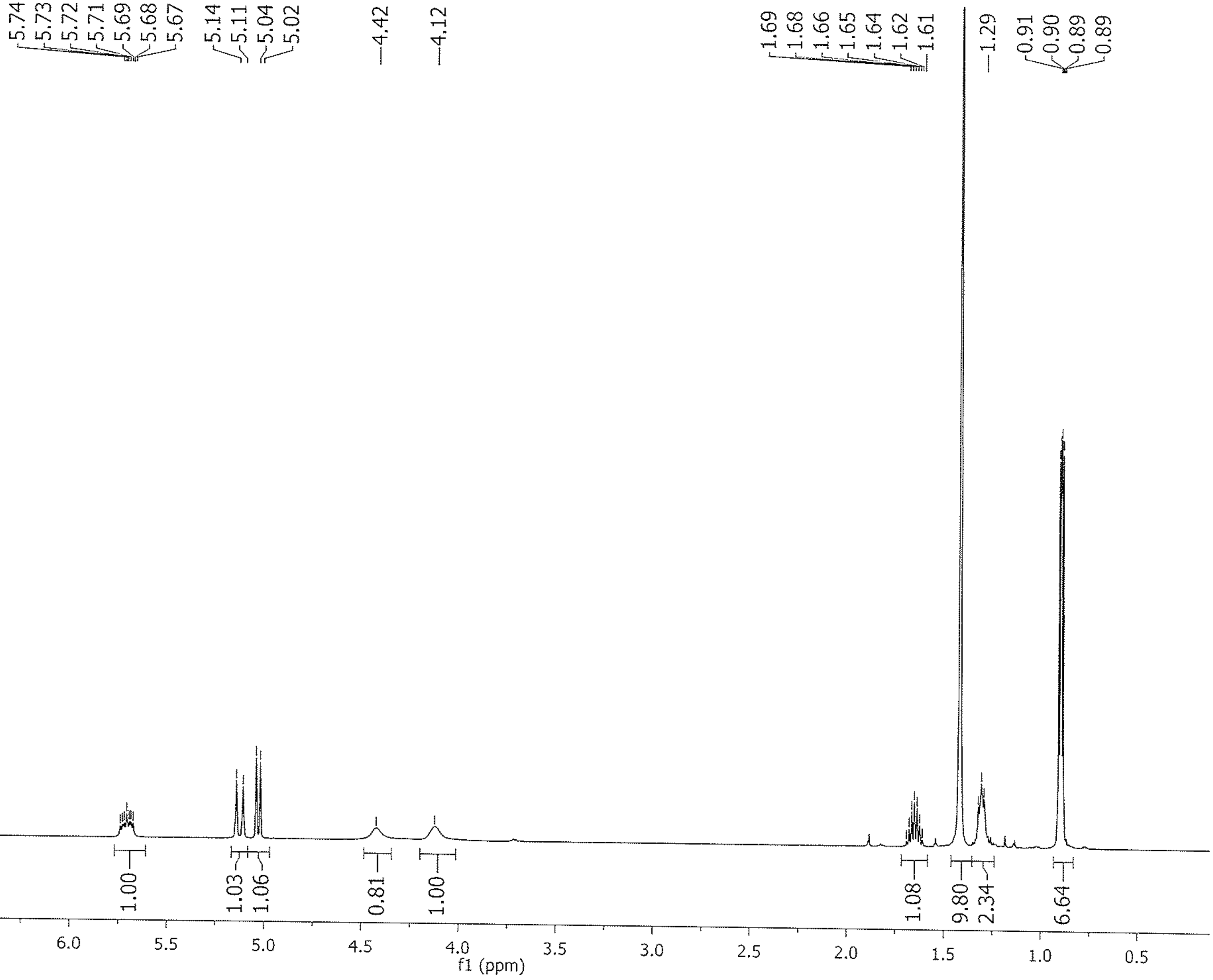

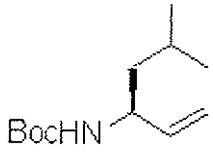

6c

CDCl$_3$, 125.68 MHz

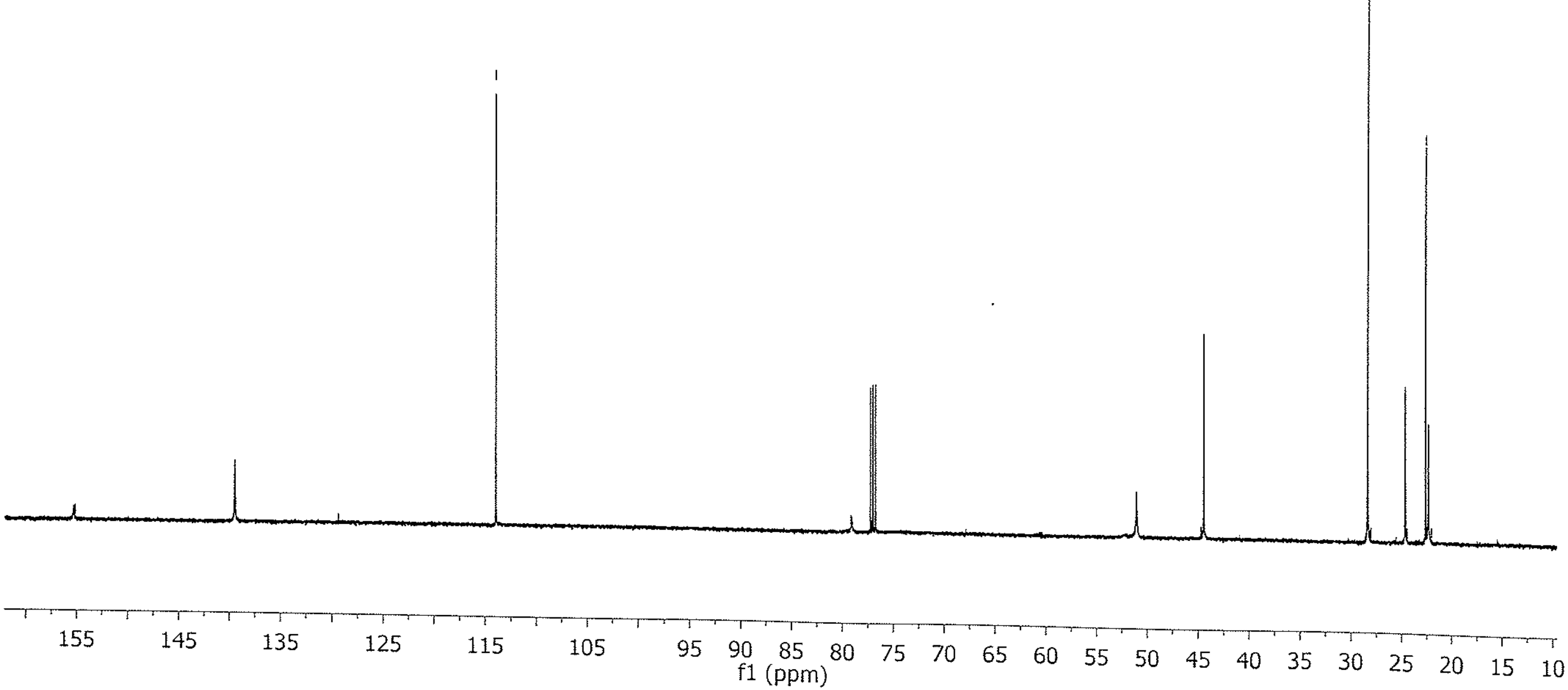

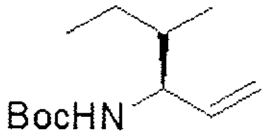

6d

$CDCl_3$, 500MHz

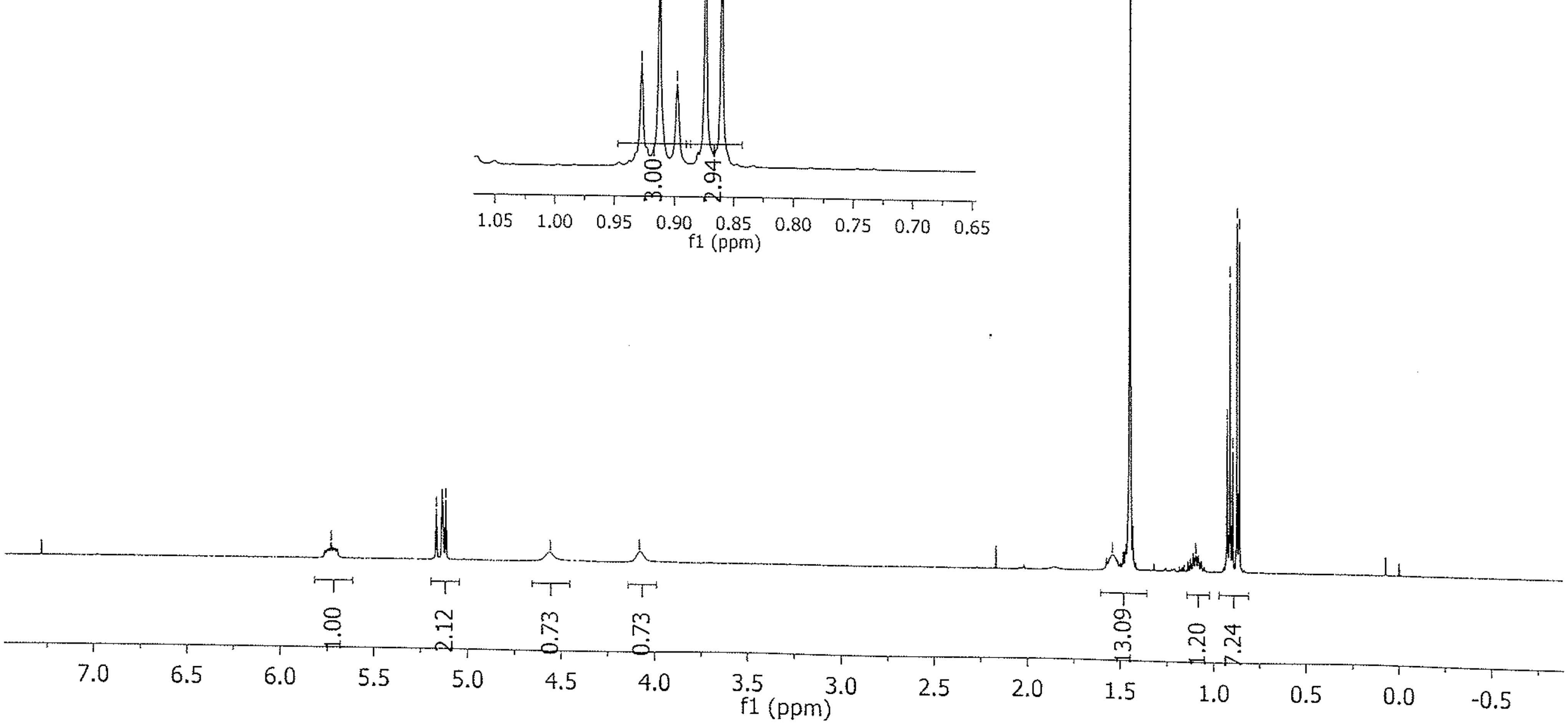

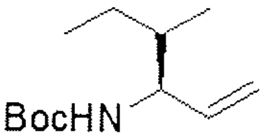

6d

$CDCl_3$, 125.68 MHz

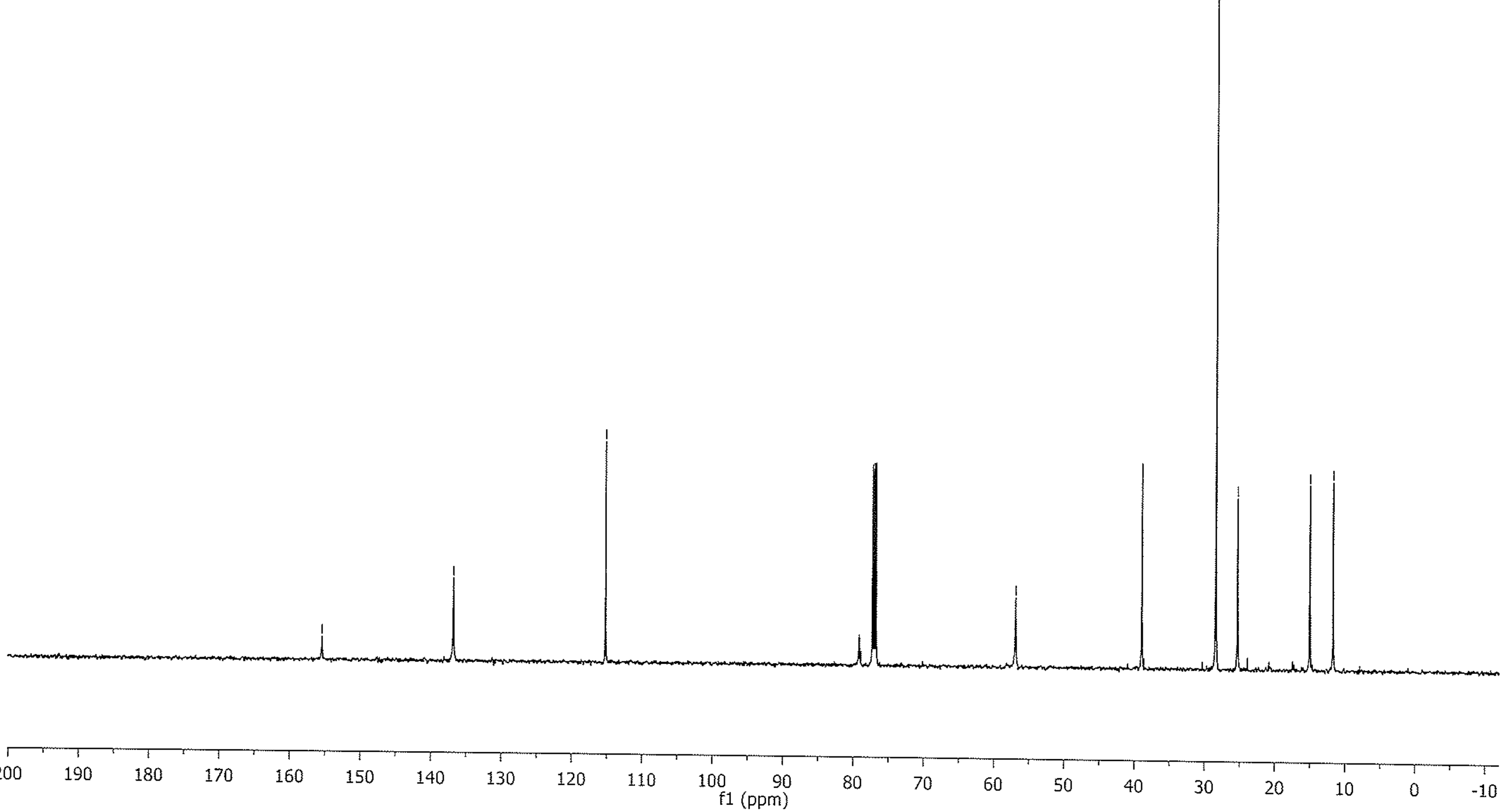

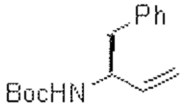

6e

$CDCl_3$, 500MHz

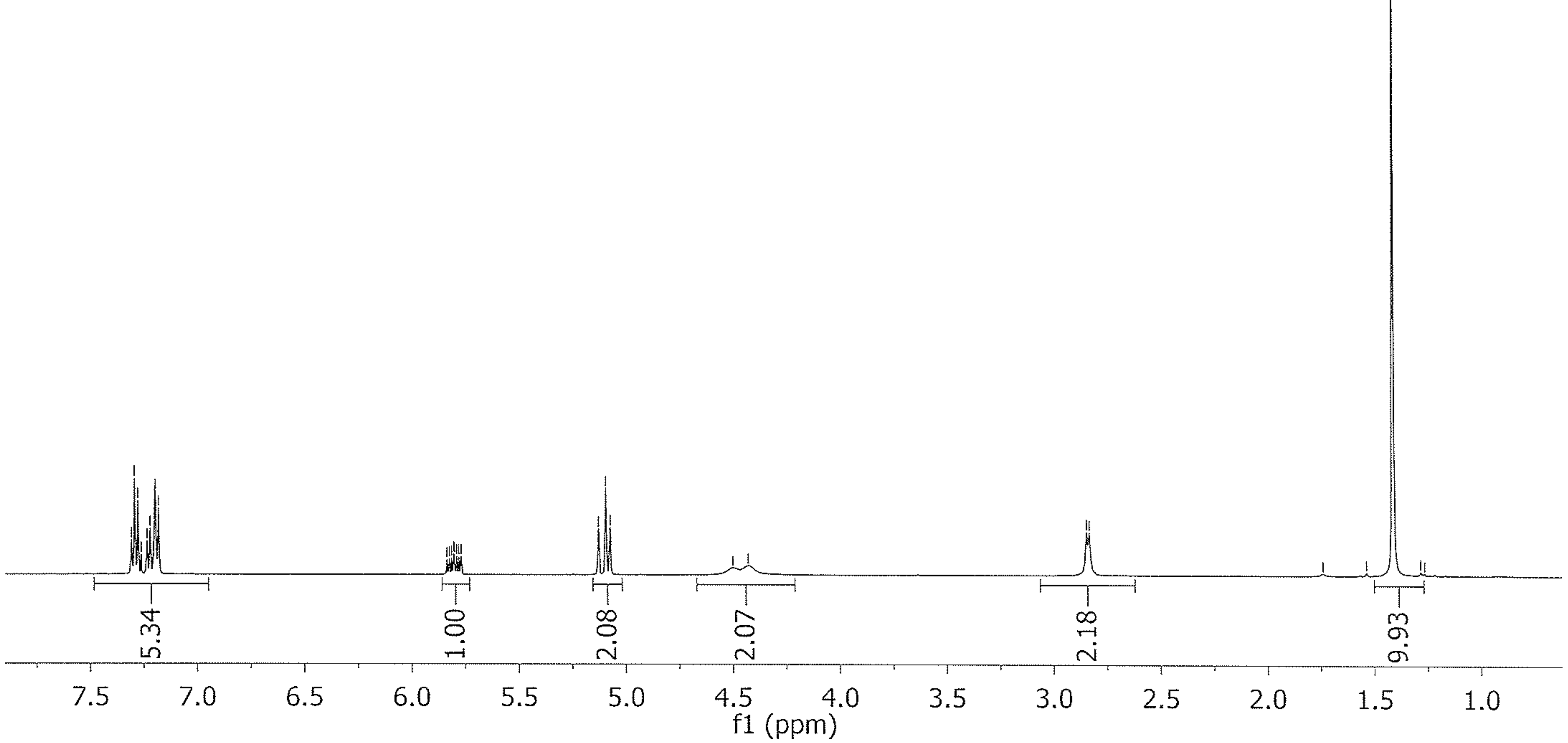

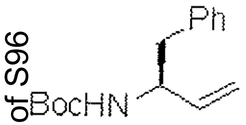

6e

CDCl$_3$, 125.68 MHz

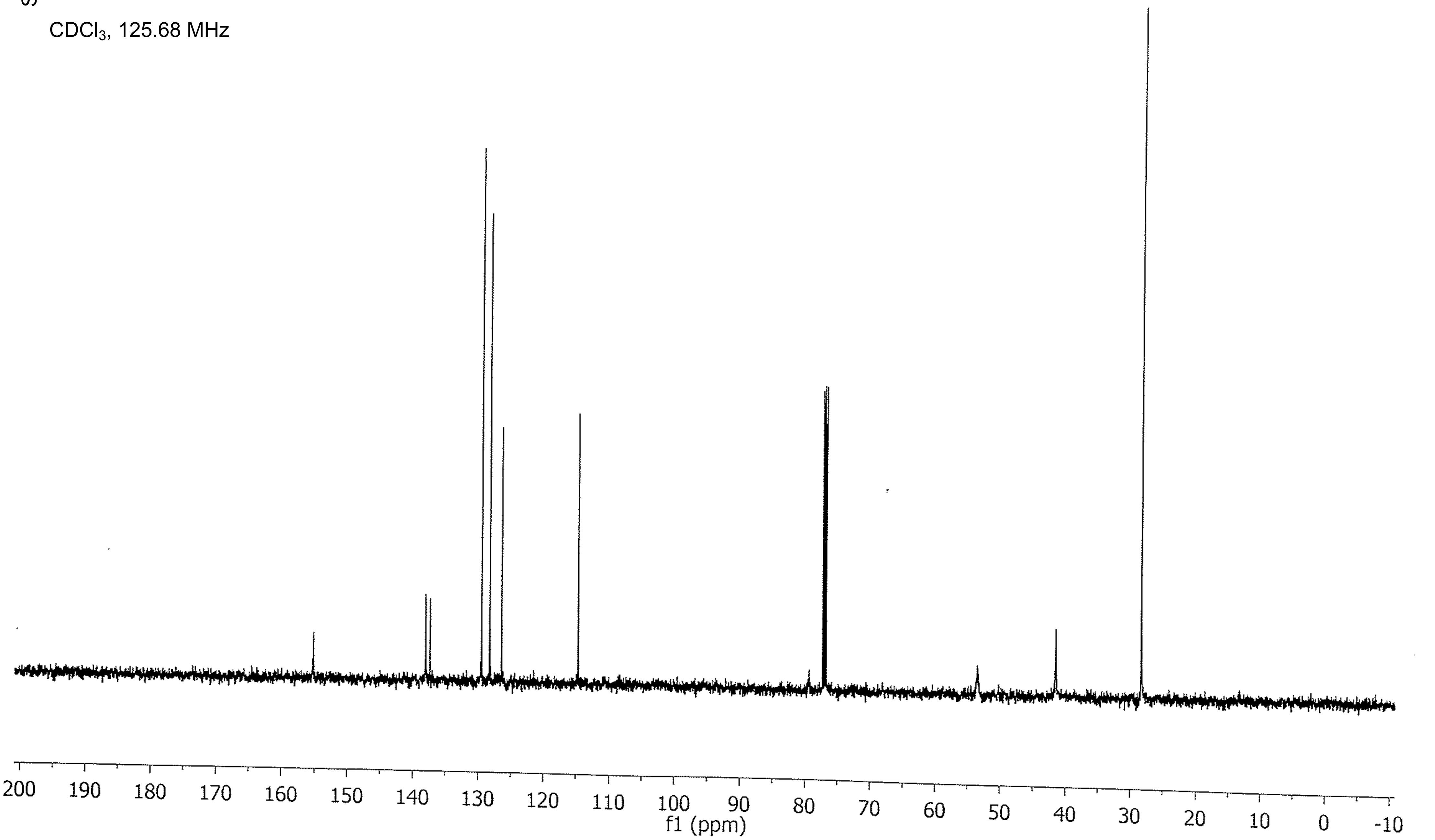

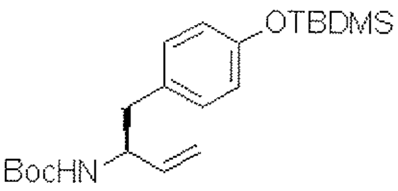

6f

$CDCl_3$, 500MHz

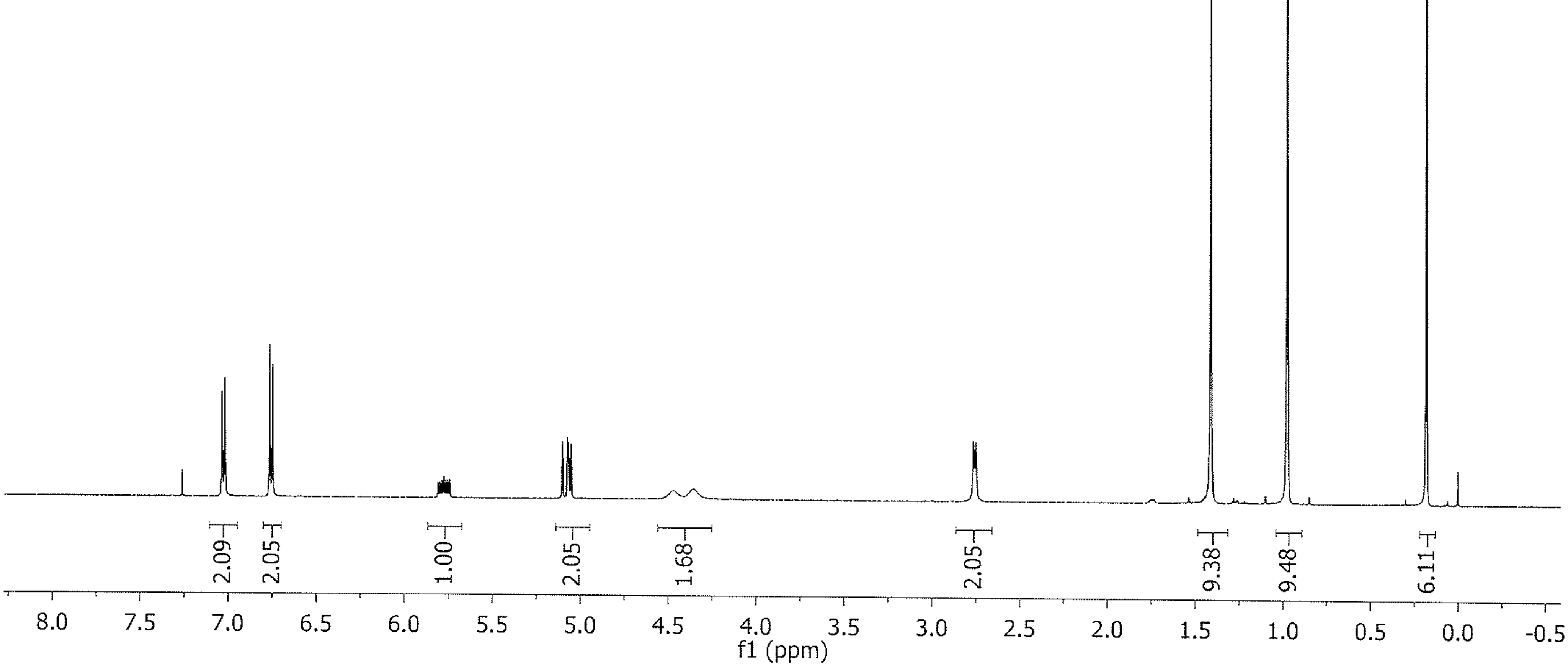

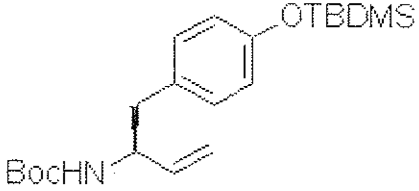

6f

$CDCl_3$, 125.68 MHz

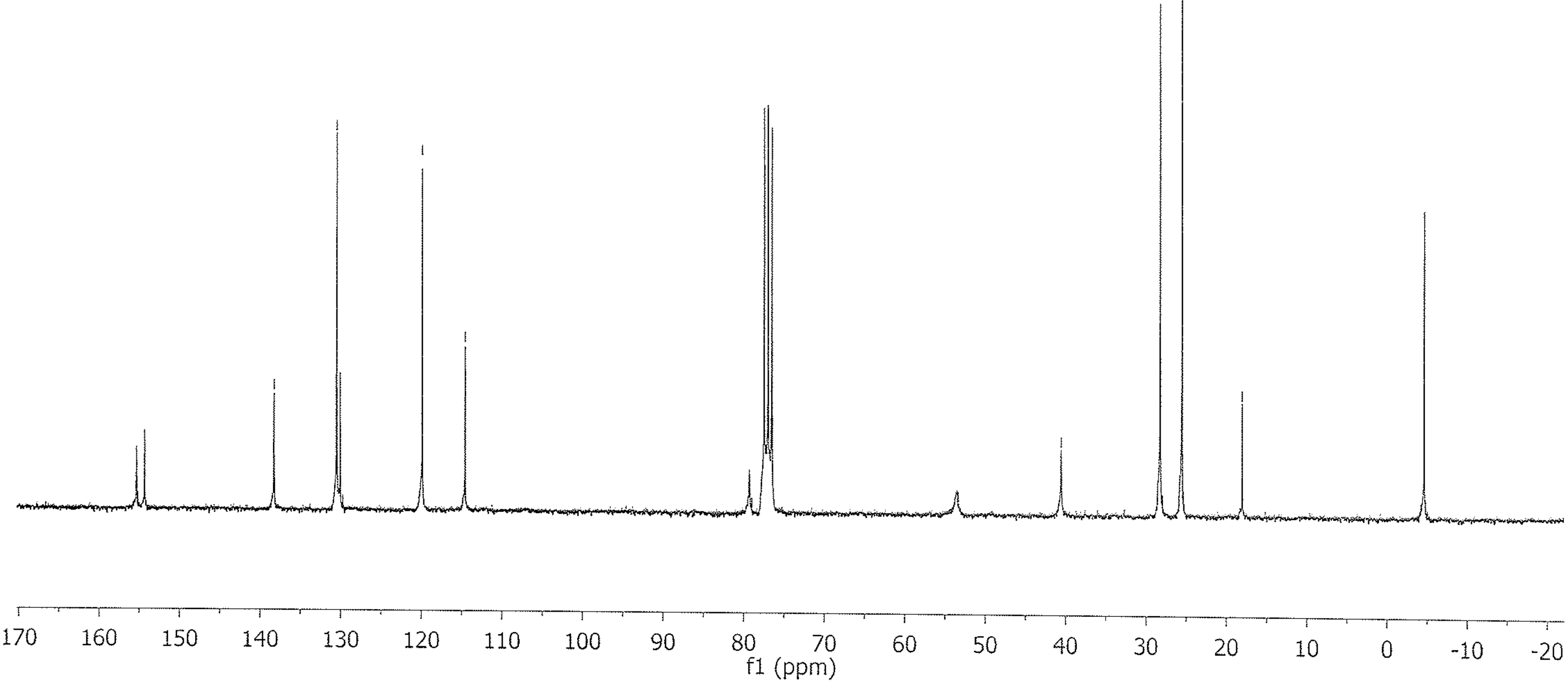

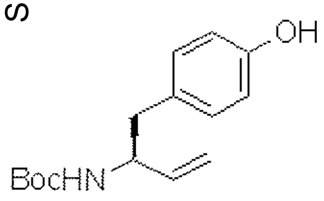

6f'

$CDCl_3$, 500MHz

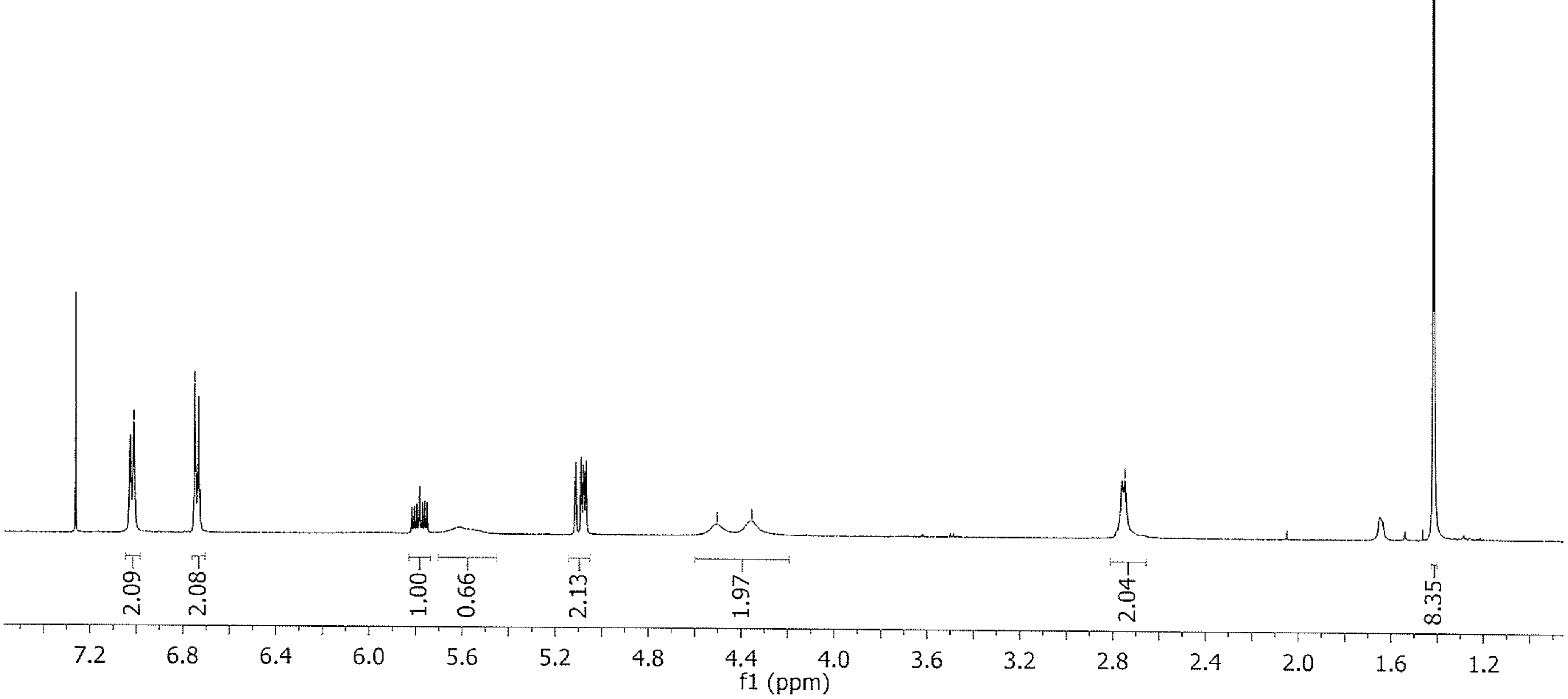

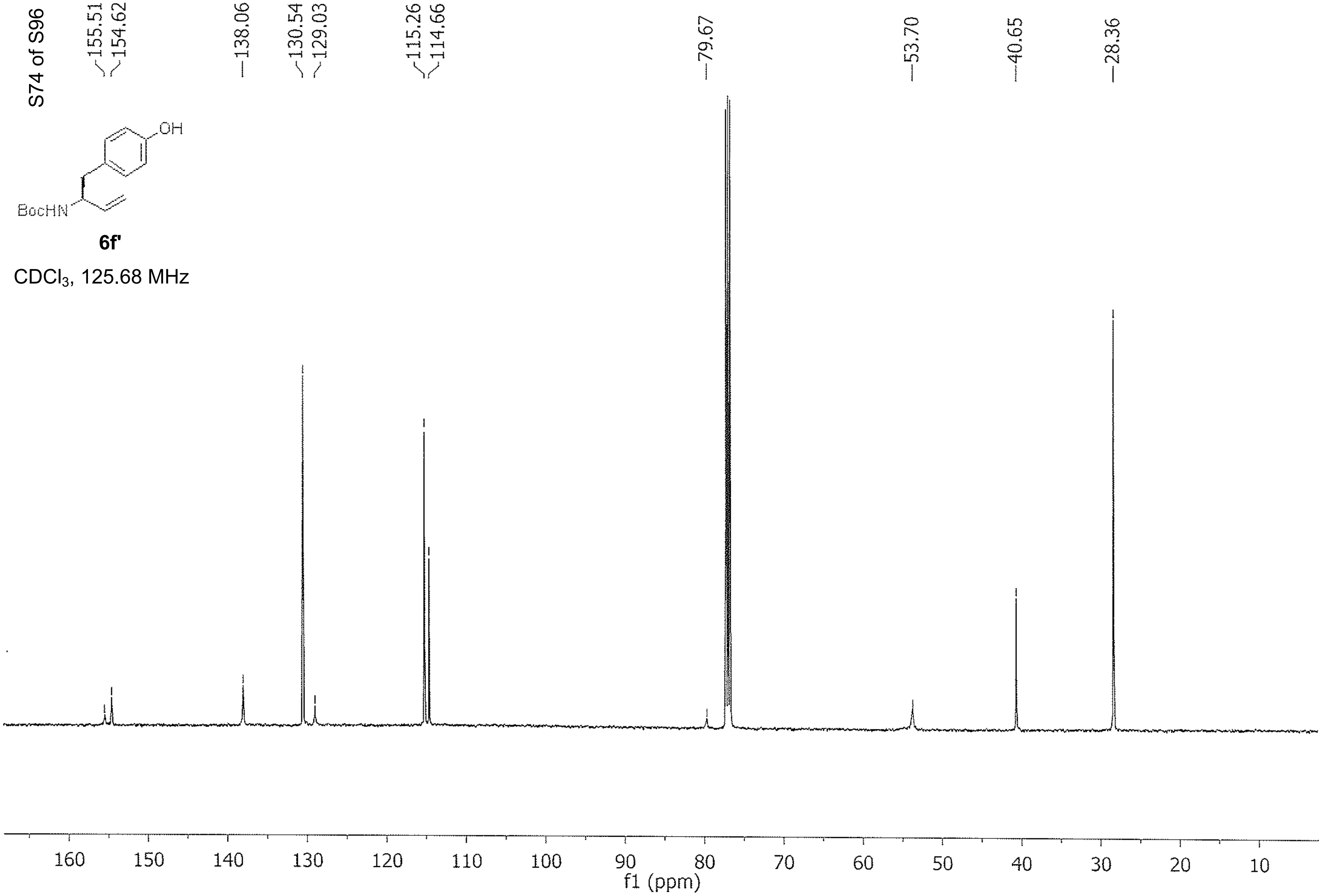

6f'

$CDCl_3$, 125.68 MHz

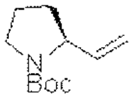

6g

$CDCl_3$, 500MHz

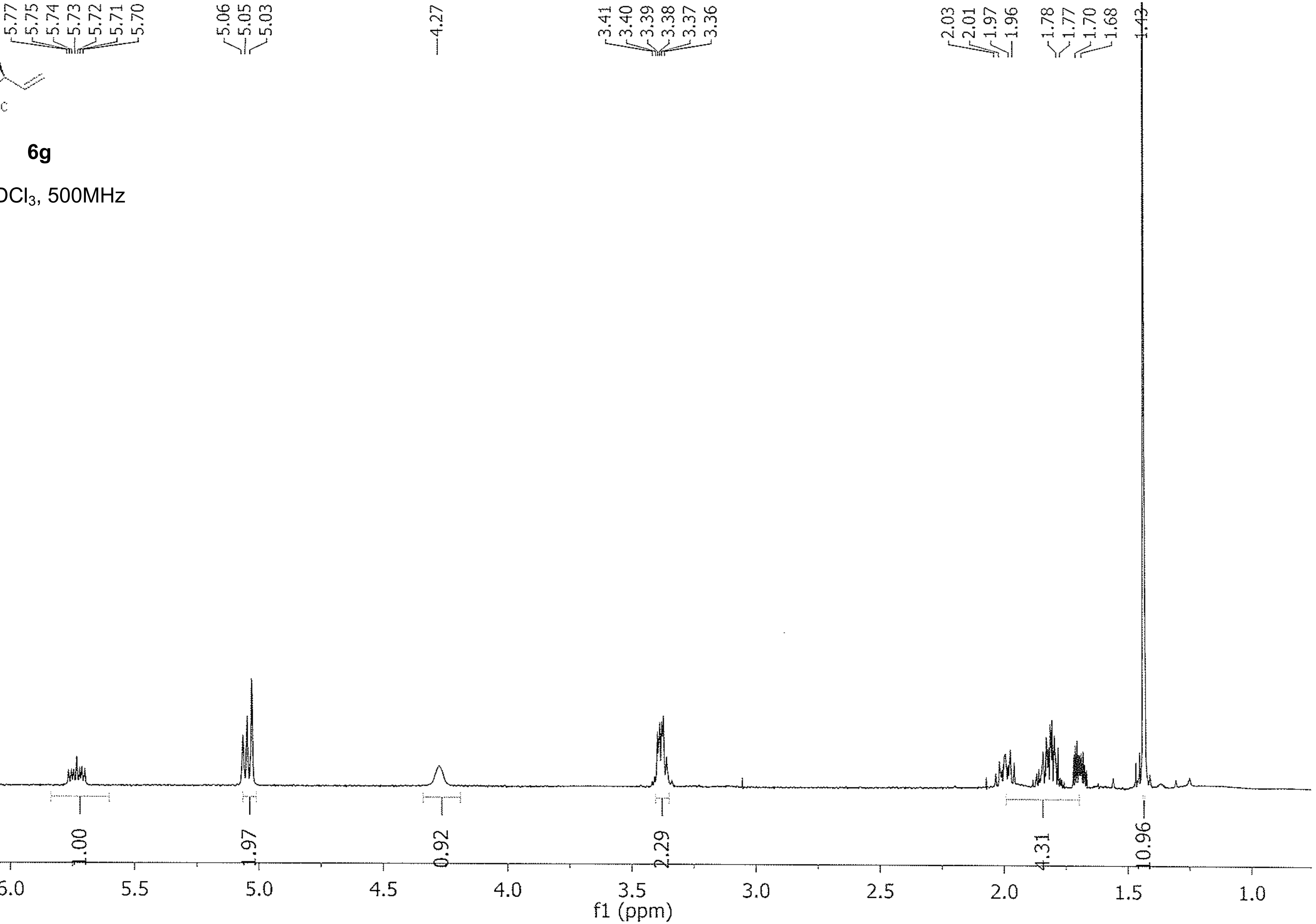

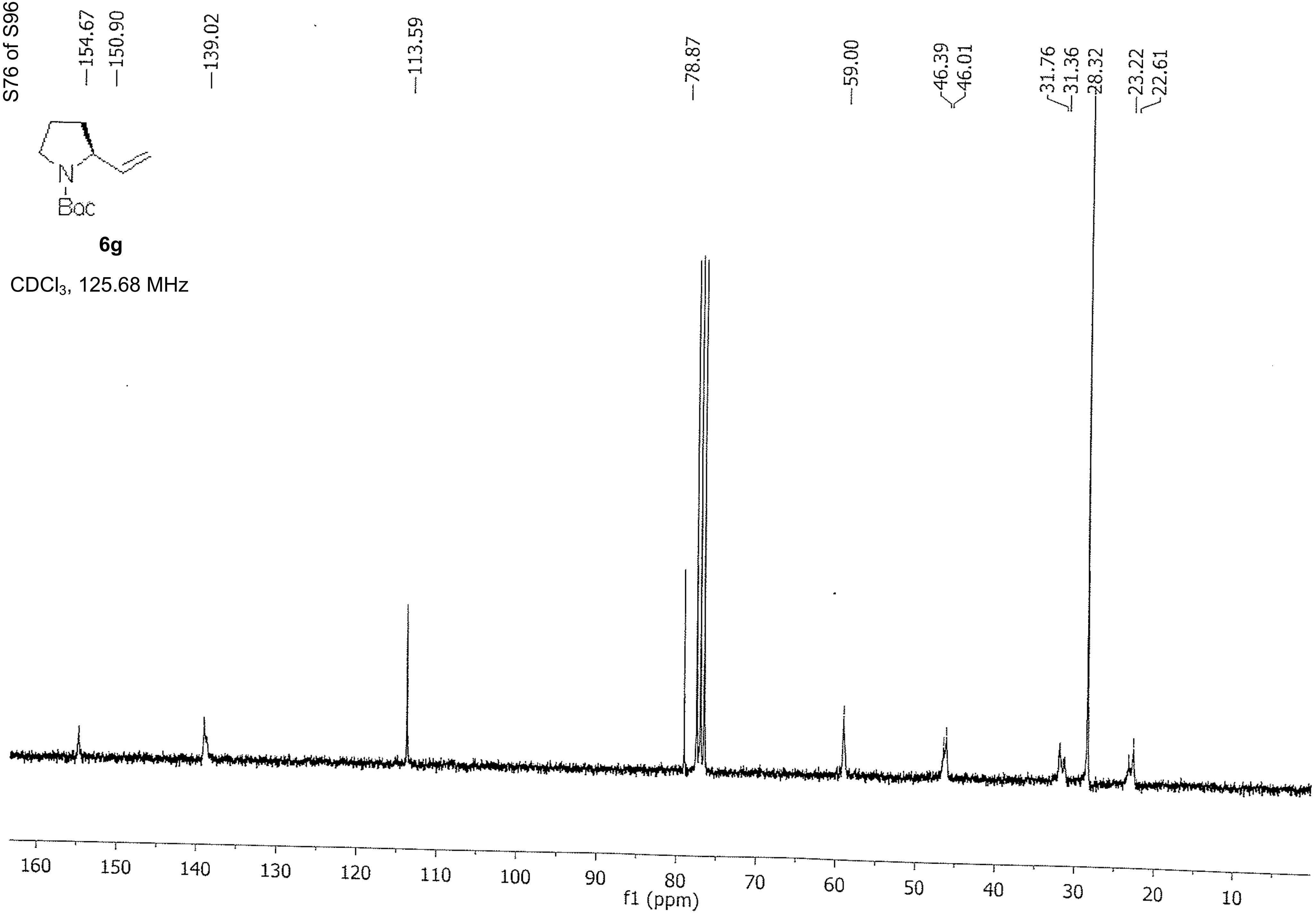

6g

$CDCl_3$, 125.68 MHz

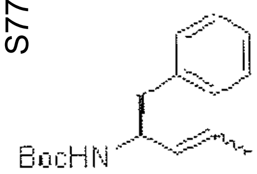

6h

$CDCl_3$, 500MHz

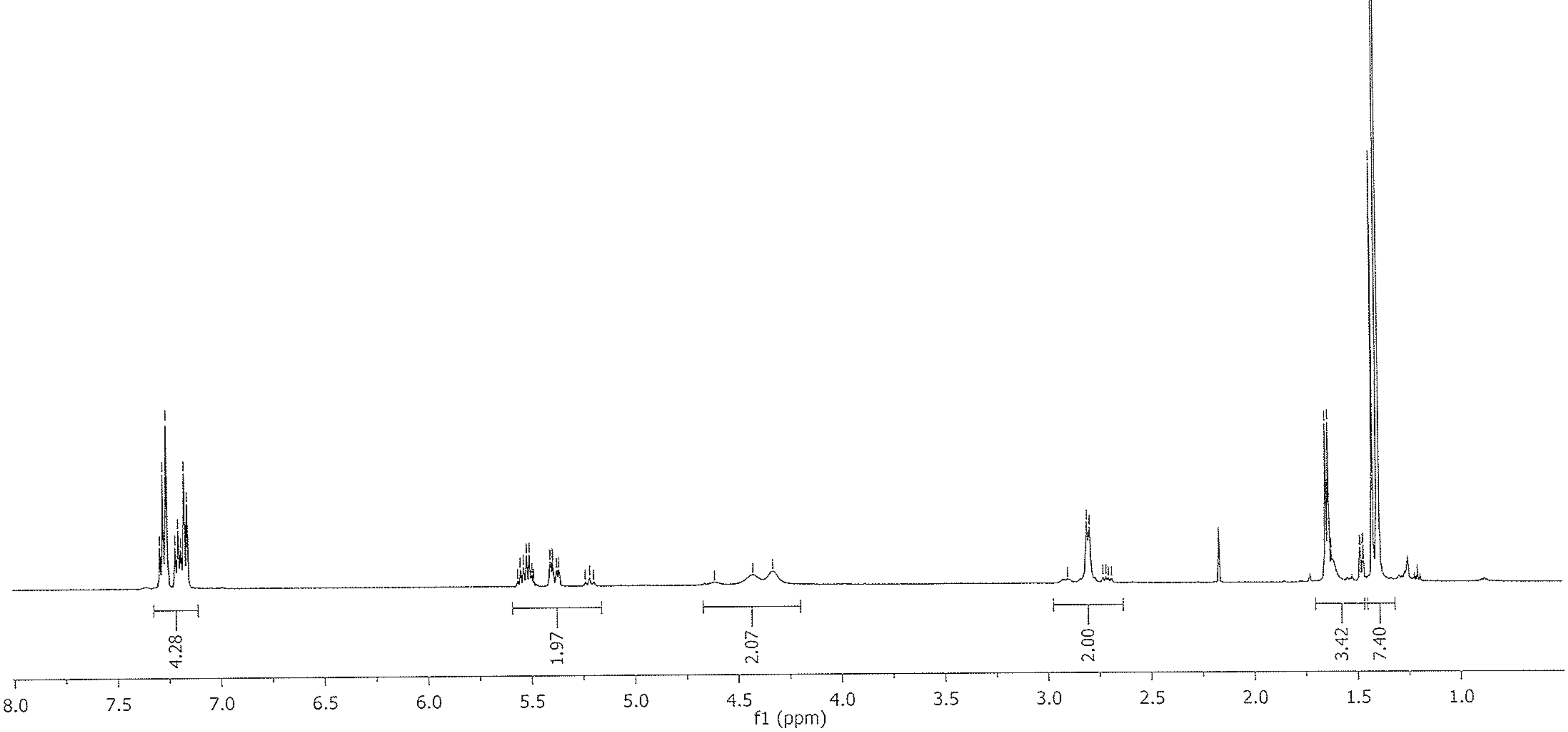

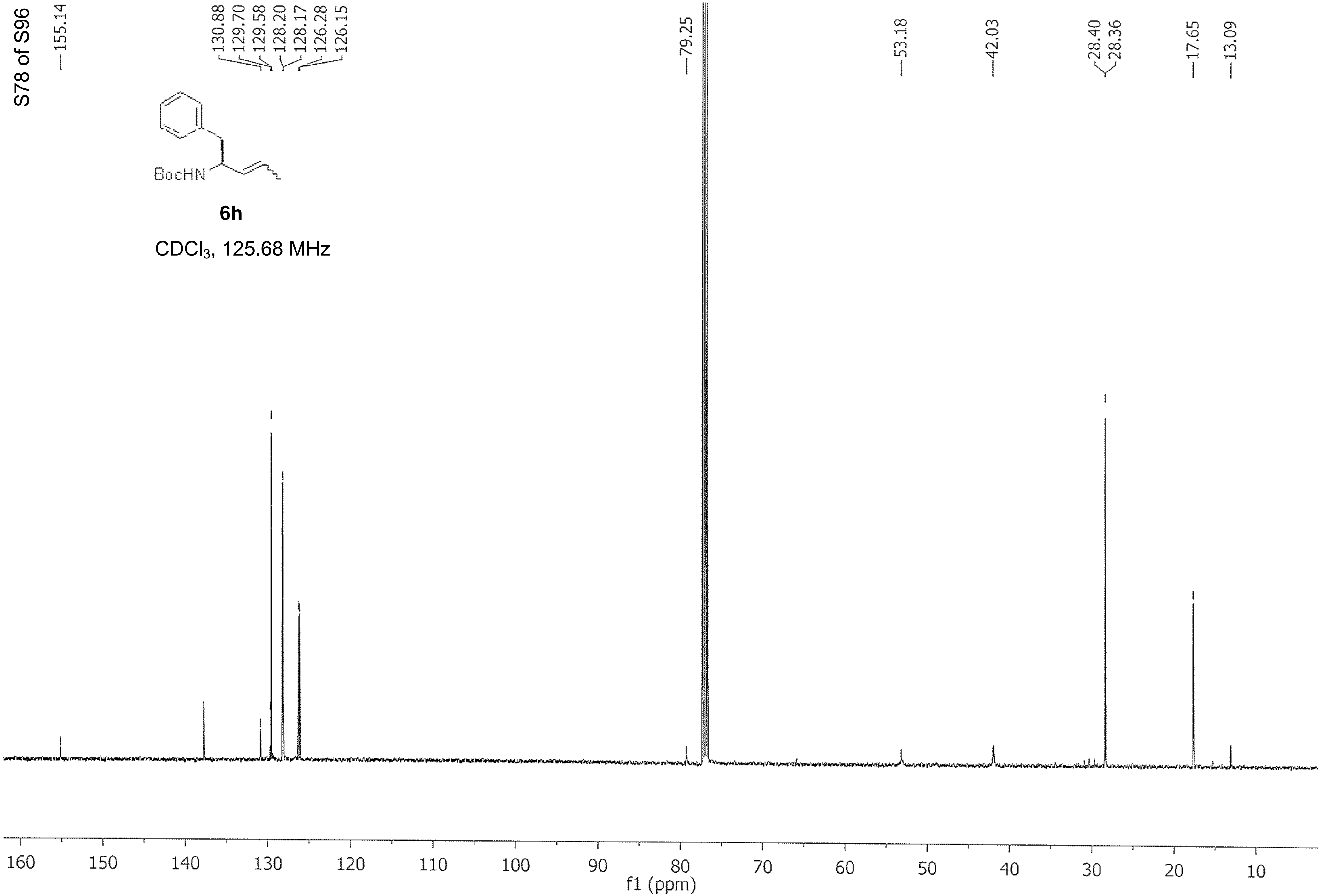
155.14
130.88
129.70
129.58
128.20
128.17
126.28
126.15
79.25
53.18
42.03
28.40
28.36
17.65
13.09
BocHN
6h
CDCl3, 125.68 MHz
160
150
140
130
120
110
100
90
80
70
60
50
40
30
20
10
f1 (ppm)

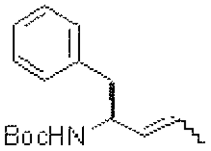

6h

CDCl$_3$, 500MHz

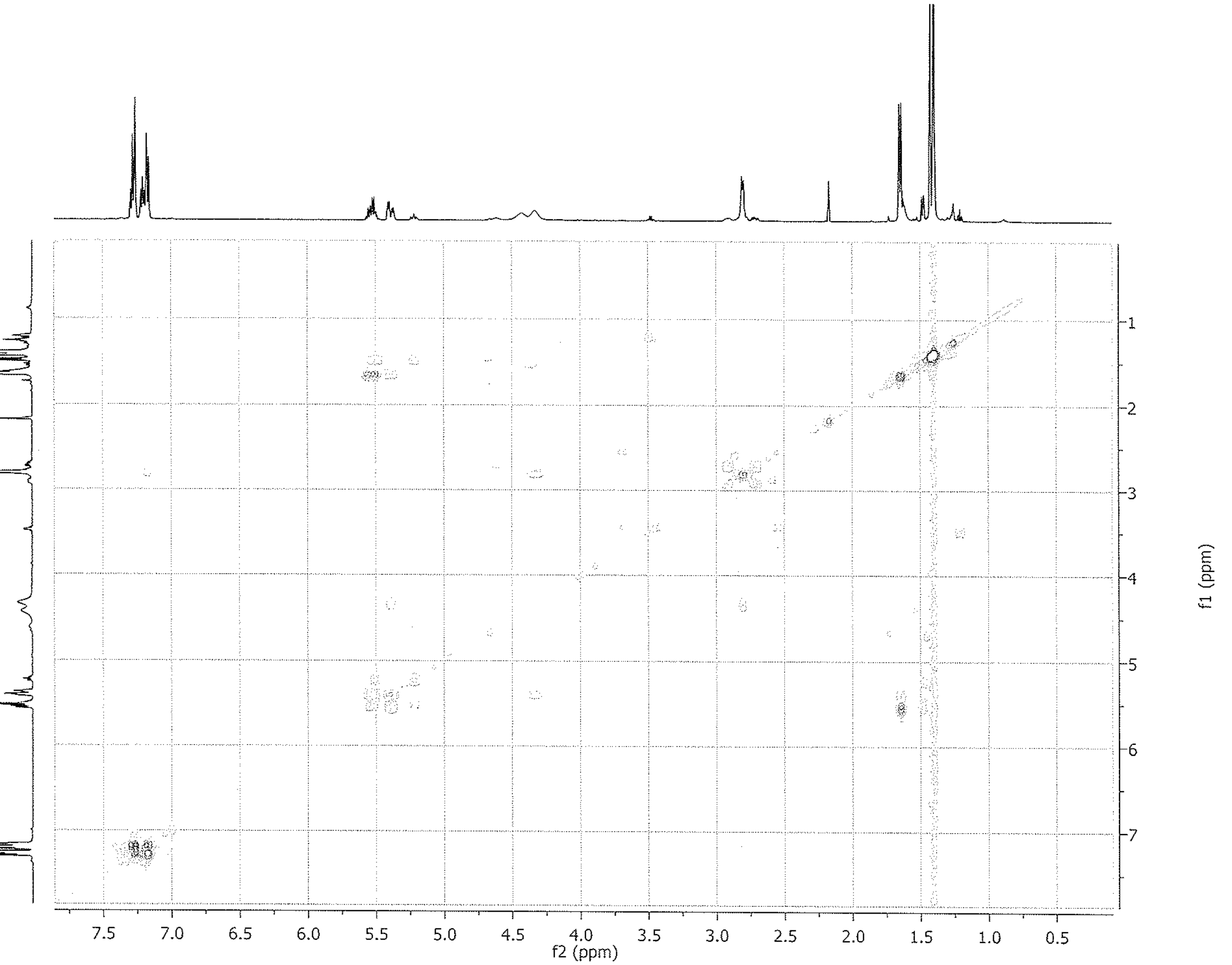

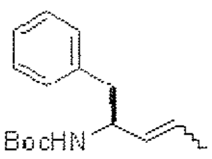

6h

$CDCl_3$

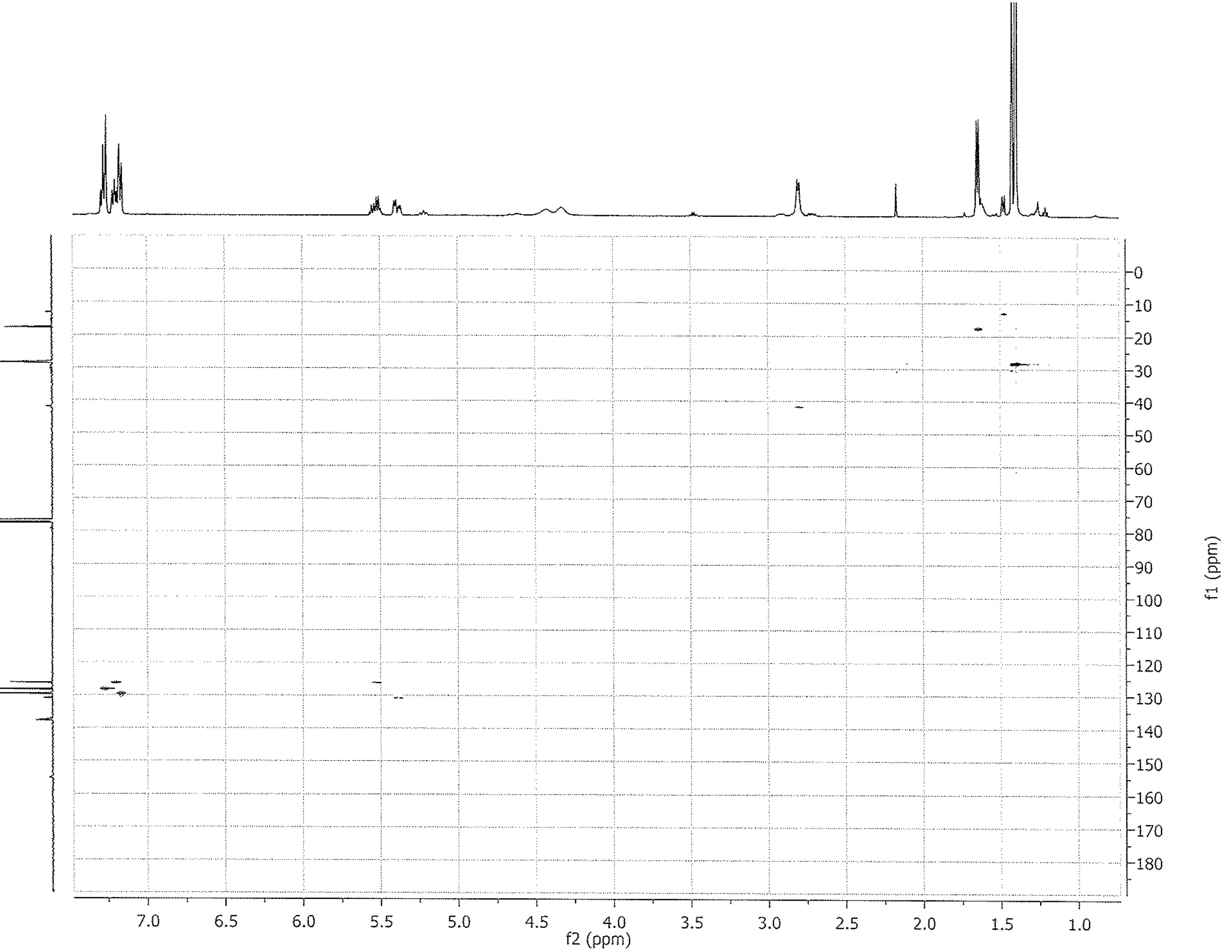

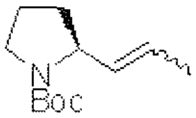

6i

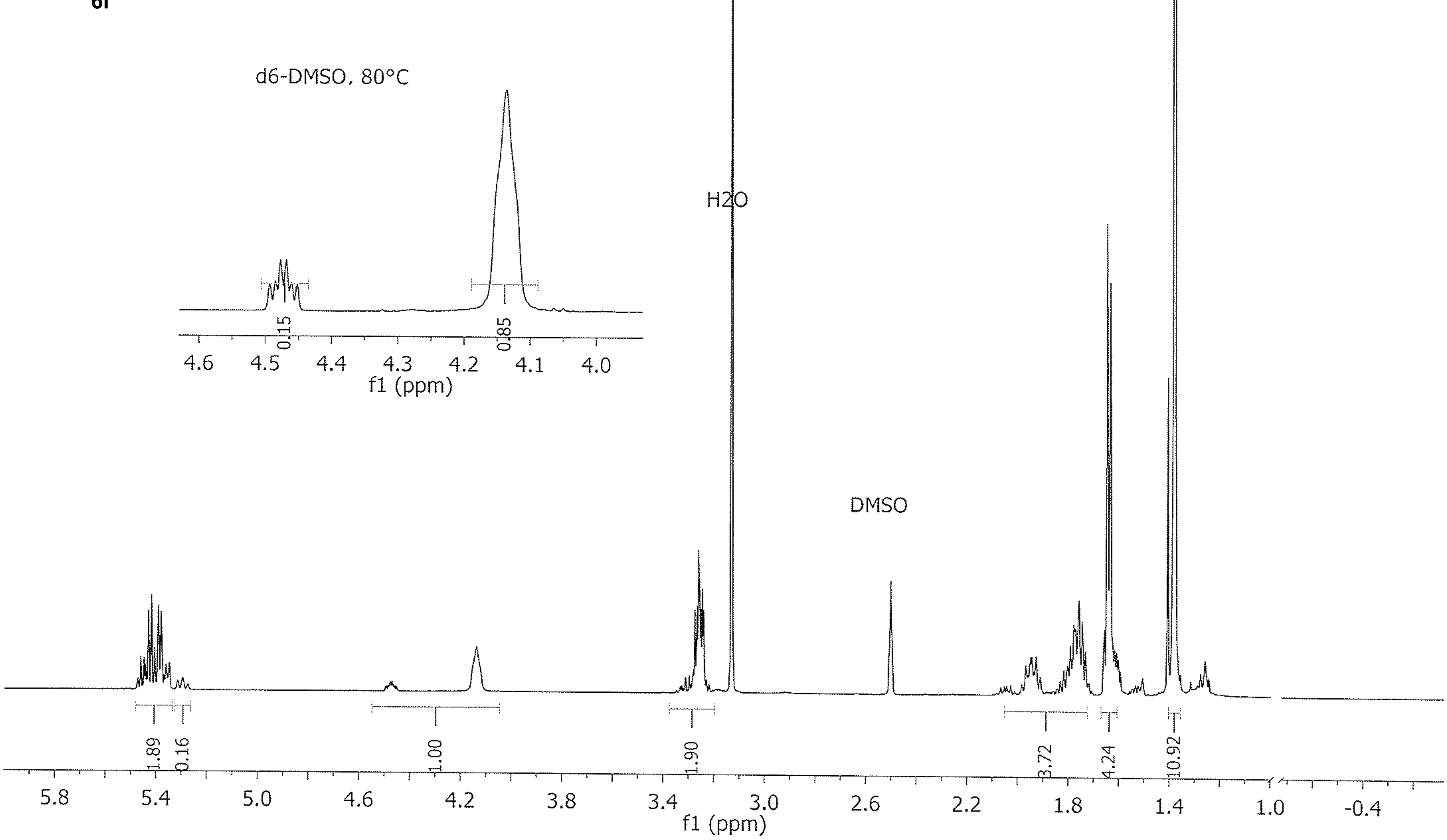
d6-DMSO, 80°C
0.15
0.85
4.6 4.5 4.4 4.3 4.2 4.1 4.0
f1 (ppm)
H2O
DMSO
1.89
0.16
1.00
1.90
3.72
4.24
10.92
5.8 5.4 5.0 4.6 4.2 3.8 3.4 3.0 2.6 2.2 1.8 1.4 1.0 -0.4
f1 (ppm)

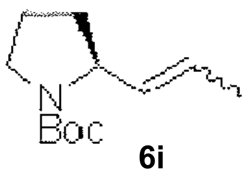

6i

d6-DMSO, 80 °C, 125.68 MHz

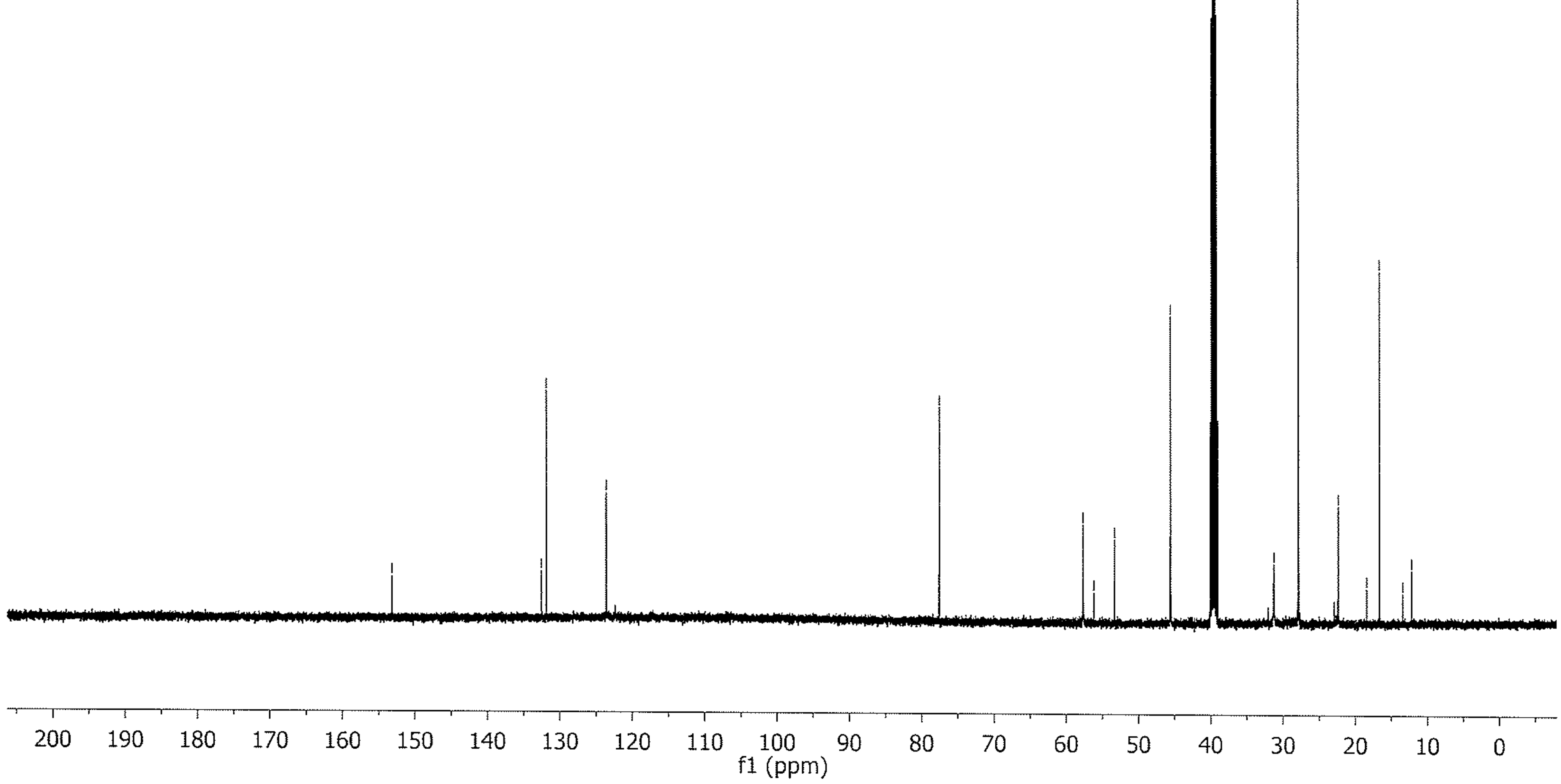

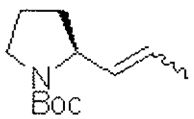

6i

d6-DMSO, 80 °C, 125.68 MHz

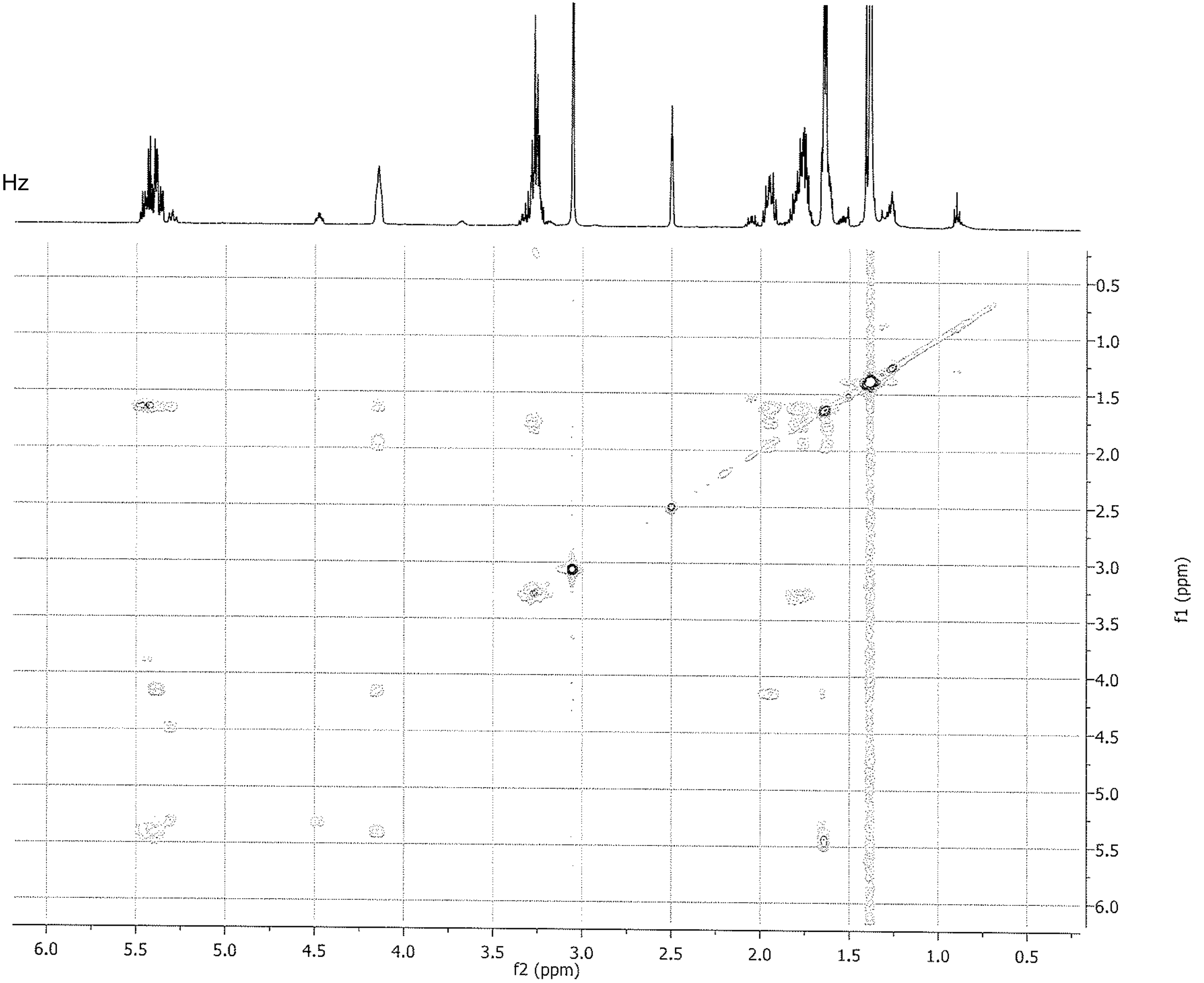

BocAllylAla-Racemo
g-cdx
90°C x 5'--> 1°C/min --> 150°C

File	:	c:\class-vp\chrom\lixa2
Method	:	c:\class-vp\methods\Fulvia2.met
Sample ID	:	LIX162-BocAllylAla
Acquired	:	May 22, 2012 04:02:06
Printed	:	Jul 31, 2012 01:50:24

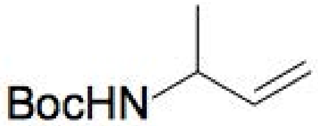

rac-6a

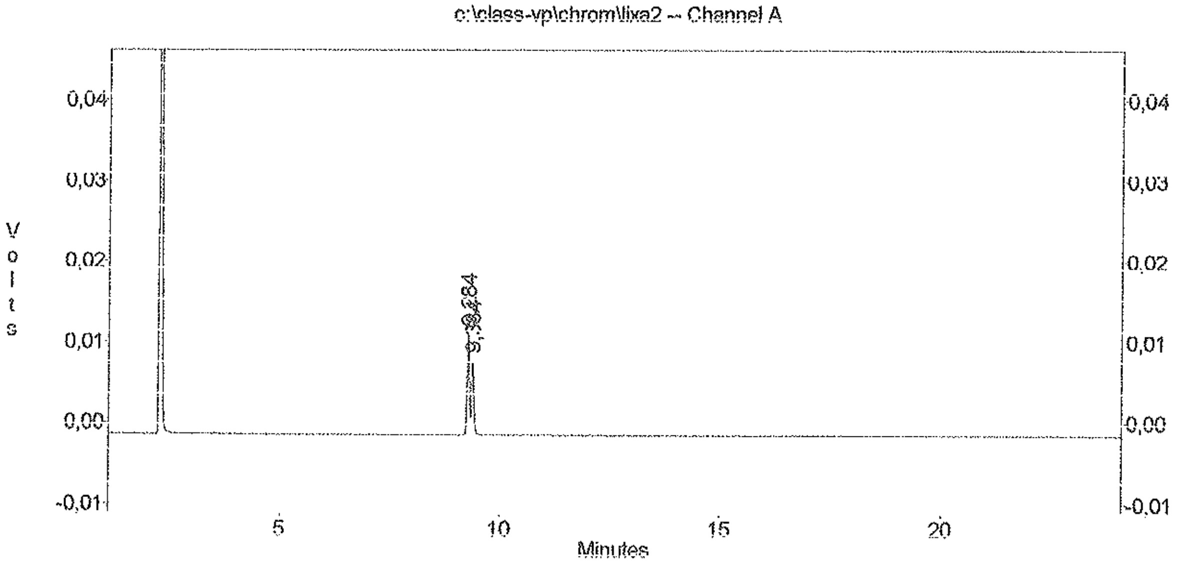

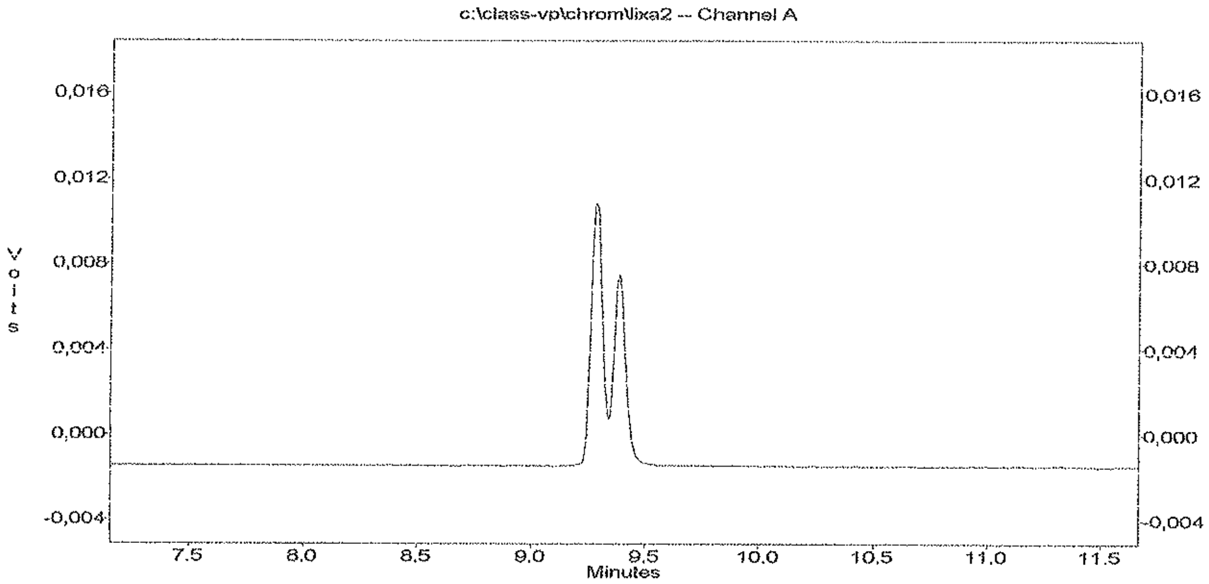

Channel A Results

Peak	Time	Area	Area %
1	9,28	41662	57,271
2	9,38	31083	42,729

Totals :

	72745	100,000

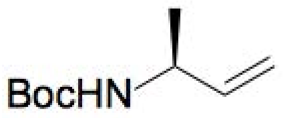

(*S*)-6a

BocAllylAla
g-cdx
90°C x 5'--> 1°C/min --> 150°C

File : c:\class-vp\chrom\lixa8
Method : c:\class-vp\methods\Fulvia2.met
Sample ID : LIX142
Acquired : May 23, 2012 04:11:49
Printed : Jul 31, 2012 01:45:06

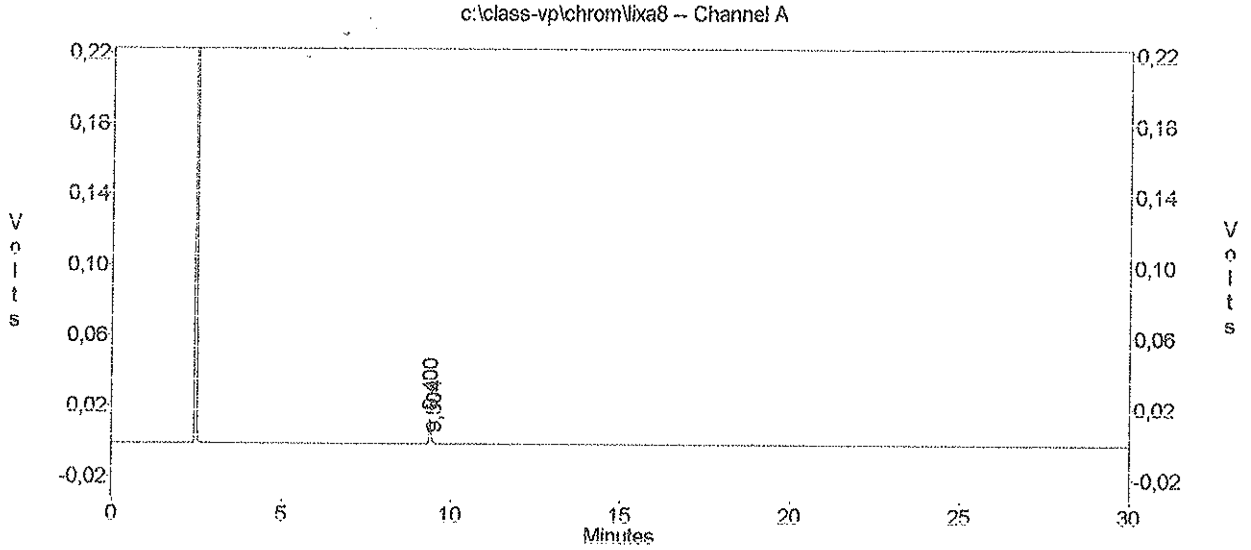

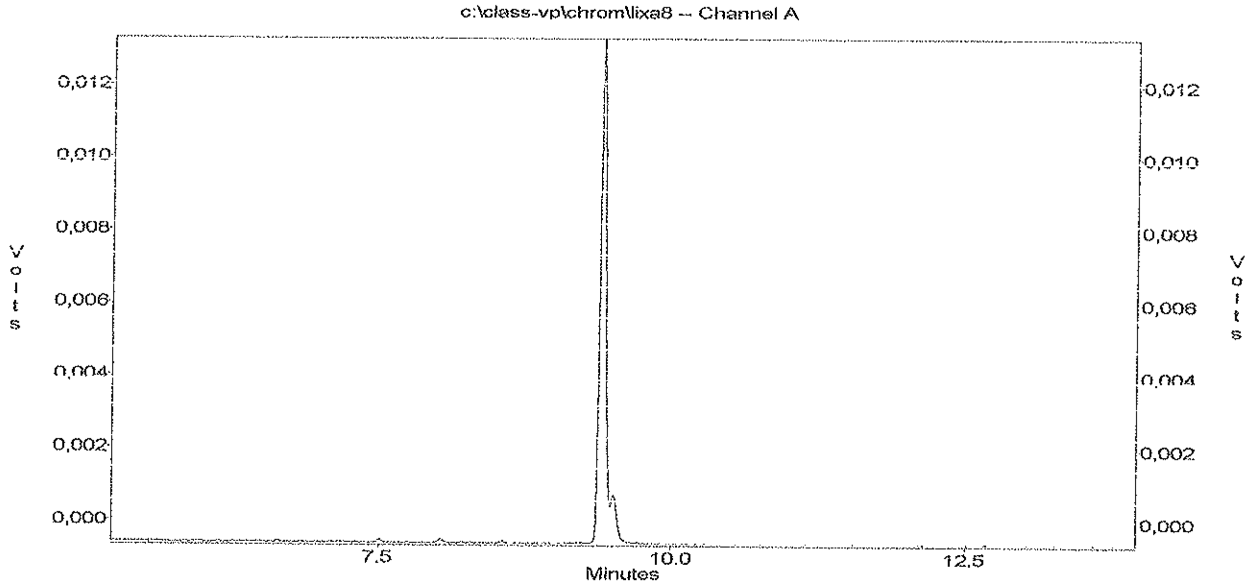

Channel A Results

Peak	Time	Area	Area %
1	9,40	44491	96,778
2	9,50	1481	3,222

Totals :

		45972	100,000

DL-BocAllylVal
β ▮-cdx 100°C x 5 min, 1°C/min -> 150°C

File : c:\class-vp\chrom\Mg11
Method : c:\class-vp\methods\Fulvia2.met
Sample ID : DL-BocAllylVal
Acquired : Jan 04, 1980 21:05:25
Printed : Jan 04, 1980 21:38:54

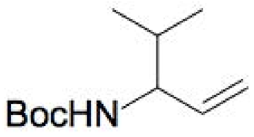

rac-6b

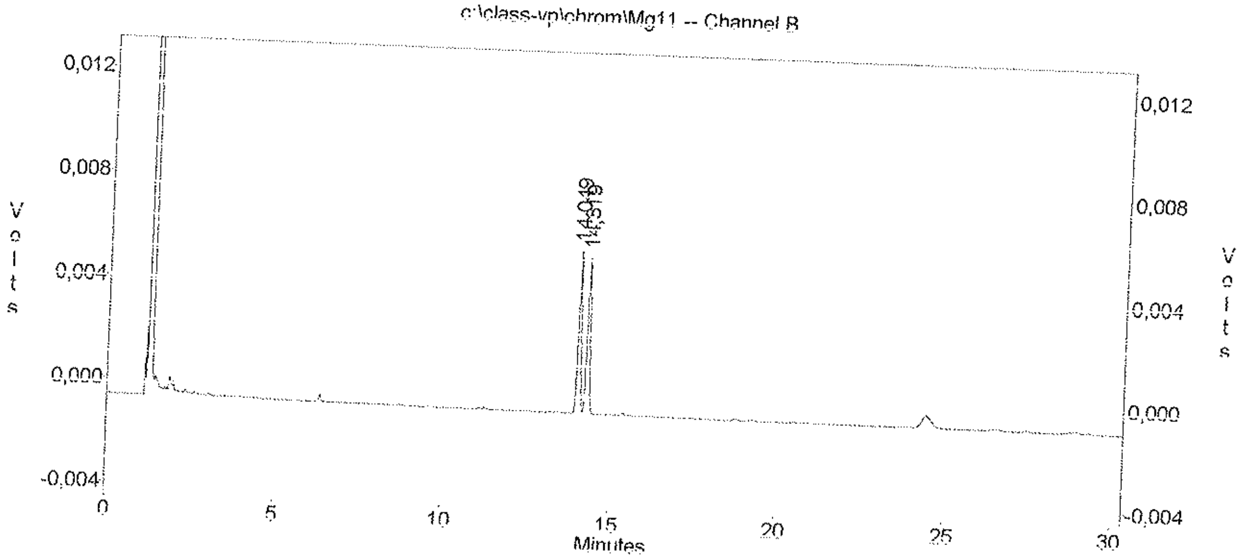

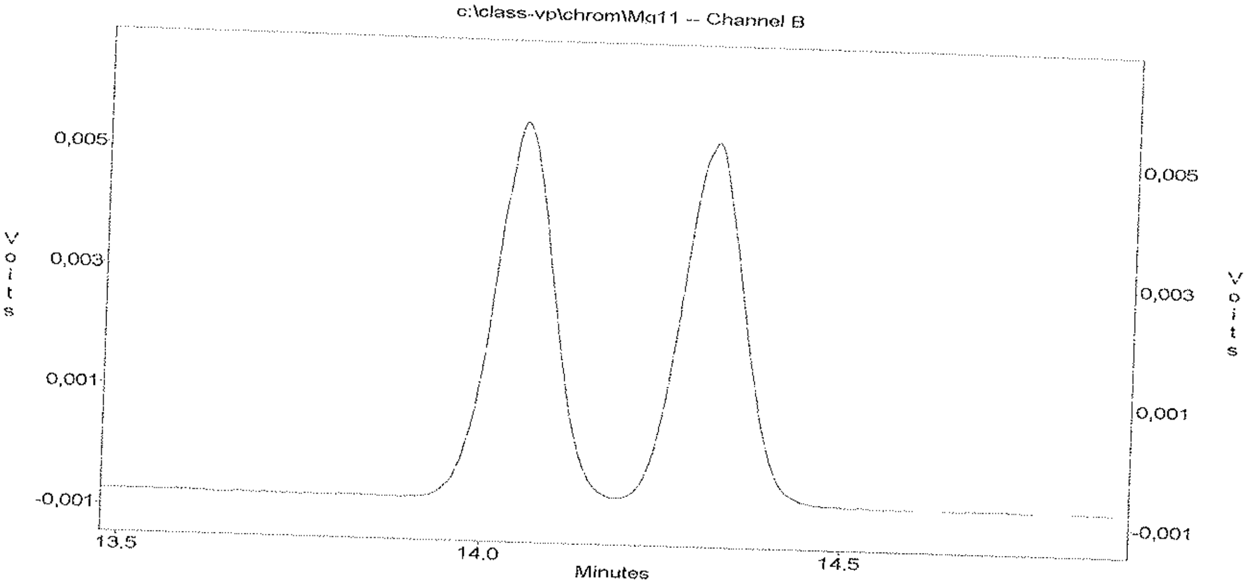

Channel B Results

Peak	Time	Area	Area %
1	14,05	35472	50,097
2	14,32	35334	49,903

Totals :

		70806	100,000

L-BocAllylVal

β-cdx 100°C x 5 min, 1°C/min -> 150 °C

File : c:\class-vp\chrom\Mg12
Method : c:\class-vp\methods\Fulvia2.met
Sample ID : L-BocAllylVal
Acquired : Jan 04, 1980 21:44:26
Printed : Jan 04, 1980 22:31:05

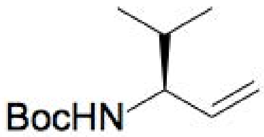

(S)-6b

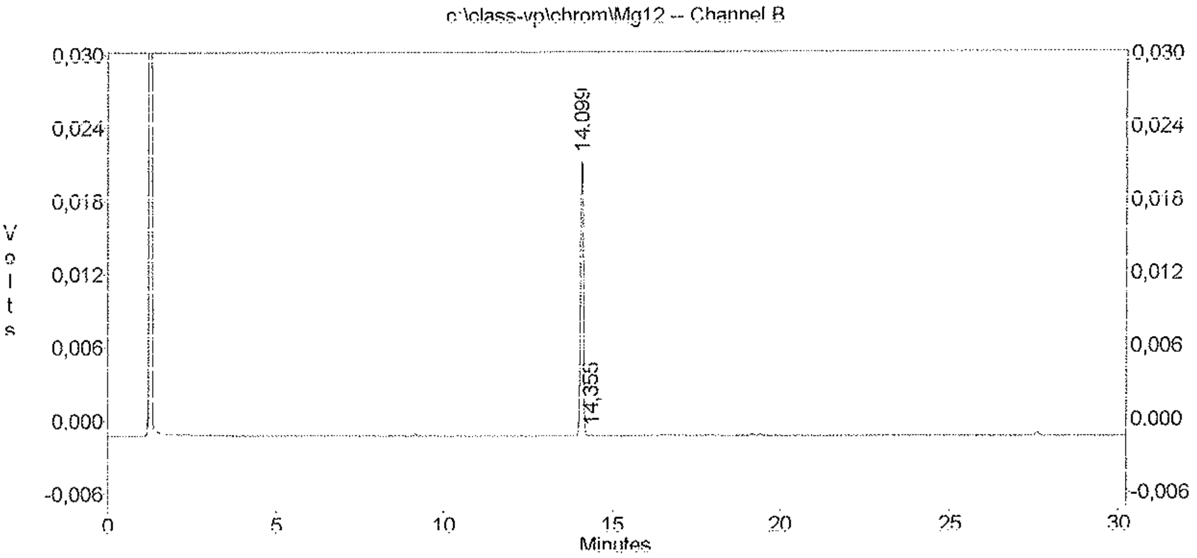

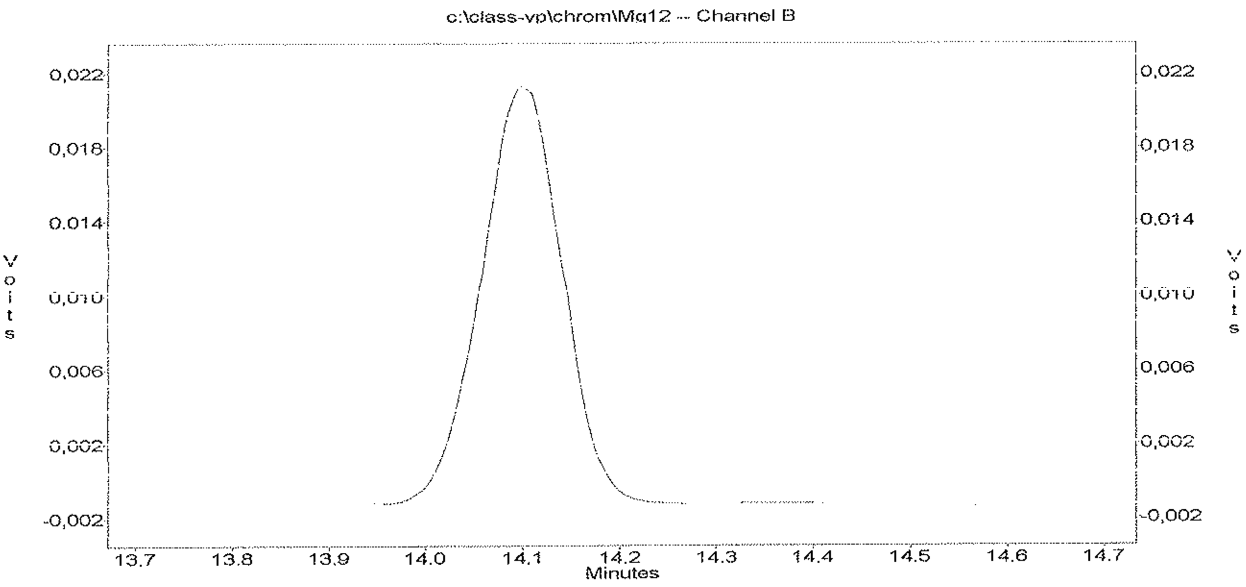

Channel B Results

Peak	Time	Area	Area %
1	14,10	129031	99,847
2	14,36	198	0,153

Totals :

		129229	100,000

DL-BocAllylLeu
β[illegible]-cdx 100°C x 5 min, 1°C/min -> 150°C

File : c:\class-vp\chrom\Mg13
Method : c:\class-vp\methods\Fulvia2.met
Sample ID : DL-BocAllylLeu
Acquired : Jan 04, 1980 22:33:48
Printed : Jan 04, 1980 22:59:46

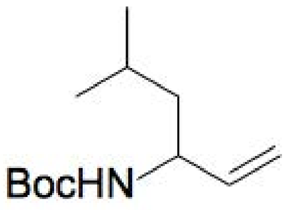

rac-6c

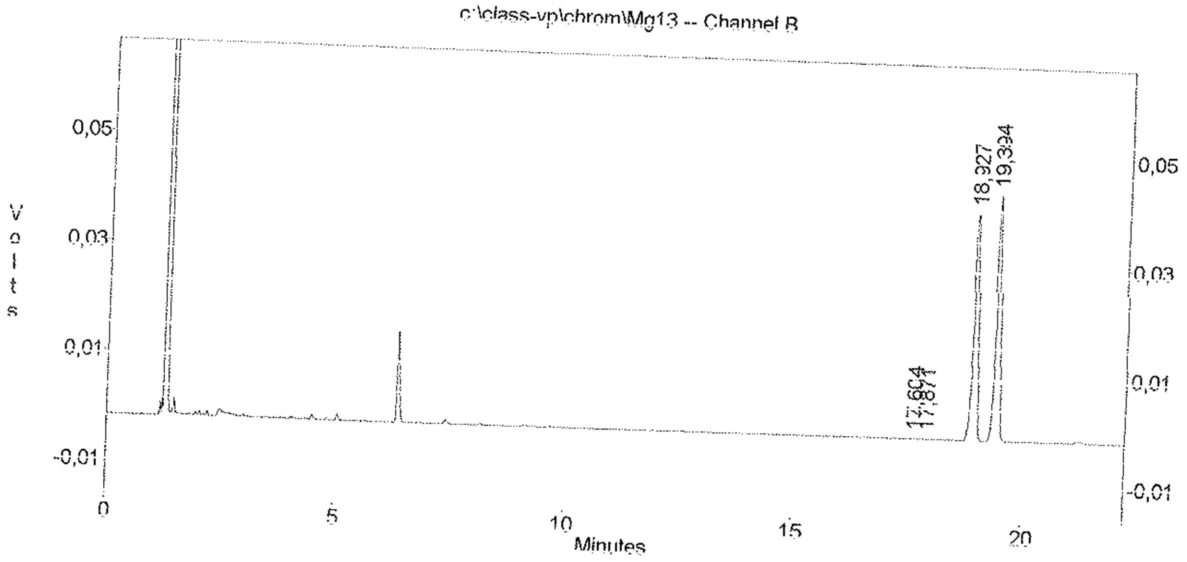

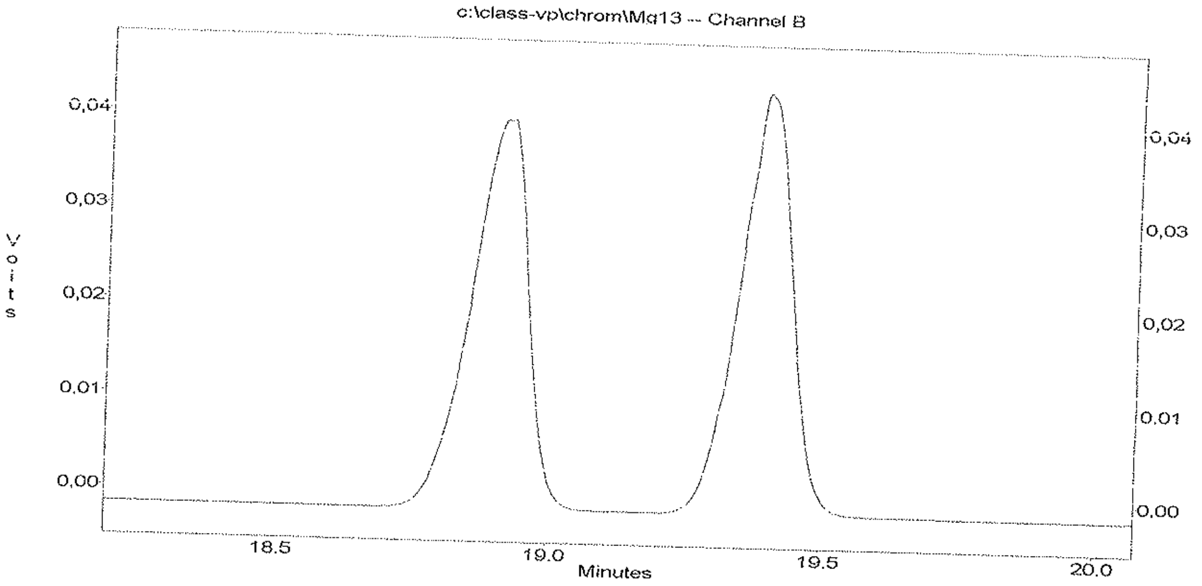

Channel B Results

Peak	Time	Area	Area %
1	17,60	354	0,059
2	17,87	466	0,078
3	18,93	296546	49,821
4	19,39	297851	50,041

Continued...

L-BocAllylLeu
βg-cdx 100°C x 5 min, 1°C/min -> 150°C

File	:	c:\class-vp\chrom\Mg14
Method	:	c:\class-vp\methods\Fulvia2.met
Sample ID	:	L-BocAllylLeu
Acquired	:	Jan 04, 1980 23:04:34
Printed	:	Jan 05, 1980 00:32:41

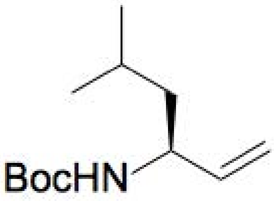

(S)-6c

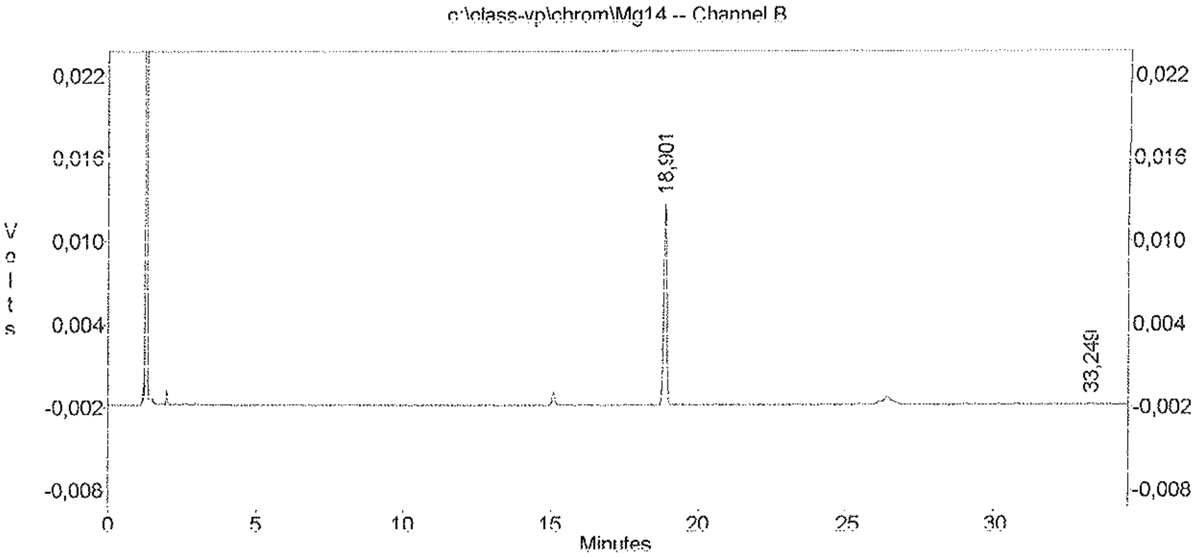

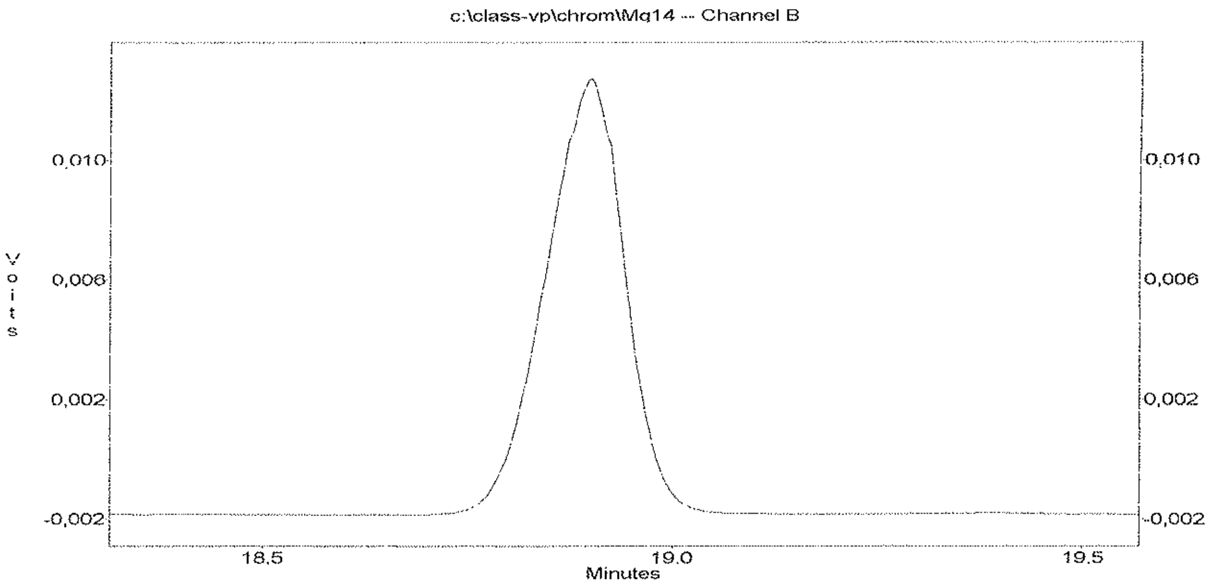

Channel B Results

Peak	Time	Area	Area %
1	18,90	96838	99,620
2	33,25	369	0,380

Totals :

	97207	100,000

DL-BocAllylIle

β-cdx 100°C x 5 min, 1°C/min ->150°C

File : c:\class-vp\chrom\Mg9
Method : c:\class-vp\methods\Fulvia2.met
Sample ID :
Acquired : Jan 04, 1980 20:02:01
Printed : Jan 04, 1980 20:32:02

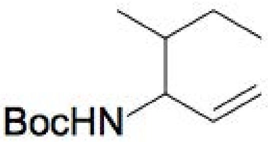

rac-6d

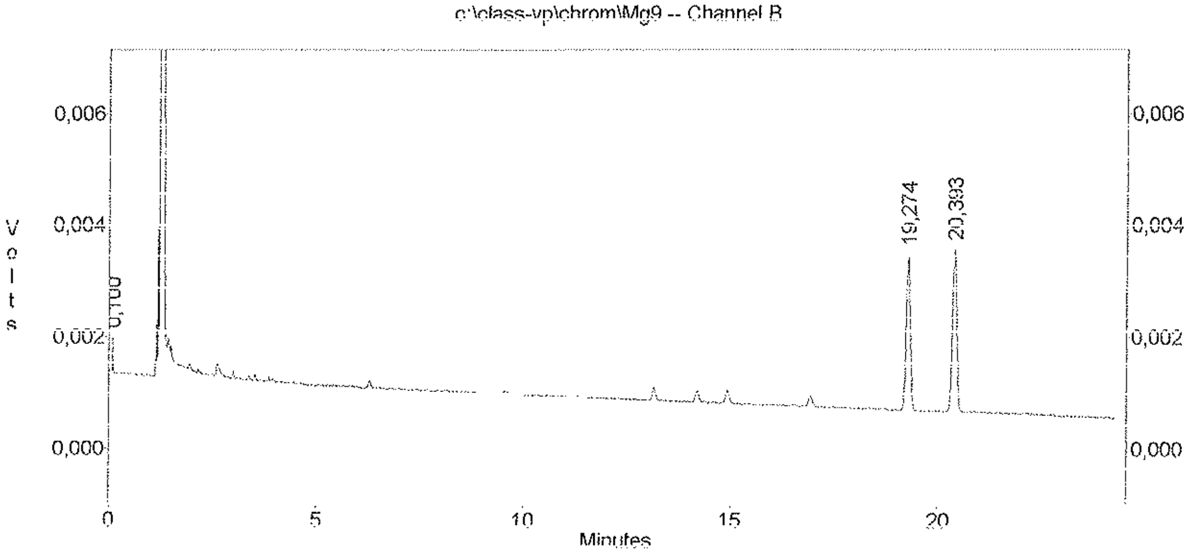

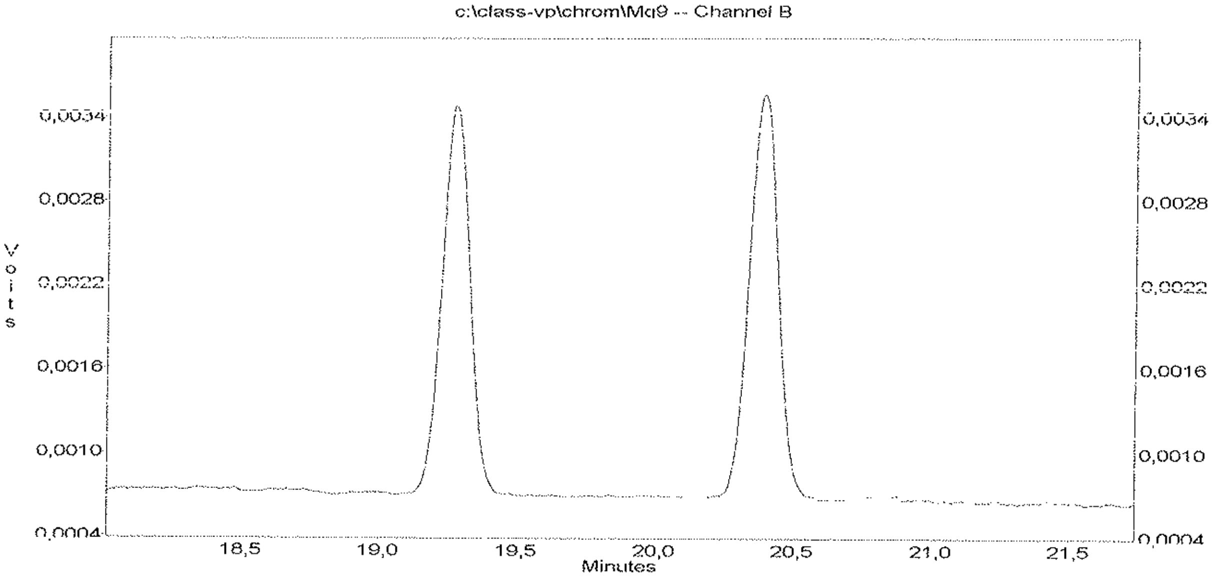

Channel B Results

Peak	Time	Area	Area %
1	0,10	419	1,029
2	19,27	19240	47,231
3	20,39	21077	51,740

Totals :

		40736	100,000

L-BocAllylIle

β-cdx 100°C x 5 min, 1°C/min -> 150°C

File	:	c:\class-vp\chrom\Mg10
Method	:	c:\class-vp\methods\Fulvia2.met
Sample ID	:	L-BocAllylIle
Acquired	:	Jan 04, 1980 20:35:40
Printed	:	Jan 04, 1980 21:01:16

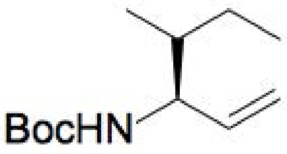

(S)-6d

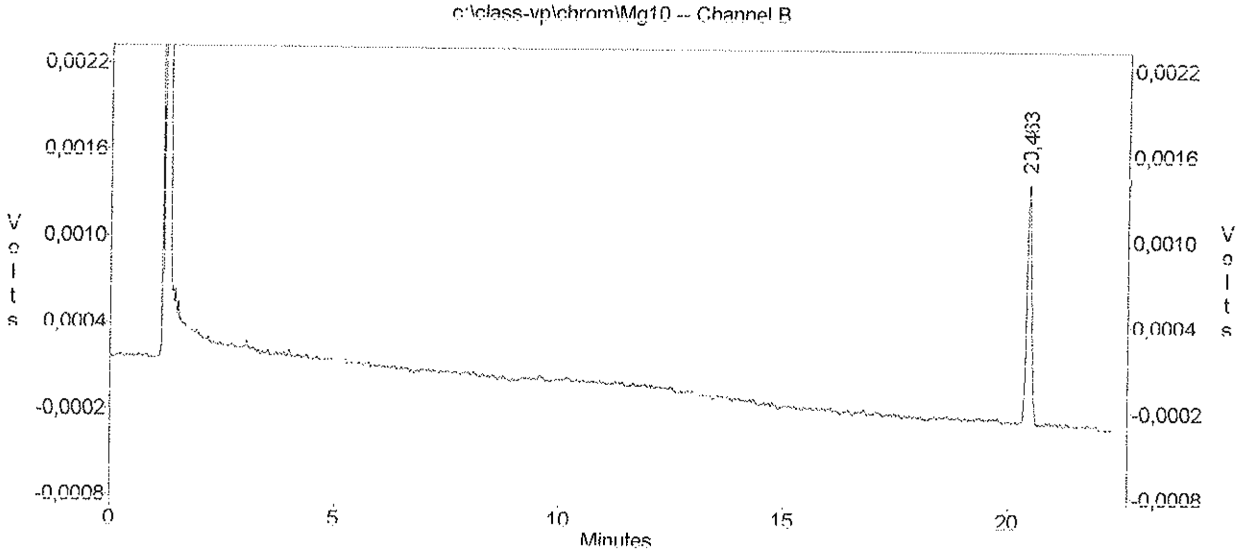

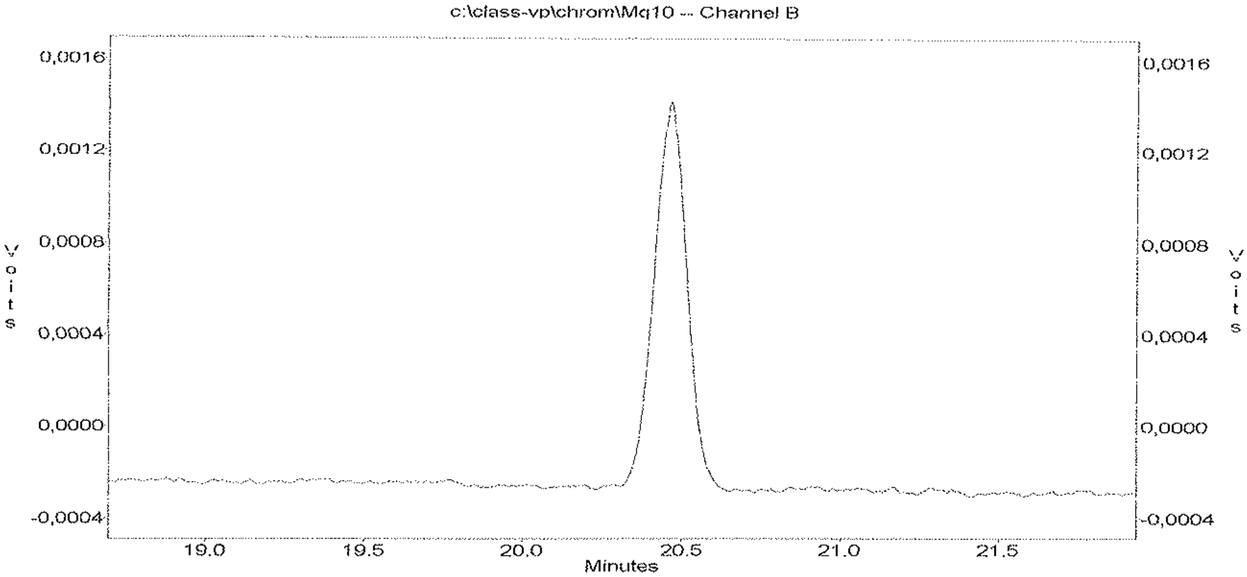

Channel B Results

Peak	Time	Area	Area %
1	20,46	12041	100,000
Totals :		12041	100,000

DL-BocAllylPhe
g-cdx isoterma 150°C
File : c:\class-vp\chrom\Mg7
Method : c:\class-vp\methods\Fulvia2.met
Sample ID :

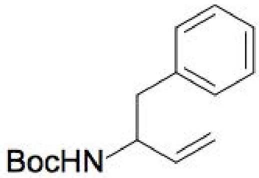

rac-6e

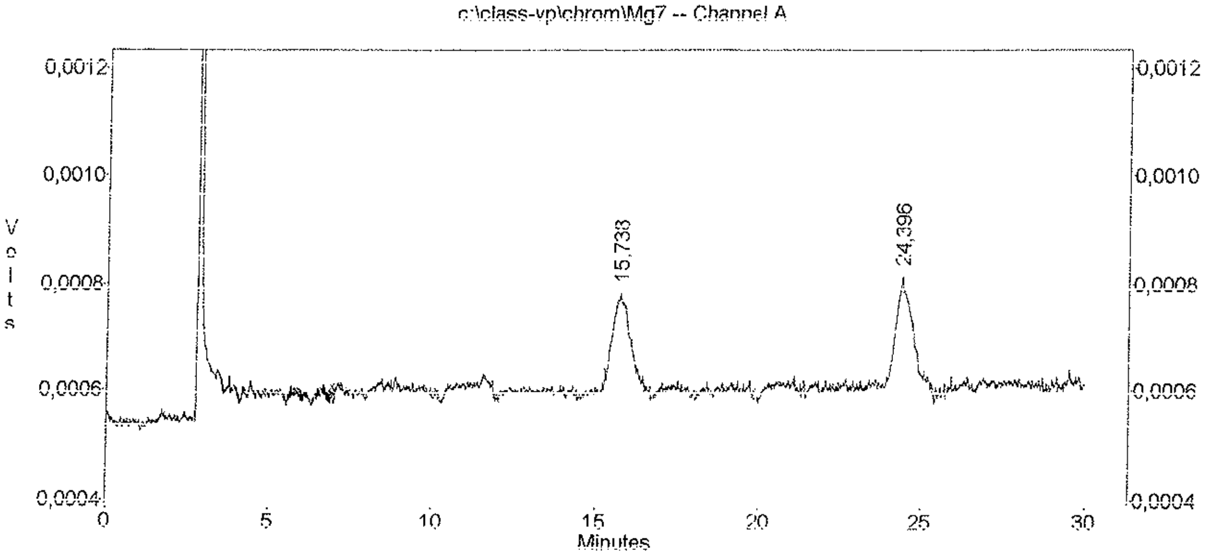

L-BocAllylPhe
g-cdx isoterma 150°C
File : c:\class-vp\chrom\Mg8
Method : c:\class-vp\methods\Fulvia2.met
Sample ID : L-AllylPhe

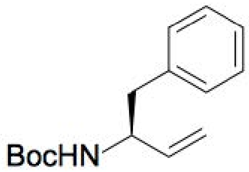

(S)-6e

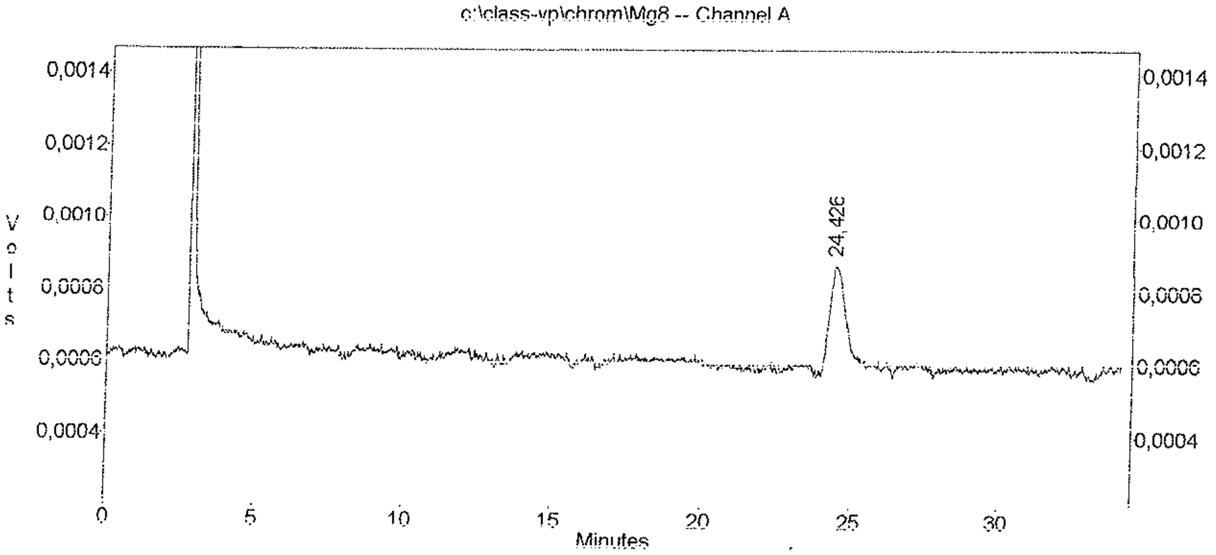

DL-BocAllylPro
b-cdx isoterma 100°C

File : c:\class-vp\chrom\Mg5
Method : c:\class-vp\methods\Fulvia2.met
Sample ID : DL-BocAllylPro bcd
Acquired : Aug 09, 2012 09:42:00
Printed : Aug 09, 2012 10:21:35

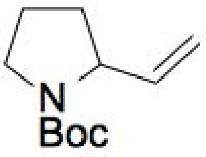

rac-6g

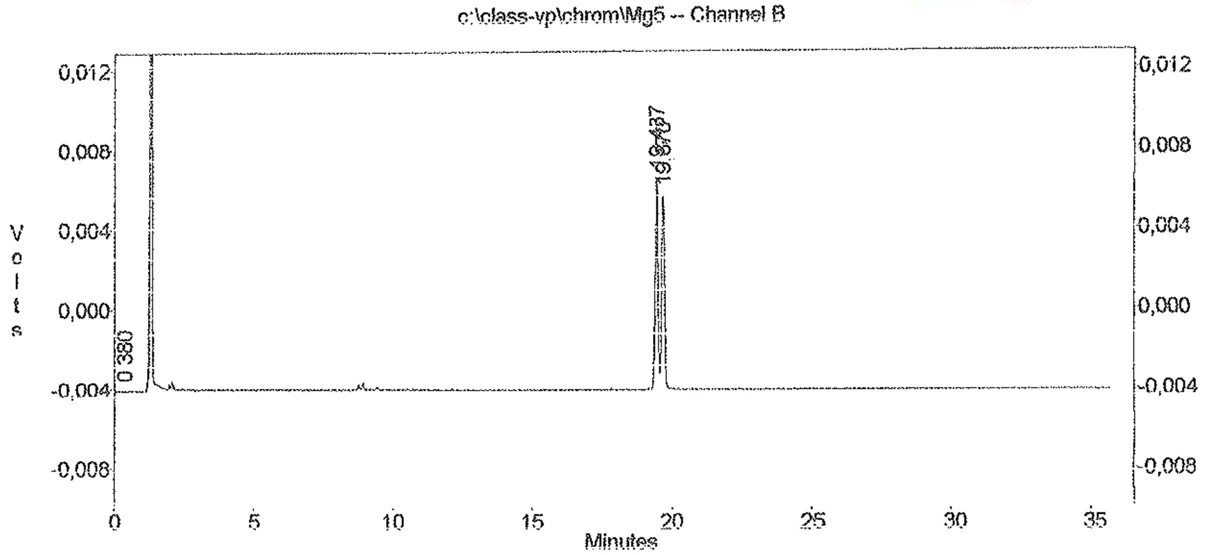

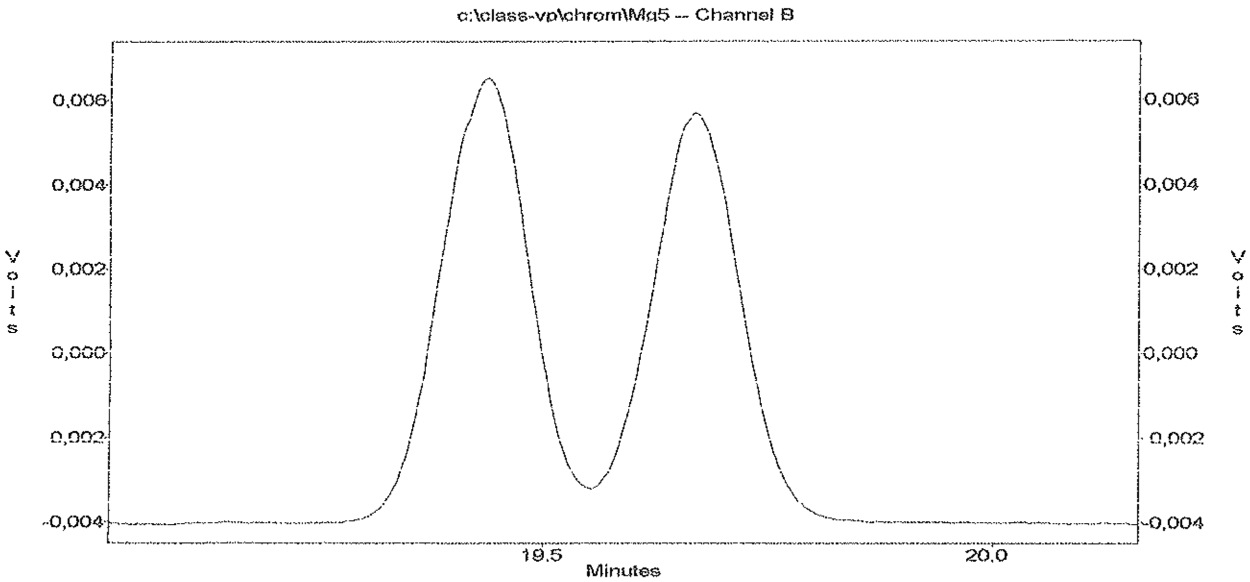

Channel B Results

Peak	Time	Area	Area %
1	0,38	107	0,084
2	19,44	66321	52,134
3	19,67	60785	47,782

Totals :

		127213	100,000

L-BocAllylPro
b-cdx isoterma 100°C

File : c:\class-vp\chrom\Mg6
Method : c:\class-vp\methods\Fulvia2.met
Sample ID : L-BocAllylPro
Acquired : Aug 09, 2012 10:25:15
Printed : Aug 09, 2012 10:58:15

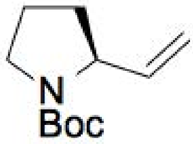

(*S*)-6g

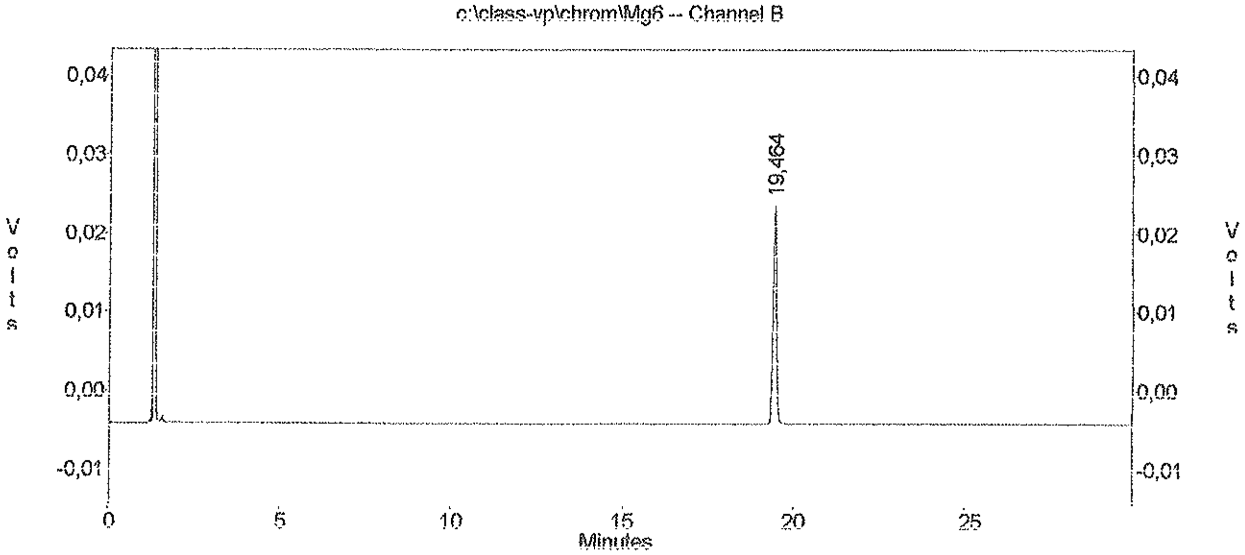

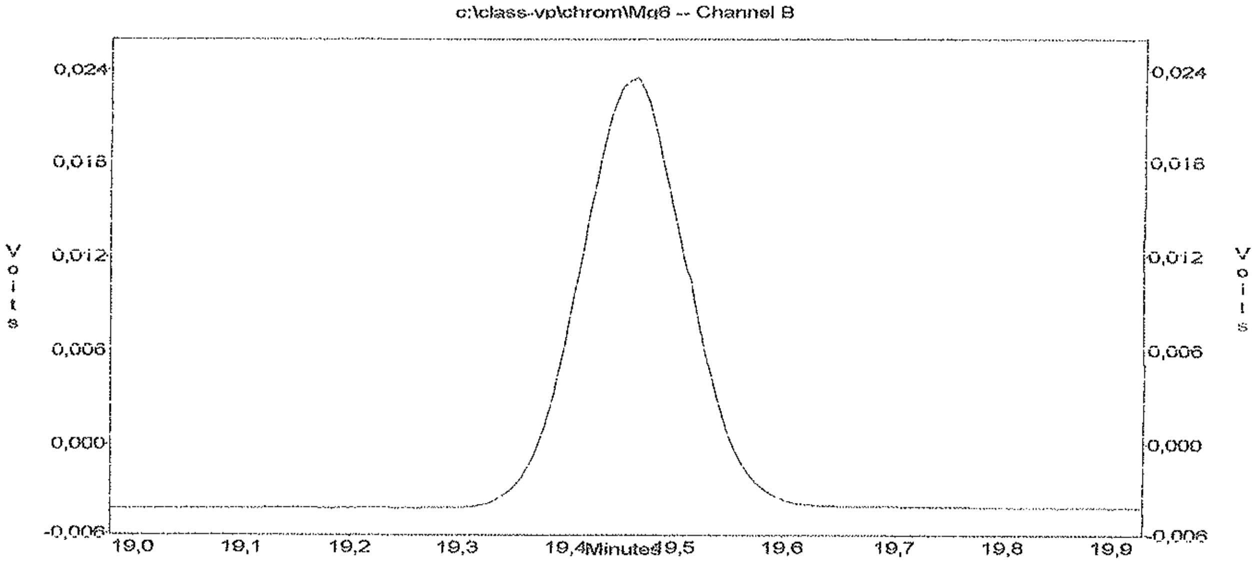

Channel B Results

Peak	Time	Area	Area %
1	19,46	188661	100,000

Totals :

	188661	100,000

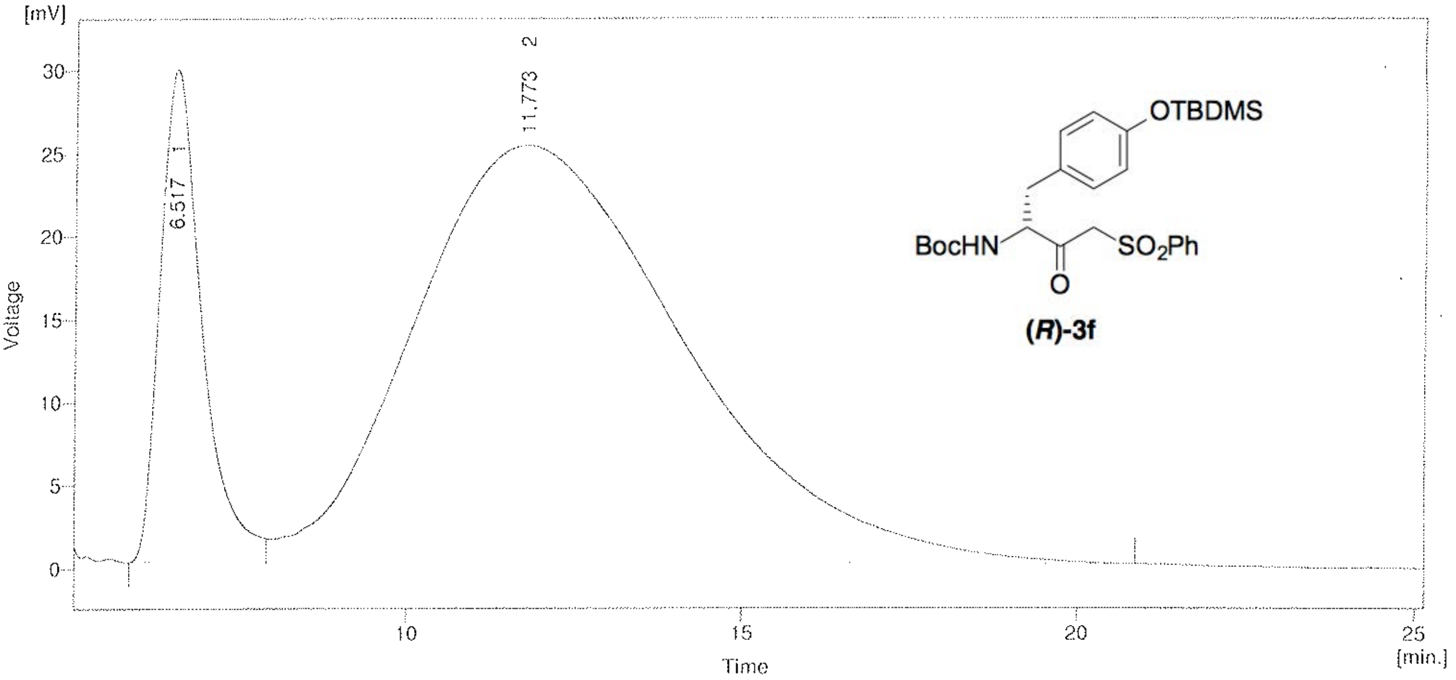

Result Table

	Reten. Time [min]	Area [mV.s]	Height [mV]	Area [%]	Height [%]	W05 [min]
1	6.517	1293.477	29.654	15.5	54.1	0,64
2	11.773	7034.776	25.134	84.5	45.9	4.32
	Total	8328.253	54.788	100.0	100.0	

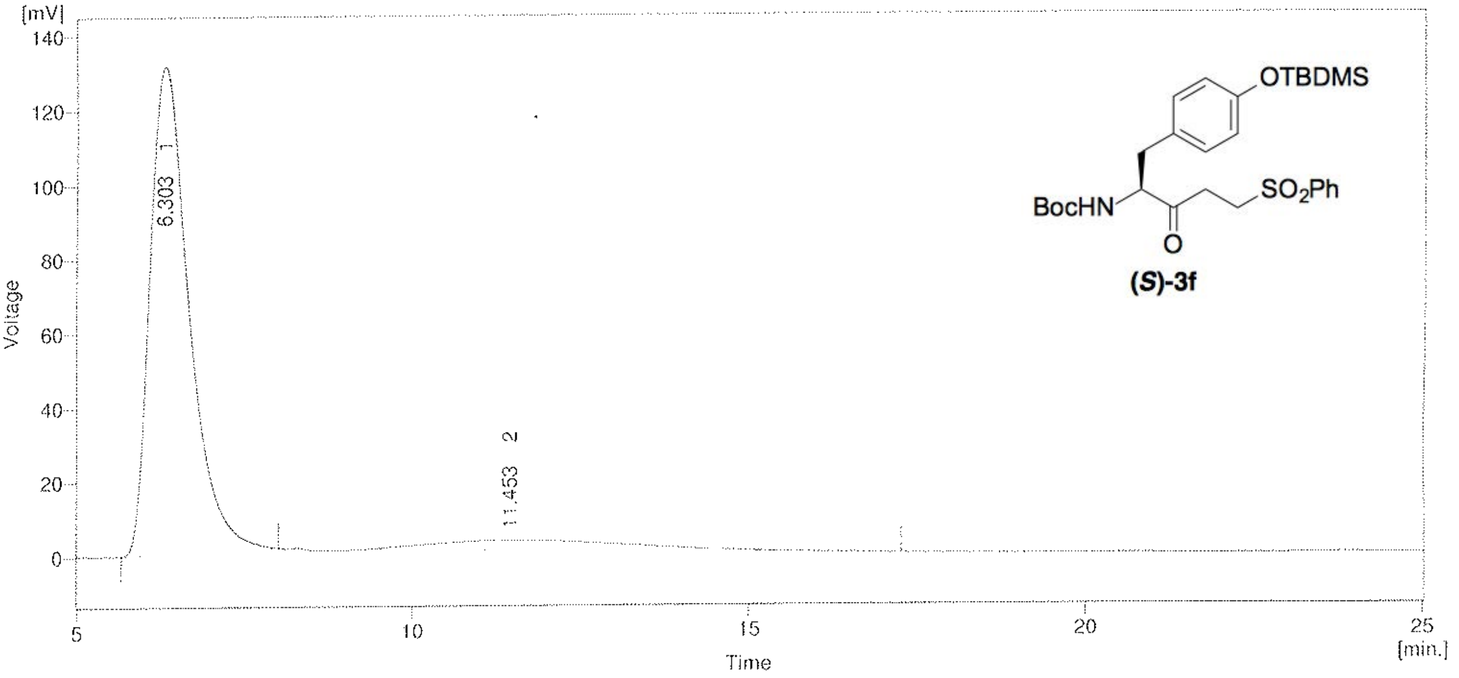

Result Table

	Reten. Time [min]	Area [mV.s]	Height [mV]	Area [%]	Height [%]	W05 [min]
1	6.303	5242.714	130.997	90.7	98.1	0,61
2	11.453	536.721	2.527	9.3	1.9	3.24
	Total	5779.435	133.524	100.0	100.0	

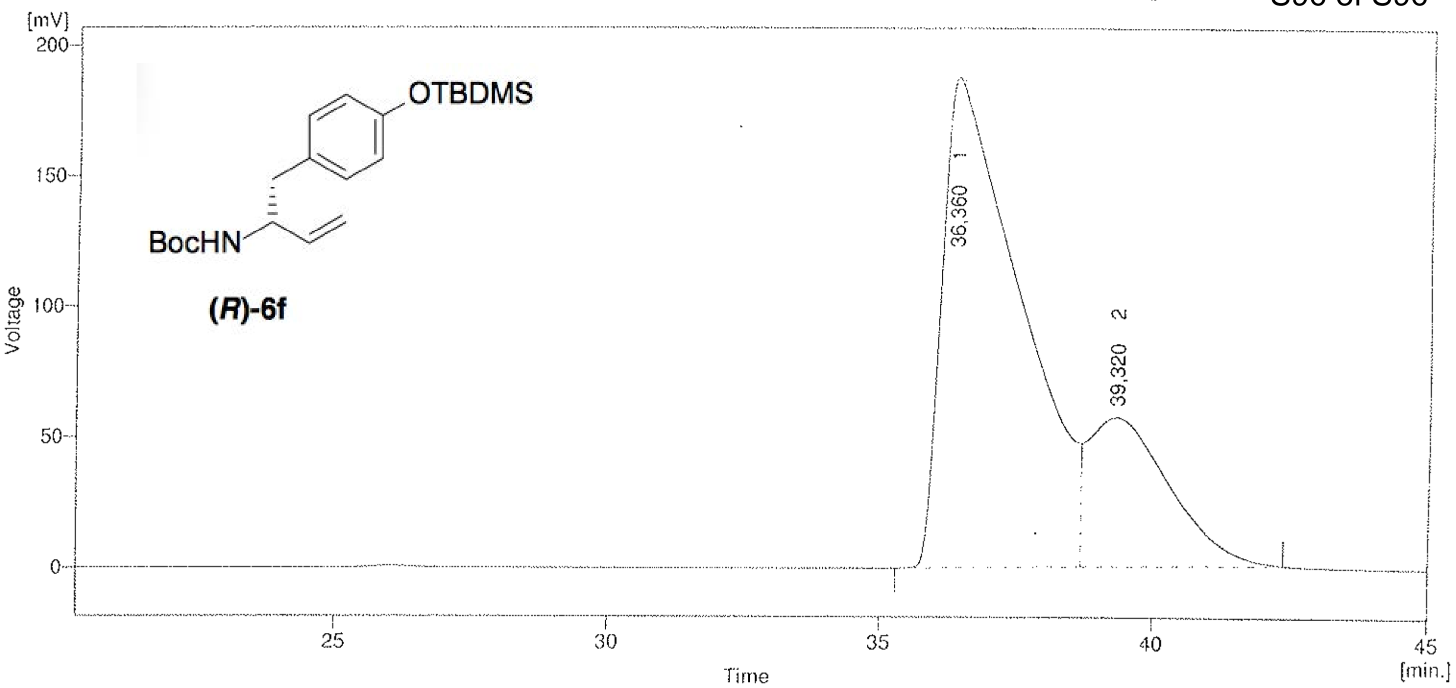

Result Table

	Reten. Time [min]	Area [mV.s]	Height [mV]	Area [%]	Height [%]	W05 [min]
1	36,360	18656,150	187,571	76,0	76,7	1,62
2	39,320	5896,603	57,075	24,0	23,3	1,69
	Total	24552,753	244,646	100,0	100,0	

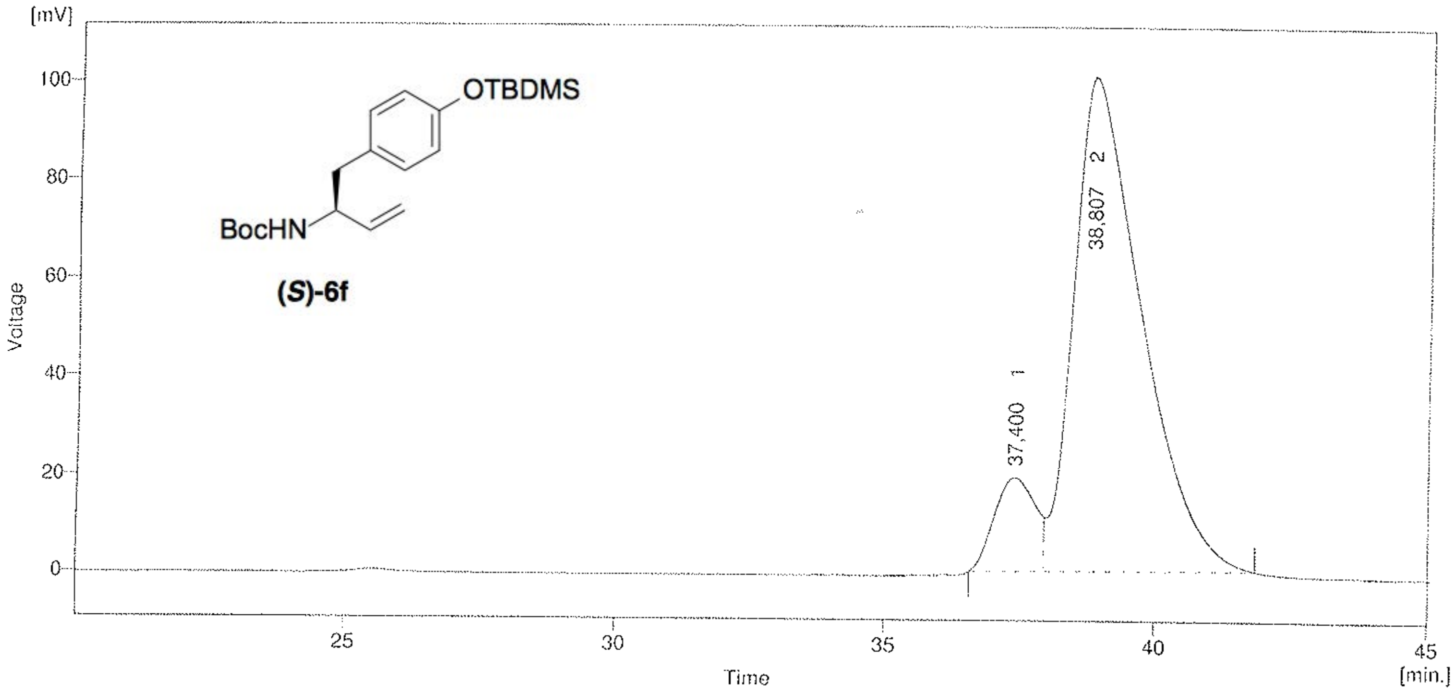

Result Table

	Reten. Time [min]	Area [mV.s]	Height [mV]	Area [%]	Height [%]	W05 [min]
1	37,400	1004,754	19,350	10,2	16,1	0,97
2	38,807	8819,375	100,597	89,8	83,9	1,34
	Total	9824,129	119,947	100,0	100,0	
